# Prenatal Phenol and Paraben Exposures and Adverse Birth Outcomes: A Prospective Analysis of U.S. Births

**DOI:** 10.1016/j.envint.2023.108378

**Published:** 2023-12-12

**Authors:** Leonardo Trasande, Morgan E. Nelson, Akram Alshawabkeh, Emily S. Barrett, Jessie P. Buckley, Dana Dabelea, Anne L. Dunlop, Julie B. Herbstman, John D. Meeker, Mrudula Naidu, Craig Newschaffer, Amy M. Padula, Megan E. Romano, Douglas M. Ruden, Sheela Sathyanarayana, Susan L. Schantz, Anne P. Starling, Taylor Etzel, Ghassan B. Hamra, collaborators in the NIH Environmental influences on Child Health Outcomes Program

**Affiliations:** a Department of Pediatrics, Division of Environmental Pediatrics, NYU Grossman School of Medicine, New York, NY, USA; b Department of Population Health, NYU Grossman School of Medicine, New York, NY, USA; c NYU Wagner School of Public Service, New York, NY, USA; d RTI International, Research Triangle Park, NC, USA; e Northeastern University, Boston, MA, USA; f Department of Biostatistics and Epidemiology, Rutgers School of Public Health, Environmental and Occupational Health Sciences Institute, Piscataway, NJ, USA; g Department of Environmental Health and Engineering, Johns Hopkins Bloomberg School of Public Health, Baltimore, MD, USA; h Lifecourse Epidemiology Adiposity and Diabetes (LEAD) Center, University of Colorado Anschutz Medical Campus, Aurora, CO, USA; i Department of Gynecology and Obstetrics, Emory University School of Medicine, Atlanta, GA, USA; j Department of Environmental Health Sciences, Columbia University Mailman School of Public Health, New York, NY, USA; k Department of Environmental Health Sciences, University of Michigan School of Public Health, Ann Arbor, MI, USA; l College of Human Health and Development, Penn State University, Hershey, PA, USA; m Department of Obstetrics, Gynecology and Reproductive Sciences, University of California, San Francisco, San Francisco, CA, USA; n Department of Epidemiology, Geisel School of Medicine at Dartmouth, Lebanon, NH, USA; o Department of Obstetrics and Gynecology, Wayne State University, Detroit, MI 48201; p Seattle Children’s Research Institute, Seattle, WA, USA; q Department of Pediatrics, University of Washington, Seattle, WA, USA; r Beckman Institute for Advanced Science and Technology, University of Illinois Urbana-Champaign, Urbana, IL; s Department of Epidemiology, Gillings School of Global Public Health, University of North Carolina at Chapel Hill, Chapel Hill, NC, USA; t Department of Epidemiology, Johns Hopkins Bloomberg School of Public Health, Baltimore, MD, USA

## Abstract

**Background:**

Synthetic chemicals are increasingly being recognized for potential independent contributions to preterm birth (PTB) and low birth weight (LBW). Bisphenols, parabens, and triclosan are consumer product chemicals that act via similar mechanisms including estrogen, androgen, and thyroid disruption and oxidative stress. Multiple cohort studies have endeavored to examine effects on birth outcomes, and systematic reviews have been limited due to measurement of 1–2 spot samples during pregnancy and limited diversity of populations.

**Objective:**

To study the effects of prenatal phenols and parabens on birth size and gestational age (GA) in 3,619 mother-infant pairs from 11 cohorts in the NIH Environmental influences on Child Health Outcomes program.

**Results:**

While many associations were modest and statistically imprecise, a 1-unit increase in log_10_ pregnancy averaged concentration of benzophenone-3 and methylparaben were associated with decreases in birthweight, birthweight adjusted for gestational age and SGA. Increases in the odds of being SGA were 29% (95% CI: 5%, 58%) and 32% (95% CI: 3%, 70%), respectively. Bisphenol S in third trimester was also associated with SGA (OR 1.52, 95% CI 1.08, 2.13). Associations of benzophenone-3 and methylparaben with PTB and LBW were null. In addition, a 1-unit increase in log_10_ pregnancy averaged concentration of 2,4-dichlorophenol was associated with 43% lower (95% CI: − 67%, − 2%) odds of low birthweight; the direction of effect was the same for the highly correlated 2,5-dichlorophenol, but with a smaller magnitude (−29%, 95% CI: − 53%, 8%).

**Discussion:**

In a large and diverse sample generally representative of the United States, benzophenone-3 and methylparaben were associated with lower birthweight as well as birthweight adjusted for gestational age and higher odds of SGA, while 2,4-dichlorophenol. These associations with smaller size at birth are concerning in light of the known consequences of intrauterine growth restriction for multiple important health outcomes emerging later in life.

## 1. Introduction

Barker et al. described the profound and long-term consequences of impaired fetal growth, particularly low birth weight (BW, LBW), and reductions in gestational length, particularly preterm birth (PTB). (Barker et al., 1992; Barker et al., 2005) These include infant and childhood mortality; (McCormick, 1985) psychological, behavioral, and educational outcomes in young adulthood; (Hack et al., 2002; Hack et al., 1995) and cardiovascular disease and diabetes in later life. (Frankel et al., 1996; Vos et al., 2006) In the United States, low birth weight (LBW) and preterm birth (PTB) occurred among 8.2% and 10.1% of live births in 2020, failing to achieve Healthy People 2020 goals (7.8% and 9.4%, respectively). (Martin et al., 2021).

Synthetic chemicals are increasingly being recognized for potential independent contributions to PTB and LBW. (Elobeid and Allison, 2008) Animal studies indicate that Bisphenol A (BPA), used in aluminum can linings, thermal paper receipts and other consumer products, induces oxidative stress, (Atkinson and Roy, 1995; Hasselberget al., 2004) and is a low-grade synthetic estrogen (potentially contributing sexually dimorphic effects on fetal growth). (Alonso-Magdalena et al., 2006; vom Saal et al., 2012) Recent attention to bisphenol-related health concerns has also led to increasing substitution with synthetic alternatives that have been identified in paper products (Liao et al., 2012b) and human urine (Liao et al., 2012a) such as bisphenol S (BPS). The few studies that have studied replacements such as BPS have identified similar genotoxicity and estrogenicity to BPA, (Audebert et al., 2011; Chen et al., 2002; Kuruto-Niwa et al., 2005; Okuda et al., 2011; Vinas and Watson, ˜ 2013; Yoshihara et al., 2004) as well as embryonal effects, (Qiu et al., 2015) and oxidative stress. (Zhao et al., 2017).

Other phenolic compounds include parabens, esters of 4-hydroxybenzoic acid used as preservatives in cosmetics, and triclosan, an antimicrobial agent used in cleaning materials and other consumer products. Parabens are known to have estrogenic (Golden et al., 2005) and antiandrogenic properties, (Morisseau and Hammock, 2013) and promote adipocyte differentiation in cell culture. (Hu et al., 2013) Triclosan is known to antagonize thyroid hormone function in algae, invertebrates and certain types of fish (Dann and Hontela, 2011) and has been identified as an oxidant stressor in human studies. (Ferguson et al., 2019) Moreover, these pathways interact; inflammation can influence hormonal regulation in pregnancy. (Challis et al., 2009) Inflammation and oxidative stress can induce endothelial activation common in preeclampsia, and oxidative stress can induce placental insufficiency as well as preeclampsia and premature rupture of membranes. (Perucci et al., 2017).

Multiple cohort studies have examined effects of bisphenols, parabens and triclosan on birth outcomes, and their results have been interrogated further in systematic reviews. One such review emphasized multiple limitations in the studies to date, most prominently the measurement of 1–2 spot samples during pregnancy, which introduces exposure imprecision and limits insight into effects that depend on the stage of fetal development. (Zhong et al., 2020) Another systematic review revealed a positive association of BPA exposure with birth weight (BW), (Zhou et al., 2019) while a third systematic review of thirteen cohort studies examining triclosan exposure and birth outcomes suggested inverse associations with birth weight and gestational age (GA)-adjusted birth weight. (Patti et al., 2021) In particular, these findings have not proven readily generalizable to the US, due to the lack of inclusion of Hispanic/Latino populations.

The NIH Environmental influences on Child Health Outcomes (ECHO) Program unites existing pediatric cohorts from across the United States in a common, harmonized, and prospective protocol to identify environmental and preventable origins of LBW, PTB and other effects on child health and development. We leveraged this large, diverse ECHO cohort to study the effects of prenatal phenol exposures on birth size and GA.

## 2. Methods

### 2.1. Overview

The ECHO cohort study leverages data from 69 unique cohorts to improve understanding of the impact of environmental insults on children’s health. Existing data are harmonized to facilitate pooled analyses, and new data are collected using a common, standardized protocol. (LeWinn et al., 2022) Eligibility for the current analysis included: ≥1 urinary phenol measurements during the index pregnancy, data on child’s GA and BW, and singleton delivery. Cohorts needed to have at least 50 participants meeting these criteria to be included in the analysis. In total, we identified 3,619 mother–child dyads from 11 cohorts with information on up to 11 unique urinary phenol metabolites.

### 2.2. Measurement of urinary phenols and parabens

Phenol metabolites in urine were measured at the Centers for Disease Control and Prevention (Silva et al., 2007), Human Health Exposure Analysis Resource labs (Philip set al. 2018; Rocha et al., 2017; Zhang et al., 2022), and California Department of Public Health. (Gavin et al., 2014) In order to be included in the current analyses, we required that a phenol was a) detectable in > 50% of samples, b) that at least 1,000 participants across cohorts had a detectable sample, and c) > 20 participants within a cohort had measurements performed of an individual chemical or class of chemicals. In addition to examining individual phenol metabolites, we summed bisphenols A, F, and S based on known similarities in chemical structure. If a cohort was missing any of the 3 bisphenols, they were excluded from analyses for summed bisphenols. Summed bisphenols, and all individual phenols and parabens were log_10_ transformed before analysis.

Before transformations, we first replaced any values that were below the lower limit of detection (LLOD) with LLOD/sqrt(2). We then adjusted for urinary dilution; this step required use of either creatinine or specific gravity, depending on availability of each from cohorts. We utilized the Boeninger method to standardize phenol and paraben biomarkers by cohort-specific median creatinine or specific gravity value, which has been shown to be valid previously. (Kuiper et al., 2022) Repeated measures within trimester, when available, were averaged and trimester specific measures or averages were later averaged to pregnancy-average values.

### 2.3. Outcomes

Our continuous outcomes of interest were GA at birth (completed weeks), BW (grams), birth length (cm), and BW for GA z-scores; the latter were standardized using child sex at birth and birth parent’s parity. (Aris et al., 2019) We also considered dichotomous outcomes including PTB (birth < 37 weeks vs. ≥ 37 weeks), SGA and LGA (based on the lower and upper 10th percentiles of z-score standardized BW for GA estimated from a US reference population (Aris et al., 2019)), LBW (<2,500 g vs ≥ 2,500 g), and LBW among preterm and term births.

### 2.4. Covariates

We adjusted all models for a priori theorized confounders. These confounders included maternal age at delivery (continuous years), maternal race and ethnicity (non-Hispanic white, non-Hispanic black, Hispanic/Latino, other/unknown), maternal education (High School Degree or GED or less, and Some college and above), parity (0, 1, and ≥ 2), and child sex at birth (male vs female). In models considering BW for GA z-scores as the outcome, we excluded child sex and parity. Racial and ethnic disparities in endocrine disrupting chemicals have been widely described, (Attina et al., 2019, Chan et al., 2021, Goin et al., 2022, Ruiz et al., 2018) with substantially greater effects described for phthalates in non-Hispanic Blacks at least one study. (Trasande et al., 2013) We therefore included race and ethnicity as a proxy for structural racism (Perry et al., 2021) in main models and evaluated effect modification by race and ethnicity.

### 2.5. Statistical analysis

We first assessed univariate associations of phenol and paraben concentrations and outcomes to major covariates. Our main analyses then considered 2,4-dichlorophenol, 2,5-dichlorophenol, benzophenone-3, triclosan, butylparaben, ethylparaben, methylparaben, propylparaben, bisphenol A, bisphenol F, bisphenol S, and summed bisphenols as primary phenols and parabens of interest. All analyses utilized linear or logistic mixed effects models and included cohort as a random effect term to account for baseline differences across cohorts. For continuous outcomes, we treated the outcome as normally distributed, while for dichotomous outcomes we applied a logistic regression framework; because outcomes are all < 10% of the total sample, we interpret odds ratios as risk ratios (RR). All models were adjusted for the covariates listed above. Because phenols and parabens were available at different time points in pregnancy and, for some mothers, at multiple time points during pregnancy, using the previously described linear or logistic mixed effects models but stratified by trimester, we explored trimester-specific effects of phenols and parabens in addition to estimating effects of pregnancy-averaged phenol and paraben metabolites.

In addition to primary analyses, we conducted sensitivity analyses to explore modifying effects of covariates. Specifically, we estimated associations of phenols and parabens on birth outcomes within strata of child biological sex at birth (male vs female), maternal education (high school vs some college or greater), parity (0, 1, ≥2 previous children), and maternal race/ethnicity (non-Hispanic white, non-Hispanic Black, Hispanic/Latino). We considered whether tobacco use during pregnancy (yes/no) additionally confounded the relationship of phenol metabolites to birth outcomes. We also considered the relationship of phenols and parabens with a more granular categorization of GA: preterm (<37.0 weeks), early term (37.0 ≤ x <39.0 weeks), and late term (≥41.0 weeks) vs term (39.0 ≤ x < 41.0 weeks) as the reference group. Finally, we conducted leave-one-out analyses to determine if the main findings were driven by results from a single cohort.

All analyses represent complete cases with all available outcomes, exposures and covariates, without imputation. Tables indicate final N. All statistical analyses were conducted in SAS statistical software (SAS Institute, Cary, NC). Statistical code to reproduce results are maintained by and available from the ECHO Data Analysis Center.

## 3. Results

**Fig. 1.**
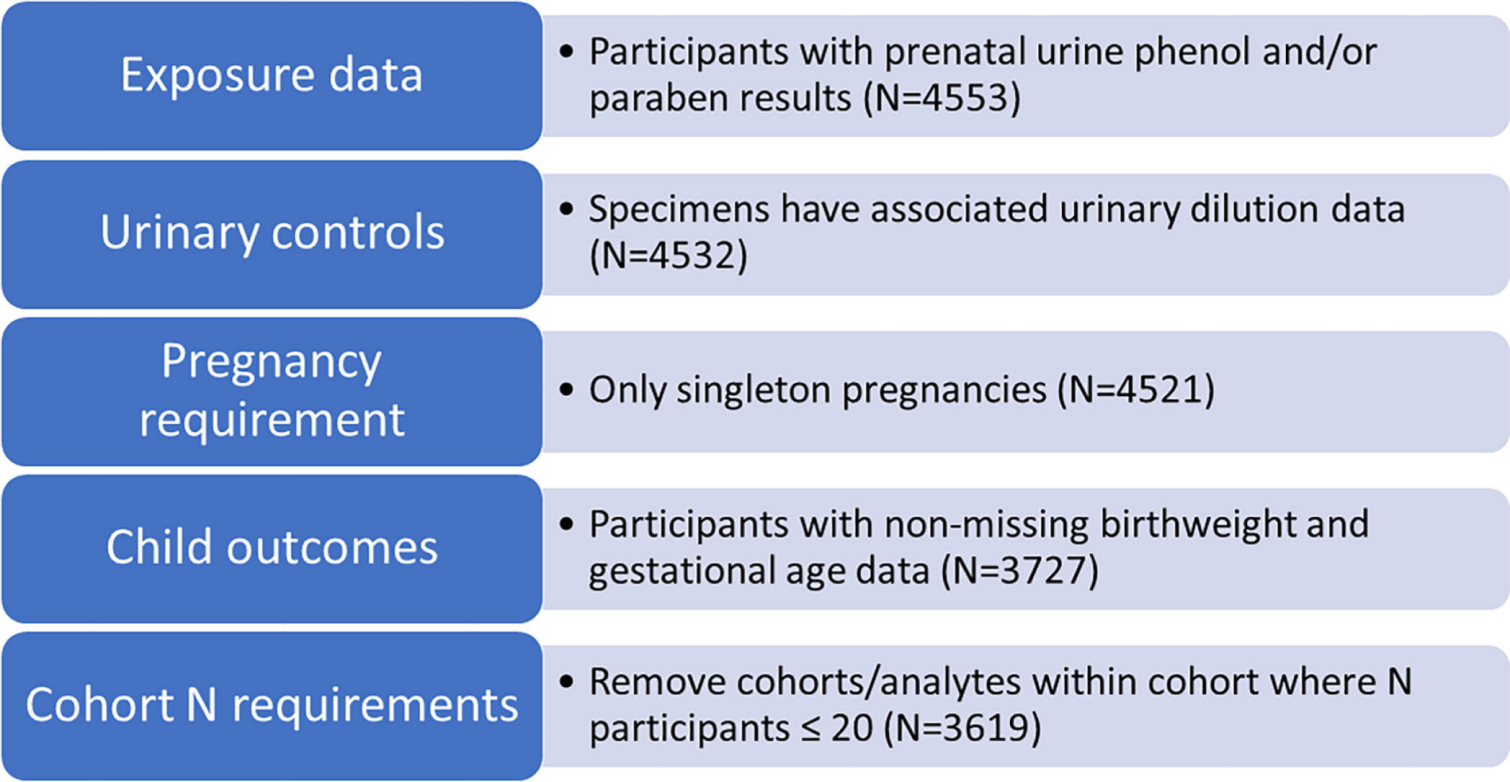
Flow chart of study participants.

Table 1 summarizes characteristics of study participants, and Fig. 1 presents a flow chart of study participants. The majority of mothers were between 25 and 34 years old at the time of participation (58.8%). A notable number of mothers were Hispanic (34.5%), though a large proportion of mothers were white (41.3%); 13.4% of mothers were non-Hispanic Black. Mothers were generally well educated, with most having some college (21.4%), a Bachelor’s degree (25.6%), or a post graduate degree (25%). Finally, many mothers had one child prior to the index child (31.1%).

The highest exposures measured were for benzophenone-3 (median 49.4 ng/mL pregnancy averaged) and methylparaben (median 76.7 ng/mL pregnancy averaged) (Tables 2; Fig. 1). Generally, phenols and parabens were not correlated with one another, with the exceptions of 2,4-dichlorophenol with 2,5-dichlorophenol and methylparaben with propylparaben (Supplemental Figure 1).

Tables 2a and Tables 2b summarize associations of phenol and paraben concentrations to major covariates considered in our work. All exposures demonstrated associations with covariates of interest, with varying magnitude and precision. Notably, Hispanic and non-Hispanic Black mothers had higher concentrations of many phenols and parabens compared to non-Hispanic White mothers. Those with higher education generally experienced lower exposures compared to those with a high school degree. Tables 2c summarizes associations between covariates and birth outcomes of interest. Many covariates were associated with all outcomes of interest and the remaining covariates were associated with at least a subset of outcomes of interest.

Most associations between phenols or parabens with -gestational age, birth length, birth weight, and birthweight for gestational age z-score) were modest and statistically imprecise (Tables 3a). There were two exceptions: benzophenone-3 and methylparaben. A 1-unit increase in log_10_ pregnancy-averaged benzophenone-3 was associated with a 29.2 g decrease in birthweight (95% CI: − 58.00, − 0.40 g), while an identical increase in methylparaben was associated with a 34.0 g decrease in birthweight (95% CI: − 68.90, 0.94 g). Generally, the strongest associations were with third trimester exposures. Third trimester ethylparaben was associated with decreased birthweight (−73.70 g, 95% CI: − 129.00, − 18.70 g). Pregnancy averaged benzophenone-3 and methylparaben were both associated with lower birthweight for gestational age Z-score (−0.08 SD units 95% CI: − 0.15, − 0.02 and − 0.01, 95% CI: − 0.18, − 0.18, respectively), as was third trimester ethylparaben (−0.16 SD units, 95% CI: − 0.28, − 0.03). Bisphenol S was associated with higher birthweight for gestational age Z-score (0.11 SD units, 95% CI 0.00, 0.21).

Associations of phenols and parabens with dichotomous outcomes mirrored those of continuous outcomes (Tables 3b). A 1-unit increase in log_10_ pregnancy averaged concentration of benzophenone-3 and methylparaben were associated with 29% (95% CI: 5%, 58%) and 32% (95% CI: 3%, 70%) increase in the odds of being SGA, respectively. Bisphenol S in third trimester was also associated with SGA (OR 1.52, 95% CI 1.08, 2.13). Associations of benzophenone-3 and methylparaben with PTB and LBW were null. In addition, a 1-unit increase in log_10_ pregnancy averaged concentration of 2,4-dichlorophenol was associated with a 43% decrease (95% CI: − 67%, − 2%) in the odds of low birthweight; the direction of effect was the same for the highly correlated 2,5-dichlorophenol, but the magnitude of the effect was smaller (−29%, 95% CI: − 53%, 8%). Bisphenol F in first trimester was significantly associated with LBW, as was pregnancy wide exposure, as well as bisphenol S and ethylparaben in third trimester.

Sensitivity analyses suggested some variability in effects by a subset of effect modifiers (Supplemental Tables 1**-**4). For example, the relationship of benzophenone-3 with birth length among boys was of a larger magnitude (−0.33 cm, 95% CI: − 0.52, − 0.11 cm) compared to girls (−0.28 cm, 95% CI: − 0.52, − 0.04 cm) (Supplemental Table 1a1). Additionally, the relationship of methylparaben with low birthweight among term births in boys (OR 3.80, 95% CI: 1.37, 10.58) was of a larger magnitude compared to girls (OR 0.88, 95% CI: 0.47, 1.63); Supplemental Table 1B1). The relationship of benzophenone-3 and methylparaben with birth length among non-Hispanic white participants was stronger than in the main analyses with effect estimates of − 0.41 cm (95% CI: − 0.77, − 0.15 cm) and − 0.44 cm (95% CI: − 0.77, − 0.11 cm), respectively (Supplemental Table 2a1) Additionally for this group, the association of methylparaben to birthweight was stronger than in the main analyses (−82.55 g, 95% CI: − 131.65, –33.44 g; Supplemental Table 2a1). The association of benzophenone-3 with birth length among non-Hispanic Black participants was stronger than in the main analyses (−0.77 cm, 95% CI: − 1.22, − 0.10 cm; Supplemental Table 2a2). The association of methylparaben to gestational age among Hispanic participants was notably stronger than in the main analyses (0.23 weeks, 95% CI: 0.03, 0.42 weeks; Supplemental Table 2a3). Methylparaben was more strongly associated with birth weight for gestational age z-scores in mothers with some college education (−0.20 SD units, 95% CI: − 0.33, − 0.11 SD units; Supplemental Table 3a2). No sensitivity analyses supported differences in directions of association by effect measure modifiers of interest (Supplemental Table 5). Finally, leave one out analyses showed that the effects estimated for the pooled group of cohorts were not notably influenced by any specific cohorts (Supplemental Figure 2).

## 4. Discussion

In a large and diverse sample generally representative of the United States, benzophenone-3 and methylparaben concentrations in maternal urine during pregnancy were associated with decreases in birthweight as well as birthweight adjusted for gestational age and small for gestational age. These are concerning findings in light of the known consequences of intrauterine growth restriction for multiple later and important health outcomes emerging later in youth and even through adulthood. (Barker et al., 1992; Barker et al., 2005; Frankel et al., 1996; Hack et al., 2002; Hack et al., 1995; McCormick, 1985; Vos et al., 2006).

The results do largely align with those of previous *meta*-analyses of environmental phenol exposures with birth outcomes. (Zhong et al., 2020) While we do not identify a positive association of BPA exposure with birth weight, we note lower BPA levels than the previous systematic review, (Zhou et al., 2019) in part due to the emergence of replacement bisphenols. When we examined bisphenol S, we observed increases in birth weight supported by the similar toxicological profile to BPA identified in previous studies. (Audebert et al., 2011; Chen et al., 2002; Kuruto-Niwa et al., 2005; Okuda et al., 2011; Qiu et al., 2015; Vinas and Watson, 2013˜ ; Yoshihara et al., 2004; Zhao et al., 2017) Notably, concentrations of BPA replacements (BPF, BPS) are generally lower than those of BPA in this sample. We do find inverse associations of triclosan with birth weight and gestational age-adjusted birth weight, as suggested in the previous *meta*-analysis, but these are also nonsignificant. (Patti et al., 2021) The very large sample size allowed us to stratify by the trimester of measurement improving exposure precision and insight into effects that depend on the stage of fetal development. (Zhong et al., 2020).

We acknowledge that our study does not interrogate the many mechanisms by which bisphenols can impair fetal growth. BPA induces oxidative stress, (Atkinson and Roy, 1995; Hasselberg et al., 2004) is directly cardiotoxic, (Khodayar et al., 2020; Quagliariello et al., 2019) and reduces the function of adiponectin, a cardioprotective adipokine. (Hugo et al., 2008) BPA is also a low-grade synthetic estrogen, (Alonso-Magdalena et al., 2006; vom Saal et al., 2012) disrupts pancreatic β-cell function *in vivo*, (Alonso-Magdalena et al., 2005) and affects glucose transport in adipocytes. (Hugo et al., 2008; Masuno et al., 2002; Sakurai et al., 2004) The few studies that have studied replacements such as BPS have identified similar genotoxicity and estrogenicity to BPA, (Audebert et al., 2011; Chen et al., 2002; Kuruto-Niwa et al., 2005; Okuda et al., 2011; Vinas and Watson, 2013˜ ; Yoshihara et al., 2004) embryonal effects, (Qiu et al., 2015) oxidative stress, (Zhao et al., 2017) cardiotoxicity, (Gu et al., 2020) disruption of osteoblast function, (Chin et al., 2018) and greater resistance to environmental degradation. (Danzl et al., 2009; Ike et al., 2006) Parabens are known estrogens (Golden et al., 2005) and antiandrogens, (Morisseau and Hammock, 2013) and promote adipocyte differentiation. (Hu et al., 2013) Triclosan is known to antagonize thyroid hormone function (Dann and Hontela, 2011) and is an oxidant stressor. (Ferguson et al., 2019) We were unable to examine these many interacting mechanisms, (Challis et al., 2009) which may explain some heterogeneity and even modesty in the statistically significant associations.

The sensitivity analyses reveal suggestive, although not always consistent, associations that vary, particularly by race/ethnicity, supportive of the need to evaluate potential modification by factors associated with structural racism. We were not able to access specific data on racism in the study population to evaluate this further. Later work should leverage ongoing data collection using validated instruments. There were also differences by sex, which are important given the sex steroid pathway disruption known to be induced by phenols and parabens. The leave one out analyses also support the rigor of the results obtained.

Strengths of the analysis include the large sample size, high quality of laboratory analyses, harmonization approach, multiple robustness checks, and the specificity of the effect to birth weight. The ECHO consortium combines data from many cohorts representing diverse populations and exposures over time, which allowed for evaluation of the impact of replacement phenols and parabens on birth outcomes. Most phenols and parabens were highly detected, which minimized the need for imputing values below the analytic limit of detection.

There are limitations to interpretation, which include the potential for unmeasured confounding. The pattern of results for benzophenone-3 is not completely inconsistent. Benzophenone-3 was significantly associated with lower birthweight, lower birthweight-for-gestational-age z-score, and higher odds of SGA, but not with higher odds of LBW or even LBW stratified by term and preterm. Regarding the nonsignificant associations we do note that even in ECHO stratified analyses have limited power. We also note that decreases in BW can be differential across the spectrum of BW, perhaps shifting in the normal range more than from normal to LBW.

As phenols and parabens are known dietary contaminants, the lack of harmonized diet data across the cohorts is important to emphasize. Although each individual cohort by itself has limited power to observe small effects when considered alone, associations were consistently in the same direction. Another limitation of the trimester-specific analyses is that different cohorts/participants contribute to each analysis, making the results difficult to directly compare across trimesters. We emphasize that other risks such as smoking are comparably small in this multifactorial condition, and also are not as readily modifiable. Pharmacokinetic studies in adults suggest that bisphenols have 12–48 h half-lives, (Mahalingaiah et al., 2008; Stahlhut et al., 2009) raising the potential for exposure imprecision introduced by relying on spot urine samples. We do note that weak indices of exposure could bias associations toward the null, (Carroll, 1998; Fleiss and Shrout, 1977; Fuller, 1987) though this post-hoc justification has limits. We also acknowledge potential residual confounding by unmeasured or unknown co-exposures.

Further studies are needed to interrogate the longer-term consequences of the observed decreases in birth weight. Additional measurements of metabolomic, epigenetic, oxidant stress, thyroid hormone function and other multiomic data would allow us to disaggregate the multiple mechanisms which may explain the complex pattern of associations identified in this manuscript.

## 5. Conclusion

In a large, diverse American sample, benzophenone-3 and methylparaben exposures were associated with decreases in BW and increases in SGA, suggesting opportunities for prevention. We also identify suggestive increases in birth weight due to bisphenol S, an emerging replacement of BPA. The findings here support further examination of later-life consequences of phenol exposure in pregnancy, as well as intermediate mechanisms that may explain the complex pattern of findings.

## Supplementary Material

1

## Figures and Tables

**Table 1:** Descriptive statistics of Demographics, Exposures, and Outcomes.

Demographic Descriptives Maternal variables		
**Age category (N [%])**		
< 25		828 [22.9 %]
25–34		2128 [58.8 %]
35+		663 [18.3 %]
**Race/Ethnicity (N [%])**		
Non-Hispanic White		1493 [41.3 %]
Non-Hispanic Black		485 [13.4 %]
Hispanic		1250 [34.5 %]
Other/Unknown		391 [10.8 %]
**Education (N [%])**		
Less than high school		396 [10.9 %]
High school degree, GED or equivalent		563 [15.6 %]
Some college, no degree; Associate’s degree;Trade school		776 [21.4 %]
Bachelor’s degree		925 [25.6 %]
Post graduate degree		904 [25.0 %]
*Missing*		*55 [1.5 %]*
**Parity (N [%])**		
0		1377 [38.0 %]
1		1125 [31.1 %]
2		349 [9.6 %]
3		114 [3.2 %]
4+		66 [1.8 %]
*Missing*		*588 [16.2 %]*
**Tobacco Use during Pregnancy (N [%])**		
Yes		129 [3.6 %]
No		2759 [76.2 %]
*Missing*		*731 [20.2 %]*

Child variables		
**Child Sex (N [%])**		
Male		1828 [50.5 %]
Female		1789 [49.4 %]
*Missing*		*2 [0.1 %]*.

Exposure Distributions		

**Sum Bisphenols**	**N**	**ng/mL Median [IQR]**
Across Pregnancy	2258	2.6 [2.5]
Trimester 1	1164	2.0 [2.6]
Trimester 2	2073	2.2 [2.8]
Trimester 3	1196	2.2 [3.1]
**Bisphenol A**	**N**	**ng/mL Median [IQR]**
Across Pregnancy	3406	1.1 [1.3]
Trimester 1	1391	0.8 [1.3]
Trimester 2	2555	0.9 [1.4]
Trimester 3	1885	1.1 [1.7]
**Bisphenol F**	**N**	**ng/mL Median [IQR]**
Across Pregnancy	2342	0.5 [1.0]
Trimester 1	1236	0.5 [0.8]
Trimester 2	2099	0.4 [0.7]
Trimester 3	1203	0.4 [0.7]
**Bisphenol S**	**N**	**ng/mL Median [IQR]**
Across Pregnancy	2749	0.4 [0.6]
Trimester 1	1202	0.3 [0.6]
Trimester 2	2269	0.4 [0.6]
Trimester 3	1469	0.3 [0.6]
**2,4-Dichlorophenol**	**N**	**ng/mL Median [IQR]**
Across Pregnancy	1689	0.8 [1.7]
Trimester 1	104	0.2 [0.3]
Trimester 2	944	0.8 [1.0]
Trimester 3	812	1.4 [3.0]
**2,5-Dichlorophenol**	**N**	**ng/mL Median [IQR]**
Across Pregnancy	1686	4.1 [34.2]
Trimester 1	101	0.4 [1.0]
Trimester 2	946	2.4 [7.8]
Trimester 3	811	22 [94]
**Benzophenone-3**	**N**	**ng/mL Median [IQR]**
Across Pregnancy	2091	49.4 [195.3]
Trimester 1	318	50.9 [210.4]
Trimester 2	1335	62.2 [232.6]
Trimester 3	837	24.0 [103.3]
**Triclosan**	**N**	**ng/mL Median [IQR]**
Across Pregnancy	2200	10.6 [53.6]
Trimester 1	379	7.2 [38]
Trimester 2	1351	10.5 [62.9]
Trimester 3	850	9.4 [53.2]
**Butyl paraben**	**N**	**ng/mL Median [IQR]**
Across Pregnancy	1816	0.2 [0.9]
Trimester 1	153	0.6 [2.0]
Trimester 2	1014	0.2 [0.8]
Trimester 3	831	0.2 [0.9]
**Ethyl paraben**	**N**	**ng/mL Median [IQR]**
Across Pregnancy	1562	1.7 [6.5]
Trimester 1	228	1.3 [5.0]
Trimester 2	1031	1.5 [6.4]
Trimester 3	494	1.4 [4.4]
**Methyl paraben**	**N**	**ng/mL Median [IQR]**
Across Pregnancy	1938	76.7 [203.8]
Trimester 1	234	70.5 [196.5]
Trimester 2	1038	55.2 [163.5]
Trimester 3	863	98 [244.4]
**Propyl paraben**	**N**	**ng/mL Median [IQR]**
Across Pregnancy	1925	15.3 [54.2]
Trimester 1	230	16.2 [75.8]
Trimester 2	1032	9.5 [44.3]
Trimester 3	859	17.7 [61.5]

Outcome Distributions		

Birth GA (weeks Mean [STD])	N=3619	38.9 [1.7]
Preterm (N [%])	N=3619	273 [7.5]
Birth Length (cm Mean [STD])	N=3118	50.5 [2.9]
Birth Weight (gm Mean [STD])	N=3619	3322.6 [523.2]
Small for GA (N [%])	N=3609	427 [11.8]
Large for GA (N [%])	N=3609	368 [10.2]
Birthweight for GA (z-score Mean [STD])	N=3022	0 [1.08]
Low Birthweight (N [%])	N=3619	199 [5.5]
LBW (Preterm) (N [%])	N=273	108 [39.6]
LBW (Term) (N [%])	N=3346	91 [2.7]

**Table 2a:** Bivariate Associations of Phenol Exposures with Covariates.

	Sum Bisphenols	Bisphenol A	Bisphenol F	Bisphenol S	2,4-Dichlorophenol	2,5-Dichlorophenol	Benzophenone-3	Triclosan
Covariate	Beta (95 % CI)	Beta (95 % CI)	Beta (95 % CI)	Beta (95 % CI)	Beta (95 % CI)	Beta (95 % CI)	Beta (95 % CI)	Beta (95 % CI)

Maternal Age	0.00 (−0.00, 0.00)	**−0.001 (−0.01, −0.01)**	**0.01 (0.00, 0.01)**	0.00 (−0.01, 0.00)	**−0.02 (−0.02, −0.02)**	**−0.06 (−0.06, −0.05)**	**0.04 (0.03, 0.04)**	0.01 (0.01, 0.02)
Race: B vs W	− 0.02 (−0.08, 0.04)	**0.17 (0.12, 0.21)**	− 0.06 (−0.15, 0.02)	0.02 (−0.05, 0.09)	**0.53 (0.45, 0.61)**	**1.39 (1.27, 1.51)**	**−0.86 (−0.10, −0.75)**	**−0.16 (−0.28, −0.05)**
Race: His vs W	− 0.01 (−0.04, 0.02)	**0.12 (0.09, 0.15)**	**−0.24 (−0.29, −0.19)**	**0.15 (0.11, 0.19)**	**0.48 (0.43, 0.54)**	**1.24 (1.16, 1.32)**	**−0.48 (−0.55, −0.40)**	− 0.01 (−0.08, 0.07)
Race: Other vs W	− 0.04 (−0.09, 0.01)	0.03 (−0.02, 0.08)	**−0.16 (−0.23, −0.09)**	**0.09 (0.04, 0.15)**	**0.24 (0.12, 0.35)**	**0.48 (0.31, 0.65)**	**−0.24 (−0.35, −0.12)**	0.08 (−0.04, 0.20)
Ed: Higher Ed vs HS	0.03 (−0.01, 0.06)	**−0.08 (−0.11, −0.05)**	**0.09 (0.03, 0.14)**	**−0.07 (−0.11, −0.03)**	**−0.37 (−0.43, −0.31)**	**−0.88 (−0.10, −0.78)**	**0.58 (0.50, 0.66)**	0.17 (0.09, 0.25)
Parity: 1 vs 0	− 0.03 (−0.06, 0.01)	**−0.06 (−0.10, −0.03)**	**−0.06 (−0.12, −0.01)**	0.03 (−0.01, 0.07)	0.01 (−0.06, 0.07)	0.05 (−0.06, 0.16)	0.01 (−0.07, 0.09)	− 0.02 (−0.01, 0.06)
Parity: 2 + vs 0	0.00 (−0.05, 0.06)	0.04 (−0.01, 0.08)	0.01 (−0.07, 0.09)	**−0.06 (−0.12, −0.01)**	− 0.01 (−0.09, 0.06)	0.03 (−0.10, 0.15)	**−0.16 (−0.25, −0.06)**	− 0.08 (−0.18, 0.01)
Tobacco use vs none	0.06 (−0.06, 0.17)	0.07 (−0.01, 0.15)	**0.16 (0.01, 0.32)**	0.04 (−0.07, 0.14)	− 0.03 (−0.16, 0.11)	− 0.01 (−0.24, 0.23)	**−0.23 (−0.42, −0.05)**	− 0.00 (−0.19, 0.18)
Child Sex: F vs M	0.00 (−0.03, 0.03)	− 0.02 (−0.04, 0.01)	0.00 (−0.04, 0.05)	0.01 (−0.02, 0.05)	0.05 (−0.01, 0.10)	**0.11 (0.02, 0.21)**	− 0.05 (−0.12, 0.02)	− 0.00 (−0.07, 0.07)

(CI = Confidence Interval, B = Non-Hispanic Black, W = Non-Hispanic White, His = Hispanic, Ed = Education, F = Female, M = Male).

**Bold** indicates significance at p < 0.05.

**Table 2b:** Bivariate Associations of Paraben Exposures with Covariates.

	Butyl paraben	Ethyl paraben	Methyl paraben	Propyl paraben
Covariate	Beta (95 % CI)	Beta (95 % CI)	Beta (95 % CI)	Beta (95 % CI)

Maternal Age	0.00 (−0.00, 0.001)	**0.01 (0.00, 0.02)**	**−0.01 (−0.01, 0.00)**	− 0.00 (−0.01, 0.00)
Race: B vs W	0.04 (−0.07, 0.15)	**0.14 (0.00, 0.28)**	**0.43 (0.34, 0.52)**	**0.43 (0.32, 0.54)**
Race: His vs W	**0.24 (0.16, 0.32)**	**0.10 (0.01, 0.18)**	**0.18 (0.12, 0.25)**	**0.12 (0.04, 0.20)**
Race: Other vs W	**0.21 (0.05, 0.37)**	0.15 (0.00, 0.31)	**0.28 (0.15, 0.41)**	**0.22 (0.06, 0.38)**
Ed: Higher Ed vs HS	**−0.11 (−0.19, −0.03)**	**0.16 (0.06, 0.25)**	**−0.11 (−0.18, −0.05)**	**−0.11 (−0.19, −0.03)**
Parity: 1 vs 0	0.03 (−0.05, 0.11)	0.02 (−0.07, 0.11)	− 0.01 (−0.08, 0.06)	0.04 (−0.05, 0.12)
Parity: 2 + vs 0	0.03 (−0.07, 0.12)	**−0.10 (−0.21, 0.00)**	− 0.03 (−0.11, 0.05)	0.00 (−0.10, 0.10)
Tobacco use vs none	− 0.08 (−0.25, 0.09)	0.03 (−0.15, 0.21)	0.03 (−0.11, 0.18)	0.11 (−0.07, 0.28)
Child Sex: F vs M	0.00 (−0.07, 0.08)	− 0.07 (−0.14, 0.01)	− 0.04 (−0.09, 0.02)	− 0.05 (−0.13, 0.02)

(CI = Confidence Interval, B = Non-Hispanic Black, W = Non-Hispanic White, His = Hispanic, Ed = Education, F = Female, M = Male).

**Bold** indicates significance at p < 0.05.

**Table 2c:** Bivariate Associations of Outcomes with Covariates.

	Gestational Age	Preterm	Birth Length	Birth Weight	Small for GA	Large for GA	BW for GA	Low Birth Weight
Covariate	Beta (95 % CI)	OR (95 % CI)	Beta (95 % CI)	Beta (95 % CI)	OR (95 % CI)	OR (95 % CI)	Beta (95 % CI)	OR (95 % CI)

Maternal Age	− 0.00 (−0.01, 0.01)	1 (0.98, 1.02)	**0.04 (0.02, 0.05)**	**7.18 (4.27, 10.01)**	**0.98 (0.96, 1.00)**	**1.03 (1.01, 1.05)**	**0.01 (0.01, 0.018)**	1.02 (0.99, 1.04)
Race: B vs W	**−0.54 (−0.71, −0.36)**	**2 (1.42, 2.82)**	**−1.08 (−1.41, −0.74)**	**−263.00 (−315.82, −210.15)**	**2.07 (1.55, 2.75)**	**0.60 (0.41, 0.86)**	**−0.40 (−0.52, −0.28)**	**2.58 (1.74, 3.82)**
Race: His vs W	**−0.28 (−0.41, −0.15)**	1.18 (0.88, 1.59)	**−0.54 (−0.76, −0.31)**	**−116.06 (−154.81, −77.30)**	1.16 (0.91, 1.48)	**0.74 (0.58, 0.94)**	**−0.18 (−0.26, −0.09)**	1.31 (0.92, 1.87)
Race: Other vs W	− 0.14 (−0.33, 0.06)	1.09 (0.70, 1.70)	− 0.30 (−0.64, 0.03)	**−111.48 (−168.91, −54.05)**	**1.48 (1.06, 2.07)**	**0.64 (0.43, 0.95)**	**−0.26 (−0.40, −0.13)**	1.54 (0.94, 2.5)
Ed: Higher Ed vs HS	0.03 (−0.10, 0.16)	0.89 (0.67, 1.17)	**0.52 (0.29, 0.75)**	**61.45 (22.91, 100.00)**	**0.78 (0.63, 0.98)**	1.14 (0.89, 1.47)	**0.14 (0.05, 0.22)**	0.87 (0.63, 1.20)
Parity: 1 vs 0	**−0.26 (−0.39, −0.12)**	1.11 (0.81, 1.51)	0.07 (−0.16, 0.31)	**81.80 (41.87, 121.74)**	**0.64 (0.49, 0.82)**	**1.44 (1.11, 1.89)**	0.06 (−0.02, 0.15)	0.81 (0.57, 1.15)
Parity: 2 + vs 0	**−0.20 (−0.37, −0.03)**	1.3 (0.89, 1.89)	0.19 (−0.12, 0.50)	**99.61 (48.78, 150.43)**	**0.42 (0.29, 0.62)**	**1.47 (1.06, 2.05)**	0.03 (−0.08, 0.14)	**0.60 (0.36, 0.99)**
Tobacco use vs none	− 0.078 (−0.38, 0.23)	0.93 (0.47, 1.86)	**−0.707 (−1.27, −0.14)**	**−152.08 (−242.57, −61.60)**	**1.97 (1.25, 3.1)**	0.58 (0.28, 1.19)	**−0.41 (−0.61, −0.22)**	1.53 (0.78, 2.97)
Child Sex: F vs M	0.06 (−0.05, 0.17)	0.87 (0.68, 1.11)	**−0.91 (−1.10, −0.71)**	**−139.56 (−173.36, −105.76)**	1.21 (0.99, 1.48)	1.01 (0.82, 1.26)	− 0.04 (−0.12, 0.04)	**1.46 (1.09, 1.95)**

(CI = Confidence Interval, B = Non-Hispanic Black, W = Non-Hispanic White, His = Hispanic, Ed = Education, F = Female, M = Male).

**Bold** indicates significance at p < 0.05.

**Table 3a:** Adjusted associations of phenol exposures with birth outcomes (continuously measured).

Outcome: Gestational Age								
	Across Pregnancy		Trimester 1		Trimester 2		Trimester 3	
				
Phenol Name	N	Beta (95 % CI)	N	Beta (95 % CI)	N	Beta (95 % CI)	N	Beta (95 % CI)

Sum Bisphenols	1667	0.04 (−0.19, 0.28)	701	− 0.13 (−0.44, 0.19)	1557	− 0.06 (−0.27, 0.16)	700	0.09 (−0.18, 0.35)
Bisphenol A	2807	0.07 (−0.08, 0.23)	927	0.03 (−0.21, 0.27)	2031	− 0.01 (−0.16, 0.15)	1385	0.02 (−0.15, 0.19)
Bisphenol F	1724	− 0.02 (−0.18, 0.13)	743	− 0.21 (−0.48, 0.05)	1576	0.10 (−0.07, 0.26)	705	0.02 (−0.19, 0.23)
Bisphenol S	2130	− 0.07 (−0.24, 0.09)	714	− 0.16 (−0.38, 0.07)	1742	− 0.07 (−0.23, 0.10)	969	− 0.03 (−0.19, 0.14)
2,4-Dichlorophenol	1573	0.07 (−0.09, 0.23)	48	0.11 (−0.88, 1.11)	877	0.07 (−0.16, 0.29)	784	0.03 (−0.17, 0.24)
2,5-Dichlorophenol	1572	0.08 (−0.03, 0.20)	47	0.27 (−0.66, 1.19)	879	0.04 (−0.11, 0.19)	783	0.06 (−0.10, 0.21)
Benzophenone-3	1965	0.01 (−0.09, 0.10)	259	0.22 (−0.10, 0.53)	1259	0.04 (−0.08, 0.15)	806	0.01 (−0.13, 0.16)
Triclosan	2096	− 0.03 (−0.11, 0.06)	338	− 0.04 (−0.27, 0.19)	1289	− 0.06 (−0.16, 0.04)	822	− 0.03 (−0.16, 0.10)
Butyl paraben	1723	0.09 (−0.01, 0.19)	126	0.05 (−0.25, 0.36)	945	**0.13 (0.01, 0.25)**	803	− 0.01 (−0.15, 0.14)
Ethyl paraben	1439	− 0.02 (−0.12, 0.08)	171	0.05 (−0.23, 0.33)	957	− 0.02 (−0.14, 0.11)	465	− 0.04 (−0.20, 0.12)
Methyl Paraben	1811	0.00 (−0.11, 0.12)	173	0.20 (−0.10, 0.49)	962	− 0.01 (−0.15, 0.13)	832	− 0.08 (−0.24, 0.09)
Propyl paraben	1799	0.04 (−0.05, 0.13)	170	**0.25 (0.01, 0.50)**	957	− 0.03 (−0.14, 0.08)	828	0.01 (−0.12, 0.19)

Outcome: Birth Length								
	Across Pregnancy		Trimester 1		Trimester 2		Trimester 3	
				
Phenol Name	N	Beta (95 % CI)	N	Beta (95 % CI)	N	Beta (95 % CI)	N	Beta (95 % CI)

Sum Bisphenols	1610	− 0.001 (−0.39, 0.38)	672	− 0.13 (−0.61, 0.345)	1504	− 0.10 (−0.46, 0.26)	667	0.03 (−0.40, 0.47)
Bisphenol A	2630	− 0.01 (−0.27, 0.24)	822	− 0.01 (−0.37, 0.35)	1914	− 0.07 (−0.33, 0.19)	1296	0.10 (−0.19, 0.39)
Bisphenol F	1662	− 0.18 (−0.43, 0.08)	713	**−0.50 (−0.90, −0.10)**	1522	− 0.04 (−0.32, 0.24)	669	0.13 (−0.22, 0.48)
Bisphenol S	2068	0.02 (−0.25, 0.30)	685	− 0.19 (−0.53, 0.15)	1687	0.14 (−0.14, 0.41)	933	− 0.21 (−0.49, 0.08)
2,4-Dichlorophenol	1525	0.02 (−0.26, 0.30)	48	**2.02 (0.26, 3.78)**	858	0.13 (−0.27, 0.53)	753	− 0.03 (−0.39, 0.34)
2,5-Dichlorophenol	1524	0.06 (−0.14, 0.26)	47	0.33 (−1.40, 2.06)	860	0.12 (−0.14, 0.38)	752	0.03 (−0.25, 0.31)
Benzophenone-3	1914	− 0.14 (−0.31, 0.03)	259	0.12 (−0.36, 0.60)	1237	− 0.08 (−0.28, 0.12)	775	− 0.12 (−0.38, 0.13)
Triclosan	1876	− 0.02 (−0.17, 0.13)	240	− 0.13 (−0.52, 0.27)	1219	− 0.12 (−0.29, 0.05)	768	0.18 (−0.05, 0.41)
Butyl paraben	1557	0.04 (−0.14, 0.22)	55	− 0.29 (−1.00, 0.42)	891	0.06 (−0.16, 0.28)	760	− 0.02 (−0.28, 0.25)
Ethyl paraben	1243	0.16 (−0.07, 0.36)	72	0.24 (−0.74, 1.21)	889	**0.27 (0.03, 0.50)**	434	− 0.09 (−0.43, 0.24)
Methyl Paraben	1589	− 0.02 (−0.24, 0.20)	74	− 0.12 (−1.03, 0.78)	894	0.05 (−0.21, 0.30)	775	− 0.14 (−0.44, 0.17)
Propyl paraben	1588	− 0.03 (−0.20, 0.14)	73	0.33 (−0.45, 1.10)	894	0.02 (−0.18, 0.22)	775	− 0.09 (−0.33, 0.15)

Outcome: Birth Weight								
	Across Pregnancy		Trimester 1		Trimester 2		Trimester 3	
				
Phenol Name	N	Beta (95 % CI)	N	Beta (95 % CI)	N	Beta (95 % CI)	N	Beta (95 % CI)

Sum Bisphenols	1667	7.73 (−62.53, 78.00)	701	13.06 (−76.3, 102.42)	1557	11.799 (−53.16, 76.76)	700	− 1.93 (−86.96, 83.09)
Bisphenol A	2807	11.04 (−34.42, 56.50)	927	43.26 (–22.61, 109.14)	2031	3.644 (−42.78, 50.07)	1385	1.30 (−50.08, 52.68)
Bisphenol F	1724	–22.82 (−69.32, 23.68)	743	− 61.89 (−135.54, 11.76)	1576	14.991 (−35.23, 65.22)	705	2.08 (−66.75, 70.91)
Bisphenol S	2130	− 0.90 (−49.34, 47.55)	714	− 17.11 (−81.17, 46.96)	1742	32.376 (−16.85, 81.60)	969	− 44.59 (−97.21, 8.03)
2,4-Dichlorophenol	1573	30.80 (−17.72, 79.33)	48	90.32 (−214.07, 394.70)	877	68.872 (−4.54, 142.29)	784	20.89 (−41.57, 83.351)
2,5-Dichlorophenol	1572	11.88 (–22.69, 46.45)	47	111.32 (−168.03, 390.68)	879	4.628 (−43.13, 52.39)	783	15.20 (−31.95, 62.34)
Benzophenone-3	1965	**−29.21 (−58.03, −0.40)**	259	− 10.65 (−98.74, 77.44)	1259	− 16.492 (−52.07, 19.09)	806	− 24.37 (−67.28, 18.55)
Triclosan	2096	− 8.18 (–33.57, 17.21)	338	− 43.19 (−109.29, 22.91)	1289	− 7.764 (−39.00, 23.47)	822	4.034 (−34.67, 42.74)
Butyl paraben	1723	3.13 (−26.68, 32.93)	126	− 13.21 (−116.27, 89.79)	945	16.321 (–22.45, 55.09)	803	− 17.80 (−61.34, 25.74)
Ethyl paraben	1439	− 17.51 (−50.34, 15.38)	171	− 6.28 (−97.82, 85.26)	957	3.765 (−37.78, 45.31)	465	**−73.74 (−128.80, −18.678)**
Methyl Paraben	1811	− 34.00 (−68.93, 0.94)	173	32.41 (−65.72, 130.55)	962	− 31.995 (−76.95, 12.96)	832	− 49.04 (−98.95, 0.87)
Propyl paraben	1799	− 4.36 (–32.15, 23.43)	170	57.20 (–23.30, 137.71)	957	− 18.066 (−54.26, 18.12)	828	− 5.52 (−45.14, 34.10)

Outcome: Birthweight for Gestational Age Z-Score								
	Across Pregnancy		Trimester 1		Trimester 2		Trimester 3	
				
Phenol Name	N	Beta (95 % CI)	N	Beta (95 % CI)	N	Beta (95 % CI)	N	Beta (95 % CI)

Sum Bisphenols	1667	0.01 (−0.14, 0.16)	701	0.07 (−0.12, 0.25)	1557	0.06 (−0.079, 0.194)	700	− 0.01 (−0.19, 0.17)
Bisphenol A	2800	− 0.01 (−0.11, 0.09)	926	0.08 (−0.06, 0.21)	2030	0.01 (−0.089, 0.106)	1380	− 0.01 (−0.13, 0.11)
Bisphenol F	1724	− 0.04 (−0.14, 0.06)	743	− 0.08 (−0.23, 0.07)	1576	− 0.01 (−0.11, 0.101)	705	0.00 (−0.14, 0.15)
Bisphenol S	2130	0.01 (−0.10, 0.11)	714	0.02 (−0.11, 0.15)	1742	**0.11 (0.004, 0.212)**	969	− 0.10 (−0.22, 0.01)
2,4-Dichlorophenol	1567	0.05 (−0.07, 0.16)	48	0.26 (−0.39, 0.91)	877	0.14 (−0.023, 0.307)	779	0.07 (−0.08, 0.21)
2,5-Dichlorophenol	1566	− 0.00 (−0.08, 0.08)	47	0.03 (−0.55, 0.60)	879	0.01 (−0.096, 0.12)	778	0.04 (−0.07, 0.15)
Benzophenone-3	1959	**−0.08 (−0.15, −0.02)**	259	− 0.13 (−0.30, 0.04)	1259	− 0.06 (−0.132, 0.023)	801	− 0.07 (−0.17, 0.04)
Triclosan	2090	− 0.01 (−0.06, 0.05)	338	− 0.10 (−0.23, 0.02)	1289	0.01 (−0.06, 0.076)	817	0.03 (−0.07, 0.12)
Butyl paraben	1717	− 0.04 (−0.11, 0.03)	126	− 0.08 (−0.32, 0.15)	945	− 0.02 (−0.109, 0.065)	798	− 0.04 (−0.14, 0.07)
Ethyl paraben	1439	− 0.03 (−0.11, 0.04)	171	− 0.04 (−0.240, 0.16)	957	0.03 (−0.061, 0.124)	465	**−0.16 (−0.28, −0.03)**
Methyl Paraben	1805	**−0.10 (−0.18, −0.02)**	173	− 0.02 (−0.24, 0.19)	962	− 0.06 (−0.162, 0.04)	827	− 0.10 (−0.21, 0.02)
Propyl paraben	1793	− 0.03 (−0.09, 0.03)	170	0.02 (−0.16, 0.19)	957	− 0.03 (−0.106, 0.055)	823	− 0.02 (−0.11, 0.07)

Models adjusted for maternal race/ethnicity, parity, and education, and child sex; cohort as random effect. BW for GA model removes parity and child sex from covariates.(CI = Confidence Interval).

Bold indicates significance at p < 0.05.

**Table 3b:** Adjusted Associations of Phenol Exposures with Categorical Birth Outcomes.

Outcome: Preterm								
	Across Pregnancy		Trimester 1		Trimester 2		Trimester 3	
				
Phenol Name	N	Beta (95 % CI)	N	Beta (95 % CI)	N	Beta (95 % CI)	N	Beta (95 % CI)

Sum Bisphenols	1667	1.24 (0.72, 2.12)	701	0.94 (0.43, 2.07)	1557	1.26 (0.77, 2.07)	700	0.86 (0.40, 1.88)
Bisphenol A	2807	0.89 (0.61, 1.29)	927	0.69 (0.40, 1.17)	2031	1.09 (0.76, 1.57)	1385	0.97 (0.61, 1.55)
Bisphenol F	1724	1.36 (0.98, 1.88)	743	1.41 (0.83, 2.41)	1576	1.06 (0.72, 1.55)	705	1.07 (0.58, 1.96)
Bisphenol S	2130	1.01 (0.66, 1.53)	714	1.06 (0.62, 1.83)	1742	1.08 (0.72, 1.61)	969	0.90 (0.53, 1.52)
2,4-Dichlorophenol	1573	0.81 (0.51, 1.27)			877	0.74 (0.38, 1.45)	784	1.17 (0.70, 1.95)
2,5-Dichlorophenol	1572	0.96 (0.70, 1.32)			879	1.05 (0.69, 1.62)	783	1.19 (0.83, 1.71)
Benzophenone-3	1965	1.02 (0.79, 1.31)	259	0.85 (0.44, 1.65)	1259	0.97 (0.72, 1.3)	806	0.96 (0.64, 1.43)
Triclosan	2096	1.08 (0.87, 1.35)	338	0.96 (0.60, 1.52)	1289	1.05 (0.81, 1.36)	822	1.36 (0.95, 1.96)
Butyl paraben	1723	0.96 (0.73, 1.25)	126	0.62 (0.21, 1.86)	945	0.90 (0.63, 1.29)	803	1.28 (0.87, 1.87)
Ethyl paraben	1439	1.09 (0.82, 1.45)	171	0.73 (0.31, 1.69)	957	1.14 (0.81, 1.61)	465	1.27 (0.72, 2.26)
Methyl Paraben	1811	0.96 (0.71, 1.31)	173	0.88 (0.40, 1.96)	962	1.05 (0.71, 1.54)	832	1.04 (0.64, 1.68)
Propyl paraben	1799	0.99 (0.77, 1.26)	170	0.71 (0.38, 1.35)	957	1.12 (0.83, 1.53)	828	0.99 (0.67, 1.44)

Outcome: Small for Gestational Age								
	Across Pregnancy		Trimester 1		Trimester 2		Trimester 3	
				
Phenol Name	N	Beta (95 % CI)	N	Beta (95 % CI)	N	Beta (95 % CI)	N	Beta (95 % CI)

Sum Bisphenols	1667	1.05 (0.65, 1.69)	701	0.92 (0.52, 1.62)	1557	0.90 (0.57, 1.40)	700	1.5 (0.89, 2.54)
Bisphenol A	2800	1.06 (0.78, 1.43)	926	0.82 (0.55, 1.21)	2030	0.95 (0.71, 1.29)	1380	1.33 (0.94, 1.88)
Bisphenol F	1724	1.12 (0.81, 1.53)	743	1.27 (0.81, 1.99)	1576	1.05 (0.74, 1.49)	705	1.06 (0.69, 1.64)
Bisphenol S	2130	1.10 (0.80, 1.53)	714	1.11 (0.75, 1.63)	1742	0.76 (0.55, 1.06)	969	**1.52 (1.08, 2.13)**
2,4-Dichlorophenol	1567	0.95 (0.67, 1.34)	48	0.11 (0.00, 6.04)	877	0.86 (0.49, 1.50)	779	0.97 (0.63, 1.51)
2,5-Dichlorophenol	1566	1.00 (0.78, 1.27)	47	0.30 (0.02, 4.90)	879	1.05 (0.74, 1.50)	778	0.96 (0.69, 1.34)
Benzophenone-3	1959	**1.29 (1.05, 1.58)**	259	1.17 (0.70, 1.95)	1259	1.19 (0.92, 1.54)	801	**1.46 (1.09, 1.96)**
Triclosan	2090	1.15 (0.96, 1.37)	338	1.02 (0.69, 1.51)	1289	1.18 (0.95, 1.47)	817	1.16 (0.89, 1.52)
Butyl paraben	1717	1.10 (0.89, 1.34)	126	1.40 (0.70, 2.80)	945	1.09 (0.84, 1.41)	798	1.04 (0.77, 1.41)
Ethyl paraben	1439	1.07 (0.85, 1.34)	171	1.19 (0.66, 2.12)	957	0.75 (0.54, 1.04)	465	**1.72 (1.21, 2.45)**
Methyl Paraben	1805	**1.32 (1.03, 1.70)**	173	1.87 (0.92, 3.80)	962	1.30 (0.92, 1.83)	827	1.11 (0.78, 1.57)
Propyl paraben	1793	1.10 (0.90, 1.34)	170	1.27 (0.76, 2.12)	957	1.14 (0.87, 1.50)	823	0.98 (0.74, 1.29)

Outcome: Large for Gestational Age								
	Across Pregnancy		Trimester 1		Trimester 2		Trimester 3	
				
Phenol Name	N	Beta (95 % CI)	N	Beta (95 % CI)	N	Beta (95 % CI)	N	Beta (95 % CI)

Sum Bisphenols	1667	1.22 (0.78, 1.91)	701	1.26 (0.65, 2.44)	1557	1.03 (0.68, 1.56)	700	1.47 (0.78, 2.78)
Bisphenol A	2800	1.08 (0.78, 1.49)	926	1.13 (0.71, 1.79)	2030	0.93 (0.67, 1.28)	1380	1.41 (0.94, 2.13)
Bisphenol F	1724	1.04 (0.78, 1.40)	743	1.38 (0.85, 2.24)	1576	1.02 (0.74, 1.40)	705	1.25 (0.76, 2.07)
Bisphenol S	2130	1.10 (0.77, 1.56)	714	0.80 (0.50, 1.28)	1742	1.18 (0.84, 1.65)	969	0.80 (0.51, 1.27)
2,4-Dichlorophenol	1567	1.11 (0.78, 1.58)	48	0.60 (0.06, 6.26)	877	1.49 (0.92, 2.41)	779	1.15 (0.71, 1.87)
2,5-Dichlorophenol	1566	1.03 (0.79, 1.34)	47	0.69 (0.08, 5.86)	879	1.25 (0.91, 1.71)	778	1.13 (0.78, 1.64)
Benzophenone-3	1959	0.81 (0.66, 1.00)	259	0.85 (0.46, 1.54)	1259	0.92 (0.73, 1.16)	801	0.85 (0.60, 1.22)
Triclosan	2090	1.13 (0.95, 1.35)	338	**0.61 (0.39, 0.96)**	1289	1.17 (0.96, 1.42)	817	**1.59 (1.18, 2.13)**
Butyl paraben	1717	0.95 (0.76, 1.19)	126	1.75 (0.87, 3.54)	945	0.90 (0.68, 1.18)	798	1.04 (0.73, 1.47)
Ethyl paraben	1439	1.05 (0.82, 1.34)	171	1.11 (0.61, 2.04)	957	1.06 (0.80, 1.40)	465	1.36 (0.81, 2.28)
Methyl Paraben	1805	0.89 (0.70, 1.15)	173	2.01 (0.93, 4.33)	962	0.79 (0.59, 1.06)	827	0.84 (0.57, 1.25)
Propyl paraben	1793	0.97 (0.79, 1.18)	170	1.63 (0.91, 2.93)	957	0.84 (0.67, 1.06)	823	0.97 (0.71, 1.33)

Outcome: Low Birthweight								
	Across Pregnancy		Trimester 1		Trimester 2		Trimester 3	
				
Phenol Name	N	Beta (95 % CI)	N	Beta (95 % CI)	N	Beta (95 % CI)	N	Beta (95 % CI)

Sum Bisphenols	119	0.76 (0.24, 2.40)	40	1.36 (0.17, 10.60)	111	0.63 (0.21, 1.91)	39	2.82 (0.34, 23.46)
Bisphenol A	195	0.87 (0.38, 2.02)	66	0.88 (0.25, 3.05)	144	0.77 (0.35, 1.70)	83	**5.96 (1.07, 33.20)**
Bisphenol F	131	1.24 (0.58, 2.65)	48	1.80 (0.42, 7.65)	115	0.63 (0.24, 1.66)	39	2.53 (0.42, 15.05)
Bisphenol S	135	0.79 (0.32, 1.92)	41	1.03 (0.24, 4.47)	116	0.73 (0.32, 1.68)	49	1.99 (0.42, 9.51)
2,4-Dichlorophenol	100	0.62 (0.15, 2.57)			58	0.21 (0.02, 1.78)	47	0.44 (0.04, 5.25)
2,5-Dichlorophenol	100	0.55 (0.18, 1.71)			58	0.39 (0.08, 1.91)	47	0.24 (0.02, 2.39)

Outcome: Low Birthweight (Preterms only)								
	Across Pregnancy		Trimester 1		Trimester 2		Trimester 3	
				
Phenol Name	N	Beta (95 % CI)	N	Beta (95 % CI)	N	Beta (95 % CI)	N	Beta (95 % CI)

Sum Bisphenols	1667	1.16 (0.62, 2.19)	701	1.33 (0.64, 2.79)	1557	0.99 (0.54, 1.79)	700	1.30 (0.63, 2.69)
Bisphenol A	2807	0.97 (0.64, 1.48)	927	0.84 (0.5, 1.4)	2031	1.01 (0.67, 1.50)	1385	1.51 (0.91, 2.53)
Bisphenol F	1724	**1.56 (1.07, 2.30)**	743	**2.11 (1.27, 3.53)**	1576	1.06 (0.67, 1.67)	705	0.86 (0.47, 1.59)
Bisphenol S	2130	0.86 (0.54, 1.38)	714	1.26 (0.75, 2.12)	1742	0.75 (0.48, 1.17)	969	**1.77 (1.07, 2.93)**
2,4-Dichlorophenol	1573	**0.57 (0.33, 0.98)**	48	0.20 (0, 71.72)	877	0.67 (0.28, 1.59)	784	1.06 (0.54, 2.08)
2,5-Dichlorophenol	1572	0.71 (0.47, 1.08)	47	0.19 (0.01, 6.81)	879	0.96 (0.58, 1.60)	783	0.91 (0.54, 1.54)
Benzophenone-3	1965	0.87 (0.63, 1.18)	259	0.80 (0.42, 1.52)	1259	0.89 (0.62, 1.29)	806	0.88 (0.54, 1.43)
Triclosan	2096	1.16 (0.89, 1.52)	338	1.24 (0.78, 1.97)	1289	1.14 (0.84, 1.56)		
Butyl paraben	1723	1.17 (0.86, 1.59)	126	1.90 (0.68, 5.37)	945	1.04 (0.70, 1.55)	803	1.25 (0.80, 1.94)
Ethyl paraben	1439	1.13 (0.80, 1.59)	171	1.02 (0.49, 2.11)	957	0.76 (0.46, 1.24)	465	**2.23 (1.32, 3.76)**
Methyl Paraben	1811	1.13 (0.77, 1.66)	173	1.19 (0.53, 2.65)	962	1.00 (0.61, 1.64)	832	1.49 (0.85, 2.64)
Propyl paraben	1799	0.97 (0.72, 1.31)	170	0.88 (0.48, 1.61)	957	1.03 (0.69, 1.52)	828	1.09 (0.70, 1.72)

Outcome: Low Birthweight (Preterms only)								
	Across Pregnancy		Trimester 1		Trimester 2		Trimester 3	
				
Phenol Name	N	Beta (95 % CI)	N	Beta (95 % CI)	N	Beta (95 % CI)	N	Beta (95 % CI)

Benzophenone-3	125	0.61 (0.26, 1.44)	19	0.00 (0.00, 6.93)	83	0.94 (0.38, 2.29)	47	0.40 (0.03, 4.52)
Triclosan	134	0.86 (0.47, 1.56)	25	0.30 (0.02, 4.39)			48	1.44 (0.33, 6.22)
Butyl paraben	113	1.45 (0.62, 3.39)			64	2.35 (0.63, 8.82)	47	1.35 (0.40, 4.63)
Ethyl paraben	92	1.11 (0.54, 2.28)			65	0.89 (0.36, 2.23)		
Methyl Paraben	120	0.97 (0.41, 2.32)			66	1.15 (0.36, 3.71)	48	3.39 (0.43, 26.85)
Propyl paraben	120	0.64 (0.32, 1.28)			66	0.72 (0.28, 1.84)	48	1.31 (0.34, 4.97)

Outcome: Low Birthweight (Terms only)								
	Across Pregnancy		Trimester 1		Trimester 2		Trimester 3	
				
Phenol Name	N	Beta (95 % CI)	N	Beta (95 % CI)	N	Beta (95 % CI)	N	Beta (95 % CI)

Sum Bisphenols	1548	1.56 (0.61, 4.00)	661	1.93 (0.70, 5.32)	1446	1.00 (0.40, 2.49)	661	1.17 (0.42, 3.25)
Bisphenol A	2612	1.27 (0.69, 2.35)	861	1.17 (0.57, 2.43)	1887	1.12 (0.60, 2.08)	1302	1.27 (0.65, 2.48)
Bisphenol F	1593	**1.94 (1.09, 3.44)**	695	**2.36 (1.11, 5.02)**	1461	1.39 (0.71, 2.72)	666	0.78 (0.33, 1.85)
Bisphenol S	1995	0.96 (0.49, 1.89)	673	1.62 (0.77, 3.38)	1626	0.69 (0.36, 1.35)	920	**2.16 (1.13, 4.14)**
2,4-Dichlorophenol	1473	0.49 (0.24, 1.03)			819	0.48 (0.12, 1.94)	737	0.62 (0.28, 1.37)
2,5-Dichlorophenol	1472	0.69 (0.44, 1.10)	45	0.09 (0.00, 33.95)	821	1.01 (0.50, 2.08)	736	0.63 (0.37, 1.09)
Benzophenone-3	1840	0.94 (0.61, 1.43)	240	1.03 (0.40, 2.64)	1176	1.02 (0.59, 1.76)	759	1.11 (0.63, 1.94)
Triclosan	1962	1.21 (0.83, 1.76)	313	1.64 (0.78, 3.45)	1204	1.14 (0.71, 1.84)	774	1.27 (0.75, 2.15)
Butyl paraben	1610	1.36 (0.91, 2.03)	117	7.49 (0.98, 57.08)	881	1.17 (0.67, 2.06)		
Ethyl paraben	1347	1.10 (0.68, 1.78)	158	1.19 (0.41, 3.48)	892	0.45 (0.20, 1.04)	445	**2.34 (1.20, 4.53)**
Methyl Paraben	1691	1.34 (0.80, 2.25)	160	1.16 (0.35, 3.85)	896	1.34 (0.62, 2.89)	784	1.56 (0.77, 3.15)
Propyl paraben	1679	1.22 (0.81, 1.84)	157	1.20 (0.48, 3.01)	891	1.42 (0.77, 2.62)	780	1.11 (0.64, 1.93)

Models adjusted for maternal race/ethnicity, parity, and education, and child sex; cohort as random effect. Blank cells reflect model non-convergence. (CI = Confidence Interval, OR = Odds Ratio).

**Bold** indicates significance at p<0.05.

## Data Availability

The ECHO Program has data sharing policies at echoprogram.org

## Abbreviations:

BW: Birth weight
BPA: bisphenol A
CI: confidence interval
ECHO: Environmental influences on Child Health Outcomes
GA: gestational age
LBW: low birth weight
PTB: preterm birth
RR: risk ratio
SA: sensitivity analysis
