# Supplementary material for: Prenatal Phenol and Paraben Exposures and Adverse Birth Outcomes: A Prospective Analysis of U.S. Births
Source: Environ Int. Author manuscript; Available in PMC 2024 May 30. (PMC11138125; doi:10.1016/j.envint.2023.108378)
Supplement: 1 [file NIHMS1960848-supplement-1.docx]

**Prenatal Phenol and Paraben Exposures and Adverse Birth Outcomes: a Prospective Analysis of U.S. Births**

Leonardo Trasande, MD, MPP^1-3+‡*^

Morgan E. Nelson, MS^4^

Akram Alshawabkeh, PhD^5+^

Emily S. Barrett, PhD^6^

Jessie P. Buckley, PhD^7,20^

Dana Dabelea, MD, PhD^8+^

Anne L. Dunlop, MD, MPH^9^

Julie B. Herbstman, PhD^10+^

John D. Meeker, PhD^11+^

Mrudula Naidu, MPH^1^

Craig Newschaffer, PhD^12+^

Amy M. Padula, PhD^13^

Megan E. Romano, PhD^14^

Douglas M. Ruden, PhD^15+^

Sheela Sathyanarayana, MD, MPH^16,17+^

Susan L. Schantz, PhD^18+^

Anne P. Starling, PhD^8, 19^

Taylor Etzel, PhD^20^

Ghassan B. Hamra, PhD^20^

^1^Department of Pediatrics, Division of Environmental Pediatrics, NYU Grossman School of Medicine, New York, NY, USA

^2^Department of Population Health, NYU Grossman School of Medicine, New York, NY, USA

^3^NYU Wagner School of Public Service, New York, NY, USA

^4^RTI International, Research Triangle Park, NC, USA

^5^Northeastern University, Boston, MA, USA

^6^Department of Biostatistics and Epidemiology, Rutgers School of Public Health, Environmental and Occupational Health Sciences Institute, Piscataway, NJ, USA

^7^Department of Environmental Health and Engineering, Johns Hopkins Bloomberg School of Public Health, Baltimore, MD, USA

^8^Lifecourse Epidemiology Adiposity and Diabetes (LEAD) Center, University of Colorado Anschutz Medical Campus, Aurora, CO, USA

^9^Department of Gynecology and Obstetrics, Emory University School of Medicine, Atlanta, GA, USA

^10^Department of Environmental Health Sciences, Columbia University Mailman School of Public Health, New York, NY, USA

^11^Department of Environmental Health Sciences, University of Michigan School of Public Health, Ann Arbor, MI, USA

^12^College of Human Health and Development, Penn State University, Hershey, PA, USA

^13^Department of Obstetrics, Gynecology and Reproductive Sciences, University of California, San Francisco, San Francisco, CA, USA

^14^Department of Epidemiology, Geisel School of Medicine at Dartmouth, Lebanon, NH, USA

^15^Department of Obstetrics and Gynecology, Wayne State University, Detroit, MI 48201

^16^Seattle Children’s Research Institute, Seattle, WA, USA

^17^Department of Pediatrics, University of Washington, Seattle, WA, USA

^18^Beckman Institute for Advanced Science and Technology, University of Illinois at Urbana-Champaign, Urbana, IL

^19^Department of Epidemiology, Gillings School of Global Public Health, University of North Carolina at Chapel Hill, Chapel Hill, NC, USA

^20^Department of Epidemiology, Johns Hopkins Bloomberg School of Public Health, Baltimore, MD, USA

on behalf of program collaborators for Environmental influences on Child Health Outcomes*

+indicates full professor status

*See Acknowledgments for full listing of collaborators

^‡^ Corresponding Author: Leonardo Trasande, MD, MPP, Department of Pediatrics, New York University School of Medicine, 227 East 30^th^ Street Rm 807, New York, NY 10016, [leonardo.trasande@nyulangone.org](mailto:leonardo.trasande@nyulangone.org), phone 646-501-2520, fax 646-754-9688

**ECHO Collaborators Acknowledgment:**

The authors wish to thank our ECHO colleagues; the medical, nursing, and program staff; and the children and families participating in the ECHO cohorts. We also acknowledge the contribution of the following ECHO program collaborators:

ECHO Components—Coordinating Center: Duke Clinical Research Institute, Durham, North Carolina: Smith PB, Newby KL; Data Analysis Center: Johns Hopkins University Bloomberg School of Public Health, Baltimore, Maryland: Jacobson LP; Research Triangle Institute, Durham, North Carolina: Catellier DJ; Person-Reported Outcomes Core: Northwestern University, Evanston, Illinois: Gershon R, Cella D.

ECHO Awardees and Cohorts—University of Georgia, Athens, GA: Cordero J; University of Tennessee Health Science Center, Memphis, TN: Tylavsky F, Mason A, Zhao Q; University of California, San Francisco, San Francisco, CA: Bush N, LeWinn KZ; AJ Drexel Autism Institute, Philadelphia, PA: Lyall K; John Hopkins Bloomberg School of Public Health, Baltimore, MD: Volk H; University of California Davis Health, MIND Institute, Davis, CA: Schmidt R; Michigan State University, East Lansing, MI: Kerver JM; Henry Ford Health, Detroit, MI: Barone C; Michigan Department of Health and Human Services, Lansing, MI: Fussman C; Michigan State University, East Lansing, MI: Paneth N; University of Michigan, Ann Arbor, MI: Elliott M; University of Minnesota, Minneapolis, MN: Nguyen R; Icahn School of Medicine at Mount Sinai, New York, NY: Swan S, Columbia University, NY: Herbstman J.

**NIH Funding Acknowledgment and Disclaimer:**

The content is solely the responsibility of the authors and does not necessarily represent the official views of the National Institutes of Health.

Research reported in this publication was supported by the National Institute of Environmental Health Sciences under award P2CES033423 and the Environmental influences on Child Health Outcomes (ECHO) program, Office of the Director, National Institutes of Health, under Award Numbers U2COD023375 (Coordinating Center), U24OD023382 (Data Analysis Center), U24OD023319 with co-funding from the Office of Behavioral and Social Science Research (PRO Core), UH3OD023285 (Kerver), UH3OD023305 (Trasande), UH3OD023251 (Alshawabkeh), UH3OD023248 (Dabelea), UH3OD023318 (Dunlop), UH3OD023271 (Karr), UH3OD023342 (Lyall), UH3OD023272 (Schantz), UH3OD023290 (Herbstman).

**Supplement Table 0: Cohorts Included in Present Study.**

| **Cohort ID** | **Cohort Name** | **N** | **Location** | **Type of Cohort** | **Target Population** | **Years of birth** | **Trimester of Phenols Sample** | **Phenols Lab** |
| --- | --- | --- | --- | --- | --- | --- | --- | --- |
| 10101 | ECHO in Puerto Rico (PROTECT) | 351 | Puerto Rico | Clinic/hospital-based | General population  Healthy women with singleton pregnancies in northern Puerto Rico | 2012-2023 | 1st, 2nd, 3rd | CDC |
| 10601 | Healthy Start | 446 | CO | Clinic/hospital-based; Community-based | General population  Healthy pregnant women without prior adverse birth outcomes | 2010-2014 | 2nd, 3rd | CDC |
| 10901 | Atlanta ECHO Cohort of Emory University | 146 | Atlanta, GA | Clinic/hospital-based | General population  Healthy, African-American, pregnant women with singletons | 2014-2021 | 1st, 2nd, 3rd | HHEAR - Emory |
| 11201 | PETALS | 367 | Northern CA | Clinic/hospital-based | General population  Pregnant women | 2014-2019 | 1st, 2nd, 3rd | CDC and California Department of Public Health |
| 11801 | New Hampshire Birth Cohort Study (NHBCS) | 106 | NH | Clinic/hospital-based | General population  Pregnant women with singletons | 2009-2023 | 1st, 2nd, 3rd | HHEAR - Wadsworth |
| 12515 | Early Autism Risk Longitudinal investigation (EARLI) | 90 | Oakland, CA Sacramento, CA Baltimore MD | Community-based | At increased likelihood of ASD due to family history  Pregnant women with at least one older child with ASD | 2002-2013 | 1st, 2nd, 3rd | CDC |
| 12901 | Archive for Research in Child Health (ARCH) | 171 | Lansing, MI | Clinic/hospital-based; Convenience sample | General population  Low-income pregnant women | 2008-2017 | 1st, 2nd, 3rd | HHEAR – Wadsworth |
| 13101 | Illinois Kids Development Study (IKIDS) | 364 | Urbana-Champaign, IL | Clinic/hospital-based | General population  Healthy pregnant women with singletons | 2014-2021 | 1st, 2nd, 3rd | CDC |
| 13301 | The NYU Children's Health and Environment Study (NYU CHES) | 541 | New York, NY | Clinic/hospital-based | General population  Pregnant women | 2016-2023 | 1st, 2nd, 3rd | HHEAR – Wadsworth |
| 20101 | The Infant Development and the Environment Study (TIDES) | 668 | Minneapolis, MN Rochester, NY San Francisco, CA Seattle, WA | Clinic/hospital-based | General population  Pregnant women | 2011-2013 | 1st, 2nd, 3rd | HHEAR – Wadsworth |
| 20201 | Columbia Center for Children's Environmental Health (CCCEH) - Mothers and Newborns (M and N) | 369 | New York, NY | Clinic/hospital-based | General population  Healthy pregnant Hispanic and African-American women with singletons | 1998-2006 | 2nd, 3rd | CDC |

**Supplement Table 1a1: Sex-Stratified Adjusted Associations of Phenol Exposures with Birth Outcomes (Continuously Measured), Male Children Only**Models adjusted for maternal race/ethnicity, parity, and education. BW for GA model removes parity from covariates.

Outcome: Gestational Age

|  | **Across Pregnancy** | | **Trimester 1** | | **Trimester 2** | | **Trimester 3** | |
| --- | --- | --- | --- | --- | --- | --- | --- | --- |
| **Phenol Name** | **N** | **Beta (95% CI)** | **N** | **Beta (95% CI)** | **N** | **Beta (95% CI)** | **N** | **Beta (95% CI)** |
| Sum Bisphenols | 836 | -0.195 (-0.548, 0.159) | 358 | -0.274 (-0.75, 0.202) | 777 | -0.246 (-0.57, 0.077) | 371 | 0.078 (-0.295, 0.452) |
| Bisphenol A | 1420 | -0.06 (-0.279, 0.16) | 485 | -0.022 (-0.36, 0.315) | 1036 | **-0.23 (-0.448, -0.012)** | 706 | 0.098 (-0.126, 0.321) |
| Bisphenol F | 871 | -0.034 (-0.257, 0.19) | 383 | -0.3 (-0.682, 0.083) | 791 | 0.205 (-0.042, 0.452) | 373 | -0.138 (-0.427, 0.15) |
| Bisphenol S | 1086 | -0.233 (-0.47, 0.003) | 364 | -0.193 (-0.527, 0.141) | 878 | -0.198 (-0.433, 0.038) | 516 | 0.072 (-0.159, 0.303) |
| 2,4-Dichlorophenol | 793 | 0.104 (-0.104, 0.311) | 22 | 0.062 (-1.369, 1.493) | 449 | -0.077 (-0.38, 0.226) | 393 | 0.039 (-0.224, 0.302) |
| 2,5-Dichlorophenol | 794 | 0.066 (-0.074, 0.206) | 22 | 0.721 (-0.936, 2.378) | 450 | -0.072 (-0.272, 0.128) | 393 | 0.033 (-0.145, 0.211) |
| Benzophenone-3 | 994 | 0.042 (-0.089, 0.173) | 141 | 0.303 (-0.115, 0.721) | 647 | **0.158 (0, 0.317)** | 406 | 0.009 (-0.175, 0.193) |
| Triclosan | 1073 | 0.026 (-0.089, 0.14) | 182 | -0.186 (-0.496, 0.124) | 673 | -0.02 (-0.156, 0.116) | 415 | 0.109 (-0.062, 0.281) |
| Butyl paraben | 871 | **0.2 (0.069, 0.331)** | 68 | -0.018 (-0.451, 0.414) | 486 | **0.325 (0.164, 0.486)** | 403 | 0.003 (-0.196, 0.201) |
| Ethyl paraben | 750 | 0.021 (-0.118, 0.161) | 87 | 0.121 (-0.305, 0.548) | 496 | 0.119 (-0.051, 0.29) | 253 | -0.062 (-0.282, 0.157) |
| Methyl Paraben | 919 | 0.093 (-0.065, 0.251) | 88 | 0.234 (-0.252, 0.721) | 498 | 0.06 (-0.126, 0.246) | 420 | 0.025 (-0.197, 0.248) |
| Propyl paraben | 913 | 0.118 (-0.007, 0.242) | 88 | 0.268 (-0.111, 0.648) | 493 | 0.06 (-0.086, 0.207) | 419 | 0.093 (-0.083, 0.269) |

Outcome: Birth Length

|  | **Across Pregnancy** | | **Trimester 1** | | **Trimester 2** | | **Trimester 3** | |
| --- | --- | --- | --- | --- | --- | --- | --- | --- |
| **Phenol Name** | **N** | **Beta (95% CI)** | **N** | **Beta (95% CI)** | **N** | **Beta (95% CI)** | **N** | **Beta (95% CI)** |
| Sum Bisphenols | 807 | -0.08 (-0.634, 0.474) | 345 | -0.286 (-0.953, 0.382) | 751 | -0.119 (-0.636, 0.398) | 359 | -0.058 (-0.663, 0.546) |
| Bisphenol A | 1328 | 0 (-0.36, 0.36) | 434 | -0.127 (-0.615, 0.362) | 977 | -0.266 (-0.62, 0.087) | 663 | 0.067 (-0.322, 0.456) |
| Bisphenol F | 839 | -0.229 (-0.577, 0.118) | 369 | **-0.696 (-1.234, -0.158)** | 764 | 0.043 (-0.349, 0.434) | 360 | -0.108 (-0.578, 0.362) |
| Bisphenol S | 1054 | 0.176 (-0.219, 0.571) | 351 | -0.126 (-0.598, 0.347) | 851 | 0.2 (-0.184, 0.584) | 502 | -0.3 (-0.707, 0.107) |
| 2,4-Dichlorophenol | 765 | 0.191 (-0.172, 0.553) | 22 | **2.535 (1.068, 4.002)** | 437 | 0.252 (-0.27, 0.774) | 375 | 0.191 (-0.285, 0.666) |
| 2,5-Dichlorophenol | 766 | **0.279 (0.036, 0.522)** | 22 | 0.411 (-1.795, 2.617) | 438 | 0.162 (-0.18, 0.504) | 375 | 0.225 (-0.094, 0.545) |
| Benzophenone-3 | 963 | **-0.288 (-0.516, -0.06)** | 141 | 0.436 (-0.165, 1.037) | 632 | **-0.305 (-0.578, -0.031)** | 388 | -0.26 (-0.591, 0.071) |
| Triclosan | 950 | -0.02 (-0.229, 0.189) | 133 | -0.184 (-0.704, 0.337) | 627 | -0.177 (-0.42, 0.066) | 385 | 0.24 (-0.08, 0.559) |
| Butyl paraben | 780 | -0.127 (-0.376, 0.121) | 31 | **-1.011 (-1.838, -0.185)** | 452 | -0.249 (-0.543, 0.045) | 381 | 0.056 (-0.313, 0.425) |
| Ethyl paraben | 641 | 0.003 (-0.262, 0.269) | 37 | 0.839 (-0.228, 1.906) | 452 | 0.128 (-0.174, 0.429) | 236 | -0.42 (-0.873, 0.034) |
| Methyl Paraben | 796 | -0.259 (-0.557, 0.038) | 38 | 0.509 (-0.683, 1.702) | 454 | **-0.419 (-0.748, -0.091)** | 389 | -0.174 (-0.594, 0.246) |
| Propyl paraben | 796 | **-0.26 (-0.489, -0.031)** | 38 | 0.05 (-0.84, 0.94) | 454 | **-0.314 (-0.569, -0.059)** | 389 | -0.172 (-0.498, 0.154) |

Outcome: Birthweight

|  | **Across Pregnancy** | | **Trimester 1** | | **Trimester 2** | | **Trimester 3** | |
| --- | --- | --- | --- | --- | --- | --- | --- | --- |
| **Phenol Name** | **N** | **Beta (95% CI)** | **N** | **Beta (95% CI)** | **N** | **Beta (95% CI)** | **N** | **Beta (95% CI)** |
| Sum Bisphenols | 836 | -61.765 (-164.77, 41.239) | 358 | -3.42 (-137.129, 130.288) | 777 | -49.395  (-143.485, 44.696) | 371 | -15.362 (-139.93, 109.207) |
| Bisphenol A | 1420 | -28.79 (-93.878, 36.298) | 485 | 44.712 (-52.605, 142.028) | 1036 | -33.495 (-98.755, 31.765) | 706 | -10.544 (-81.298, 60.21) |
| Bisphenol F | 871 | **-65.568**  **(-130.132, -1.005)** | 383 | -102.429 (-209.645, 4.787) | 791 | -3.865 (-75.429, 67.7) | 373 | -48.513 (-144.439, 47.414) |
| Bisphenol S | 1086 | -1.432 (-71.938, 69.075) | 364 | 19.585 (-74.471, 113.642) | 878 | 23.592 (-46.22, 93.405) | 516 | -23.774 (-99.668, 52.12) |
| 2,4-Dichlorophenol | 793 | 54.229 (-7.864, 116.323) | 22 | 103.975  (-291.756, 499.707) | 449 | 57.825 (-34.26, 149.909) | 393 | 48.861 (-31.26, 128.982) |
| 2,5-Dichlorophenol | 794 | 30.178 (-11.786, 72.142) | 22 | 215.953  (-244.113, 676.019) | 450 | -25.206 (-85.992, 35.58) | 393 | 27.043 (-27.093, 81.18) |
| Benzophenone-3 | 994 | -28.083 (-67.333, 11.167) | 141 | 59.326 (-62.832, 181.485) | 647 | -4.131 (-52.691, 44.428) | 406 | -47.66 (-103.411, 8.091) |
| Triclosan | 1073 | -11.377 (-46.346, 23.592) | 182 | -73.94 (-166.852, 18.971) | 673 | -9.873 (-52.191, 32.445) | 415 | 4.827 (-47.878, 57.532) |
| Butyl paraben | 871 | -10.875 (-50.911, 29.162) | 68 | **-149.128 (-296.532, -1.723)** | 486 | 8.815 (-41.688, 59.317) | 403 | -33.377 (-93.63, 26.877) |
| Ethyl paraben | 750 | -38.799 (-81.889, 4.291) | 87 | -34.361 (-171.496, 102.773) | 496 | -5.942 (-58.619, 46.736) | 253 | **-104.732**  **(-176.918, -32.545)** |
| Methyl Paraben | 919 | -36.316 (-84.423, 11.792) | 88 | 69.767 (-86.746, 226.281) | 498 | **-58.781 (-115.878, -1.684)** | 420 | -57.097 (-125.054, 10.86) |
| Propyl paraben | 913 | -18.121 (-55.943, 19.702) | 88 | 29.722 (-93.496, 152.94) | 493 | -32.359 (-77.312, 12.593) | 419 | -16.852 (-70.853, 37.15) |

Outcome: Birthweight for Gestational Age Z-Score

|  | **Across Pregnancy** | | **Trimester 1** | | **Trimester 2** | | **Trimester 3** | |
| --- | --- | --- | --- | --- | --- | --- | --- | --- |
| **Phenol Name** | **N** | **Beta (95% CI)** | **N** | **Beta (95% CI)** | **N** | **Beta (95% CI)** | **N** | **Beta (95% CI)** |
| Sum Bisphenols | 836 | -0.033 (-0.243, 0.178) | 358 | 0.098 (-0.161, 0.358) | 777 | -0.003 (-0.192, 0.186) | 371 | -0.035 (-0.29, 0.22) |
| Bisphenol A | 1418 | -0.048 (-0.188, 0.091) | 485 | 0.096 (-0.096, 0.287) | 1036 | 0.007 (-0.127, 0.14) | 704 | -0.059 (-0.216, 0.099) |
| Bisphenol F | 871 | -0.116 (-0.247, 0.016) | 383 | -0.113 (-0.323, 0.097) | 791 | -0.089 (-0.232, 0.055) | 373 | -0.018 (-0.215, 0.179) |
| Bisphenol S | 1086 | 0.093 (-0.055, 0.24) | 364 | 0.097 (-0.084, 0.278) | 878 | **0.142 (0.001, 0.283)** | 516 | -0.106 (-0.267, 0.055) |
| 2,4-Dichlorophenol | 791 | 0.108 (-0.038, 0.254) | 22 | 0.108 (-0.692, 0.908) | 449 | 0.195 (-0.015, 0.406) | 391 | 0.123 (-0.07, 0.316) |
| 2,5-Dichlorophenol | 792 | 0.074 (-0.025, 0.173) | 22 | -0.151 (-0.903, 0.602) | 450 | 0 (-0.139, 0.14) | 391 | 0.082 (-0.048, 0.213) |
| Benzophenone-3 | 992 | **-0.095 (-0.184, -0.007)** | 141 | 0.02 (-0.21, 0.251) | 647 | -0.081 (-0.186, 0.024) | 404 | **-0.134 (-0.268, -0.001)** |
| Triclosan | 1071 | -0.043 (-0.121, 0.035) | 182 | -0.121 (-0.29, 0.047) | 673 | -0.018 (-0.11, 0.074) | 413 | -0.045 (-0.171, 0.081) |
| Butyl paraben | 869 | **-0.117 (-0.211, -0.024)** | 68 | **-0.393 (-0.725, -0.061)** | 486 | **-0.122 (-0.235, -0.008)** | 401 | -0.063 (-0.208, 0.082) |
| Ethyl paraben | 750 | **-0.101 (-0.197, -0.005)** | 87 | -0.146 (-0.425, 0.133) | 496 | -0.049 (-0.167, 0.069) | 253 | **-0.235 (-0.396, -0.075)** |
| Methyl Paraben | 917 | **-0.143 (-0.253, -0.032)** | 88 | 0.02 (-0.3, 0.341) | 498 | **-0.161 (-0.288, -0.033)** | 418 | -0.156 (-0.318, 0.005) |
| Propyl paraben | 911 | **-0.099 (-0.186, -0.012)** | 88 | -0.116 (-0.368, 0.136) | 493 | -0.089 (-0.189, 0.012) | 417 | -0.078 (-0.207, 0.051) |

**Supplement Table 1b1: Sex-Stratified Adjusted Associations of Phenol Exposures with Categorical Birth Outcomes, Male Children Only**Models adjusted for maternal race/ethnicity, parity, and education. Blank cells reflect model non-convergence.

Outcome: Preterm

|  | **Across Pregnancy** | | **Trimester 1** | | **Trimester 2** | | **Trimester 3** | |
| --- | --- | --- | --- | --- | --- | --- | --- | --- |
| **Phenol Name** | **N** | **OR (95% CI)** | **N** | **OR (95% CI)** | **N** | **OR (95% CI)** | **N** | **OR (95% CI)** |
| Sum Bisphenols | 836 | **2.18 (1.09, 4.36)** | 358 | 0.98 (0.39, 2.47) | 777 | **1.98 (1.02, 3.86)** | 371 | 1.34 (0.48, 3.76) |
| Bisphenol A | 1420 | 1.09 (0.66, 1.78) | 485 | 0.62 (0.32, 1.2) | 1036 | 1.37 (0.85, 2.22) | 706 | 1.04 (0.56, 1.95) |
| Bisphenol F | 871 | **1.76 (1.16, 2.67)** | 383 | 1.38 (0.75, 2.53) | 791 | 1.25 (0.75, 2.08) | 373 | **2.52 (1.15, 5.5)** |
| Bisphenol S | 1086 | 1.48 (0.85, 2.57) | 364 | 0.9 (0.47, 1.76) | 878 | 1.56 (0.91, 2.67) | 516 | 0.84 (0.41, 1.7) |
| 2,4-Dichlorophenol | 793 | 0.91 (0.52, 1.59) |  |  | 449 | 0.94 (0.41, 2.19) | 393 | 1.61 (0.78, 3.34) |
| 2,5-Dichlorophenol | 794 | 1.23 (0.86, 1.76) |  |  |  |  | 393 | 1.59 (0.95, 2.66) |
| Benzophenone-3 | 994 | 0.78 (0.55, 1.11) | 141 | 0.69 (0.26, 1.81) | 647 | 0.71 (0.46, 1.09) | 406 | 0.78 (0.44, 1.38) |
| Triclosan | 1073 | 0.83 (0.61, 1.14) | 182 | 1.04 (0.54, 2) | 673 | 0.81 (0.55, 1.2) | 415 | 0.85 (0.51, 1.43) |
| Butyl paraben | 871 | 0.81 (0.56, 1.18) | 68 | 0.47 (0.09, 2.52) | 486 | 0.67 (0.4, 1.13) | 403 | 1.28 (0.76, 2.16) |
| Ethyl paraben | 750 | 0.99 (0.69, 1.44) | 87 | 0.75 (0.24, 2.34) | 496 | 0.8 (0.49, 1.3) | 253 | 1.46 (0.73, 2.92) |
| Methyl Paraben | 919 | 0.74 (0.49, 1.11) | 88 | 0.94 (0.32, 2.72) | 498 | 0.93 (0.56, 1.56) | 420 | 0.66 (0.36, 1.24) |
| Propyl paraben | 913 | 0.8 (0.58, 1.1) | 88 | 0.66 (0.28, 1.56) | 493 | 0.97 (0.65, 1.46) | 419 | 0.81 (0.49, 1.33) |

Outcome: Small for Gestational Age

|  | **Across Pregnancy** | | **Trimester 1** | | **Trimester 2** | | **Trimester 3** | |
| --- | --- | --- | --- | --- | --- | --- | --- | --- |
| **Phenol Name** | **N** | **OR (95% CI)** | **N** | **OR (95% CI)** | **N** | **OR (95% CI)** | **N** | **OR (95% CI)** |
| Sum Bisphenols | 836 | 1.08 (0.5, 2.34) | 358 | 0.53 (0.2, 1.37) | 777 | 1.34 (0.66, 2.72) | 371 | 1.99 (0.87, 4.51) |
| Bisphenol A | 1418 | 0.99 (0.64, 1.55) | 485 | 0.64 (0.34, 1.19) | 1036 | 0.92 (0.58, 1.44) | 704 | 1.34 (0.82, 2.17) |
| Bisphenol F | 871 | 1.41 (0.88, 2.26) | 383 | 1.27 (0.64, 2.53) | 791 | 1.44 (0.85, 2.46) | 373 | 1.33 (0.71, 2.52) |
| Bisphenol S | 1086 | 0.98 (0.59, 1.62) | 364 | 0.75 (0.4, 1.4) | 878 | 0.86 (0.52, 1.43) | 516 | **1.7 (1.02, 2.82)** |
| 2,4-Dichlorophenol | 791 | 1.06 (0.67, 1.67) | 22 | 0.31 (0, 37.32) | 449 | 0.84 (0.38, 1.87) | 391 | 1.1 (0.64, 1.89) |
| 2,5-Dichlorophenol | 792 | 1.03 (0.75, 1.41) | 22 | 0.06 (0, 78.27) | 450 | 1.25 (0.76, 2.08) | 391 | 1.05 (0.72, 1.52) |
| Benzophenone-3 | 992 | 1.29 (0.97, 1.72) | 141 | 0.79 (0.36, 1.74) | 647 | 1.07 (0.73, 1.56) | 404 | **1.76 (1.2, 2.59)** |
| Triclosan | 1071 | 1.22 (0.95, 1.57) | 182 | 0.97 (0.56, 1.69) | 673 | 1.25 (0.92, 1.71) | 413 | 1.31 (0.89, 1.91) |
| Butyl paraben | 869 | **1.45 (1.11, 1.91)** | 68 | 1.93 (0.78, 4.75) | 486 | **1.57 (1.08, 2.28)** | 401 | 1.2 (0.8, 1.81) |
| Ethyl paraben | 750 | 1.18 (0.86, 1.62) | 87 | 1.42 (0.62, 3.28) | 496 | 0.93 (0.59, 1.47) | 253 | **1.95 (1.19, 3.2)** |
| Methyl Paraben | 917 | **1.61 (1.11, 2.33)** | 88 | 1.69 (0.6, 4.76) | 498 | **1.72 (1.03, 2.88)** | 418 | 1.52 (0.92, 2.5) |
| Propyl paraben | 911 | **1.35 (1.01, 1.79)** | 88 | 2.11 (0.92, 4.84) | 493 | 1.31 (0.89, 1.91) | 417 | 1.27 (0.86, 1.89) |

Outcome: Large for Gestational Age

|  | **Across Pregnancy** | | **Trimester 1** | | **Trimester 2** | | **Trimester 3** | |
| --- | --- | --- | --- | --- | --- | --- | --- | --- |
| **Phenol Name** | **N** | **OR (95% CI)** | **N** | **OR (95% CI)** | **N** | **OR (95% CI)** | **N** | **OR (95% CI)** |
| Sum Bisphenols | 836 | 1.06 (0.56, 2.01) | 358 | 1.3 (0.56, 3.02) | 777 | 0.87 (0.48, 1.58) | 371 | 1.35 (0.56, 3.21) |
| Bisphenol A | 1418 | 0.88 (0.57, 1.36) | 485 | 1.23 (0.69, 2.21) | 1036 | 0.9 (0.59, 1.36) | 704 | 1.07 (0.62, 1.85) |
| Bisphenol F | 871 | 0.9 (0.6, 1.35) | 383 | 1.36 (0.74, 2.51) | 791 | 0.9 (0.57, 1.42) | 373 | 1.01 (0.53, 1.96) |
| Bisphenol S | 1086 | 1.43 (0.89, 2.32) | 364 | 0.83 (0.44, 1.54) | 878 | 1.47 (0.94, 2.3) | 516 | 0.75 (0.4, 1.4) |
| 2,4-Dichlorophenol | 791 | 1.35 (0.84, 2.16) |  |  | 449 | 1.77 (0.9, 3.47) | 391 | 1.28 (0.66, 2.48) |
| 2,5-Dichlorophenol | 792 | 1.18 (0.85, 1.64) | 22 | 0.52 (0, 33339.73) | 450 | 1.37 (0.88, 2.16) | 391 | 1.07 (0.68, 1.68) |
| Benzophenone-3 | 992 | 0.83 (0.62, 1.11) | 141 | 1.45 (0.69, 3.06) | 647 | 0.83 (0.6, 1.15) | 404 | 1.02 (0.61, 1.7) |
| Triclosan | 1071 | 0.98 (0.77, 1.25) | 182 | 0.56 (0.29, 1.08) | 673 | 0.95 (0.72, 1.25) | 413 | **1.6 (1.02, 2.52)** |
| Butyl paraben | 869 | 0.83 (0.6, 1.15) |  |  | 486 | 0.81 (0.55, 1.21) | 401 | 1.01 (0.59, 1.74) |
| Ethyl paraben | 750 | 0.86 (0.6, 1.22) | 87 | 0.86 (0.28, 2.62) | 496 | 0.82 (0.55, 1.23) | 253 | 1.63 (0.78, 3.42) |
| Methyl Paraben | 917 | 0.83 (0.57, 1.19) | 88 | 7.52 (0.79, 71.81) | 498 | 0.68 (0.45, 1.03) | 418 | 0.74 (0.4, 1.37) |
| Propyl paraben | 911 | 0.87 (0.65, 1.16) | 88 | 2.36 (0.75, 7.5) | 493 | 0.82 (0.59, 1.13) | 417 | 0.94 (0.58, 1.52) |

Outcome: Low Birthweight

|  | **Across Pregnancy** | | **Trimester 1** | | **Trimester 2** | | **Trimester 3** | |
| --- | --- | --- | --- | --- | --- | --- | --- | --- |
| **Phenol Name** | **N** | **OR (95% CI)** | **N** | **OR (95% CI)** | **N** | **OR (95% CI)** | **N** | **OR (95% CI)** |
| Sum Bisphenols | 836 | 1.11 (0.4, 3.04) | 358 | 0.91 (0.3, 2.82) | 777 | 1.3 (0.5, 3.33) | 371 | 2.13 (0.67, 6.79) |
| Bisphenol A | 1420 | 1.19 (0.61, 2.29) | 485 | 0.7 (0.32, 1.54) | 1036 | 1.39 (0.75, 2.6) | 706 | 2.16 (0.91, 5.14) |
| Bisphenol F | 871 | 1.61 (0.89, 2.9) | 383 | 1.76 (0.86, 3.61) | 791 | 1.01 (0.47, 2.19) | 373 | 1.42 (0.58, 3.48) |
| Bisphenol S | 1086 | 1.28 (0.59, 2.78) | 364 | 0.82 (0.36, 1.86) | 878 | 0.87 (0.43, 1.77) | 516 | **3.2 (1.26, 8.12)** |
| 2,4-Dichlorophenol | 793 | 0.97 (0.44, 2.13) |  |  | 449 | 1.66 (0.54, 5.08) | 393 |  |
| 2,5-Dichlorophenol | 794 | 1.06 (0.63, 1.78) |  |  | 450 | **2.25 (1.06, 4.78)** | 393 |  |
| Benzophenone-3 | 994 | 0.62 (0.37, 1.06) |  |  | 647 | 0.56 (0.29, 1.1) | 406 |  |
| Triclosan | 1073 | 1.14 (0.76, 1.72) | 182 | **1.96 (1.05, 3.64)** | 673 | 1.01 (0.6, 1.7) | 415 |  |
| Butyl paraben |  |  | 68 | 3.85 (0.87, 16.99) | 486 | 1.15 (0.56, 2.33) | 403 |  |
| Ethyl paraben | 750 | 1.62 (0.99, 2.66) | 87 | 1.84 (0.66, 5.18) | 496 | 0.53 (0.2, 1.41) | 253 | **6.77 (2.28, 20.08)** |
| Methyl Paraben | 919 | 1.88 (0.99, 3.57) | 88 | 1.48 (0.45, 4.86) | 498 | 1.94 (0.75, 5) | 420 |  |
| Propyl paraben | 913 | 1.09 (0.68, 1.76) | 88 | 1.2 (0.52, 2.79) | 493 | 1.03 (0.52, 2.02) | 419 |  |

Outcome: Low Birthweight (Preterms only)

|  | **Across Pregnancy** | | **Trimester 1** | | **Trimester 2** | | **Trimester 3** | |
| --- | --- | --- | --- | --- | --- | --- | --- | --- |
| **Phenol Name** | **N** | **OR (95% CI)** | **N** | **OR (95% CI)** | **N** | **OR (95% CI)** | **N** | **OR (95% CI)** |
| Sum Bisphenols | 69 | 0.63 (0.11, 3.65) | 29 | 3.24 (0.2, 52.71) | 64 | 0.63 (0.15, 2.68) | 22 | 6.67 (0.08, 556.84) |
| Bisphenol A | 109 | 0.76 (0.26, 2.25) | 44 | 2.22 (0.45, 10.97) | 80 | 0.9 (0.36, 2.27) | 45 | 13.62 (0.9, 206.24) |
| Bisphenol F | 78 | 1.17 (0.38, 3.65) | 35 | 3.2 (0.34, 30.29) | 67 | 0.99 (0.32, 3.12) | 22 | 1.21 (0.05, 29.92) |
| Bisphenol S | 79 | 1 (0.27, 3.64) | 29 | 2.5 (0.29, 21.69) | 66 | 0.75 (0.26, 2.14) | 30 | 7.75 (0.53, 114.1) |
| 2,4-Dichlorophenol | 53 | 0.72 (0.12, 4.23) |  |  | 30 | 45.66 (0.04, 55858.41) | 25 | 0.36 (0.03, 4.58) |
| 2,5-Dichlorophenol | 53 | 0.54 (0.14, 2) |  |  | 30 | 11.28 (0.17, 728.77) | 25 | 0.45 (0.08, 2.5) |
| Benzophenone-3 | 65 | 0.72 (0.21, 2.44) |  |  | 42 | 0.6 (0.11, 3.16) | 25 | 1.41 (0.12, 17.19) |
| Triclosan | 70 | 1.68 (0.78, 3.62) |  |  |  |  | 26 | 1.42 (0.19, 10.32) |
| Butyl paraben | 62 | 2.11 (0.74, 6.02) |  |  | 34 | 16.38 (0.81, 332.61) | 25 | 1.26 (0.32, 5.01) |
| Ethyl paraben | 54 | 1.47 (0.64, 3.42) |  |  |  |  |  |  |
| Methyl Paraben | 66 | 1.94 (0.65, 5.82) |  |  | 35 | 4.2 (0.51, 34.64) | 26 | 4.86 (0.3, 79.18) |
| Propyl paraben | 66 | 0.87 (0.38, 1.96) |  |  |  |  | 26 | 1.46 (0.21, 10.13) |

Outcome: Low Birthweight (Terms only)

|  | **Across Pregnancy** | | **Trimester 1** | | **Trimester 2** | | **Trimester 3** | |
| --- | --- | --- | --- | --- | --- | --- | --- | --- |
| **Phenol Name** | **N** | **OR (95% CI)** | **N** | **OR (95% CI)** | **N** | **OR (95% CI)** | **N** | **OR (95% CI)** |
| Sum Bisphenols | 767 | 0.37 (0.04, 3.93) | 329 | 0.15 (0.01, 2.53) | 713 | 0.57 (0.07, 4.6) | 349 | 2.23 (0.41, 12.11) |
| Bisphenol A | 1311 | 1.7 (0.57, 5.07) | 441 | 0.53 (0.12, 2.4) | 956 | 1.61 (0.52, 4.99) |  |  |
| Bisphenol F | 793 | 0.87 (0.24, 3.21) | 348 | 0.89 (0.17, 4.56) | 724 | 0.21 (0.02, 2.15) | 351 | 0.67 (0.14, 3.29) |
| Bisphenol S | 1007 | 1.08 (0.28, 4.19) | 335 | 0.87 (0.18, 4.15) | 812 | 0.38 (0.1, 1.44) | 486 | **4.89 (1.1, 21.83)** |
| 2,4-Dichlorophenol | 740 | 0.89 (0.3, 2.62) |  |  | 419 | 0.76 (0.08, 7.53) |  |  |
| 2,5-Dichlorophenol | 741 | 1.21 (0.6, 2.47) |  |  | 420 | 2.65 (0.72, 9.73) |  |  |
| Benzophenone-3 | 929 | 0.81 (0.39, 1.7) | 131 | 0.42 (0.05, 3.4) | 605 | 0.91 (0.29, 2.91) |  |  |
| Triclosan | 1003 | 1.06 (0.56, 2.01) | 168 | 45.04 (0.45, 4476.83) | 631 | 0.87 (0.33, 2.27) |  |  |
| Butyl paraben | 809 | 1.65 (0.9, 3.05) |  |  | 452 | 1.21 (0.33, 4.49) |  |  |
| Ethyl paraben | 696 | 2.11 (0.97, 4.6) | 79 | 3.14 (0.43, 22.96) | 461 | 0.11 (0.01, 2.23) | 239 | **18.48 (1.82, 187.24)** |
| Methyl Paraben | 853 | **3.8 (1.37, 10.58)** | 80 | 1.43 (0.16, 13) | 463 | 7.01 (0.9, 54.84) | 394 | **5.14 (1.22, 21.67)** |
| Propyl paraben | 847 | 1.88 (0.9, 3.96) | 80 | 3.57 (0.58, 22.03) | 458 | 1.22 (0.37, 4.06) |  |  |

**Supplement Table 1a2: Sex-Stratified Adjusted Associations of Phenol Exposures with Birth Outcomes (Continuously Measured), Female Children Only**Models adjusted for maternal race/ethnicity, parity, and education. BW for GA model removes parity from covariates.

Outcome: Gestational Age

|  | **Across Pregnancy** | | **Trimester 1** | | **Trimester 2** | | **Trimester 3** | |
| --- | --- | --- | --- | --- | --- | --- | --- | --- |
| **Phenol Name** | **N** | **Beta (95% CI)** | **N** | **Beta (95% CI)** | **N** | **Beta (95% CI)** | **N** | **Beta (95% CI)** |
| Sum Bisphenols | 831 | 0.194 (-0.108, 0.496) | 343 | -0.034 (-0.435, 0.368) | 780 | 0.039 (-0.242, 0.319) | 329 | 0.007 (-0.35, 0.364) |
| Bisphenol A | 1387 | **0.216 (0.011, 0.42)** | 442 | 0.005 (-0.324, 0.334) | 995 | 0.12 (-0.096, 0.335) | 679 | 0.037 (-0.191, 0.266) |
| Bisphenol F | 853 | 0.059 (-0.149, 0.267) | 360 | -0.038 (-0.371, 0.296) | 785 | 0.039 (-0.185, 0.263) | 332 | 0.263 (-0.044, 0.57) |
| Bisphenol S | 1044 | -0.086 (-0.293, 0.12) | 350 | **-0.306 (-0.586, -0.025)** | 864 | -0.003 (-0.218, 0.213) | 453 | -0.221 (-0.449, 0.006) |
| 2,4-Dichlorophenol | 780 | 0.193 (-0.014, 0.401) | 26 | -0.105 (-1.286, 1.077) | 428 | 0.098 (-0.245, 0.44) | 391 | 0.199 (-0.071, 0.469) |
| 2,5-Dichlorophenol | 778 | **0.171 (0.028, 0.314)** | 25 | -0.572 (-1.578, 0.434) | 429 | 0.088 (-0.132, 0.307) | 390 | **0.212 (0.018, 0.407)** |
| Benzophenone-3 | 971 | -0.033 (-0.167, 0.1) | 118 | 0.165 (-0.287, 0.617) | 612 | -0.018 (-0.179, 0.143) | 400 | -0.016 (-0.222, 0.19) |
| Triclosan | 1023 | -0.066 (-0.188, 0.055) | 156 | 0.123 (-0.206, 0.451) | 616 | -0.051 (-0.195, 0.093) | 407 | -0.165 (-0.354, 0.024) |
| Butyl paraben | 852 | 0.049 (-0.085, 0.183) | 58 | 0.04 (-0.41, 0.49) | 459 | 0.017 (-0.152, 0.186) | 400 | 0.025 (-0.181, 0.23) |
| Ethyl paraben | 689 | -0.026 (-0.174, 0.123) | 84 | -0.063 (-0.423, 0.297) | 461 | -0.084 (-0.274, 0.105) | 212 | 0.003 (-0.237, 0.243) |
| Methyl Paraben | 892 | 0.081 (-0.072, 0.234) | 85 | 0.176 (-0.189, 0.541) | 464 | 0.1 (-0.094, 0.293) | 412 | -0.06 (-0.293, 0.173) |
| Propyl paraben | 886 | 0.068 (-0.057, 0.193) | 82 | 0.238 (-0.07, 0.546) | 464 | 0.036 (-0.12, 0.193) | 409 | -0.021 (-0.211, 0.169) |

Outcome: Birth Length

|  | **Across Pregnancy** | | **Trimester 1** | | **Trimester 2** | | **Trimester 3** | |
| --- | --- | --- | --- | --- | --- | --- | --- | --- |
| **Phenol Name** | **N** | **Beta (95% CI)** | **N** | **Beta (95% CI)** | **N** | **Beta (95% CI)** | **N** | **Beta (95% CI)** |
| Sum Bisphenols | 803 | 0.032 (-0.497, 0.56) | 327 | 0.133 (-0.545, 0.811) | 753 | -0.076 (-0.569, 0.418) | 308 | 0.059 (-0.566, 0.684) |
| Bisphenol A | 1302 | 0.218 (-0.128, 0.563) | 388 | 0.142 (-0.382, 0.665) | 937 | 0.004 (-0.354, 0.361) | 633 | 0.32 (-0.092, 0.733) |
| Bisphenol F | 823 | -0.114 (-0.486, 0.257) | 344 | -0.219 (-0.817, 0.379) | 758 | -0.115 (-0.507, 0.276) | 309 | 0.347 (-0.189, 0.884) |
| Bisphenol S | 1014 | 0.245 (-0.125, 0.616) | 334 | -0.252 (-0.748, 0.244) | 836 | 0.174 (-0.209, 0.556) | 431 | -0.221 (-0.633, 0.19) |
| 2,4-Dichlorophenol | 760 | **0.494 (0.114, 0.875)** | 26 | 2.428 (-0.642, 5.499) | 421 | 0.358 (-0.259, 0.975) | 378 | **0.577 (0.083, 1.07)** |
| 2,5-Dichlorophenol | 758 | **0.372 (0.11, 0.633)** | 25 | 0.286 (-2.449, 3.021) | 422 | 0.123 (-0.276, 0.522) | 377 | **0.513 (0.163, 0.863)** |
| Benzophenone-3 | 951 | **-0.276 (-0.516, -0.037)** | 118 | -0.189 (-0.952, 0.574) | 605 | -0.034 (-0.321, 0.252) | 387 | **-0.485 (-0.857, -0.114)** |
| Triclosan | 926 | -0.078 (-0.294, 0.138) | 107 | -0.079 (-0.704, 0.546) | 592 | -0.178 (-0.424, 0.068) | 383 | 0.104 (-0.245, 0.453) |
| Butyl paraben | 777 | 0.219 (-0.043, 0.482) | 24 | -0.43 (-1.594, 0.734) | 439 | 0.148 (-0.172, 0.469) | 379 | 0.175 (-0.203, 0.554) |
| Ethyl paraben | 602 | 0.133 (-0.178, 0.445) | 35 | -0.249 (-2.218, 1.721) | 437 | 0.178 (-0.189, 0.545) | 198 | 0.033 (-0.455, 0.52) |
| Methyl Paraben | 793 | -0.121 (-0.427, 0.186) | 36 | -0.653 (-2.083, 0.778) | 440 | 0.009 (-0.362, 0.38) | 386 | -0.285 (-0.723, 0.154) |
| Propyl paraben | 792 | -0.049 (-0.301, 0.203) | 35 | 0.407 (-0.947, 1.761) | 440 | -0.053 (-0.354, 0.249) | 386 | -0.143 (-0.497, 0.211) |

Outcome: Birthweight

|  | **Across Pregnancy** | | **Trimester 1** | | **Trimester 2** | | **Trimester 3** | |
| --- | --- | --- | --- | --- | --- | --- | --- | --- |
| **Phenol Name** | **N** | **Beta (95% CI)** | **N** | **Beta (95% CI)** | **N** | **Beta (95% CI)** | **N** | **Beta (95% CI)** |
| Sum Bisphenols | 831 | 38.301 (-55.69, 132.293) | 343 | 31.302 (-86.465, 149.068) | 780 | 56.019 (-32.08, 144.118) | 329 | 1.911 (-113.014, 116.835) |
| Bisphenol A | 1387 | **74.046 (14.495, 133.598)** | 442 | 41.78 (-46.467, 130.026) | 995 | 31.727 (-31.516, 94.97) | 679 | 62.069 (-7.131, 131.268) |
| Bisphenol F | 853 | 4.575 (-60.044, 69.194) | 360 | -12.89 (-111.93, 86.151) | 785 | 16.869 (-53.176, 86.914) | 332 | 47.604 (-51.568, 146.777) |
| Bisphenol S | 1044 | 12.711 (-51.51, 76.931) | 350 | -67.197 (-150.15, 15.757) | 864 | 48.416 (-18.868, 115.699) | 453 | -67.373 (-140.32, 5.575) |
| 2,4-Dichlorophenol | 780 | **88.386 (23.703, 153.069)** | 26 | 156.791 (-275.177, 588.76) | 428 | 113.333 (-2.119, 228.785) | 391 | **95.322 (14.946, 175.698)** |
| 2,5-Dichlorophenol | 778 | **57.167 (12.452, 101.883)** | 25 | 14.696 (-348.781, 378.173) | 429 | 33.165 (-41.261, 107.591) | 390 | **84.653 (27.13, 142.176)** |
| Benzophenone-3 | 971 | **-50.635 (-91.18, -10.09)** | 118 | -84.272 (-208.6, 40.056) | 612 | -36.715 (-87.774, 14.345) | 400 | -47.593 (-109.262, 14.076) |
| Triclosan | 1023 | -7.373 (-44.425, 29.68) | 156 | -9.158 (-102.088, 83.771) | 616 | -19.326 (-64.737, 26.084) | 407 | 8.193 (-48.986, 65.372) |
| Butyl paraben | 852 | 16.828 (-25.382, 59.038) | 58 | 113.59 (-36.39, 263.569) | 459 | 1.029 (-55.84, 57.898) | 400 | 25.876 (-35.626, 87.378) |
| Ethyl paraben | 689 | -2.372 (-52.332, 47.589) | 84 | 20.194 (-100.229, 140.618) | 461 | -1.517 (-65.983, 62.948) | 212 | -34.644 (-119.324, 50.037) |
| Methyl Paraben | 892 | -29.633 (-77.874, 18.607) | 85 | 16.97 (-105.735, 139.675) | 464 | -30.223 (-95.602, 35.155) | 412 | -36.969 (-107.309, 33.372) |
| Propyl paraben | 886 | 1.994 (-37.461, 41.448) | 82 | 92.302 (-11.161, 195.764) | 464 | -35.705 (-88.501, 17.092) | 409 | 7.566 (-49.752, 64.884) |

Outcome: Birthweight for Gestational Age Z-Score

|  | **Across Pregnancy** | | **Trimester 1** | | **Trimester 2** | | **Trimester 3** | |
| --- | --- | --- | --- | --- | --- | --- | --- | --- |
| **Phenol Name** | **N** | **Beta (95% CI)** | **N** | **Beta (95% CI)** | **N** | **Beta (95% CI)** | **N** | **Beta (95% CI)** |
| Sum Bisphenols | 831 | -0.004 (-0.215, 0.207) | 343 | 0.065 (-0.192, 0.322) | 780 | 0.114 (-0.083, 0.311) | 329 | 0.014 (-0.231, 0.259) |
| Bisphenol A | 1382 | 0.088 (-0.044, 0.22) | 441 | 0.096 (-0.084, 0.276) | 994 | 0.023 (-0.115, 0.161) | 676 | 0.144 (-0.011, 0.3) |
| Bisphenol F | 853 | -0.029 (-0.174, 0.116) | 360 | -0.03 (-0.248, 0.188) | 785 | 0.009 (-0.148, 0.165) | 332 | 0.01 (-0.201, 0.221) |
| Bisphenol S | 1044 | 0.05 (-0.096, 0.195) | 350 | -0.059 (-0.242, 0.125) | 864 | 0.116 (-0.035, 0.267) | 453 | -0.082 (-0.242, 0.078) |
| 2,4-Dichlorophenol | 776 | **0.164 (0.012, 0.317)** | 26 | 0.606 (-0.458, 1.67) | 428 | 0.223 (-0.044, 0.49) | 388 | **0.197 (0.011, 0.383)** |
| 2,5-Dichlorophenol | 774 | 0.081 (-0.024, 0.186) | 25 | 0.182 (-0.75, 1.114) | 429 | 0.016 (-0.156, 0.188) | 387 | **0.154 (0.021, 0.287)** |
| Benzophenone-3 | 967 | **-0.12 (-0.213, -0.027)** | 118 | **-0.297 (-0.537, -0.056)** | 612 | -0.088 (-0.203, 0.027) | 397 | -0.11 (-0.252, 0.032) |
| Triclosan | 1019 | 0.015 (-0.07, 0.1) | 156 | -0.088 (-0.271, 0.095) | 616 | -0.026 (-0.128, 0.076) | 404 | 0.107 (-0.024, 0.239) |
| Butyl paraben | 848 | 0.022 (-0.077, 0.121) | 58 | 0.259 (-0.071, 0.59) | 459 | -0.012 (-0.143, 0.118) | 397 | 0.058 (-0.085, 0.201) |
| Ethyl paraben | 689 | 0.001 (-0.114, 0.116) | 84 | 0.073 (-0.206, 0.351) | 461 | 0.041 (-0.106, 0.189) | 212 | -0.115 (-0.298, 0.068) |
| Methyl Paraben | 888 | -0.109 (-0.221, 0.004) | 85 | -0.035 (-0.32, 0.249) | 464 | -0.122 (-0.272, 0.027) | 409 | -0.048 (-0.211, 0.115) |
| Propyl paraben | 882 | -0.021 (-0.113, 0.071) | 82 | 0.163 (-0.077, 0.404) | 464 | -0.099 (-0.22, 0.022) | 406 | 0.028 (-0.105, 0.16) |

**Supplement Table 1b2: Sex-Stratified Adjusted Associations of Phenol Exposures with Categorical Birth Outcomes, Female Children Only**Models adjusted for maternal race/ethnicity, parity, and education. Blank cells reflect model non-convergence.

Outcome: Preterm

|  | **Across Pregnancy** | | **Trimester 1** | | **Trimester 2** | | **Trimester 3** | |
| --- | --- | --- | --- | --- | --- | --- | --- | --- |
| **Phenol Name** | **N** | **OR (95% CI)** | **N** | **OR (95% CI)** | **N** | **OR (95% CI)** | **N** | **OR (95% CI)** |
| Sum Bisphenols | 831 | 0.6 (0.26, 1.37) | 343 | 0.84 (0.18, 3.99) | 780 | 0.73 (0.34, 1.55) | 329 | 0.62 (0.2, 1.99) |
| Bisphenol A | 1387 | 0.78 (0.47, 1.29) | 442 | 0.88 (0.36, 2.13) | 995 | 0.88 (0.52, 1.5) | 679 | 0.93 (0.47, 1.84) |
| Bisphenol F | 853 | 0.91 (0.52, 1.57) | 360 | 1.6 (0.53, 4.8) | 785 | 0.84 (0.46, 1.54) | 332 | **0.23 (0.06, 0.85)** |
| Bisphenol S | 1044 | 0.76 (0.42, 1.39) | 350 | 1.69 (0.63, 4.53) | 864 | 0.68 (0.37, 1.23) | 453 | 0.89 (0.38, 2.06) |
| 2,4-Dichlorophenol | 780 | 0.85 (0.47, 1.55) |  |  | 428 | 0.61 (0.22, 1.71) | 391 | 0.86 (0.41, 1.82) |
| 2,5-Dichlorophenol | 778 | 0.84 (0.56, 1.27) |  |  | 429 | 0.61 (0.3, 1.22) | 390 | 0.9 (0.53, 1.53) |
| Benzophenone-3 | 971 | 1.23 (0.87, 1.74) | 118 | 0.93 (0.35, 2.45) | 612 | 1.21 (0.81, 1.81) | 400 | 1.15 (0.62, 2.11) |
| Triclosan | 1023 | **1.42 (1.04, 1.93)** | 156 | 0.85 (0.4, 1.8) | 616 | 1.24 (0.88, 1.75) | 407 | **2.39 (1.36, 4.19)** |
| Butyl paraben | 852 | 1.15 (0.79, 1.66) | 58 | 1.08 (0.17, 6.86) | 459 | 1.09 (0.69, 1.73) | 400 | 1.36 (0.76, 2.44) |
| Ethyl paraben | 689 | 1.1 (0.7, 1.72) | 84 | 0.87 (0.22, 3.45) | 461 | 1.37 (0.84, 2.24) | 212 | 1 (0.32, 3.08) |
| Methyl Paraben | 892 | 1.01 (0.66, 1.55) | 85 | 0.88 (0.24, 3.27) | 464 | 0.83 (0.49, 1.42) | 412 | 2.01 (0.92, 4.39) |
| Propyl paraben | 886 | 1.08 (0.76, 1.54) | 82 | 0.87 (0.31, 2.47) | 464 | 1.01 (0.66, 1.54) | 409 | 1.35 (0.74, 2.47) |

Outcome: Small for Gestational Age

|  | **Across Pregnancy** | | **Trimester 1** | | **Trimester 2** | | **Trimester 3** | |
| --- | --- | --- | --- | --- | --- | --- | --- | --- |
| **Phenol Name** | **N** | **OR (95% CI)** | **N** | **OR (95% CI)** | **N** | **OR (95% CI)** | **N** | **OR (95% CI)** |
| Sum Bisphenols | 831 | 1.12 (0.61, 2.03) | 343 | 1.23 (0.6, 2.56) | 780 | 0.73 (0.42, 1.29) | 329 | 1.29 (0.65, 2.57) |
| Bisphenol A | 1382 | 1.09 (0.74, 1.6) | 441 | 0.96 (0.57, 1.61) | 994 | 1.03 (0.7, 1.53) | 676 | 1.19 (0.76, 1.87) |
| Bisphenol F | 853 | 1.04 (0.69, 1.57) | 360 | 1.28 (0.7, 2.37) | 785 | 0.91 (0.58, 1.42) | 332 | 0.91 (0.49, 1.69) |
| Bisphenol S | 1044 | 1.03 (0.67, 1.56) | 350 | 1.39 (0.83, 2.32) | 864 | 0.7 (0.45, 1.07) | 453 | 1.41 (0.89, 2.25) |
| 2,4-Dichlorophenol | 776 | 0.65 (0.41, 1.04) |  |  | 428 | 0.71 (0.32, 1.57) | 388 | 0.61 (0.34, 1.09) |
| 2,5-Dichlorophenol | 774 | 0.76 (0.56, 1.04) | 25 | 0.09 (0, 43.31) | 429 | 0.96 (0.59, 1.55) | 387 | **0.66 (0.44, 0.98)** |
| Benzophenone-3 | 967 | **1.36 (1.04, 1.78)** | 118 | 1.72 (0.84, 3.5) | 612 | 1.36 (0.97, 1.9) | 397 | 1.31 (0.87, 1.97) |
| Triclosan | 1019 | 1.1 (0.86, 1.42) | 156 | 1.01 (0.57, 1.81) | 616 | 1.23 (0.91, 1.65) | 404 | 1.02 (0.69, 1.5) |
| Butyl paraben | 848 | 0.87 (0.65, 1.18) | 58 | 0.49 (0.05, 4.84) | 459 | 0.96 (0.67, 1.38) | 397 | 0.77 (0.49, 1.21) |
| Ethyl paraben | 689 | 1.03 (0.74, 1.44) | 84 | 1.1 (0.41, 2.94) | 461 | 0.75 (0.48, 1.16) | 212 | 1.55 (0.92, 2.59) |
| Methyl Paraben | 888 | 1.18 (0.85, 1.65) | 85 | 2.72 (0.83, 8.95) | 464 | 1.28 (0.83, 1.98) | 409 | 0.79 (0.49, 1.28) |
| Propyl paraben | 882 | 1 (0.77, 1.31) | 82 | 0.82 (0.36, 1.88) | 464 | 1.29 (0.9, 1.83) | 406 | 0.74 (0.5, 1.09) |

Outcome: Large for Gestational Age

|  | **Across Pregnancy** | | **Trimester 1** | | **Trimester 2** | | **Trimester 3** | |
| --- | --- | --- | --- | --- | --- | --- | --- | --- |
| **Phenol Name** | **N** | **OR (95% CI)** | **N** | **OR (95% CI)** | **N** | **OR (95% CI)** | **N** | **OR (95% CI)** |
| Sum Bisphenols | 831 | 1.35 (0.74, 2.48) | 343 | 1.09 (0.39, 3.02) | 780 | 1.28 (0.74, 2.22) | 329 | 1.73 (0.65, 4.6) |
| Bisphenol A | 1382 | **1.58 (1.03, 2.42)** | 441 | 0.88 (0.41, 1.91) | 994 | 1.12 (0.73, 1.71) | 676 | **2.27 (1.28, 4.02)** |
| Bisphenol F | 853 | 1.18 (0.78, 1.77) | 360 | 1.32 (0.61, 2.87) | 785 | 1.07 (0.69, 1.67) | 332 | 1.6 (0.74, 3.47) |
| Bisphenol S | 1044 | 0.99 (0.62, 1.56) | 350 | 0.73 (0.35, 1.52) | 864 | 0.95 (0.6, 1.51) | 453 | 0.83 (0.42, 1.64) |
| 2,4-Dichlorophenol | 776 | 1.19 (0.78, 1.81) | 26 | 0.1 (0, 8.15) | 428 | 1.66 (0.87, 3.18) | 388 | 1.19 (0.68, 2.07) |
| 2,5-Dichlorophenol | 774 | 1.17 (0.88, 1.57) | 25 | 0.05 (0, 5.5) | 429 | 1.21 (0.78, 1.86) | 387 | 1.31 (0.86, 1.99) |
| Benzophenone-3 | 967 | **0.72 (0.55, 0.94)** | 118 | 0.24 (0.05, 1.28) | 612 | 0.89 (0.65, 1.21) | 397 | 0.65 (0.41, 1.05) |
| Triclosan | 1019 | 1.24 (0.97, 1.57) | 156 | 0.61 (0.28, 1.36) | 616 | 1.28 (0.97, 1.7) | 404 | **1.6 (1.08, 2.37)** |
| Butyl paraben | 848 | 1 (0.77, 1.31) | 58 | 3.13 (0.89, 11.03) | 459 | 0.85 (0.61, 1.19) | 397 | 1.13 (0.73, 1.76) |
| Ethyl paraben | 689 | 1.11 (0.81, 1.53) | 84 | 1.2 (0.57, 2.52) | 461 | 1.08 (0.75, 1.54) | 212 | 1.17 (0.55, 2.49) |
| Methyl Paraben | 888 | 0.82 (0.61, 1.12) | 85 | 1.45 (0.6, 3.54) | 464 | **0.65 (0.44, 0.95)** | 409 | 0.88 (0.53, 1.48) |
| Propyl paraben | 882 | 0.93 (0.72, 1.19) | 82 | 1.27 (0.62, 2.6) | 464 | **0.67 (0.49, 0.91)** | 406 | 0.98 (0.65, 1.49) |

Outcome: Low Birthweight

|  | **Across Pregnancy** | | **Trimester 1** | | **Trimester 2** | | **Trimester 3** | |
| --- | --- | --- | --- | --- | --- | --- | --- | --- |
| **Phenol Name** | **N** | **OR (95% CI)** | **N** | **OR (95% CI)** | **N** | **OR (95% CI)** | **N** | **OR (95% CI)** |
| Sum Bisphenols | 831 | 1.22 (0.54, 2.73) | 343 | 1.81 (0.67, 4.85) | 780 | 0.86 (0.41, 1.82) | 329 | 1.02 (0.4, 2.61) |
| Bisphenol A | 1387 | 0.8 (0.48, 1.35) | 442 | 0.93 (0.48, 1.83) | 995 | 0.8 (0.48, 1.35) | 679 | 0.98 (0.52, 1.83) |
| Bisphenol F | 853 | 1.55 (0.94, 2.57) | 360 | **2.37 (1.11, 5.03)** | 785 | 1.1 (0.63, 1.93) | 332 | 0.67 (0.28, 1.64) |
| Bisphenol S | 1044 | 0.73 (0.41, 1.3) | 350 | 1.89 (0.94, 3.82) | 864 | 0.7 (0.4, 1.24) | 453 | 1.47 (0.8, 2.71) |
| 2,4-Dichlorophenol | 780 | **0.37 (0.17, 0.82)** |  |  | 428 | **0.24 (0.07, 0.87)** | 391 | 0.61 (0.25, 1.49) |
| 2,5-Dichlorophenol | 778 | **0.49 (0.3, 0.81)** | 25 | 0.09 (0, 43.31) | 429 | **0.45 (0.21, 0.97)** | 390 | 0.53 (0.28, 1.01) |
| Benzophenone-3 | 971 | 1.07 (0.73, 1.57) | 118 | 1.17 (0.52, 2.61) | 612 | 1.11 (0.71, 1.75) | 400 | 1.04 (0.56, 1.95) |
| Triclosan | 1023 | 1.17 (0.82, 1.67) | 156 | 0.59 (0.24, 1.48) | 616 | 1.21 (0.82, 1.79) | 407 | 1.69 (0.95, 3) |
| Butyl paraben | 852 | 0.97 (0.63, 1.49) | 58 | 0.01 (0, 3236071.53) | 459 | 1 (0.61, 1.64) | 400 | 1.26 (0.69, 2.3) |
| Ethyl paraben | 689 | 0.79 (0.48, 1.28) | 84 | 0.48 (0.11, 2.01) | 461 | 0.87 (0.48, 1.58) | 212 | 1.1 (0.5, 2.41) |
| Methyl Paraben | 892 | 0.77 (0.48, 1.23) | 85 | 1 (0.32, 3.13) | 464 | 0.73 (0.39, 1.36) | 412 | 1.04 (0.51, 2.11) |
| Propyl paraben | 886 | 0.89 (0.6, 1.31) | 82 | 0.61 (0.23, 1.67) | 464 | 1.08 (0.66, 1.76) | 409 | 0.9 (0.5, 1.62) |

Outcome: Low Birthweight (Preterms only)

|  | **Across Pregnancy** | | **Trimester 1** | | **Trimester 2** | | **Trimester 3** | |
| --- | --- | --- | --- | --- | --- | --- | --- | --- |
| **Phenol Name** | **N** | **OR (95% CI)** | **N** | **OR (95% CI)** | **N** | **OR (95% CI)** | **N** | **OR (95% CI)** |
| Sum Bisphenols | 50 | 0.9 (0.2, 4.07) |  |  | 47 | 0.68 (0.11, 4.1) | 17 | 3.46 (0.08, 148.29) |
| Bisphenol A | 86 | 0.49 (0.17, 1.4) | 22 | 0 (0, 2979801.07) | 64 | 0.41 (0.1, 1.66) | 38 | 2.79 (0.54, 14.41) |
| Bisphenol F | 53 | 0.98 (0.33, 2.9) |  |  | 48 | 0.42 (0.08, 2.33) |  |  |
| Bisphenol S | 56 | 0.6 (0.17, 2.18) |  |  | 50 | 0.69 (0.18, 2.59) | 19 | 0.43 (0.02, 10.67) |
| 2,4-Dichlorophenol | 47 | 0.2 (0.03, 1.37) |  |  | 28 | 0 (0, 1.74) |  |  |
| 2,5-Dichlorophenol | 47 | **0.18 (0.04, 0.93)** |  |  | 28 | 0 (0, 2.37) |  |  |
| Benzophenone-3 | 60 | 1.39 (0.51, 3.83) |  |  |  |  |  |  |
| Triclosan | 64 | 0.54 (0.24, 1.19) |  |  |  |  |  |  |
| Butyl paraben |  |  |  |  | 30 | 1.17 (0.25, 5.46) |  |  |
| Ethyl paraben | 38 | 0.76 (0.18, 3.25) |  |  | 30 | 2 (0.41, 9.72) |  |  |
| Methyl Paraben | 54 | 0.41 (0.11, 1.56) |  |  | 31 | 0.82 (0.12, 5.86) |  |  |
| Propyl paraben | 54 | 0.41 (0.14, 1.23) |  |  | 31 | 0.63 (0.14, 2.85) |  |  |

Outcome: Low Birthweight (Terms only)

|  | **Across Pregnancy** | | **Trimester 1** | | **Trimester 2** | | **Trimester 3** | |
| --- | --- | --- | --- | --- | --- | --- | --- | --- |
| **Phenol Name** | **N** | **OR (95% CI)** | **N** | **OR (95% CI)** | **N** | **OR (95% CI)** | **N** | **OR (95% CI)** |
| Sum Bisphenols | 781 | 2.13 (0.74, 6.11) | 332 | **3.87 (1.18, 12.64)** | 733 | 1.2 (0.44, 3.32) | 312 | 0.82 (0.24, 2.81) |
| Bisphenol A | 1301 | 1.13 (0.54, 2.36) | 420 | 1.47 (0.62, 3.51) | 931 | 0.94 (0.45, 1.95) | 641 | 0.78 (0.35, 1.76) |
| Bisphenol F | 800 | **2.4 (1.23, 4.67)** | 347 | **3.35 (1.33, 8.45)** | 737 | 1.87 (0.91, 3.84) | 315 | 0.83 (0.27, 2.51) |
| Bisphenol S | 988 | 0.92 (0.43, 1.97) | 338 | 2.14 (0.89, 5.16) | 814 | 0.9 (0.42, 1.95) | 434 | 1.63 (0.78, 3.4) |
| 2,4-Dichlorophenol | 733 | **0.34 (0.12, 0.95)** |  |  | 400 | 0.27 (0.04, 1.74) | 369 | 0.49 (0.16, 1.55) |
| 2,5-Dichlorophenol | 731 | **0.45 (0.23, 0.88)** | 24 | 0.09 (0, 44.8) | 401 | 0.57 (0.21, 1.54) | 368 | **0.39 (0.17, 0.94)** |
| Benzophenone-3 | 911 | 1.01 (0.6, 1.69) | 109 | 1.5 (0.51, 4.37) | 571 | 1.08 (0.56, 2.12) | 378 | 1.11 (0.53, 2.33) |
| Triclosan | 959 | 1.28 (0.79, 2.07) | 145 | 0.89 (0.27, 2.89) | 573 | 1.16 (0.64, 2.1) | 385 | 1.52 (0.77, 3.01) |
| Butyl paraben | 801 | 1.17 (0.7, 1.96) |  |  | 429 | 1.19 (0.62, 2.29) | 378 | 1.22 (0.6, 2.47) |
| Ethyl paraben | 651 | 0.7 (0.36, 1.36) | 79 | 0.36 (0.04, 3.22) | 431 | 0.56 (0.22, 1.42) | 206 | 1.17 (0.45, 3.08) |
| Methyl Paraben | 838 | 0.88 (0.47, 1.63) | 80 | 1.21 (0.19, 7.79) | 433 | 0.9 (0.36, 2.25) | 390 | 0.9 (0.39, 2.07) |
| Propyl paraben | 832 | 1.05 (0.63, 1.75) | 77 | 0.38 (0.05, 2.69) | 433 | 1.78 (0.81, 3.89) | 387 | 0.84 (0.43, 1.67) |

**Supplement Table 2a1: Race/Ethnicity-Stratified Adjusted Associations of Phenol Exposures with Birth Outcomes (Continuously Measured),
Non-Hispanic White Only**Models adjusted for maternal parity, and education, and child sex. BW for GA model removes parity from covariates.

Outcome: Gestational Age

|  | **Across Pregnancy** | | **Trimester 1** | | **Trimester 2** | | **Trimester 3** | |
| --- | --- | --- | --- | --- | --- | --- | --- | --- |
| **Phenol Name** | **N** | **Beta (95% CI)** | **N** | **Beta (95% CI)** | **N** | **Beta (95% CI)** | **N** | **Beta (95% CI)** |
| Sum Bisphenols | 694 | 0.03 (-0.28, 0.341) | 284 | -0.202 (-0.598, 0.194) | 634 | -0.242 (-0.52, 0.036) | 289 | 0.121 (-0.263, 0.506) |
| Bisphenol A | 981 | 0.127 (-0.092, 0.346) | 309 | -0.116 (-0.414, 0.182) | 787 | -0.144 (-0.35, 0.061) | 425 | **0.25 (0.007, 0.493)** |
| Bisphenol F | 709 | 0.022 (-0.176, 0.219) | 300 | -0.044 (-0.363, 0.274) | 638 | -0.029 (-0.244, 0.187) | 289 | 0.026 (-0.284, 0.337) |
| Bisphenol S | 958 | **-0.207 (-0.409, -0.005)** | 297 | **-0.428 (-0.718, -0.137)** | 753 | -0.183 (-0.397, 0.03) | 423 | -0.044 (-0.277, 0.188) |
| 2,4-Dichlorophenol | 621 | 0.016 (-0.269, 0.3) | 36 | -0.092 (-0.999, 0.814) | 452 | 0.082 (-0.269, 0.433) | 160 | 0.154 (-0.286, 0.593) |
| 2,5-Dichlorophenol | 621 | -0.03 (-0.232, 0.171) | 35 | -0.174 (-1.105, 0.757) | 454 | -0.014 (-0.257, 0.228) | 160 | 0.245 (-0.053, 0.543) |
| Benzophenone-3 | 706 | -0.074 (-0.213, 0.064) | 81 | -0.144 (-0.648, 0.361) | 529 | -0.004 (-0.162, 0.155) | 177 | -0.055 (-0.272, 0.163) |
| Triclosan | 789 | -0.02 (-0.143, 0.103) | 129 | 0.018 (-0.272, 0.308) | 547 | -0.048 (-0.195, 0.098) | 189 | -0.002 (-0.205, 0.2) |
| Butyl paraben | 699 | 0.106 (-0.029, 0.241) | 70 | -0.084 (-0.523, 0.355) | 481 | 0.078 (-0.085, 0.242) | 183 | -0.019 (-0.283, 0.246) |
| Ethyl paraben | 749 | 0.044 (-0.088, 0.176) | 104 | -0.038 (-0.379, 0.304) | 491 | 0.071 (-0.089, 0.231) | 193 | -0.017 (-0.259, 0.225) |
| Methyl Paraben | 750 | -0.025 (-0.175, 0.124) | 106 | 0.175 (-0.196, 0.545) | 491 | -0.029 (-0.196, 0.139) | 193 | -0.142 (-0.38, 0.097) |
| Propyl paraben | 741 | 0.02 (-0.1, 0.14) | 104 | 0.11 (-0.191, 0.411) | 488 | 0.018 (-0.117, 0.153) | 189 | -0.079 (-0.278, 0.119) |

Outcome: Birth Length

|  | **Across Pregnancy** | | **Trimester 1** | | **Trimester 2** | | **Trimester 3** | |
| --- | --- | --- | --- | --- | --- | --- | --- | --- |
| **Phenol Name** | **N** | **Beta (95% CI)** | **N** | **Beta (95% CI)** | **N** | **Beta (95% CI)** | **N** | **Beta (95% CI)** |
| Sum Bisphenols | 670 | -0.258 (-0.789, 0.274) | 270 | -0.323 (-0.994, 0.348) | 611 | -0.279 (-0.769, 0.211) | 270 | -0.002 (-0.642, 0.639) |
| Bisphenol A | 955 | 0.015 (-0.379, 0.409) | 295 | -0.026 (-0.54, 0.488) | 763 | -0.22 (-0.589, 0.149) | 405 | 0.135 (-0.311, 0.58) |
| Bisphenol F | 685 | -0.199 (-0.543, 0.145) | 286 | -0.298 (-0.872, 0.276) | 615 | -0.118 (-0.495, 0.258) | 270 | 0.102 (-0.414, 0.618) |
| Bisphenol S | 932 | 0.028 (-0.34, 0.396) | 283 | **-0.794 (-1.315, -0.273)** | 729 | 0.055 (-0.327, 0.437) | 403 | -0.359 (-0.782, 0.065) |
| 2,4-Dichlorophenol | 615 | 0.044 (-0.505, 0.594) | 36 | 2.002 (-0.097, 4.102) | 447 | 0.374 (-0.282, 1.03) | 158 | 0.401 (-0.505, 1.307) |
| 2,5-Dichlorophenol | 615 | 0.162 (-0.226, 0.55) | 35 | -0.33 (-2.621, 1.96) | 449 | 0.194 (-0.258, 0.646) | 158 | 0.259 (-0.353, 0.871) |
| Benzophenone-3 | 699 | **-0.408 (-0.669, -0.147)** | 81 | 0.067 (-0.843, 0.977) | 523 | **-0.362 (-0.65, -0.074)** | 175 | -0.228 (-0.681, 0.225) |
| Triclosan | 671 | **-0.318 (-0.563, -0.073)** | 64 | 0.039 (-0.57, 0.648) | 513 | **-0.373 (-0.646, -0.1)** | 169 | -0.269 (-0.727, 0.19) |
| Butyl paraben | 618 | -0.011 (-0.293, 0.272) | 24 | 0.007 (-0.782, 0.797) | 456 | -0.091 (-0.411, 0.229) | 172 | -0.276 (-0.841, 0.289) |
| Ethyl paraben | 632 | 0.002 (-0.277, 0.281) | 39 | 0.411 (-1.112, 1.934) | 458 | 0.002 (-0.307, 0.311) | 173 | -0.313 (-0.85, 0.225) |
| Methyl Paraben | 633 | **-0.377 (-0.69, -0.064)** | 41 | 0.013 (-1.384, 1.411) | 458 | -0.28 (-0.601, 0.04) | 173 | **-0.559 (-1.089, -0.028)** |
| Propyl paraben | 632 | -0.233 (-0.483, 0.017) | 40 | **1.217 (0.096, 2.337)** | 458 | -0.232 (-0.489, 0.024) | 173 | **-0.444 (-0.864, -0.023)** |

Outcome: Birthweight

|  | **Across Pregnancy** | | **Trimester 1** | | **Trimester 2** | | **Trimester 3** | |
| --- | --- | --- | --- | --- | --- | --- | --- | --- |
| **Phenol Name** | **N** | **Beta (95% CI)** | **N** | **Beta (95% CI)** | **N** | **Beta (95% CI)** | **N** | **Beta (95% CI)** |
| Sum Bisphenols | 694 | -57.396 (-152.353, 37.561) | 284 | -75.989 (-196.86, 44.882) | 634 | -17.9 (-104.684, 68.884) | 289 | 38.695 (-80.588, 157.977) |
| Bisphenol A | 981 | 40.518 (-29.406, 110.441) | 309 | -8.014 (-101.854, 85.825) | 787 | 18.111 (-48.123, 84.345) | 425 | 66.996 (-10.482, 144.473) |
| Bisphenol F | 709 | -35.279 (-95.684, 25.125) | 300 | -35.247 (-133.653, 63.16) | 638 | -11.682 (-78.653, 55.288) | 289 | 5.224 (-91.193, 101.641) |
| Bisphenol S | 958 | **-65.245 (-128.806, -1.685)** | 297 | **-156.631 (-245.426, -67.835)** | 753 | -5.49 (-73.007, 62.027) | 423 | -47.457 (-121.038, 26.124) |
| 2,4-Dichlorophenol | 621 | 2.054 (-90.217, 94.325) | 36 | 51.56 (-263.275, 366.395) | 452 | **125.029 (11.093, 238.966)** | 160 | 28.125 (-124.319, 180.569) |
| 2,5-Dichlorophenol | 621 | -32.883 (-98.397, 32.63) | 35 | -27.524 (-347.831, 292.784) | 454 | -0.589 (-79.566, 78.389) | 160 | 4.501 (-99.616, 108.619) |
| Benzophenone-3 | 706 | **-64.909 (-110.089, -19.729)** | 81 | -51.887 (-211.381, 107.607) | 529 | -51.057 (-103.427, 1.314) | 177 | -31.521 (-107.148, 44.106) |
| Triclosan | 789 | -29.638 (-70.912, 11.635) | 129 | -47.946 (-144.607, 48.715) | 547 | -25.973 (-74.881, 22.935) | 189 | -28.421 (-101.727, 44.884) |
| Butyl paraben | 699 | 7.61 (-37.07, 52.29) | 70 | 46.173 (-92.639, 184.986) | 481 | 9.355 (-45.222, 63.931) | 183 | -33.363 (-124.811, 58.085) |
| Ethyl paraben | 749 | -24.629 (-68.408, 19.151) | 104 | 4.763 (-109.063, 118.589) | 491 | -23.98 (-77.076, 29.115) | 193 | -74.857 (-160.244, 10.53) |
| Methyl Paraben | 750 | **-82.466 (-131.552, -33.381)** | 106 | 76.934 (-46.055, 199.923) | 491 | **-75.779 (-130.913, -20.645)** | 193 | **-112.147 (-195.927, -28.367)** |
| Propyl paraben | 741 | **-44.564 (-84.146, -4.982)** | 104 | 75.911 (-23.559, 175.381) | 488 | **-63.418 (-107.903, -18.933)** | 189 | -47.631 (-117.733, 22.472) |

Outcome: Birthweight for Gestational Age Z-Score

|  | **Across Pregnancy** | | **Trimester 1** | | **Trimester 2** | | **Trimester 3** | |
| --- | --- | --- | --- | --- | --- | --- | --- | --- |
| **Phenol Name** | **N** | **Beta (95% CI)** | **N** | **Beta (95% CI)** | **N** | **Beta (95% CI)** | **N** | **Beta (95% CI)** |
| Sum Bisphenols | 694 | -0.148 (-0.366, 0.069) | 284 | -0.143 (-0.418, 0.132) | 634 | 0.051 (-0.146, 0.248) | 289 | 0.059 (-0.203, 0.321) |
| Bisphenol A | 981 | 0.036 (-0.124, 0.196) | 309 | -0.007 (-0.217, 0.204) | 787 | 0.094 (-0.056, 0.244) | 425 | 0.057 (-0.115, 0.229) |
| Bisphenol F | 709 | -0.081 (-0.219, 0.058) | 300 | -0.111 (-0.335, 0.114) | 638 | -0.004 (-0.156, 0.147) | 289 | 0.024 (-0.188, 0.236) |
| Bisphenol S | 958 | -0.093 (-0.239, 0.054) | 297 | **-0.237 (-0.442, -0.031)** | 753 | 0.058 (-0.096, 0.212) | 423 | -0.131 (-0.293, 0.032) |
| 2,4-Dichlorophenol | 621 | -0.034 (-0.254, 0.185) | 36 | 0.258 (-0.46, 0.977) | 452 | 0.246 (-0.025, 0.516) | 160 | -0.037 (-0.368, 0.294) |
| 2,5-Dichlorophenol | 621 | -0.063 (-0.219, 0.093) | 35 | -0.099 (-0.818, 0.62) | 454 | 0.015 (-0.171, 0.201) | 160 | -0.08 (-0.311, 0.151) |
| Benzophenone-3 | 706 | **-0.127 (-0.232, -0.022)** | 81 | -0.054 (-0.381, 0.273) | 529 | **-0.126 (-0.247, -0.004)** | 177 | -0.063 (-0.232, 0.105) |
| Triclosan | 789 | -0.073 (-0.168, 0.022) | 129 | -0.135 (-0.336, 0.065) | 547 | -0.053 (-0.166, 0.06) | 189 | -0.08 (-0.241, 0.081) |
| Butyl paraben | 699 | -0.014 (-0.118, 0.091) | 70 | 0.171 (-0.119, 0.46) | 481 | 0.009 (-0.12, 0.138) | 183 | -0.072 (-0.269, 0.125) |
| Ethyl paraben | 749 | -0.08 (-0.182, 0.021) | 104 | 0.04 (-0.212, 0.292) | 491 | -0.091 (-0.215, 0.033) | 193 | -0.187 (-0.376, 0.001) |
| Methyl Paraben | 750 | **-0.205 (-0.319, -0.091)** | 106 | 0.118 (-0.155, 0.391) | 491 | **-0.177 (-0.306, -0.048)** | 193 | **-0.242 (-0.428, -0.055)** |
| Propyl paraben | 741 | **-0.121 (-0.213, -0.03)** | 104 | 0.16 (-0.058, 0.378) | 488 | **-0.156 (-0.26, -0.053)** | 189 | -0.109 (-0.264, 0.046) |

**Supplement Table 2b1: Race/Ethnicity -Stratified Adjusted Associations of Phenol Exposures with Categorical Birth Outcomes, Non-Hispanic White Only**Models adjusted for maternal parity, and education, and child sex. Blank cells reflect model non-convergence.

Outcome: Preterm

|  | **Across Pregnancy** | | **Trimester 1** | | **Trimester 2** | | **Trimester 3** | |
| --- | --- | --- | --- | --- | --- | --- | --- | --- |
| **Phenol Name** | **N** | **OR (95% CI)** | **N** | **OR (95% CI)** | **N** | **OR (95% CI)** | **N** | **OR (95% CI)** |
| Sum Bisphenols | 694 | 1.15 (0.49, 2.74) | 284 | 1.12 (0.21, 6.06) | 634 | 1.01 (0.46, 2.26) | 289 | 0.67 (0.19, 2.42) |
| Bisphenol A | 981 | 0.53 (0.28, 1.01) | 309 | 0.83 (0.27, 2.55) | 787 | 0.85 (0.46, 1.58) | 425 | 0.52 (0.23, 1.18) |
| Bisphenol F | 709 | 1.63 (0.98, 2.72) | 300 | 1.76 (0.57, 5.41) | 638 | 1.2 (0.67, 2.17) | 289 | 1.23 (0.46, 3.28) |
| Bisphenol S | 958 | 1.27 (0.68, 2.35) | 297 | 1.72 (0.51, 5.83) | 753 | 1.06 (0.55, 2.02) | 423 | 0.73 (0.31, 1.75) |
| 2,4-Dichlorophenol | 621 | 0.48 (0.18, 1.26) |  |  | 452 | 0.34 (0.11, 1.05) | 160 | 0.29 (0.02, 4.71) |
| 2,5-Dichlorophenol | 621 | 1.28 (0.7, 2.34) |  |  | 454 | 1.06 (0.5, 2.27) | 160 | 1.1 (0.29, 4.2) |
| Benzophenone-3 | 706 | 1.21 (0.78, 1.86) | 81 | 0.86 (0.11, 6.95) | 529 | 1.06 (0.66, 1.68) | 177 | 1.78 (0.52, 6.07) |
| Triclosan | 789 | 0.97 (0.65, 1.43) | 129 | 1.02 (0.41, 2.53) | 547 | 0.84 (0.54, 1.32) | 189 | 1.2 (0.36, 3.96) |
| Butyl paraben | 699 | 1.05 (0.67, 1.63) | 70 | 1.52 (0.27, 8.39) | 481 | 1.12 (0.67, 1.87) | 183 | 2.33 (0.5, 10.86) |
| Ethyl paraben | 749 | 0.91 (0.6, 1.38) | 104 | 1.4 (0.33, 5.93) | 491 | 0.99 (0.62, 1.58) | 193 | 1.05 (0.27, 4.11) |
| Methyl Paraben | 750 | 0.86 (0.54, 1.37) | 106 | 0.67 (0.18, 2.56) | 491 | 1.01 (0.61, 1.67) | 193 | 0.98 (0.24, 3.94) |
| Propyl paraben | 741 | 0.92 (0.63, 1.34) | 104 | 0.91 (0.31, 2.71) | 488 | 1.02 (0.69, 1.51) | 189 | 0.82 (0.27, 2.52) |

Outcome: Small for Gestational Age

|  | **Across Pregnancy** | | **Trimester 1** | | **Trimester 2** | | **Trimester 3** | |
| --- | --- | --- | --- | --- | --- | --- | --- | --- |
| **Phenol Name** | **N** | **OR (95% CI)** | **N** | **OR (95% CI)** | **N** | **OR (95% CI)** | **N** | **OR (95% CI)** |
| Sum Bisphenols | 694 | 1.22 (0.53, 2.85) | 284 | 1 (0.38, 2.63) | 634 | 0.77 (0.35, 1.7) | 289 | 1.93 (0.78, 4.75) |
| Bisphenol A | 981 | 0.85 (0.49, 1.47) | 309 | 0.81 (0.39, 1.7) | 787 | 0.72 (0.43, 1.2) | 425 | 1.53 (0.8, 2.92) |
| Bisphenol F | 709 | 1.01 (0.59, 1.73) | 300 | 1.25 (0.6, 2.59) | 638 | 0.87 (0.47, 1.62) | 289 | 0.97 (0.45, 2.13) |
| Bisphenol S | 958 | 1.43 (0.86, 2.37) | 297 | **2.13 (1.07, 4.27)** | 753 | 0.76 (0.44, 1.32) | 423 | **2.48 (1.42, 4.32)** |
| 2,4-Dichlorophenol | 621 | 1.34 (0.63, 2.84) | 36 | 0.11 (0, 4.84) | 452 | 0.96 (0.36, 2.58) | 160 | 1.85 (0.6, 5.73) |
| 2,5-Dichlorophenol | 621 | 1.11 (0.65, 1.89) | 35 | 0.08 (0, 3.77) | 454 | 1.22 (0.65, 2.3) | 160 | 1.18 (0.54, 2.57) |
| Benzophenone-3 | 706 | 1.33 (0.91, 1.93) | 81 | 0.85 (0.19, 3.8) | 529 | 1.34 (0.87, 2.06) | 177 | 1.41 (0.71, 2.82) |
| Triclosan | 789 | 1.27 (0.92, 1.75) | 129 | 1.32 (0.66, 2.62) | 547 | 1.41 (0.95, 2.09) | 189 | 1.51 (0.77, 2.98) |
| Butyl paraben | 699 | 1.32 (0.95, 1.84) | 70 | 1.02 (0.33, 3.2) | 481 | 1.15 (0.78, 1.72) | 183 | 1.72 (0.72, 4.1) |
| Ethyl paraben | 749 | 1.13 (0.81, 1.59) | 104 | 1.06 (0.47, 2.41) | 491 | 0.97 (0.63, 1.5) | 193 | 2.04 (0.98, 4.25) |
| Methyl Paraben | 750 | **2.08 (1.36, 3.19)** | 106 | 1.66 (0.6, 4.55) | 491 | **2.01 (1.26, 3.22)** | 193 | 1.6 (0.65, 3.92) |
| Propyl paraben | 741 | **1.42 (1.03, 1.97)** | 104 | 0.99 (0.47, 2.06) | 488 | **1.48 (1.02, 2.14)** | 189 | 1.45 (0.71, 2.96) |

Outcome: Large for Gestational Age

|  | **Across Pregnancy** | | **Trimester 1** | | **Trimester 2** | | **Trimester 3** | |
| --- | --- | --- | --- | --- | --- | --- | --- | --- |
| **Phenol Name** | **N** | **OR (95% CI)** | **N** | **OR (95% CI)** | **N** | **OR (95% CI)** | **N** | **OR (95% CI)** |
| Sum Bisphenols | 694 | 0.76 (0.41, 1.38) | 284 | 0.64 (0.25, 1.6) | 634 | 0.95 (0.56, 1.63) | 289 | 1.94 (0.82, 4.6) |
| Bisphenol A | 981 | 0.97 (0.61, 1.55) | 309 | 0.89 (0.47, 1.69) | 787 | 0.96 (0.63, 1.47) | 425 | 1.82 (0.94, 3.55) |
| Bisphenol F | 709 | 0.88 (0.6, 1.29) | 300 | 1.22 (0.6, 2.48) | 638 | 0.91 (0.6, 1.4) | 289 | 1.51 (0.75, 3.04) |
| Bisphenol S | 958 | 0.83 (0.54, 1.29) | 297 | 0.57 (0.29, 1.13) | 753 | 0.93 (0.6, 1.44) | 423 | 0.69 (0.37, 1.29) |
| 2,4-Dichlorophenol | 621 | 0.93 (0.49, 1.76) | 36 | 0.19 (0.01, 5.04) | 452 | 1.57 (0.78, 3.17) | 160 | 2.76 (0.65, 11.68) |
| 2,5-Dichlorophenol | 621 | 0.93 (0.59, 1.47) | 35 | 0.26 (0.02, 3.92) | 454 | 1.16 (0.72, 1.88) | 160 | 1.91 (0.83, 4.44) |
| Benzophenone-3 | 706 | **0.65 (0.48, 0.88)** | 81 | 1.32 (0.45, 3.92) | 529 | 0.76 (0.55, 1.05) | 177 | 0.92 (0.4, 2.1) |
| Triclosan | 789 | 0.88 (0.67, 1.15) | 129 | **0.52 (0.28, 0.97)** | 547 | 1.02 (0.76, 1.36) | 189 | 2.1 (0.96, 4.59) |
| Butyl paraben | 699 | 0.94 (0.7, 1.27) | 70 | 2.02 (0.69, 5.86) | 481 | 0.85 (0.6, 1.2) | 183 | 1.59 (0.63, 4.03) |
| Ethyl paraben | 749 | 0.93 (0.69, 1.24) | 104 | 1.59 (0.78, 3.22) | 491 | 0.76 (0.54, 1.07) | 193 | 1.63 (0.7, 3.84) |
| Methyl Paraben | 750 | 0.76 (0.55, 1.06) | 106 | 2.7 (0.99, 7.37) | 491 | **0.57 (0.4, 0.81)** | 193 | 1.06 (0.42, 2.72) |
| Propyl paraben | 741 | 0.8 (0.61, 1.04) | 104 | 1.7 (0.8, 3.57) | 488 | **0.62 (0.47, 0.82)** | 189 | 1.4 (0.65, 3.01) |

Outcome: Low Birthweight

|  | **Across Pregnancy** | | **Trimester 1** | | **Trimester 2** | | **Trimester 3** | |
| --- | --- | --- | --- | --- | --- | --- | --- | --- |
| **Phenol Name** | **N** | **OR (95% CI)** | **N** | **OR (95% CI)** | **N** | **OR (95% CI)** | **N** | **OR (95% CI)** |
| Sum Bisphenols | 694 | 0.75 (0.17, 3.23) | 284 | 2.11 (0.44, 10.26) | 634 | 0.63 (0.18, 2.22) | 289 | 2.03 (0.48, 8.55) |
| Bisphenol A | 981 | 0.49 (0.21, 1.13) | 309 | 0.89 (0.26, 3.06) | 787 | 0.46 (0.2, 1.06) | 425 | 1.19 (0.41, 3.47) |
| Bisphenol F | 709 | 1.8 (0.81, 4.01) | 300 | 2.09 (0.73, 5.96) | 638 | 0.9 (0.34, 2.39) | 289 | 1.44 (0.43, 4.83) |
| Bisphenol S | 958 | 0.95 (0.41, 2.2) | 297 | **4.76 (1.53, 14.85)** | 753 | 0.69 (0.27, 1.75) | 423 | 2.17 (0.92, 5.13) |
| 2,4-Dichlorophenol | 621 | 0.35 (0.07, 1.89) |  |  | 452 | **0.07 (0.01, 0.81)** | 160 | 1.83 (0.1, 34.7) |
| 2,5-Dichlorophenol | 621 | 0.55 (0.16, 1.92) | 35 | 0.28 (0, 136.02) | 454 | 0.99 (0.18, 5.44) | 160 | 0.28 (0.01, 5.64) |
| Benzophenone-3 | 706 | 1 (0.51, 1.98) | 81 | 0.55 (0.07, 4.48) |  |  | 177 | 0.85 (0.2, 3.59) |
| Triclosan | 789 | 1.11 (0.59, 2.08) | 129 | 1.31 (0.46, 3.71) |  |  | 189 | 2.76 (0.46, 16.48) |
| Butyl paraben | 699 | 1.45 (0.74, 2.82) | 70 | 2.43 (0.23, 25.22) | 481 | 0.85 (0.31, 2.35) | 183 | 1.1 (0.32, 3.79) |
| Ethyl paraben | 749 | 0.72 (0.37, 1.43) | 104 | 0.67 (0.17, 2.7) | 491 | 0.74 (0.28, 1.92) | 193 | 1.58 (0.34, 7.34) |
| Methyl Paraben | 750 | 1.55 (0.75, 3.21) | 106 | 0.8 (0.23, 2.77) | 491 | 1.44 (0.54, 3.84) | 193 | 2.63 (0.48, 14.31) |
| Propyl paraben | 741 | 1.05 (0.6, 1.84) | 104 | 0.51 (0.18, 1.43) |  |  | 189 | 1.28 (0.36, 4.49) |

Outcome: Low Birthweight (Preterms only)

|  | **Across Pregnancy** | | **Trimester 1** | | **Trimester 2** | | **Trimester 3** | |
| --- | --- | --- | --- | --- | --- | --- | --- | --- |
| **Phenol Name** | **N** | **OR (95% CI)** | **N** | **OR (95% CI)** | **N** | **OR (95% CI)** | **N** | **OR (95% CI)** |
| Sum Bisphenols | 43 | 0.43 (0.07, 2.71) |  |  | 39 | 0.4 (0.04, 4.08) | 13 | 0.53 (0.01, 42.21) |
| Bisphenol A | 52 | 0.42 (0.1, 1.68) |  |  | 44 | 0.13 (0.01, 1.38) | 17 | 2.21 (0.1, 48.55) |
| Bisphenol F | 45 | 0.7 (0.2, 2.48) |  |  | 40 | 0.61 (0.11, 3.4) |  |  |
| Bisphenol S | 52 | 0.54 (0.13, 2.28) |  |  | 43 | 0.73 (0.15, 3.62) | 17 | 0.04 (0, 5.37) |
| 2,4-Dichlorophenol | 35 | 0.02 (0, 7.73) |  |  | 30 | 0 (0, 10.08) |  |  |
| 2,5-Dichlorophenol | 35 | 0.02 (0, 3.24) |  |  | 30 | 0.01 (0, 3.57) |  |  |
| Benzophenone-3 | 38 | 1.81 (0.32, 10.23) |  |  | 33 | 2.01 (0.32, 12.62) |  |  |
| Triclosan | 44 | 0.38 (0.06, 2.35) |  |  | 35 | 0.88 (0.16, 4.72) |  |  |
| Butyl paraben | 39 | 2.93 (0.46, 18.91) |  |  | 31 | 1.45 (0.23, 8.98) |  |  |
| Ethyl paraben | 43 | 1.52 (0.32, 7.23) |  |  | 33 | 1.68 (0.36, 7.82) |  |  |
| Methyl Paraben | 43 | 2.66 (0.22, 32.67) |  |  | 33 | 1.86 (0.26, 13.54) |  |  |
| Propyl paraben | 43 | 1.33 (0.27, 6.46) |  |  | 33 | 1.05 (0.25, 4.41) |  |  |

Outcome: Low Birthweight (Terms only)

|  | **Across Pregnancy** | | **Trimester 1** | | **Trimester 2** | | **Trimester 3** | |
| --- | --- | --- | --- | --- | --- | --- | --- | --- |
| **Phenol Name** | **N** | **OR (95% CI)** | **N** | **OR (95% CI)** | **N** | **OR (95% CI)** | **N** | **OR (95% CI)** |
| Sum Bisphenols | 651 | 1.96 (0.16, 23.44) | 275 | 2.73 (0.36, 20.8) | 595 | 0.34 (0.04, 2.68) | 276 | 5.83 (0.86, 39.69) |
| Bisphenol A | 929 | 0.66 (0.18, 2.45) | 298 | 1.08 (0.18, 6.41) |  |  |  |  |
| Bisphenol F |  |  |  |  |  |  | 276 | 1.99 (0.3, 13.16) |
| Bisphenol S | 906 | 1.25 (0.36, 4.38) |  |  | 710 | 0.44 (0.1, 1.87) |  |  |
| 2,4-Dichlorophenol | 586 | 0.36 (0.04, 3.12) |  |  | 422 | 0.25 (0, 14.73) | 156 | 1.76 (0.09, 33.62) |
| 2,5-Dichlorophenol | 586 | 0.64 (0.12, 3.36) | 34 | 0.28 (0, 137.44) | 424 | 14.19 (0.99, 202.64) | 156 | 0.27 (0.01, 5.47) |
| Benzophenone-3 |  |  | 77 | 0.44 (0.01, 16.16) | 496 | 0.39 (0.08, 1.97) | 173 | 0.85 (0.2, 3.54) |
| Triclosan | 745 | 1.37 (0.56, 3.34) |  |  | 512 | 0.17 (0.01, 2.83) | 185 | 2.75 (0.47, 16.16) |
| Butyl paraben |  |  |  |  | 450 | 0.19 (0.01, 4.66) | 179 | 1.09 (0.32, 3.76) |
| Ethyl paraben | 706 | 0.58 (0.23, 1.44) | 98 | 0.56 (0.09, 3.34) | 458 | 0.32 (0.07, 1.46) | 189 | 1.57 (0.34, 7.22) |
| Methyl Paraben | 707 | 2.14 (0.8, 5.73) | 100 | 1.01 (0.16, 6.22) | 458 | 2.04 (0.34, 12.19) | 189 | 2.61 (0.49, 14.05) |
| Propyl paraben | 698 | 1.05 (0.5, 2.22) | 98 | 0.31 (0.05, 1.95) |  |  | 185 | 1.28 (0.37, 4.48) |

**Supplement Table 2a2: Race/Ethnicity-Stratified Adjusted Associations of Phenol Exposures with Birth Outcomes (Continuously Measured),
Non-Hispanic Black Only**Models adjusted for maternal parity, and education, and child sex. BW for GA model removes parity from covariates. Blank cells reflect model non-convergence.

Outcome: Gestational Age

|  | **Across Pregnancy** | | **Trimester 1** | | **Trimester 2** | | **Trimester 3** | |
| --- | --- | --- | --- | --- | --- | --- | --- | --- |
| **Phenol Name** | **N** | **Beta (95% CI)** | **N** | **Beta (95% CI)** | **N** | **Beta (95% CI)** | **N** | **Beta (95% CI)** |
| Sum Bisphenols | 73 | -0.288 (-1.927, 1.352) | 33 | -0.177 (-2.999, 2.645) | 67 | -0.047 (-1.299, 1.206) | 32 | 0.535 (-1.279, 2.35) |
| Bisphenol A | 399 | 0.002 (-0.56, 0.563) | 153 | 0.442 (-0.505, 1.388) | 196 | -0.054 (-0.756, 0.647) | 240 | 0.006 (-0.586, 0.598) |
| Bisphenol F | 114 | 0.048 (-0.596, 0.693) | 58 | -0.238 (-1.144, 0.667) | 82 | 0.02 (-0.878, 0.918) | 37 | 0.17 (-0.989, 1.329) |
| Bisphenol S | 123 | 0.113 (-0.703, 0.929) | 33 | -0.052 (-1.271, 1.168) | 79 | 0.618 (-0.325, 1.56) | 70 | 0.278 (-0.465, 1.022) |
| 2,4-Dichlorophenol | 203 | -0.13 (-0.572, 0.312) |  |  | 32 | -0.222 (-1.34, 0.896) | 171 | -0.215 (-0.709, 0.279) |
| 2,5-Dichlorophenol | 202 | -0.048 (-0.347, 0.251) |  |  | 32 | 0.358 (-0.344, 1.061) | 170 | -0.139 (-0.487, 0.208) |
| Benzophenone-3 | 219 | -0.132 (-0.496, 0.233) | 11 | -1.188 (-3.922, 1.547) | 50 | -0.447 (-1.267, 0.373) | 170 | -0.076 (-0.5, 0.348) |
| Triclosan | 238 | -0.051 (-0.389, 0.288) | 24 | -0.939 (-2.662, 0.783) | 54 | **-0.613 (-1.092, -0.135)** | 171 | 0.207 (-0.215, 0.628) |
| Butyl paraben | 250 | 0.131 (-0.191, 0.452) | 33 | 0.238 (-0.279, 0.756) | 53 | 0.371 (-0.305, 1.046) | 171 | 0.102 (-0.318, 0.522) |
| Ethyl paraben | 135 | 0.088 (-0.241, 0.416) | 37 | -0.183 (-0.787, 0.421) | 56 | 0.238 (-0.279, 0.755) | 49 | -0.222 (-0.703, 0.259) |
| Methyl Paraben | 264 | -0.046 (-0.399, 0.308) | 37 | -0.268 (-0.91, 0.374) | 58 | -0.152 (-0.737, 0.433) | 176 | -0.093 (-0.564, 0.378) |
| Propyl paraben | 264 | 0.109 (-0.196, 0.414) | 37 | 0.188 (-0.317, 0.693) | 58 | -0.119 (-0.611, 0.374) | 176 | 0.078 (-0.327, 0.483) |

Outcome: Birth Length

|  | **Across Pregnancy** | | **Trimester 1** | | **Trimester 2** | | **Trimester 3** | |
| --- | --- | --- | --- | --- | --- | --- | --- | --- |
| **Phenol Name** | **N** | **Beta (95% CI)** | **N** | **Beta (95% CI)** | **N** | **Beta (95% CI)** | **N** | **Beta (95% CI)** |
| Sum Bisphenols | 70 | -0.859 (-3.237, 1.52) | 31 | -2.209 (-6.056, 1.638) | 64 | -0.325 (-2.185, 1.536) | 29 | 0.226 (-2.718, 3.17) |
| Bisphenol A | 309 | 0.023 (-0.883, 0.929) | 77 | **-1.755 (-3.419, -0.092)** | 135 | -0.728 (-1.755, 0.3) | 210 | 0.712 (-0.341, 1.766) |
| Bisphenol F | 106 | -0.877 (-1.842, 0.088) | 55 | **-1.506 (-2.802, -0.21)** | 78 | -0.801 (-2.077, 0.476) | 31 | 0.91 (-1.231, 3.05) |
| Bisphenol S | 120 | 0.156 (-1.181, 1.493) | 31 | -0.077 (-1.809, 1.656) | 76 | 0.965 (-0.466, 2.396) | 67 | -1.278 (-2.629, 0.074) |
| 2,4-Dichlorophenol | 201 | **0.824 (0.127, 1.521)** |  |  | 32 | -0.256 (-2.468, 1.955) | 169 | **0.888 (0.125, 1.652)** |
| 2,5-Dichlorophenol | 200 | **0.599 (0.137, 1.062)** |  |  | 32 | 0.156 (-1.254, 1.565) | 168 | **0.686 (0.161, 1.212)** |
| Benzophenone-3 | 217 | **-0.697 (-1.292, -0.102)** | 11 | -2.881 (-6.63, 0.867) | 50 | -0.942 (-2.504, 0.619) | 168 | -0.64 (-1.299, 0.02) |
| Triclosan | 217 | -0.016 (-0.598, 0.566) | 11 | -0.573 (-5.705, 4.56) | 48 | **-1.486 (-2.478, -0.493)** | 169 | 0.574 (-0.086, 1.234) |
| Butyl paraben | 232 | 0.065 (-0.493, 0.623) | 23 | -0.351 (-1.55, 0.848) | 50 | 0.014 (-1.051, 1.078) | 166 | 0.306 (-0.368, 0.98) |
| Ethyl paraben | 111 | -0.199 (-0.844, 0.446) | 23 | -0.496 (-1.778, 0.786) | 49 | 0.089 (-0.715, 0.893) | 46 | -0.235 (-1.381, 0.911) |
| Methyl Paraben | 238 | -0.49 (-1.086, 0.106) | 23 | **-1.353 (-2.605, -0.101)** | 51 | -0.686 (-1.555, 0.183) | 171 | -0.109 (-0.861, 0.643) |
| Propyl paraben | 238 | -0.488 (-1.005, 0.029) | 23 | -1.012 (-2.024, 0.001) | 51 | -0.165 (-0.91, 0.581) | 171 | -0.275 (-0.919, 0.368) |

Outcome: Birthweight

|  | **Across Pregnancy** | | **Trimester 1** | | **Trimester 2** | | **Trimester 3** | |
| --- | --- | --- | --- | --- | --- | --- | --- | --- |
| **Phenol Name** | **N** | **Beta (95% CI)** | **N** | **Beta (95% CI)** | **N** | **Beta (95% CI)** | **N** | **Beta (95% CI)** |
| Sum Bisphenols | 73 | -152.256 (-592.517, 288.006) | 33 | 116.373 (-670.598, 903.343) | 67 | 5.349 (-331.671, 342.369) | 32 | 421.122 (-138.678, 980.922) |
| Bisphenol A | 399 | -19.896 (-168.339, 128.547) | 153 | -91.862 (-335.424, 151.7) | 196 | -135.653 (-309.674, 38.368) | 240 | 104.78 (-65.373, 274.933) |
| Bisphenol F | 114 | -121.742 (-296.844, 53.361) | 58 | -134.206 (-394.261, 125.849) | 82 | -57.536 (-294.305, 179.234) | 37 | 252.345 (-99.985, 604.674) |
| Bisphenol S | 123 | 63.912 (-172.852, 300.676) | 33 | 124.002 (-213.584, 461.588) | 79 | **263.226 (0.069, 526.384)** | 70 | 10.37 (-226.966, 247.705) |
| 2,4-Dichlorophenol | 203 | 123.454 (-1.006, 247.913) |  |  | 32 | -13.286 (-380.254, 353.682) | 171 | 115.482 (-20.794, 251.757) |
| 2,5-Dichlorophenol | 202 | **93.566 (10.439, 176.694)** |  |  | 32 | -17.205 (-251.06, 216.651) | 170 | **97.74 (3.286, 192.194)** |
| Benzophenone-3 | 219 | **-114.886 (-219.106, -10.666)** | 11 | -594.763 (-1280.158, 90.632) | 50 | -174.234 (-440.399, 91.93) | 170 | **-119.339 (-236.112, -2.566)** |
| Triclosan | 238 | -6.451 (-104.68, 91.778) | 24 | -146.083 (-704.588, 412.422) | 54 | **-171.02 (-329.666, -12.374)** | 171 | 74.225 (-42.57, 191.019) |
| Butyl paraben | 250 | -12.917 (-104.721, 78.886) | 33 | -108.512 (-333.713, 116.689) | 53 | 64.74 (-125.964, 255.444) | 171 | 9.306 (-108.081, 126.692) |
| Ethyl paraben | 135 | -43.969 (-146.503, 58.564) | 37 | -120.244 (-346.936, 106.448) | 56 | 72.727 (-78.397, 223.851) | 49 | -153.24 (-310.361, 3.881) |
| Methyl Paraben | 264 | -77.864 (-178.645, 22.918) | 37 | -195.281 (-432.359, 41.797) | 58 | -91.601 (-260.445, 77.243) | 176 | -51.742 (-183.212, 79.728) |
| Propyl paraben | 264 | -46.725 (-133.782, 40.331) | 37 | -63.141 (-254.997, 128.716) | 58 | 28.851 (-114.557, 172.259) | 176 | -59.924 (-172.704, 52.856) |

Outcome: Birthweight for Gestational Age Z-Score

|  | **Across Pregnancy** | | **Trimester 1** | | **Trimester 2** | | **Trimester 3** | |
| --- | --- | --- | --- | --- | --- | --- | --- | --- |
| **Phenol Name** | **N** | **Beta (95% CI)** | **N** | **Beta (95% CI)** | **N** | **Beta (95% CI)** | **N** | **Beta (95% CI)** |
| Sum Bisphenols | 73 | -0.268 (-1.08, 0.544) | 33 | -0.306 (-1.561, 0.949) | 67 | 0.057 (-0.582, 0.696) | 32 | 0.936 (-0.116, 1.988) |
| Bisphenol A | 395 | -0.072 (-0.385, 0.241) | 152 | **-0.496 (-0.941, -0.05)** | 195 | **-0.354 (-0.679, -0.029)** | 237 | 0.248 (-0.152, 0.647) |
| Bisphenol F | 114 | -0.338 (-0.679, 0.004) | 58 | -0.267 (-0.764, 0.231) | 82 | -0.259 (-0.697, 0.18) | 37 | 0.383 (-0.309, 1.074) |
| Bisphenol S | 123 | -0.008 (-0.466, 0.45) | 33 | 0.279 (-0.284, 0.842) | 79 | 0.393 (-0.103, 0.89) | 70 | -0.207 (-0.67, 0.256) |
| 2,4-Dichlorophenol | 200 | **0.47 (0.166, 0.774)** |  |  | 32 | 0.186 (-0.685, 1.058) | 168 | **0.505 (0.168, 0.841)** |
| 2,5-Dichlorophenol | 199 | **0.311 (0.108, 0.514)** |  |  | 32 | -0.039 (-0.552, 0.473) | 167 | **0.383 (0.151, 0.615)** |
| Benzophenone-3 | 216 | -0.225 (-0.466, 0.016) | 11 | **-0.881 (-1.689, -0.073)** | 50 | -0.139 (-0.602, 0.324) | 167 | -0.271 (-0.564, 0.022) |
| Triclosan | 235 | 0.04 (-0.186, 0.267) | 24 | 0.3 (-0.516, 1.116) | 54 | -0.15 (-0.464, 0.164) | 168 | 0.124 (-0.171, 0.418) |
| Butyl paraben | 247 | -0.111 (-0.331, 0.11) | 33 | -0.529 (-1.068, 0.01) | 53 | -0.035 (-0.42, 0.351) | 168 | -0.014 (-0.307, 0.28) |
| Ethyl paraben | 135 | -0.149 (-0.373, 0.075) | 37 | -0.212 (-0.722, 0.299) | 56 | 0.054 (-0.237, 0.346) | 49 | -0.307 (-0.663, 0.049) |
| Methyl Paraben | 261 | -0.178 (-0.417, 0.061) | 37 | -0.311 (-0.843, 0.221) | 58 | -0.099 (-0.434, 0.236) | 173 | -0.093 (-0.423, 0.237) |
| Propyl paraben | 261 | -0.179 (-0.384, 0.026) | 37 | -0.3 (-0.727, 0.127) | 58 | 0.175 (-0.103, 0.452) | 173 | -0.186 (-0.468, 0.097) |

**Supplement Table 2b2: Race/Ethnicity -Stratified Adjusted Associations of Phenol Exposures with Categorical Birth Outcomes, Non-Hispanic Black Only**Models adjusted for maternal parity, and education, and child sex. Blank cells reflect model non-convergence.

Outcome: Preterm

|  | **Across Pregnancy** | | **Trimester 1** | | **Trimester 2** | | **Trimester 3** | |
| --- | --- | --- | --- | --- | --- | --- | --- | --- |
| **Phenol Name** | **N** | **OR (95% CI)** | **N** | **OR (95% CI)** | **N** | **OR (95% CI)** | **N** | **OR (95% CI)** |
| Sum Bisphenols | 73 | 0.37 (0.02, 5.86) | 33 | 0.17 (0, 49.33) | 67 | 1 (0.17, 6.05) | 32 | 0.08 (0, 5.74) |
| Bisphenol A | 399 | 0.94 (0.41, 2.17) | 153 | 0.41 (0.11, 1.49) | 196 | 0.95 (0.37, 2.46) | 240 | 0.9 (0.32, 2.54) |
| Bisphenol F | 114 | 0.55 (0.17, 1.75) | 58 | 0.7 (0.16, 3.06) | 82 | 0.91 (0.23, 3.63) | 37 | 0.14 (0, 7.16) |
| Bisphenol S | 123 | 0.56 (0.1, 2.94) | 33 | 2.81 (0.21, 36.72) | 79 | 0.66 (0.14, 3.05) | 70 | 0.76 (0.15, 3.75) |
| 2,4-Dichlorophenol | 203 | 1.92 (0.86, 4.31) |  |  |  |  | 171 | 1.75 (0.75, 4.1) |
| 2,5-Dichlorophenol | 202 | 1.57 (0.83, 2.97) |  |  |  |  | 170 | 1.62 (0.8, 3.26) |
| Benzophenone-3 | 219 | 1.24 (0.64, 2.42) |  |  |  |  | 170 | 1.41 (0.69, 2.89) |
| Triclosan | 238 | 1.43 (0.77, 2.66) | 24 | 11.09 (0.17, 731.94) | 54 | 14.96 (0.06, 3477.93) | 171 | 1.01 (0.49, 2.09) |
| Butyl paraben | 250 | 0.89 (0.5, 1.58) | 33 | 0.01 (0, 62.29) | 53 | 0.77 (0.16, 3.65) | 171 | 1.04 (0.5, 2.15) |
| Ethyl paraben | 135 | 0.65 (0.3, 1.42) | 37 | 0.49 (0.1, 2.46) | 56 | 0.69 (0.2, 2.41) | 49 | 2082.31 (0.01, 291951028.98) |
| Methyl Paraben | 264 | 1.25 (0.65, 2.41) | 37 | 7.44 (0.26, 212.56) | 58 | 1.15 (0.29, 4.57) | 176 | 1.33 (0.55, 3.25) |
| Propyl paraben | 264 | 1.03 (0.6, 1.78) | 37 | 0.79 (0.23, 2.64) | 58 | 0.69 (0.2, 2.43) | 176 | 1.41 (0.67, 2.98) |

Outcome: Small for Gestational Age

|  | **Across Pregnancy** | | **Trimester 1** | | **Trimester 2** | | **Trimester 3** | |
| --- | --- | --- | --- | --- | --- | --- | --- | --- |
| **Phenol Name** | **N** | **OR (95% CI)** | **N** | **OR (95% CI)** | **N** | **OR (95% CI)** | **N** | **OR (95% CI)** |
| Sum Bisphenols | 73 | 1.39 (0.12, 15.68) | 33 | 0.34 (0.01, 14.69) | 67 | 1.43 (0.2, 10.35) | 32 | 0.86 (0.02, 39.92) |
| Bisphenol A | 395 | 1.17 (0.55, 2.48) | 152 | 1.28 (0.38, 4.38) | 195 | 1.77 (0.73, 4.29) | 237 | 1.11 (0.46, 2.66) |
| Bisphenol F | 114 | 2.19 (0.76, 6.32) | 58 | 2.31 (0.32, 16.53) | 82 | 2.45 (0.54, 11.01) | 37 | 0.25 (0.01, 4.3) |
| Bisphenol S | 123 | 2.09 (0.63, 6.89) | 33 | 1.41 (0.21, 9.39) | 79 | 0.82 (0.17, 4.08) | 70 | 1.92 (0.58, 6.38) |
| 2,4-Dichlorophenol | 200 | 0.59 (0.28, 1.27) |  |  | 32 | 0.07 (0, 198.25) | 168 | 0.53 (0.24, 1.19) |
| 2,5-Dichlorophenol | 199 | 0.67 (0.41, 1.1) |  |  | 32 | 5.7 (0.28, 115.32) | 167 | **0.59 (0.35, 0.99)** |
| Benzophenone-3 | 216 | **2.14 (1.23, 3.72)** |  |  | 50 | 2.24 (0.38, 13.09) | 167 | **2.41 (1.29, 4.49)** |
| Triclosan | 235 | 1.02 (0.59, 1.75) | 24 | 1 (0.05, 19.95) | 54 | 0.86 (0.29, 2.53) | 168 | 0.92 (0.48, 1.75) |
| Butyl paraben | 247 | 1.09 (0.67, 1.77) | 33 | 1.38 (0.46, 4.17) | 53 | 1.23 (0.27, 5.67) | 168 | 0.89 (0.47, 1.69) |
| Ethyl paraben | 135 | 1.25 (0.71, 2.22) | 37 | 0.83 (0.25, 2.71) | 56 | 0.02 (0, 1.09) | 49 | **3.17 (1.1, 9.16)** |
| Methyl Paraben | 261 | 1.37 (0.78, 2.42) | 37 | 2.08 (0.4, 10.93) | 58 | 4.69 (0.55, 39.89) | 173 | 1.08 (0.54, 2.16) |
| Propyl paraben | 261 | 1.34 (0.82, 2.17) | 37 | 1.26 (0.44, 3.58) | 58 | 2.38 (0.58, 9.77) | 173 | 1.13 (0.62, 2.05) |

Outcome: Large for Gestational Age

|  | **Across Pregnancy** | | **Trimester 1** | | **Trimester 2** | | **Trimester 3** | |
| --- | --- | --- | --- | --- | --- | --- | --- | --- |
| **Phenol Name** | **N** | **OR (95% CI)** | **N** | **OR (95% CI)** | **N** | **OR (95% CI)** | **N** | **OR (95% CI)** |
| Sum Bisphenols | 73 | 0.39 (0.02, 9.72) | 33 | 9.18 (0.02, 5289.66) | 67 | 0.23 (0.01, 4.54) | 32 | 0.37 (0, 96.93) |
| Bisphenol A | 395 | 0.77 (0.28, 2.12) | 152 | 0.3 (0.06, 1.48) | 195 | 0.26 (0.07, 1.05) | 237 | 2.74 (0.83, 9) |
| Bisphenol F | 114 | 0.85 (0.24, 3.07) | 58 | 0.51 (0.07, 3.5) | 82 | 1.02 (0.17, 6.33) | 37 | 1.27 (0.07, 24.26) |
| Bisphenol S | 123 | 1.31 (0.18, 9.5) | 33 | 1.46 (0.11, 18.62) | 79 | 1.09 (0.19, 6.18) | 70 | 2.14 (0.18, 25.82) |
| 2,4-Dichlorophenol | 200 | 1.59 (0.7, 3.62) |  |  | 32 | 0 (0, 2304.16) | 168 | 1.6 (0.67, 3.79) |
| 2,5-Dichlorophenol | 199 | 1.37 (0.75, 2.5) |  |  | 32 | 0.08 (0, 24.56) | 167 | 1.47 (0.74, 2.9) |
| Benzophenone-3 | 216 | 0.61 (0.29, 1.29) |  |  | 50 | 0.39 (0.05, 3.18) | 167 | 0.66 (0.28, 1.59) |
| Triclosan | 235 | 1.53 (0.82, 2.84) | 24 | 5.8 (0.15, 218.38) | 54 | 0.77 (0.2, 2.97) | 168 | **2.1 (1.01, 4.38)** |
| Butyl paraben | 247 | 0.97 (0.5, 1.9) | 33 | 0.97 (0.26, 3.69) | 53 | 0.48 (0.03, 6.99) | 168 | 1.3 (0.59, 2.85) |
| Ethyl paraben | 135 | 0.6 (0.16, 2.21) | 37 | 0 (0, 49.35) | 56 | 2.97 (0.38, 23.53) | 49 | 0.84 (0.02, 30.26) |
| Methyl Paraben | 261 | 0.74 (0.36, 1.52) | 37 | 0.58 (0.08, 4.06) | 58 | 0.88 (0.16, 4.92) | 173 | 0.72 (0.3, 1.74) |
| Propyl paraben | 261 | 0.94 (0.5, 1.75) | 37 | 0.9 (0.23, 3.64) | 58 | 1.72 (0.38, 7.9) | 173 | 0.8 (0.36, 1.75) |

Outcome: Low Birthweight

|  | **Across Pregnancy** | | **Trimester 1** | | **Trimester 2** | | **Trimester 3** | |
| --- | --- | --- | --- | --- | --- | --- | --- | --- |
| **Phenol Name** | **N** | **OR (95% CI)** | **N** | **OR (95% CI)** | **N** | **OR (95% CI)** | **N** | **OR (95% CI)** |
| Sum Bisphenols | 73 | 1.27 (0.13, 12.63) | 33 | 1.32 (0.03, 62.19) | 67 | 2.17 (0.44, 10.67) | 32 | 0.7 (0.03, 15.6) |
| Bisphenol A | 399 | 1.01 (0.39, 2.62) | 153 | 0.84 (0.22, 3.25) | 196 | 2.4 (0.91, 6.29) | 240 | 1 (0.31, 3.26) |
| Bisphenol F | 114 | 1.16 (0.39, 3.42) | 58 | 2.31 (0.35, 15.37) | 82 | 1.76 (0.55, 5.62) | 37 | 0.26 (0.02, 4.22) |
| Bisphenol S | 123 | 1.68 (0.43, 6.52) | 33 | 0.71 (0.1, 5.15) | 79 | 0.77 (0.18, 3.17) | 70 | 2.48 (0.65, 9.47) |
| 2,4-Dichlorophenol | 203 | 0.53 (0.17, 1.62) |  |  | 32 | 59.36 (0.05, 68760.89) | 171 | 0.44 (0.13, 1.49) |
| 2,5-Dichlorophenol | 202 | 0.5 (0.24, 1.05) |  |  | 32 | 2.87 (0.13, 65.41) | 170 | 0.45 (0.2, 1.01) |
| Benzophenone-3 | 219 | 0.87 (0.37, 2.04) |  |  | 50 | 1.09 (0.16, 7.51) | 170 | 0.82 (0.29, 2.28) |
| Triclosan | 238 | 1.13 (0.54, 2.36) | 24 | 6.64 (0.18, 249.71) | 54 | 2.36 (0.6, 9.3) | 171 | 0.74 (0.28, 1.96) |
| Butyl paraben | 250 | 0.95 (0.49, 1.85) | 33 | 7.52 (0.39, 145.31) | 53 | 0.64 (0.11, 3.61) | 171 | 0.62 (0.2, 1.91) |
| Ethyl paraben | 135 | 1.57 (0.82, 3.03) | 37 | 1.17 (0.3, 4.57) | 56 | 0.63 (0.16, 2.52) | 49 | 182.82 (0.51, 65152.47) |
| Methyl Paraben | 264 | 1.55 (0.71, 3.39) | 37 | 9.8 (0.43, 225.28) | 58 | 1.14 (0.29, 4.58) | 176 | 1.13 (0.38, 3.37) |
| Propyl paraben | 264 | 1.31 (0.69, 2.5) | 37 | 2.32 (0.57, 9.38) | 58 | 0.79 (0.25, 2.54) | 176 | 1.14 (0.45, 2.87) |

Outcome: Low Birthweight (Preterms only)

|  | **Across Pregnancy** | | **Trimester 1** | | **Trimester 2** | | **Trimester 3** | |
| --- | --- | --- | --- | --- | --- | --- | --- | --- |
| **Phenol Name** | **N** | **OR (95% CI)** | **N** | **OR (95% CI)** | **N** | **OR (95% CI)** | **N** | **OR (95% CI)** |
| Sum Bisphenols |  |  |  |  |  |  |  |  |
| Bisphenol A | 49 | 0.55 (0.11, 2.6) | 21 | 0.8 (0.04, 16.82) | 25 | 12.62 (0.01, 16439.15) | 29 | 0.85 (0.07, 10.11) |
| Bisphenol F |  |  |  |  |  |  |  |  |
| Bisphenol S |  |  |  |  |  |  |  |  |
| 2,4-Dichlorophenol | 19 | 0.6 (0.06, 6.17) |  |  |  |  | 18 | 0.54 (0.02, 11.81) |
| 2,5-Dichlorophenol | 19 | 0.16 (0.02, 1.59) |  |  |  |  | 18 | 0.19 (0.01, 5.19) |
| Benzophenone-3 | 21 | 0.38 (0.02, 6.18) |  |  |  |  | 18 | 0.43 (0, 36.57) |
| Triclosan | 22 | 1.59 (0.29, 8.78) |  |  |  |  | 18 | 2.13 (0.09, 48.7) |
| Butyl paraben | 28 | 0.01 (0, 1.82) |  |  |  |  |  |  |
| Ethyl paraben |  |  |  |  |  |  |  |  |
| Methyl Paraben | 29 | 0.85 (0.2, 3.54) |  |  |  |  | 18 | 0.25 (0.01, 4.59) |
| Propyl paraben | 29 | 0.4 (0.1, 1.51) |  |  |  |  | 18 | 0.26 (0.02, 3.01) |

Outcome: Low Birthweight (Terms only)

|  | **Across Pregnancy** | | **Trimester 1** | | **Trimester 2** | | **Trimester 3** | |
| --- | --- | --- | --- | --- | --- | --- | --- | --- |
| **Phenol Name** | **N** | **OR (95% CI)** | **N** | **OR (95% CI)** | **N** | **OR (95% CI)** | **N** | **OR (95% CI)** |
| Sum Bisphenols | 64 | 2.01 (0.04, 109.02) | 29 | 0.26 (0, 28.94) | 58 | 44.66 (0.39, 5087.51) | 27 | 0.27 (0, 28.35) |
| Bisphenol A | 350 | 2.64 (0.53, 13.22) | 132 | 5.33 (0.38, 75.12) | 171 | **9.84 (1.86, 52.23)** | 211 | 1.22 (0.23, 6.51) |
| Bisphenol F | 96 | 2.47 (0.55, 11.09) | 48 | 11.68 (0.47, 289.37) | 70 | 6.19 (0.6, 63.64) | 32 | 0.19 (0.01, 4.75) |
| Bisphenol S | 112 | 3.11 (0.51, 19.09) | 29 | 0.56 (0.04, 7.32) | 70 | 0.99 (0.09, 10.87) | 63 | 1.79 (0.3, 10.77) |
| 2,4-Dichlorophenol | 184 | **0.15 (0.02, 1)** |  |  |  |  | 153 | **0.12 (0.02, 0.92)** |
| 2,5-Dichlorophenol | 183 | **0.27 (0.08, 0.9)** |  |  |  |  | 152 | **0.15 (0.03, 0.7)** |
| Benzophenone-3 | 198 | 0.73 (0.19, 2.86) |  |  |  |  | 152 | 0.83 (0.19, 3.65) |
| Triclosan | 216 | 0.42 (0.11, 1.58) |  |  |  |  | 153 | 0.44 (0.1, 2.04) |
| Butyl paraben | 222 | 1.64 (0.75, 3.59) | 29 | 6.57 (0.42, 102.3) |  |  | 153 | 1.13 (0.33, 3.92) |
| Ethyl paraben | 122 | 2.38 (0.83, 6.82) | 32 | 2.27 (0.26, 19.84) |  |  |  |  |
| Methyl Paraben | 235 | 2.06 (0.61, 6.98) |  |  |  |  | 158 | 1.59 (0.36, 6.95) |
| Propyl paraben | 235 | **3.19 (1.09, 9.37)** |  |  |  |  | 158 | 1.62 (0.43, 6.14) |

**Supplement Table 2a3: Race/Ethnicity-Stratified Adjusted Associations of Phenol Exposures with Birth Outcomes (Continuously Measured), Hispanic Only**Models adjusted for maternal parity, and education, and child sex. BW for GA model removes parity from covariates. Blank cells reflect model non-convergence.

Outcome: Gestational Age

|  | **Across Pregnancy** | | **Trimester 1** | | **Trimester 2** | | **Trimester 3** | |
| --- | --- | --- | --- | --- | --- | --- | --- | --- |
| **Phenol Name** | **N** | **Beta (95% CI)** | **N** | **Beta (95% CI)** | **N** | **Beta (95% CI)** | **N** | **Beta (95% CI)** |
| Sum Bisphenols | 691 | 0.02 (-0.372, 0.413) | 277 | -0.197 (-0.717, 0.322) | 661 | 0.129 (-0.236, 0.494) | 310 | -0.212 (-0.609, 0.185) |
| Bisphenol A | 1114 | 0.088 (-0.144, 0.319) | 313 | -0.091 (-0.511, 0.33) | 778 | 0.069 (-0.189, 0.326) | 621 | -0.047 (-0.282, 0.188) |
| Bisphenol F | 692 | 0.062 (-0.215, 0.339) | 278 | -0.263 (-0.711, 0.186) | 661 | **0.354 (0.064, 0.645)** | 310 | -0.045 (-0.372, 0.283) |
| Bisphenol S | 806 | -0.139 (-0.425, 0.146) | 277 | -0.211 (-0.581, 0.158) | 704 | -0.084 (-0.353, 0.185) | 384 | -0.224 (-0.486, 0.038) |
| 2,4-Dichlorophenol | 660 | **0.233 (0.027, 0.438)** |  |  | 334 | -0.055 (-0.4, 0.29) | 412 | 0.2 (-0.038, 0.438) |
| 2,5-Dichlorophenol | 660 | **0.206 (0.065, 0.347)** |  |  | 334 | -0.04 (-0.264, 0.184) | 412 | **0.175 (0.006, 0.345)** |
| Benzophenone-3 | 813 | 0.028 (-0.119, 0.174) | 84 | 0.593 (-0.023, 1.21) | 484 | 0.094 (-0.095, 0.283) | 416 | -0.001 (-0.188, 0.187) |
| Triclosan | 825 | -0.02 (-0.153, 0.113) | 93 | 0.267 (-0.248, 0.782) | 485 | 0.042 (-0.123, 0.207) | 417 | -0.127 (-0.3, 0.047) |
| Butyl paraben | 673 | 0.122 (-0.029, 0.273) | 13 | 0.445 (-1.527, 2.417) | 347 | 0.185 (-0.008, 0.378) | 406 | -0.002 (-0.194, 0.189) |
| Ethyl paraben | 453 | -0.099 (-0.303, 0.105) | 17 | -0.554 (-2.251, 1.143) | 344 | -0.14 (-0.381, 0.101) | 184 | 0.078 (-0.203, 0.358) |
| Methyl Paraben | 689 | **0.226 (0.031, 0.421)** | 17 | -0.162 (-2.067, 1.743) | 347 | 0.243 (-0.021, 0.506) | 418 | 0.033 (-0.206, 0.271) |
| Propyl paraben | 689 | 0.139 (-0.011, 0.29) | 17 | 0.277 (-1.149, 1.703) | 347 | 0.079 (-0.127, 0.285) | 418 | 0.066 (-0.118, 0.25) |

Outcome: Birth Length

|  | **Across Pregnancy** | | **Trimester 1** | | **Trimester 2** | | **Trimester 3** | |
| --- | --- | --- | --- | --- | --- | --- | --- | --- |
| **Phenol Name** | **N** | **Beta (95% CI)** | **N** | **Beta (95% CI)** | **N** | **Beta (95% CI)** | **N** | **Beta (95% CI)** |
| Sum Bisphenols | 670 | 0.508 (-0.158, 1.173) | 270 | 0.226 (-0.572, 1.023) | 640 | 0.347 (-0.278, 0.972) | 303 | 0.136 (-0.548, 0.82) |
| Bisphenol A | 1065 | 0.287 (-0.107, 0.68) | 305 | 0.195 (-0.429, 0.82) | 754 | 0.115 (-0.314, 0.544) | 588 | 0.25 (-0.168, 0.669) |
| Bisphenol F | 671 | 0.126 (-0.343, 0.594) | 271 | -0.245 (-0.931, 0.442) | 640 | 0.262 (-0.238, 0.763) | 303 | 0.019 (-0.546, 0.584) |
| Bisphenol S | 782 | **0.55 (0.056, 1.044)** | 270 | 0.211 (-0.351, 0.773) | 682 | 0.421 (-0.044, 0.886) | 375 | -0.061 (-0.521, 0.4) |
| 2,4-Dichlorophenol | 622 | 0.301 (-0.059, 0.66) |  |  | 321 | 0.279 (-0.298, 0.855) | 386 | 0.204 (-0.24, 0.648) |
| 2,5-Dichlorophenol | 622 | **0.304 (0.058, 0.55)** |  |  | 321 | 0.079 (-0.295, 0.453) | 386 | 0.29 (-0.023, 0.604) |
| Benzophenone-3 | 774 | -0.186 (-0.446, 0.075) | 84 | 0.331 (-0.485, 1.147) | 470 | -0.025 (-0.359, 0.309) | 390 | **-0.377 (-0.72, -0.033)** |
| Triclosan | 765 | 0.113 (-0.118, 0.345) | 82 | 0.15 (-0.59, 0.889) | 464 | 0.077 (-0.202, 0.357) | 388 | 0.192 (-0.127, 0.512) |
| Butyl paraben | 619 | 0.084 (-0.196, 0.365) |  |  | 327 | 0.04 (-0.313, 0.393) | 380 | 0.06 (-0.298, 0.418) |
| Ethyl paraben | 417 | 0.203 (-0.166, 0.572) |  |  | 324 | 0.393 (-0.05, 0.836) | 178 | -0.161 (-0.665, 0.342) |
| Methyl Paraben | 630 | 0.096 (-0.263, 0.456) |  |  | 327 | 0.097 (-0.386, 0.579) | 389 | -0.13 (-0.575, 0.316) |
| Propyl paraben | 630 | 0.022 (-0.258, 0.301) |  |  | 327 | -0.032 (-0.412, 0.348) | 389 | -0.054 (-0.399, 0.291) |

Outcome: Birthweight

|  | **Across Pregnancy** | | **Trimester 1** | | **Trimester 2** | | **Trimester 3** | |
| --- | --- | --- | --- | --- | --- | --- | --- | --- |
| **Phenol Name** | **N** | **Beta (95% CI)** | **N** | **Beta (95% CI)** | **N** | **Beta (95% CI)** | **N** | **Beta (95% CI)** |
| Sum Bisphenols | 691 | 29.719 (-89.349, 148.787) | 277 | 51.87 (-96.82, 200.56) | 661 | 33.262 (-76.84, 143.364) | 310 | -129.471 (-265.485, 6.543) |
| Bisphenol A | 1114 | 18.177 (-49.679, 86.033) | 313 | 74.404 (-40.765, 189.572) | 778 | 5.7 (-70.174, 81.574) | 621 | -16.994 (-91.377, 57.389) |
| Bisphenol F | 692 | -5.893 (-89.36, 77.573) | 278 | -42.662 (-169.854, 84.531) | 661 | 37.356 (-50.504, 125.216) | 310 | -70.937 (-183.488, 41.615) |
| Bisphenol S | 806 | **97.878 (11.731, 184.026)** | 277 | 16.261 (-89.755, 122.278) | 704 | **92.121 (11.531, 172.712)** | 384 | -73.689 (-162.434, 15.056) |
| 2,4-Dichlorophenol | 660 | **63.794 (3.129, 124.459)** |  |  | 334 | 43.562 (-63.431, 150.556) | 412 | 52.783 (-19.127, 124.693) |
| 2,5-Dichlorophenol | 660 | **50.504 (8.758, 92.25)** |  |  | 334 | 2.082 (-67.525, 71.689) | 412 | **53.64 (2.495, 104.784)** |
| Benzophenone-3 | 813 | -23.562 (-65.877, 18.753) | 84 | 43.519 (-110.296, 197.334) | 484 | -18.243 (-74.36, 37.875) | 416 | -33.758 (-90.208, 22.692) |
| Triclosan | 825 | -3.69 (-42.248, 34.867) | 93 | -24.247 (-153.757, 105.263) | 485 | -1.118 (-50.224, 47.989) | 417 | -9.162 (-61.565, 43.241) |
| Butyl paraben | 673 | 3.136 (-41.927, 48.198) | 13 | -143.612 (-733.649, 446.426) | 347 | -5.373 (-65.85, 55.104) | 406 | -8.366 (-65.743, 49.01) |
| Ethyl paraben | 453 | -28.353 (-91.449, 34.743) | 17 | **-385.47 (-767.451, -3.488)** | 344 | 17.007 (-58.907, 92.921) | 184 | -60.804 (-154.407, 32.799) |
| Methyl Paraben | 689 | 11.425 (-46.727, 69.577) | 17 | -384.359 (-818.743, 50.025) | 347 | -2.854 (-85.611, 79.903) | 418 | -18.489 (-90.394, 53.415) |
| Propyl paraben | 689 | 24.689 (-20.147, 69.524) | 17 | -239.591 (-577.695, 98.513) | 347 | -5.351 (-69.699, 58.997) | 418 | 11.867 (-43.686, 67.42) |

Outcome: Birthweight for Gestational Age Z-Score

|  | **Across Pregnancy** | | **Trimester 1** | | **Trimester 2** | | **Trimester 3** | |
| --- | --- | --- | --- | --- | --- | --- | --- | --- |
| **Phenol Name** | **N** | **Beta (95% CI)** | **N** | **Beta (95% CI)** | **N** | **Beta (95% CI)** | **N** | **Beta (95% CI)** |
| Sum Bisphenols | 691 | 0.076 (-0.174, 0.327) | 277 | 0.204 (-0.087, 0.495) | 661 | 0.035 (-0.194, 0.265) | 310 | -0.193 (-0.475, 0.088) |
| Bisphenol A | 1111 | 0.022 (-0.124, 0.168) | 313 | 0.217 (-0.001, 0.435) | 778 | -0.007 (-0.163, 0.15) | 619 | 0.001 (-0.164, 0.166) |
| Bisphenol F | 692 | -0.029 (-0.204, 0.146) | 278 | 0.019 (-0.229, 0.268) | 661 | -0.069 (-0.253, 0.114) | 310 | -0.122 (-0.353, 0.109) |
| Bisphenol S | 806 | **0.279 (0.098, 0.461)** | 277 | 0.132 (-0.077, 0.34) | 704 | **0.248 (0.081, 0.416)** | 384 | -0.091 (-0.277, 0.094) |
| 2,4-Dichlorophenol | 657 | 0.08 (-0.06, 0.22) |  |  | 334 | 0.134 (-0.107, 0.374) | 410 | 0.07 (-0.096, 0.236) |
| 2,5-Dichlorophenol | 657 | 0.048 (-0.048, 0.144) |  |  | 334 | 0.004 (-0.152, 0.16) | 410 | 0.082 (-0.036, 0.2) |
| Benzophenone-3 | 810 | -0.087 (-0.182, 0.009) | 84 | -0.2 (-0.485, 0.085) | 484 | -0.085 (-0.208, 0.037) | 414 | -0.098 (-0.227, 0.032) |
| Triclosan | 822 | 0.003 (-0.083, 0.09) | 93 | -0.212 (-0.438, 0.015) | 485 | -0.018 (-0.125, 0.088) | 415 | 0.037 (-0.084, 0.158) |
| Butyl paraben | 670 | -0.055 (-0.159, 0.049) | 13 | -0.237 (-1.036, 0.563) | 347 | -0.123 (-0.257, 0.011) | 404 | 0.007 (-0.125, 0.139) |
| Ethyl paraben | 453 | -0.022 (-0.163, 0.12) | 17 | **-0.838 (-1.655, -0.022)** | 344 | 0.128 (-0.042, 0.298) | 184 | **-0.199 (-0.395, -0.004)** |
| Methyl Paraben | 686 | -0.067 (-0.2, 0.067) | 17 | -0.336 (-1.08, 0.407) | 347 | -0.091 (-0.278, 0.095) | 416 | -0.044 (-0.209, 0.121) |
| Propyl paraben | 686 | 0.005 (-0.098, 0.108) | 17 | -0.239 (-0.827, 0.349) | 347 | -0.025 (-0.169, 0.119) | 416 | 0.015 (-0.113, 0.142) |

**Supplement Table 2b3: Race/Ethnicity-Stratified Adjusted Associations of Phenol Exposures with Categorical Birth Outcomes, Hispanic Only**Models adjusted for maternal parity, and education, and child sex. Blank cells reflect model non-convergence.

Outcome: Preterm

|  | **Across Pregnancy** | | **Trimester 1** | | **Trimester 2** | | **Trimester 3** | |
| --- | --- | --- | --- | --- | --- | --- | --- | --- |
| **Phenol Name** | **N** | **OR (95% CI)** | **N** | **OR (95% CI)** | **N** | **OR (95% CI)** | **N** | **OR (95% CI)** |
| Sum Bisphenols | 691 | 1.5 (0.63, 3.59) | 277 | 1.4 (0.44, 4.45) | 661 | 1.3 (0.58, 2.94) | 310 | 1.91 (0.54, 6.75) |
| Bisphenol A | 1114 | 1.27 (0.71, 2.28) | 313 | 1.17 (0.48, 2.82) | 778 | 1.31 (0.73, 2.35) | 621 | 1.58 (0.72, 3.47) |
| Bisphenol F | 692 | 1.19 (0.66, 2.15) | 278 | 1.34 (0.56, 3.21) | 661 | 0.85 (0.43, 1.66) | 310 | 1.26 (0.45, 3.54) |
| Bisphenol S | 806 | 1.22 (0.6, 2.49) | 277 | 1.69 (0.69, 4.16) | 704 | 1.17 (0.62, 2.2) | 384 | 1.22 (0.47, 3.17) |
| 2,4-Dichlorophenol | 660 | 0.85 (0.48, 1.49) |  |  | 334 | 1.28 (0.6, 2.74) | 412 | 1.1 (0.54, 2.23) |
| 2,5-Dichlorophenol | 660 | 0.83 (0.57, 1.22) |  |  | 334 | 1.2 (0.72, 2.01) | 412 | 1.01 (0.61, 1.68) |
| Benzophenone-3 | 813 | 0.82 (0.56, 1.19) | 84 | 0.62 (0.21, 1.87) | 484 | 0.94 (0.6, 1.45) | 416 | 0.63 (0.34, 1.17) |
| Triclosan | 825 | 1.21 (0.88, 1.66) | 93 | 0.64 (0.26, 1.58) | 485 | 1.13 (0.78, 1.63) | 417 | **1.8 (1.1, 2.93)** |
| Butyl paraben | 673 | 0.91 (0.6, 1.38) |  |  | 347 | 0.71 (0.41, 1.23) | 406 | 1.3 (0.77, 2.2) |
| Ethyl paraben | 453 | 1.33 (0.82, 2.17) |  |  | 344 | 1.51 (0.85, 2.67) | 184 | 0.89 (0.38, 2.05) |
| Methyl Paraben | 689 | 0.73 (0.44, 1.22) |  |  | 347 | 0.74 (0.38, 1.42) | 418 | 0.91 (0.46, 1.8) |
| Propyl paraben | 689 | 0.86 (0.58, 1.28) |  |  | 347 | 1.02 (0.62, 1.68) | 418 | 0.87 (0.51, 1.48) |

Outcome: Small for Gestational Age

|  | **Across Pregnancy** | | **Trimester 1** | | **Trimester 2** | | **Trimester 3** | |
| --- | --- | --- | --- | --- | --- | --- | --- | --- |
| **Phenol Name** | **N** | **OR (95% CI)** | **N** | **OR (95% CI)** | **N** | **OR (95% CI)** | **N** | **OR (95% CI)** |
| Sum Bisphenols | 691 | 1.35 (0.67, 2.71) | 277 | 0.95 (0.38, 2.37) | 661 | 1.2 (0.62, 2.33) | 310 | 2.05 (0.95, 4.45) |
| Bisphenol A | 1111 | 1.26 (0.8, 1.99) | 313 | 0.68 (0.34, 1.35) | 778 | 1.27 (0.78, 2.07) | 619 | 1.2 (0.73, 1.96) |
| Bisphenol F | 692 | 1.25 (0.78, 2.02) | 278 | 1.33 (0.65, 2.72) | 661 | 1.19 (0.71, 2.01) | 310 | 1.35 (0.74, 2.47) |
| Bisphenol S | 806 | 0.67 (0.39, 1.16) | 277 | 0.84 (0.45, 1.59) | 704 | 0.66 (0.39, 1.09) | 384 | 1.44 (0.84, 2.5) |
| 2,4-Dichlorophenol | 657 | 0.83 (0.54, 1.28) |  |  | 334 | 0.77 (0.37, 1.6) | 410 | 0.82 (0.49, 1.35) |
| 2,5-Dichlorophenol | 657 | 0.91 (0.68, 1.22) |  |  | 334 | 0.96 (0.61, 1.51) | 410 | 0.87 (0.61, 1.24) |
| Benzophenone-3 | 810 | 1.19 (0.88, 1.59) | 84 | 2.05 (0.63, 6.61) | 484 | 1.1 (0.75, 1.62) | 414 | 1.29 (0.88, 1.9) |
| Triclosan | 822 | 1.24 (0.95, 1.62) | 93 | 0.96 (0.31, 2.96) | 485 | 1.24 (0.9, 1.72) | 415 | 1.28 (0.89, 1.84) |
| Butyl paraben | 670 | 0.93 (0.68, 1.28) | 13 | 10.98 (0.02, 7647.49) | 347 | 1.02 (0.7, 1.49) | 404 | 0.87 (0.58, 1.32) |
| Ethyl paraben | 453 | 0.96 (0.64, 1.43) | 17 | 482.43 (0.01, 41886788.73) | 344 | 0.65 (0.38, 1.11) | 184 | 1.34 (0.79, 2.26) |
| Methyl Paraben | 686 | 0.84 (0.57, 1.24) | 17 | 7.64 (0.1, 561.86) | 347 | 0.81 (0.49, 1.36) | 416 | 0.8 (0.49, 1.3) |
| Propyl paraben | 686 | 0.88 (0.65, 1.19) | 17 | 2.7 (0.25, 29.01) | 347 | 0.95 (0.64, 1.43) | 416 | 0.76 (0.52, 1.11) |

Outcome: Large for Gestational Age

|  | **Across Pregnancy** | | **Trimester 1** | | **Trimester 2** | | **Trimester 3** | |
| --- | --- | --- | --- | --- | --- | --- | --- | --- |
| **Phenol Name** | **N** | **OR (95% CI)** | **N** | **OR (95% CI)** | **N** | **OR (95% CI)** | **N** | **OR (95% CI)** |
| Sum Bisphenols | 691 | 1.96 (0.9, 4.26) | 277 | 1.63 (0.47, 5.63) | 661 | 1.57 (0.75, 3.3) | 310 | 0.73 (0.22, 2.43) |
| Bisphenol A | 1111 | 1.45 (0.89, 2.35) | 313 | 1.93 (0.74, 4.99) | 778 | 1.56 (0.92, 2.67) | 619 | 1.25 (0.69, 2.26) |
| Bisphenol F | 692 | 1.16 (0.67, 2.02) | 278 | 2.28 (0.92, 5.65) | 661 | 1.04 (0.58, 1.87) | 310 | 0.59 (0.19, 1.81) |
| Bisphenol S | 806 | **1.89 (1.02, 3.48)** | 277 | 0.47 (0.18, 1.22) | 704 | **1.96 (1.11, 3.45)** | 384 | 0.69 (0.3, 1.56) |
| 2,4-Dichlorophenol | 657 | 1.19 (0.78, 1.82) |  |  | 334 | **1.99 (1.01, 3.9)** | 410 | 0.89 (0.51, 1.56) |
| 2,5-Dichlorophenol | 657 | 1.18 (0.87, 1.59) |  |  | 334 | 1.54 (0.98, 2.41) | 410 | 0.98 (0.66, 1.44) |
| Benzophenone-3 | 810 | 0.93 (0.69, 1.26) | 84 | 0.59 (0.14, 2.55) | 484 | 1 (0.69, 1.45) | 414 | 0.82 (0.53, 1.26) |
| Triclosan | 822 | 1.29 (0.99, 1.67) | 93 | 0.69 (0.23, 2.06) | 485 | 1.19 (0.87, 1.63) | 415 | 1.44 (0.98, 2.1) |
| Butyl paraben | 670 | 0.87 (0.62, 1.23) |  |  | 347 | 0.79 (0.5, 1.23) | 404 | 0.88 (0.56, 1.39) |
| Ethyl paraben | 453 | 1.19 (0.76, 1.88) |  |  | 344 | 1.37 (0.83, 2.25) | 184 | 1.2 (0.56, 2.57) |
| Methyl Paraben | 686 | 0.84 (0.55, 1.28) |  |  | 347 | 0.82 (0.46, 1.47) | 416 | 0.77 (0.44, 1.34) |
| Propyl paraben | 686 | 1.04 (0.75, 1.43) |  |  | 347 | 0.97 (0.62, 1.5) | 416 | 0.83 (0.54, 1.27) |

Outcome: Low Birthweight

|  | **Across Pregnancy** | | **Trimester 1** | | **Trimester 2** | | **Trimester 3** | |
| --- | --- | --- | --- | --- | --- | --- | --- | --- |
| **Phenol Name** | **N** | **OR (95% CI)** | **N** | **OR (95% CI)** | **N** | **OR (95% CI)** | **N** | **OR (95% CI)** |
| Sum Bisphenols | 691 | 1.13 (0.46, 2.78) | 277 | 0.89 (0.29, 2.76) | 661 | 0.88 (0.37, 2.11) | 310 | 1.75 (0.64, 4.81) |
| Bisphenol A | 1114 | 1.15 (0.61, 2.16) | 313 | 0.74 (0.33, 1.68) | 778 | 1.1 (0.59, 2.04) | 621 | 1.77 (0.82, 3.79) |
| Bisphenol F | 692 | 1.34 (0.74, 2.42) | 278 | 1.5 (0.65, 3.46) | 661 | 0.77 (0.37, 1.59) | 310 | 0.87 (0.36, 2.08) |
| Bisphenol S | 806 | 0.68 (0.33, 1.4) | 277 | 0.87 (0.39, 1.95) | 704 | 0.65 (0.34, 1.24) | 384 | 2.07 (0.94, 4.58) |
| 2,4-Dichlorophenol | 660 | 0.74 (0.37, 1.5) |  |  | 334 | 0.99 (0.39, 2.51) | 412 | 0.99 (0.45, 2.17) |
| 2,5-Dichlorophenol | 660 | 0.86 (0.55, 1.35) |  |  | 334 | 1.13 (0.64, 2.01) | 412 | 0.97 (0.56, 1.69) |
| Benzophenone-3 | 813 | 0.9 (0.58, 1.4) | 84 | 0.35 (0.06, 2.07) | 484 | 0.98 (0.6, 1.62) | 416 | 0.91 (0.5, 1.66) |
| Triclosan | 825 | 1.19 (0.81, 1.76) | 93 | 0.85 (0.28, 2.59) | 485 | 1.1 (0.72, 1.7) | 417 | 1.51 (0.88, 2.59) |
| Butyl paraben | 673 | 1.06 (0.68, 1.66) |  |  | 347 | 1.03 (0.63, 1.68) | 406 | 1.23 (0.7, 2.15) |
| Ethyl paraben | 453 | 1.19 (0.7, 2.03) |  |  | 344 | 0.76 (0.38, 1.49) | 184 | 1.65 (0.82, 3.31) |
| Methyl Paraben | 689 | 0.81 (0.46, 1.42) | 17 | 2.06 (0.09, 46.09) | 347 | 0.89 (0.45, 1.77) | 418 | 1.02 (0.5, 2.11) |
| Propyl paraben | 689 | 0.82 (0.52, 1.28) | 17 | 1.29 (0.1, 15.88) | 347 | 1.04 (0.6, 1.79) | 418 | 0.83 (0.47, 1.48) |

Outcome: Low Birthweight (Preterms only)

|  | **Across Pregnancy** | | **Trimester 1** | | **Trimester 2** | | **Trimester 3** | |
| --- | --- | --- | --- | --- | --- | --- | --- | --- |
| **Phenol Name** | **N** | **OR (95% CI)** | **N** | **OR (95% CI)** | **N** | **OR (95% CI)** | **N** | **OR (95% CI)** |
| Sum Bisphenols | 50 | 0.69 (0.07, 7.15) | 17 | 0.13 (0, 9.7) | 47 | 0.57 (0.1, 3.19) |  |  |
| Bisphenol A | 71 | 0.53 (0.13, 2.21) | 23 | 0.52 (0.06, 4.23) | 56 | 1.02 (0.3, 3.46) | 30 | 116.76 (0.33, 41153.49) |
| Bisphenol F | 51 | 1.52 (0.26, 8.89) | 18 | 8.41 (0.11, 662.94) | 47 | 0.42 (0.08, 2.15) | 17 | 2.45 (0.01, 564.37) |
| Bisphenol S | 53 | 0.44 (0.08, 2.38) | 17 | 0.19 (0.01, 3.37) | 48 | 0.22 (0.03, 1.37) | 19 | 13.82 (0.09, 2064.21) |
| 2,4-Dichlorophenol | 41 | 0.09 (0.01, 1.23) |  |  | 25 | 0.25 (0.02, 3.27) |  |  |
| 2,5-Dichlorophenol | 41 | 0.17 (0.02, 1.19) |  |  | 25 | 0.4 (0.05, 3.18) | 22 | 0 (0, 63.04) |
| Benzophenone-3 | 52 | 1.46 (0.43, 4.99) | 10 | 0 (0, 80) | 36 | 1.13 (0.25, 5.16) | 22 | 1.1 (0.03, 41.3) |
| Triclosan | 54 | 0.6 (0.27, 1.33) |  |  | 36 | 0.38 (0.13, 1.13) | 23 | 0.02 (0, 76.01) |
| Butyl paraben | 41 | 2.88 (0.66, 12.53) |  |  | 25 | 7.61 (0.27, 215.59) |  |  |
| Ethyl paraben | 32 | 1.16 (0.39, 3.44) |  |  | 24 | 0.72 (0.2, 2.59) |  |  |
| Methyl Paraben | 43 | 1.79 (0.43, 7.53) |  |  | 25 | 2.22 (0.21, 23.46) | 23 | 88.97 (0.28, 28391.91) |
| Propyl paraben | 43 | 0.89 (0.31, 2.58) |  |  | 25 | 0.67 (0.12, 3.58) | 23 | 3.53 (0.39, 31.91) |

Outcome: Low Birthweight (Terms only)

|  | **Across Pregnancy** | | **Trimester 1** | | **Trimester 2** | | **Trimester 3** | |
| --- | --- | --- | --- | --- | --- | --- | --- | --- |
| **Phenol Name** | **N** | **OR (95% CI)** | **N** | **OR (95% CI)** | **N** | **OR (95% CI)** | **N** | **OR (95% CI)** |
| Sum Bisphenols | 641 | 1.28 (0.35, 4.74) | 260 | 1.12 (0.27, 4.72) | 614 | 1 (0.26, 3.84) | 293 | 1.03 (0.26, 4.03) |
| Bisphenol A | 1043 | 1.27 (0.53, 3.05) | 290 | 0.7 (0.24, 2.06) | 722 | 0.91 (0.36, 2.28) | 591 | 1.38 (0.54, 3.54) |
| Bisphenol F | 641 | 1.62 (0.7, 3.75) | 260 | 1.22 (0.36, 4.07) | 614 | 1.3 (0.49, 3.48) | 293 | 0.67 (0.2, 2.28) |
| Bisphenol S | 753 | 0.62 (0.22, 1.74) | 260 | 0.95 (0.33, 2.71) | 656 | 0.77 (0.3, 1.99) | 365 | 1.8 (0.63, 5.12) |
| 2,4-Dichlorophenol | 619 | 0.76 (0.31, 1.87) |  |  | 309 | 0.8 (0.16, 3.92) | 390 | 0.91 (0.35, 2.37) |
| 2,5-Dichlorophenol | 619 | 0.92 (0.51, 1.64) |  |  | 309 | 0.98 (0.4, 2.42) | 390 | 0.89 (0.46, 1.75) |
| Benzophenone-3 | 761 | 1.32 (0.73, 2.39) | 74 | 3.26 (0.21, 51.69) | 448 | 1.56 (0.73, 3.31) | 394 | 1.24 (0.6, 2.55) |
| Triclosan | 771 | 1.29 (0.75, 2.24) |  |  | 449 | 1.25 (0.63, 2.5) | 394 | 1.42 (0.73, 2.74) |
| Butyl paraben | 632 | 1.11 (0.61, 2) |  |  | 322 | 1.4 (0.71, 2.77) | 384 | 0.94 (0.44, 2.02) |
| Ethyl paraben | 421 | 1.04 (0.44, 2.43) |  |  | 320 | 0.39 (0.11, 1.37) | 172 | 1.91 (0.68, 5.36) |
| Methyl Paraben | 646 | 0.85 (0.4, 1.82) |  |  | 322 | 0.96 (0.35, 2.66) | 395 | 0.98 (0.39, 2.45) |
| Propyl paraben | 646 | 0.96 (0.53, 1.76) |  |  | 322 | 1.39 (0.61, 3.17) | 395 | 0.79 (0.38, 1.63) |

**Supplement Table 3a1: Education-Stratified Adjusted Associations of Phenol Exposures with Birth Outcomes (Continuously Measured),
High School Education/GED or less Only**Models adjusted for maternal race/ethnicity and parity, and child sex. BW for GA model removes parity from covariates. Blank cells reflect model non-convergence.

Outcome: Gestational Age

|  | **Across Pregnancy** | | **Trimester 1** | | **Trimester 2** | | **Trimester 3** | |
| --- | --- | --- | --- | --- | --- | --- | --- | --- |
| **Phenol Name** | **N** | **Beta (95% CI)** | **N** | **Beta (95% CI)** | **N** | **Beta (95% CI)** | **N** | **Beta (95% CI)** |
| Sum Bisphenols | 297 | -0.111 (-0.717, 0.496) | 190 | 0.029 (-0.59, 0.647) | 286 | -0.136 (-0.734, 0.462) | 173 | -0.055 (-0.551, 0.44) |
| Bisphenol A | 798 | 0.062 (-0.217, 0.342) | 272 | -0.1 (-0.509, 0.308) | 410 | -0.062 (-0.398, 0.274) | 556 | -0.014 (-0.288, 0.261) |
| Bisphenol F | 319 | -0.016 (-0.433, 0.401) | 204 | -0.031 (-0.538, 0.476) | 293 | 0.14 (-0.344, 0.624) | 174 | 0.127 (-0.275, 0.53) |
| Bisphenol S | 433 | -0.213 (-0.578, 0.153) | 192 | -0.221 (-0.669, 0.227) | 331 | -0.095 (-0.471, 0.28) | 262 | -0.161 (-0.471, 0.149) |
| 2,4-Dichlorophenol | 458 | 0.223 (-0.018, 0.463) |  |  | 94 | 0.224 (-0.445, 0.894) | 379 | 0.151 (-0.104, 0.406) |
| 2,5-Dichlorophenol | 457 | **0.246 (0.07, 0.422)** |  |  | 94 | 0.257 (-0.165, 0.678) | 378 | 0.165 (-0.022, 0.353) |
| Benzophenone-3 | 490 | -0.122 (-0.329, 0.085) | 27 | -0.484 (-1.141, 0.173) | 125 | 0.024 (-0.359, 0.407) | 379 | -0.058 (-0.287, 0.171) |
| Triclosan | 553 | -0.008 (-0.178, 0.162) | 59 | -0.231 (-0.66, 0.198) | 145 | 0.051 (-0.25, 0.352) | 390 | -0.014 (-0.214, 0.186) |
| Butyl paraben | 507 | 0.087 (-0.095, 0.269) | 38 | 0.135 (-0.494, 0.765) | 117 | 0.241 (-0.067, 0.549) | 374 | -0.053 (-0.276, 0.17) |
| Ethyl paraben | 275 | 0.079 (-0.164, 0.323) | 52 | -0.143 (-0.785, 0.499) | 122 | 0.068 (-0.313, 0.449) | 124 | 0.035 (-0.278, 0.348) |
| Methyl Paraben | 545 | 0.098 (-0.116, 0.313) | 52 | 0.059 (-0.531, 0.648) | 124 | 0.161 (-0.219, 0.541) | 391 | -0.023 (-0.285, 0.239) |
| Propyl paraben | 539 | 0.148 (-0.025, 0.32) | 51 | 0.156 (-0.332, 0.645) | 120 | 0.291 (-0.037, 0.619) | 390 | 0.034 (-0.174, 0.241) |

Outcome: Birth Length

|  | **Across Pregnancy** | | **Trimester 1** | | **Trimester 2** | | **Trimester 3** | |
| --- | --- | --- | --- | --- | --- | --- | --- | --- |
| **Phenol Name** | **N** | **Beta (95% CI)** | **N** | **Beta (95% CI)** | **N** | **Beta (95% CI)** | **N** | **Beta (95% CI)** |
| Sum Bisphenols | 278 | 0.29 (-0.653, 1.233) | 179 | -0.075 (-1.045, 0.896) | 268 | -0.097 (-1.04, 0.846) | 163 | -0.434 (-1.254, 0.386) |
| Bisphenol A | 702 | 0.311 (-0.174, 0.797) | 210 | -0.375 (-1.083, 0.333) | 350 | -0.06 (-0.617, 0.496) | 511 | 0.139 (-0.369, 0.648) |
| Bisphenol F | 297 | -0.111 (-0.757, 0.534) | 192 | -0.451 (-1.259, 0.357) | 274 | -0.347 (-1.1, 0.405) | 163 | 0.168 (-0.506, 0.843) |
| Bisphenol S | 413 | 0.088 (-0.513, 0.69) | 181 | -0.292 (-0.98, 0.397) | 313 | 0.215 (-0.389, 0.819) | 251 | -0.436 (-0.985, 0.114) |
| 2,4-Dichlorophenol | 434 | **0.44 (0.003, 0.878)** |  |  | 89 | 0.278 (-0.839, 1.395) | 360 | 0.433 (-0.053, 0.918) |
| 2,5-Dichlorophenol | 433 | **0.5 (0.184, 0.816)** |  |  | 89 | 0.139 (-0.563, 0.841) | 359 | **0.54 (0.189, 0.891)** |
| Benzophenone-3 | 465 | **-0.609 (-0.99, -0.228)** | 27 | -0.978 (-2.245, 0.29) | 119 | -0.476 (-1.177, 0.224) | 360 | **-0.649 (-1.085, -0.212)** |
| Triclosan | 463 | **0.435 (0.101, 0.768)** | 25 | **1.205 (0.14, 2.27)** | 118 | 0.296 (-0.301, 0.892) | 361 | **0.475 (0.09, 0.859)** |
| Butyl paraben | 438 | 0.317 (-0.039, 0.673) | 15 | -1.141 (-2.402, 0.12) | 96 | 0.142 (-0.397, 0.681) | 349 | **0.465 (0.03, 0.9)** |
| Ethyl paraben | 201 | 0.19 (-0.272, 0.653) | 17 | -0.989 (-2.063, 0.086) | 95 | **0.735 (0.06, 1.41)** | 112 | -0.026 (-0.675, 0.624) |
| Methyl Paraben | 453 | 0.031 (-0.412, 0.474) | 17 | -1.89 (-4.633, 0.853) | 97 | 0.102 (-0.605, 0.81) | 361 | -0.042 (-0.57, 0.486) |
| Propyl paraben | 453 | 0.076 (-0.275, 0.426) | 17 | **-1.639 (-2.939, -0.338)** | 97 | 0.298 (-0.269, 0.865) | 361 | 0.054 (-0.359, 0.466) |

Outcome: Birthweight

|  | **Across Pregnancy** | | **Trimester 1** | | **Trimester 2** | | **Trimester 3** | |
| --- | --- | --- | --- | --- | --- | --- | --- | --- |
| **Phenol Name** | **N** | **Beta (95% CI)** | **N** | **Beta (95% CI)** | **N** | **Beta (95% CI)** | **N** | **Beta (95% CI)** |
| Sum Bisphenols | 297 | 66.984 (-116.968, 250.936) | 190 | 128.833 (-59.816, 317.481) | 286 | 21.031 (-160.176, 202.239) | 173 | **-174.608 (-335.793, -13.422)** |
| Bisphenol A | 798 | 40.024 (-42.223, 122.272) | 272 | 29.624 (-93.515, 152.763) | 410 | 14.645 (-84.576, 113.866) | 556 | -15.416 (-100.322, 69.489) |
| Bisphenol F | 319 | -30.543 (-156.154, 95.068) | 204 | 18.242 (-137.834, 174.317) | 293 | -67.794 (-212.427, 76.838) | 174 | -58.24 (-190.382, 73.901) |
| Bisphenol S | 433 | 33.664 (-77.128, 144.456) | 192 | 0.949 (-136.389, 138.286) | 331 | 95.404 (-17.591, 208.399) | 262 | -91.106 (-190.362, 8.151) |
| 2,4-Dichlorophenol | 458 | **73.699 (4.198, 143.2)** |  |  | 94 | -9.023 (-195.865, 177.819) | 379 | **83.272 (6.577, 159.967)** |
| 2,5-Dichlorophenol | 457 | **81.352 (30.864, 131.841)** |  |  | 94 | 63.95 (-53.71, 181.611) | 378 | **78.532 (22.593, 134.47)** |
| Benzophenone-3 | 490 | **-94.206 (-155.163, -33.248)** | 27 | **-325.752 (-546.3, -105.204)** | 125 | -92.204 (-214.652, 30.245) | 379 | **-73.579 (-142.38, -4.779)** |
| Triclosan | 553 | 27.799 (-23.694, 79.293) | 59 | 17.175 (-129.99, 164.34) | 145 | 19.204 (-81.422, 119.83) | 390 | 44.449 (-15.891, 104.789) |
| Butyl paraben | 507 | 20.626 (-32.507, 73.759) | 38 | -166.958 (-370.631, 36.716) | 117 | 19.591 (-68.748, 107.931) | 374 | 29.013 (-37.817, 95.843) |
| Ethyl paraben | 275 | -18.119 (-90.917, 54.679) | 52 | -133.086 (-333.317, 67.145) | 122 | 79.268 (-29.994, 188.529) | 124 | -34.612 (-136.114, 66.889) |
| Methyl Paraben | 545 | 17.87 (-45.363, 81.102) | 52 | 31.875 (-154.469, 218.22) | 124 | 24.523 (-85.366, 134.412) | 391 | -2.727 (-82.319, 76.866) |
| Propyl paraben | 539 | 42.341 (-8.641, 93.324) | 51 | 20.688 (-135.383, 176.758) | 120 | 68.263 (-26.715, 163.24) | 390 | 24.903 (-37.909, 87.715) |

Outcome: Birthweight for Gestational Age Z-Score

|  | **Across Pregnancy** | | **Trimester 1** | | **Trimester 2** | | **Trimester 3** | |
| --- | --- | --- | --- | --- | --- | --- | --- | --- |
| **Phenol Name** | **N** | **Beta (95% CI)** | **N** | **Beta (95% CI)** | **N** | **Beta (95% CI)** | **N** | **Beta (95% CI)** |
| Sum Bisphenols | 297 | 0.185 (-0.186, 0.556) | 190 | 0.28 (-0.075, 0.634) | 286 | 0.104 (-0.259, 0.467) | 173 | **-0.43 (-0.779, -0.08)** |
| Bisphenol A | 794 | 0.06 (-0.123, 0.244) | 272 | 0.066 (-0.182, 0.315) | 410 | 0.039 (-0.168, 0.246) | 553 | -0.012 (-0.212, 0.187) |
| Bisphenol F | 319 | -0.095 (-0.353, 0.162) | 204 | 0.046 (-0.258, 0.349) | 293 | -0.242 (-0.532, 0.048) | 174 | -0.197 (-0.489, 0.094) |
| Bisphenol S | 433 | 0.129 (-0.1, 0.359) | 192 | 0.074 (-0.181, 0.329) | 331 | **0.261 (0.03, 0.492)** | 262 | -0.192 (-0.41, 0.026) |
| 2,4-Dichlorophenol | 454 | 0.118 (-0.054, 0.291) |  |  | 94 | -0.123 (-0.621, 0.374) | 376 | 0.181 (-0.009, 0.371) |
| 2,5-Dichlorophenol | 453 | 0.113 (-0.013, 0.239) |  |  | 94 | 0.008 (-0.308, 0.324) | 375 | **0.153 (0.015, 0.291)** |
| Benzophenone-3 | 486 | **-0.156 (-0.308, -0.004)** | 27 | **-0.569 (-1.039, -0.1)** | 125 | -0.163 (-0.471, 0.145) | 376 | -0.142 (-0.314, 0.029) |
| Triclosan | 549 | 0.103 (-0.023, 0.228) | 59 | 0.14 (-0.166, 0.446) | 145 | 0.051 (-0.193, 0.295) | 387 | 0.144 (-0.005, 0.293) |
| Butyl paraben | 503 | 0.008 (-0.123, 0.139) | 38 | **-0.54 (-0.991, -0.089)** | 117 | -0.054 (-0.274, 0.166) | 371 | 0.113 (-0.052, 0.279) |
| Ethyl paraben | 275 | -0.091 (-0.261, 0.079) | 52 | -0.288 (-0.731, 0.156) | 122 | 0.169 (-0.096, 0.435) | 124 | -0.124 (-0.361, 0.113) |
| Methyl Paraben | 541 | 0.009 (-0.143, 0.162) | 52 | -0.038 (-0.438, 0.362) | 124 | -0.016 (-0.286, 0.254) | 388 | 0.038 (-0.158, 0.235) |
| Propyl paraben | 535 | 0.053 (-0.07, 0.176) | 51 | -0.079 (-0.414, 0.255) | 120 | 0.051 (-0.182, 0.283) | 387 | 0.074 (-0.081, 0.229) |

**Supplement Table 3b1: Education-Stratified Adjusted Associations of Phenol Exposures with Categorical Birth Outcomes,
High School Education/GED or less Only**Models adjusted for maternal race/ethnicity and parity, and child sex. Blank cells reflect model non-convergence.

Outcome: Preterm

|  | **Across Pregnancy** | | **Trimester 1** | | **Trimester 2** | | **Trimester 3** | |
| --- | --- | --- | --- | --- | --- | --- | --- | --- |
| **Phenol Name** | **N** | **OR (95% CI)** | **N** | **OR (95% CI)** | **N** | **OR (95% CI)** | **N** | **OR (95% CI)** |
| Sum Bisphenols | 297 | 1.01 (0.29, 3.6) | 190 | 0.57 (0.12, 2.73) | 286 | 1.54 (0.44, 5.4) | 173 | 0.52 (0.08, 3.44) |
| Bisphenol A | 798 | 0.96 (0.49, 1.87) | 272 | 0.62 (0.24, 1.61) | 410 | 0.98 (0.48, 2.02) | 556 | 1.64 (0.69, 3.86) |
| Bisphenol F | 319 | 1.08 (0.5, 2.33) | 204 | 1.32 (0.51, 3.4) | 293 | 1.18 (0.47, 2.97) | 174 | 0.57 (0.12, 2.73) |
| Bisphenol S | 433 | 1.15 (0.47, 2.83) | 192 | 1.26 (0.44, 3.64) | 331 | 0.84 (0.35, 2.03) | 262 | 0.8 (0.28, 2.3) |
| 2,4-Dichlorophenol | 458 | 0.85 (0.44, 1.62) |  |  | 94 | 0.24 (0.02, 3.77) | 379 | 1.02 (0.5, 2.1) |
| 2,5-Dichlorophenol | 457 | 0.82 (0.52, 1.32) |  |  | 94 | 0.39 (0.06, 2.43) | 378 | 0.99 (0.58, 1.68) |
| Benzophenone-3 | 490 | 1.54 (0.92, 2.6) | 27 | 187.84 (0, .) |  |  | 379 | 1.49 (0.83, 2.67) |
| Triclosan | 553 | 1.16 (0.72, 1.85) | 59 | 2.53 (0.46, 13.83) | 145 | 0.29 (0.06, 1.57) | 390 | 1.26 (0.73, 2.17) |
| Butyl paraben | 507 | 0.89 (0.56, 1.42) | 38 | 0.67 (0.1, 4.52) | 117 | 0.39 (0.07, 2.07) | 374 | 1.18 (0.66, 2.11) |
| Ethyl paraben | 275 | 0.56 (0.29, 1.11) | 52 | 0.91 (0.25, 3.29) | 122 | 0.28 (0.06, 1.33) | 124 | 0.9 (0.28, 2.87) |
| Methyl Paraben | 545 | 0.9 (0.53, 1.53) | 52 | 0.96 (0.27, 3.38) | 124 | 0.54 (0.14, 2.06) | 391 | 1.29 (0.62, 2.69) |
| Propyl paraben | 539 | 0.93 (0.61, 1.43) | 51 | 0.94 (0.36, 2.47) | 120 | 0.62 (0.22, 1.79) | 390 | 1.21 (0.68, 2.15) |

Outcome: Small for Gestational Age

|  | **Across Pregnancy** | | **Trimester 1** | | **Trimester 2** | | **Trimester 3** | |
| --- | --- | --- | --- | --- | --- | --- | --- | --- |
| **Phenol Name** | **N** | **OR (95% CI)** | **N** | **OR (95% CI)** | **N** | **OR (95% CI)** | **N** | **OR (95% CI)** |
| Sum Bisphenols | 297 | 1.46 (0.52, 4.12) | 190 | 0.62 (0.2, 1.93) | 286 | 1.79 (0.64, 5.02) | 173 | **3.55 (1.3, 9.71)** |
| Bisphenol A | 794 | 1.06 (0.63, 1.78) | 272 | 0.75 (0.36, 1.56) | 410 | 1.23 (0.68, 2.25) | 553 | 1.27 (0.73, 2.2) |
| Bisphenol F | 319 | 1.48 (0.76, 2.9) | 204 | 0.99 (0.41, 2.4) | 293 | 2.11 (0.98, 4.54) | 174 | 1.42 (0.68, 3) |
| Bisphenol S | 433 | 1.15 (0.59, 2.25) | 192 | 1.03 (0.46, 2.32) | 331 | 0.9 (0.46, 1.77) | 262 | 1.7 (0.87, 3.29) |
| 2,4-Dichlorophenol | 454 | 0.81 (0.5, 1.31) |  |  | 94 | 1.37 (0.37, 5.01) | 376 | 0.63 (0.37, 1.07) |
| 2,5-Dichlorophenol | 453 | 0.86 (0.61, 1.21) |  |  | 94 | 0.92 (0.38, 2.23) | 375 | 0.74 (0.52, 1.07) |
| Benzophenone-3 | 486 | 1.43 (0.96, 2.13) | 27 | 1.61 (0.02, 167.82) | 125 | 0.86 (0.36, 2.03) | 376 | **1.57 (1.02, 2.4)** |
| Triclosan | 549 | 1.05 (0.75, 1.48) | 59 | 0.34 (0.07, 1.73) | 145 | 1.31 (0.66, 2.59) |  |  |
| Butyl paraben | 503 | 1.08 (0.77, 1.52) | 38 | 10.93 (0.57, 210.33) | 117 | 1.13 (0.64, 2) | 371 | 0.85 (0.54, 1.33) |
| Ethyl paraben | 275 | **1.58 (1.02, 2.44)** | 52 | 0.98 (0.29, 3.25) | 122 | 0.52 (0.19, 1.42) | 124 | **2.67 (1.4, 5.11)** |
| Methyl Paraben | 541 | 1.1 (0.73, 1.64) | 52 | 1.06 (0.29, 3.91) | 124 | 1.33 (0.62, 2.86) | 388 | 0.89 (0.54, 1.47) |
| Propyl paraben | 535 | 0.89 (0.64, 1.23) | 51 | 0.93 (0.37, 2.36) | 120 | 1.02 (0.55, 1.91) |  |  |

Outcome: Large for Gestational Age

|  | **Across Pregnancy** | | **Trimester 1** | | **Trimester 2** | | **Trimester 3** | |
| --- | --- | --- | --- | --- | --- | --- | --- | --- |
| **Phenol Name** | **N** | **OR (95% CI)** | **N** | **OR (95% CI)** | **N** | **OR (95% CI)** | **N** | **OR (95% CI)** |
| Sum Bisphenols | 297 | **4.06 (1.47, 11.18)** | 190 | 2.6 (0.76, 8.87) | 286 | **3.69 (1.22, 11.15)** | 173 | 0.49 (0.11, 2.23) |
| Bisphenol A | 794 | 1.3 (0.75, 2.26) | 272 | 1.24 (0.51, 3.02) | 410 | 1.31 (0.67, 2.59) | 553 | 1.24 (0.65, 2.36) |
| Bisphenol F | 319 | 1.89 (0.95, 3.75) | 204 | **3.45 (1.36, 8.71)** | 293 | 1.32 (0.56, 3.13) | 174 | 0.58 (0.14, 2.31) |
| Bisphenol S | 433 | 2.17 (0.98, 4.82) | 192 | 0.59 (0.22, 1.6) | 331 | **3.73 (1.6, 8.71)** | 262 | 0.53 (0.2, 1.4) |
| 2,4-Dichlorophenol | 454 | 1.03 (0.62, 1.7) |  |  |  |  |  |  |
| 2,5-Dichlorophenol |  |  |  |  |  |  |  |  |
| Benzophenone-3 | 486 | 0.72 (0.46, 1.13) |  |  | 125 | 0.73 (0.31, 1.72) |  |  |
| Triclosan |  |  | 59 | 2.27 (0.52, 9.88) |  |  | 387 | 1.43 (0.95, 2.14) |
| Butyl paraben |  |  | 38 | 0.04 (0, 36.28) |  |  | 371 | 1.22 (0.78, 1.93) |
| Ethyl paraben | 275 | 1.15 (0.6, 2.2) | 52 | 0.42 (0.06, 2.81) |  |  | 124 | 2.07 (0.67, 6.4) |
| Methyl Paraben | 541 | 1.06 (0.66, 1.71) | 52 | 5.2 (0.43, 63.21) |  |  | 388 | 0.86 (0.49, 1.53) |
| Propyl paraben |  |  | 51 | 2.27 (0.54, 9.58) |  |  | 387 | 0.98 (0.63, 1.53) |

Outcome: Low Birthweight

|  | **Across Pregnancy** | | **Trimester 1** | | **Trimester 2** | | **Trimester 3** | |
| --- | --- | --- | --- | --- | --- | --- | --- | --- |
| **Phenol Name** | **N** | **OR (95% CI)** | **N** | **OR (95% CI)** | **N** | **OR (95% CI)** | **N** | **OR (95% CI)** |
| Sum Bisphenols | 297 | 0.67 (0.18, 2.47) | 190 | 0.5 (0.14, 1.83) | 286 | 0.88 (0.25, 3.05) | 173 | 1.29 (0.32, 5.26) |
| Bisphenol A | 798 | 0.85 (0.42, 1.72) | 272 | 0.87 (0.38, 1.99) | 410 | 0.97 (0.46, 2.05) | 556 | 1.67 (0.72, 3.87) |
| Bisphenol F | 319 | 1.16 (0.54, 2.5) | 204 | 1.3 (0.56, 3.04) | 293 | 1.47 (0.6, 3.6) | 174 | 0.58 (0.17, 2.05) |
| Bisphenol S | 433 | 1.3 (0.52, 3.23) | 192 | 0.83 (0.34, 2.03) | 331 | 0.92 (0.39, 2.16) | 262 | **2.56 (1.01, 6.48)** |
| 2,4-Dichlorophenol | 458 | 0.43 (0.17, 1.08) |  |  | 94 | 0.15 (0, 15.54) | 379 | 0.4 (0.15, 1.06) |
| 2,5-Dichlorophenol | 457 | **0.5 (0.27, 0.92)** |  |  | 94 | 0.33 (0.02, 6.53) |  |  |
| Benzophenone-3 | 490 | 1.08 (0.54, 2.18) |  |  | 125 | 11.48 (0.48, 276.42) | 379 | 0.76 (0.34, 1.71) |
| Triclosan | 553 | 1.12 (0.61, 2.03) | 59 | 2.11 (0.45, 9.84) | 145 | 1.21 (0.18, 8.3) | 390 | 1.03 (0.52, 2.03) |
| Butyl paraben |  |  | 38 | 3.77 (0.5, 28.32) | 117 | 0.44 (0.06, 3.47) | 374 | 0.72 (0.32, 1.62) |
| Ethyl paraben | 275 | 1.7 (0.88, 3.28) | 52 | 1.62 (0.41, 6.31) | 122 | 0.06 (0, 5.59) | 124 | 4.43 (0.92, 21.27) |
| Methyl Paraben | 545 | 0.79 (0.41, 1.51) | 52 | 0.8 (0.21, 2.98) | 124 | 0.77 (0.12, 4.77) | 391 | 0.87 (0.36, 2.07) |
| Propyl paraben | 539 | 0.87 (0.51, 1.48) | 51 | 1.26 (0.48, 3.31) |  |  | 390 | 0.82 (0.4, 1.66) |

Outcome: Low Birthweight (Preterms only)

|  | **Across Pregnancy** | | **Trimester 1** | | **Trimester 2** | | **Trimester 3** | |
| --- | --- | --- | --- | --- | --- | --- | --- | --- |
| **Phenol Name** | **N** | **OR (95% CI)** | **N** | **OR (95% CI)** | **N** | **OR (95% CI)** | **N** | **OR (95% CI)** |
| Sum Bisphenols | 23 | 10.05 (0.02, 4679.29) |  |  | 22 | 2.77 (0.03, 291.34) |  |  |
| Bisphenol A | 59 | 0.31 (0.07, 1.43) | 22 | 4.03 (0.18, 89.98) | 32 | 2.25 (0.14, 35.53) | 35 | 1.19 (0.11, 13.2) |
| Bisphenol F | 31 | 0.71 (0.03, 16.94) | 17 | 8.02 (0.03, 2075.44) | 25 | 2.25 (0.05, 93.05) |  |  |
| Bisphenol S | 28 | 1.66 (0.13, 20.68) |  |  | 22 | 1.2 (0.13, 11.32) |  |  |
| 2,4-Dichlorophenol | 29 | 0.04 (0, 8.22) |  |  |  |  | 23 | 0.05 (0, 9.32) |
| 2,5-Dichlorophenol | 29 | 0.08 (0, 6.87) |  |  |  |  | 23 | 0.05 (0, 14.64) |
| Benzophenone-3 | 29 | 1.92 (0.1, 35.58) |  |  |  |  | 23 | 0.39 (0.01, 24.81) |
| Triclosan | 33 | 2.56 (0.57, 11.62) |  |  |  |  | 24 | 3.91 (0.15, 103.27) |
| Butyl paraben | 38 | 0.19 (0.02, 2.43) |  |  |  |  |  |  |
| Ethyl paraben | 23 | 3 (0.61, 14.7) |  |  |  |  |  |  |
| Methyl Paraben | 41 | 0.67 (0.15, 2.97) |  |  |  |  | 24 | 0.42 (0.02, 7.77) |
| Propyl paraben | 41 | 0.57 (0.19, 1.74) |  |  |  |  | 24 | 0.35 (0.03, 3.61) |

Outcome: Low Birthweight (Terms only)

|  | **Across Pregnancy** | | **Trimester 1** | | **Trimester 2** | | **Trimester 3** | |
| --- | --- | --- | --- | --- | --- | --- | --- | --- |
| **Phenol Name** | **N** | **OR (95% CI)** | **N** | **OR (95% CI)** | **N** | **OR (95% CI)** | **N** | **OR (95% CI)** |
| Sum Bisphenols | 274 | 0.75 (0.12, 4.9) | 178 | 0.35 (0.06, 2.05) | 264 | 1.05 (0.18, 6.15) | 163 | 1.04 (0.19, 5.69) |
| Bisphenol A | 739 | 1.31 (0.51, 3.39) |  |  | 378 | 1.14 (0.38, 3.36) | 521 | 1.58 (0.59, 4.21) |
| Bisphenol F | 288 | 1.34 (0.43, 4.13) |  |  | 268 | 1.94 (0.56, 6.75) | 164 | 0.41 (0.08, 2.17) |
| Bisphenol S | 405 | 1.79 (0.5, 6.45) |  |  | 309 | 1.59 (0.46, 5.52) | 248 | 2.3 (0.74, 7.11) |
| 2,4-Dichlorophenol | 429 | 0.48 (0.16, 1.4) |  |  |  |  | 356 | 0.43 (0.14, 1.26) |
| 2,5-Dichlorophenol |  |  |  |  |  |  |  |  |
| Benzophenone-3 | 461 | 0.59 (0.22, 1.61) |  |  |  |  | 356 | 0.65 (0.25, 1.69) |
| Triclosan | 520 | 1 (0.48, 2.07) | 55 | 0.91 (0.06, 13.95) |  |  | 366 | 0.92 (0.43, 2.01) |
| Butyl paraben |  |  | 32 | 6.57 (0.43, 101.6) |  |  | 351 | 0.92 (0.39, 2.18) |
| Ethyl paraben | 252 | 2.36 (0.88, 6.29) | 44 | 1.07 (0.21, 5.41) |  |  | 118 | 13.18 (0.95, 183.61) |
| Methyl Paraben | 504 | 0.9 (0.39, 2.08) | 44 | 0.78 (0.08, 7.26) |  |  | 367 | 0.8 (0.29, 2.18) |
| Propyl paraben | 498 | 1.08 (0.55, 2.14) | 43 | 2.39 (0.46, 12.54) |  |  | 366 | 0.76 (0.34, 1.73) |

**Supplement Table 3a2: Education-Stratified Adjusted Associations of Phenol Exposures with Birth Outcomes (Continuously Measured),
Some College or more Only**Models adjusted for maternal race/ethnicity and parity, and child sex. BW for GA model removes parity from covariates.

Outcome: Gestational Age

|  | **Across Pregnancy** | | **Trimester 1** | | **Trimester 2** | | **Trimester 3** | |
| --- | --- | --- | --- | --- | --- | --- | --- | --- |
| **Phenol Name** | **N** | **Beta (95% CI)** | **N** | **Beta (95% CI)** | **N** | **Beta (95% CI)** | **N** | **Beta (95% CI)** |
| Sum Bisphenols | 1370 | 0.038 (-0.212, 0.289) | 511 | -0.225 (-0.589, 0.139) | 1271 | -0.101 (-0.328, 0.127) | 527 | 0.178 (-0.126, 0.483) |
| Bisphenol A | 2009 | 0.097 (-0.081, 0.275) | 655 | 0.031 (-0.259, 0.321) | 1621 | -0.051 (-0.225, 0.123) | 829 | 0.139 (-0.057, 0.335) |
| Bisphenol F | 1405 | 0.024 (-0.139, 0.188) | 539 | -0.234 (-0.531, 0.062) | 1283 | 0.12 (-0.056, 0.296) | 531 | 0.068 (-0.179, 0.315) |
| Bisphenol S | 1697 | -0.136 (-0.309, 0.037) | 522 | -0.212 (-0.462, 0.038) | 1411 | -0.107 (-0.283, 0.07) | 707 | -0.021 (-0.211, 0.17) |
| 2,4-Dichlorophenol | 1115 | 0.09 (-0.101, 0.281) | 41 | 0.323 (-0.637, 1.283) | 783 | -0.038 (-0.279, 0.204) | 405 | 0.059 (-0.226, 0.344) |
| 2,5-Dichlorophenol | 1115 | 0.039 (-0.086, 0.164) | 40 | 0.489 (-0.453, 1.431) | 785 | -0.052 (-0.209, 0.105) | 405 | 0.072 (-0.117, 0.26) |
| Benzophenone-3 | 1475 | 0.04 (-0.065, 0.145) | 232 | 0.315 (-0.028, 0.657) | 1134 | 0.079 (-0.04, 0.198) | 427 | 0.013 (-0.158, 0.184) |
| Triclosan | 1543 | -0.024 (-0.119, 0.072) | 279 | -0.014 (-0.271, 0.242) | 1144 | -0.05 (-0.155, 0.055) | 432 | -0.042 (-0.207, 0.123) |
| Butyl paraben | 1216 | **0.146 (0.037, 0.255)** | 88 | -0.024 (-0.372, 0.323) | 828 | **0.145 (0.018, 0.272)** | 429 | 0.05 (-0.135, 0.235) |
| Ethyl paraben | 1164 | -0.016 (-0.127, 0.096) | 119 | 0.045 (-0.261, 0.35) | 835 | 0.014 (-0.119, 0.148) | 341 | -0.033 (-0.224, 0.157) |
| Methyl Paraben | 1266 | 0.067 (-0.061, 0.195) | 121 | 0.17 (-0.18, 0.519) | 838 | 0.056 (-0.086, 0.199) | 441 | -0.044 (-0.248, 0.16) |
| Propyl paraben | 1260 | 0.06 (-0.042, 0.162) | 119 | 0.244 (-0.04, 0.529) | 837 | 0.003 (-0.109, 0.116) | 438 | 0.016 (-0.15, 0.183) |

Outcome: Birth Length

|  | **Across Pregnancy** | | **Trimester 1** | | **Trimester 2** | | **Trimester 3** | |
| --- | --- | --- | --- | --- | --- | --- | --- | --- |
| **Phenol Name** | **N** | **Beta (95% CI)** | **N** | **Beta (95% CI)** | **N** | **Beta (95% CI)** | **N** | **Beta (95% CI)** |
| Sum Bisphenols | 1332 | -0.051 (-0.47, 0.369) | 493 | -0.111 (-0.659, 0.437) | 1236 | -0.092 (-0.478, 0.294) | 504 | 0.26 (-0.254, 0.774) |
| Bisphenol A | 1928 | 0.063 (-0.228, 0.353) | 612 | 0.149 (-0.267, 0.564) | 1564 | -0.107 (-0.39, 0.176) | 785 | 0.246 (-0.091, 0.582) |
| Bisphenol F | 1365 | -0.162 (-0.439, 0.114) | 521 | **-0.516 (-0.977, -0.056)** | 1248 | 0.035 (-0.263, 0.333) | 506 | 0.156 (-0.26, 0.572) |
| Bisphenol S | 1655 | 0.258 (-0.044, 0.561) | 504 | -0.152 (-0.546, 0.243) | 1374 | 0.175 (-0.128, 0.478) | 682 | -0.159 (-0.499, 0.181) |
| 2,4-Dichlorophenol | 1091 | 0.267 (-0.073, 0.607) | 41 | **2.366 (0.389, 4.343)** | 769 | 0.288 (-0.143, 0.718) | 393 | 0.355 (-0.138, 0.847) |
| 2,5-Dichlorophenol | 1091 | **0.227 (0.005, 0.448)** | 40 | 0.497 (-1.563, 2.556) | 771 | 0.165 (-0.115, 0.445) | 393 | 0.221 (-0.103, 0.544) |
| Benzophenone-3 | 1449 | **-0.195 (-0.378, -0.012)** | 232 | 0.244 (-0.273, 0.761) | 1118 | -0.142 (-0.349, 0.065) | 415 | -0.211 (-0.504, 0.083) |
| Triclosan | 1413 | **-0.19 (-0.357, -0.023)** | 215 | -0.259 (-0.68, 0.163) | 1101 | **-0.224 (-0.405, -0.043)** | 407 | -0.066 (-0.359, 0.227) |
| Butyl paraben | 1119 | -0.068 (-0.278, 0.141) | 40 | 0.116 (-0.68, 0.911) | 795 | -0.081 (-0.321, 0.158) | 411 | -0.171 (-0.496, 0.153) |
| Ethyl paraben | 1042 | 0.053 (-0.173, 0.28) | 55 | 0.633 (-0.561, 1.828) | 794 | 0.077 (-0.176, 0.329) | 322 | -0.233 (-0.625, 0.158) |
| Methyl Paraben | 1136 | **-0.272 (-0.516, -0.029)** | 57 | 0.071 (-0.979, 1.122) | 797 | -0.239 (-0.504, 0.027) | 414 | **-0.361 (-0.721, 0)** |
| Propyl paraben | 1135 | **-0.242 (-0.437, -0.048)** | 56 | 0.806 (-0.041, 1.653) | 797 | **-0.233 (-0.444, -0.023)** | 414 | **-0.313 (-0.602, -0.025)** |

Outcome: Birthweight

|  | **Across Pregnancy** | | **Trimester 1** | | **Trimester 2** | | **Trimester 3** | |
| --- | --- | --- | --- | --- | --- | --- | --- | --- |
| **Phenol Name** | **N** | **Beta (95% CI)** | **N** | **Beta (95% CI)** | **N** | **Beta (95% CI)** | **N** | **Beta (95% CI)** |
| Sum Bisphenols | 1370 | -19.247 (-93.889, 55.395) | 511 | -24.721 (-125.223, 75.782) | 1271 | 2.411 (-65.684, 70.507) | 527 | 69.073 (-30.869, 169.015) |
| Bisphenol A | 2009 | 26.452 (-25.717, 78.621) | 655 | 51.155 (-27.114, 129.424) | 1621 | -2.733 (-54.113, 48.647) | 829 | 58.821 (-2.039, 119.681) |
| Bisphenol F | 1405 | -23.447 (-72.033, 25.139) | 539 | **-86.49 (-168.469, -4.512)** | 1283 | 25.376 (-27.329, 78.08) | 531 | 25.081 (-56.005, 106.166) |
| Bisphenol S | 1697 | 1.361 (-51.083, 53.805) | 522 | -21.723 (-91.387, 47.941) | 1411 | 17.961 (-35.525, 71.447) | 707 | -22.852 (-84.864, 39.159) |
| 2,4-Dichlorophenol | 1115 | 57.63 (-1.913, 117.173) | 41 | 120.834 (-196.543, 438.211) | 783 | **90.192 (11.035, 169.349)** | 405 | 60.02 (-25.575, 145.615) |
| 2,5-Dichlorophenol | 1115 | 14.398 (-24.717, 53.513) | 40 | 145.913 (-157.75, 449.576) | 785 | -4.585 (-56.177, 47.006) | 405 | 36.001 (-20.656, 92.658) |
| Benzophenone-3 | 1475 | -21.789 (-53.587, 10.01) | 232 | 25.541 (-68.729, 119.81) | 1134 | -12.81 (-49.674, 24.053) | 427 | -32.071 (-83.684, 19.541) |
| Triclosan | 1543 | -22.33 (-51.502, 6.842) | 279 | -51.105 (-123.764, 21.554) | 1144 | -19.706 (-52.33, 12.917) | 432 | -22.815 (-73.323, 27.693) |
| Butyl paraben | 1216 | -1.583 (-36.223, 33.057) | 88 | 69.936 (-53.328, 193.2) | 828 | 2.642 (-39.515, 44.798) | 429 | -32.993 (-88.923, 22.938) |
| Ethyl paraben | 1164 | -22.15 (-58.833, 14.533) | 119 | 24.406 (-77.913, 126.725) | 835 | -15.742 (-60.139, 28.655) | 341 | **-77.631 (-143.316, -11.947)** |
| Methyl Paraben | 1266 | **-64.384 (-104.717, -24.051)** | 121 | 29.473 (-88.058, 147.004) | 838 | **-55.83 (-102.728, -8.933)** | 441 | **-80.149 (-141.995, -18.303)** |
| Propyl paraben | 1260 | **-33.399 (-65.626, -1.171)** | 119 | 77.226 (-18.268, 172.72) | 837 | **-46.477 (-83.465, -9.489)** | 438 | -32.314 (-82.885, 18.257) |

Outcome: Birthweight for Gestational Age Z-Score

|  | **Across Pregnancy** | | **Trimester 1** | | **Trimester 2** | | **Trimester 3** | |
| --- | --- | --- | --- | --- | --- | --- | --- | --- |
| **Phenol Name** | **N** | **Beta (95% CI)** | **N** | **Beta (95% CI)** | **N** | **Beta (95% CI)** | **N** | **Beta (95% CI)** |
| Sum Bisphenols | 1370 | -0.05 (-0.212, 0.113) | 511 | -0.005 (-0.219, 0.21) | 1271 | 0.052 (-0.095, 0.199) | 527 | 0.111 (-0.096, 0.319) |
| Bisphenol A | 2006 | 0.023 (-0.09, 0.135) | 654 | 0.082 (-0.074, 0.238) | 1620 | 0.015 (-0.093, 0.124) | 827 | 0.085 (-0.047, 0.216) |
| Bisphenol F | 1405 | -0.051 (-0.157, 0.054) | 539 | -0.12 (-0.295, 0.054) | 1283 | 0.009 (-0.105, 0.122) | 531 | 0.047 (-0.121, 0.215) |
| Bisphenol S | 1697 | 0.046 (-0.069, 0.162) | 522 | 0.006 (-0.144, 0.157) | 1411 | 0.09 (-0.026, 0.205) | 707 | -0.069 (-0.201, 0.064) |
| 2,4-Dichlorophenol | 1113 | 0.116 (-0.021, 0.252) | 41 | 0.229 (-0.506, 0.964) | 783 | **0.242 (0.064, 0.419)** | 403 | 0.135 (-0.056, 0.326) |
| 2,5-Dichlorophenol | 1113 | 0.039 (-0.051, 0.128) | 40 | 0.021 (-0.645, 0.687) | 785 | 0.021 (-0.095, 0.136) | 403 | 0.085 (-0.041, 0.212) |
| Benzophenone-3 | 1473 | **-0.089 (-0.16, -0.019)** | 232 | -0.086 (-0.266, 0.094) | 1134 | -0.076 (-0.156, 0.004) | 425 | -0.11 (-0.224, 0.005) |
| Triclosan | 1541 | -0.054 (-0.118, 0.01) | 279 | -0.134 (-0.27, 0.002) | 1144 | -0.032 (-0.102, 0.039) | 430 | -0.053 (-0.166, 0.059) |
| Butyl paraben | 1214 | -0.066 (-0.145, 0.013) | 88 | 0.187 (-0.077, 0.451) | 828 | -0.06 (-0.155, 0.035) | 427 | -0.091 (-0.216, 0.033) |
| Ethyl paraben | 1164 | -0.045 (-0.127, 0.038) | 119 | 0.035 (-0.187, 0.258) | 835 | -0.038 (-0.137, 0.061) | 341 | **-0.183 (-0.324, -0.041)** |
| Methyl Paraben | 1264 | **-0.203 (-0.294, -0.112)** | 121 | -0.005 (-0.262, 0.251) | 838 | **-0.164 (-0.268, -0.059)** | 439 | **-0.199 (-0.336, -0.061)** |
| Propyl paraben | 1258 | **-0.116 (-0.189, -0.043)** | 119 | 0.079 (-0.131, 0.289) | 837 | **-0.111 (-0.193, -0.028)** | 436 | -0.105 (-0.218, 0.007) |

**Supplement Table 3b2: Education-Stratified Adjusted Associations of Phenol Exposures with Categorical Birth Outcomes,
Some College or more Only**Models adjusted for maternal race/ethnicity and parity, and child sex. Blank cells reflect model non-convergence.

Outcome: Preterm

|  | **Across Pregnancy** | | **Trimester 1** | | **Trimester 2** | | **Trimester 3** | |
| --- | --- | --- | --- | --- | --- | --- | --- | --- |
| **Phenol Name** | **N** | **OR (95% CI)** | **N** | **OR (95% CI)** | **N** | **OR (95% CI)** | **N** | **OR (95% CI)** |
| Sum Bisphenols | 1370 | 1.3 (0.71, 2.37) | 511 | 1.18 (0.46, 3.06) | 1271 | 1.22 (0.71, 2.11) | 527 | 0.75 (0.3, 1.9) |
| Bisphenol A | 2009 | 0.89 (0.58, 1.36) | 655 | 0.7 (0.36, 1.35) | 1621 | 1.14 (0.75, 1.72) | 829 | 0.73 (0.42, 1.27) |
| Bisphenol F | 1405 | 1.41 (0.97, 2.04) | 539 | 1.53 (0.79, 2.96) | 1283 | 0.98 (0.64, 1.51) | 531 | 1.06 (0.5, 2.29) |
| Bisphenol S | 1697 | 1.08 (0.69, 1.7) | 522 | 0.98 (0.51, 1.88) | 1411 | 1.18 (0.75, 1.83) | 707 | 0.82 (0.43, 1.57) |
| 2,4-Dichlorophenol | 1115 | 0.88 (0.52, 1.51) |  |  | 783 | 0.91 (0.46, 1.82) | 405 | 1.3 (0.61, 2.75) |
| 2,5-Dichlorophenol | 1115 | 1.18 (0.84, 1.64) |  |  | 785 | 1.24 (0.81, 1.9) | 405 | 1.36 (0.82, 2.26) |
| Benzophenone-3 | 1475 | 0.85 (0.65, 1.12) | 232 | 0.71 (0.35, 1.43) | 1134 | 0.91 (0.68, 1.23) | 427 | 0.67 (0.38, 1.17) |
| Triclosan | 1543 | 1.06 (0.83, 1.35) | 279 | 0.88 (0.53, 1.47) | 1144 | 1.06 (0.82, 1.38) | 432 | 1.45 (0.88, 2.41) |
| Butyl paraben | 1216 | 0.95 (0.69, 1.31) | 88 | 0.85 (0.13, 5.53) | 828 | 0.92 (0.63, 1.32) | 429 | 1.34 (0.8, 2.26) |
| Ethyl paraben | 1164 | 1.2 (0.87, 1.65) | 119 | 0.69 (0.18, 2.74) | 835 | 1.16 (0.81, 1.65) | 341 | 1.3 (0.65, 2.6) |
| Methyl Paraben | 1266 | 0.87 (0.61, 1.25) | 121 | 1.32 (0.35, 4.92) | 838 | 0.94 (0.63, 1.39) | 441 | 0.87 (0.47, 1.64) |
| Propyl paraben | 1260 | 0.94 (0.7, 1.25) | 119 | 0.78 (0.27, 2.24) | 837 | 1.06 (0.77, 1.44) | 438 | 0.84 (0.51, 1.4) |

Outcome: Small for Gestational Age

|  | **Across Pregnancy** | | **Trimester 1** | | **Trimester 2** | | **Trimester 3** | |
| --- | --- | --- | --- | --- | --- | --- | --- | --- |
| **Phenol Name** | **N** | **OR (95% CI)** | **N** | **OR (95% CI)** | **N** | **OR (95% CI)** | **N** | **OR (95% CI)** |
| Sum Bisphenols | 1370 | 0.99 (0.58, 1.69) | 511 | 0.98 (0.51, 1.89) | 1271 | 0.78 (0.48, 1.28) | 527 | 1.15 (0.61, 2.2) |
| Bisphenol A | 2006 | 1 (0.7, 1.42) | 654 | 0.84 (0.52, 1.35) | 1620 | 0.9 (0.64, 1.27) | 827 | 1.26 (0.82, 1.91) |
| Bisphenol F | 1405 | 1.05 (0.74, 1.5) | 539 | 1.38 (0.81, 2.36) | 1283 | 0.9 (0.61, 1.32) | 531 | 0.96 (0.56, 1.64) |
| Bisphenol S | 1697 | 0.98 (0.68, 1.41) | 522 | 1.1 (0.71, 1.71) | 1411 | 0.71 (0.49, 1.03) | 707 | **1.49 (1, 2.22)** |
| 2,4-Dichlorophenol | 1113 | 0.91 (0.58, 1.43) | 41 | 0.11 (0, 6.14) | 783 | 0.74 (0.39, 1.38) | 403 | 1.2 (0.66, 2.18) |
| 2,5-Dichlorophenol | 1113 | 0.93 (0.7, 1.25) | 40 | 0.3 (0.02, 4.93) | 785 | 1.11 (0.76, 1.62) | 403 | 1 (0.67, 1.5) |
| Benzophenone-3 | 1473 | **1.28 (1.02, 1.61)** | 232 | 1.13 (0.66, 1.94) | 1134 | 1.28 (0.98, 1.66) | 425 | 1.45 (1, 2.12) |
| Triclosan | 1541 | 1.21 (0.98, 1.49) | 279 | 1.17 (0.77, 1.79) | 1144 | 1.25 (1, 1.57) | 430 | 1.23 (0.85, 1.79) |
| Butyl paraben | 1214 | 1.16 (0.91, 1.49) | 88 | 0.83 (0.3, 2.26) | 828 | 1.23 (0.92, 1.64) | 427 | 1.1 (0.72, 1.66) |
| Ethyl paraben | 1164 | 0.97 (0.74, 1.27) | 119 | 1.34 (0.66, 2.75) | 835 | 0.89 (0.63, 1.24) | 341 | 1.34 (0.85, 2.1) |
| Methyl Paraben | 1264 | **1.54 (1.13, 2.09)** | 121 | 2.6 (0.95, 7.09) | 838 | **1.5 (1.05, 2.14)** | 439 | 1.2 (0.75, 1.93) |
| Propyl paraben | 1258 | **1.31 (1.03, 1.68)** | 119 | 1.45 (0.71, 2.94) | 837 | **1.36 (1.03, 1.81)** | 436 | 1.09 (0.74, 1.59) |

Outcome: Large for Gestational Age

|  | **Across Pregnancy** | | **Trimester 1** | | **Trimester 2** | | **Trimester 3** | |
| --- | --- | --- | --- | --- | --- | --- | --- | --- |
| **Phenol Name** | **N** | **OR (95% CI)** | **N** | **OR (95% CI)** | **N** | **OR (95% CI)** | **N** | **OR (95% CI)** |
| Sum Bisphenols | 1370 | 0.94 (0.58, 1.54) | 511 | 0.95 (0.44, 2.06) | 1271 | 0.87 (0.56, 1.36) | 527 | 1.99 (0.96, 4.12) |
| Bisphenol A | 2006 | 1.18 (0.82, 1.69) | 654 | 1.1 (0.64, 1.89) | 1620 | 0.93 (0.67, 1.3) | 827 | **1.89 (1.13, 3.16)** |
| Bisphenol F | 1405 | 0.93 (0.67, 1.28) | 539 | 1.02 (0.56, 1.89) | 1283 | 0.95 (0.68, 1.34) | 531 | 1.47 (0.83, 2.6) |
| Bisphenol S | 1697 | 1.07 (0.74, 1.53) | 522 | 0.89 (0.52, 1.52) | 1411 | 0.96 (0.67, 1.36) | 707 | 0.89 (0.53, 1.49) |
| 2,4-Dichlorophenol | 1113 | 1.35 (0.9, 2.02) | 41 | 0.6 (0.06, 6.33) | 783 | **1.73 (1.06, 2.82)** | 403 | 1.96 (0.98, 3.92) |
| 2,5-Dichlorophenol | 1113 | 1.18 (0.9, 1.54) | 40 | 0.69 (0.08, 5.91) | 785 | 1.29 (0.93, 1.77) | 403 | 1.5 (0.95, 2.38) |
| Benzophenone-3 | 1473 | **0.8 (0.64, 0.99)** | 232 | 1.07 (0.58, 1.99) | 1134 | 0.87 (0.69, 1.1) | 425 | 0.88 (0.55, 1.4) |
| Triclosan | 1541 | 1.01 (0.83, 1.23) | 279 | **0.54 (0.33, 0.88)** | 1144 | 1.07 (0.87, 1.31) | 430 | **1.83 (1.18, 2.83)** |
| Butyl paraben | 1214 | 0.9 (0.7, 1.15) | 88 | **3.91 (1.28, 11.97)** | 828 | 0.89 (0.67, 1.16) | 427 | 0.92 (0.56, 1.53) |
| Ethyl paraben | 1164 | 0.96 (0.75, 1.24) | 119 | 1.36 (0.69, 2.65) | 835 | 0.93 (0.7, 1.23) | 341 | 1.24 (0.67, 2.29) |
| Methyl Paraben | 1264 | **0.72 (0.55, 0.95)** | 121 | 2.03 (0.82, 5.06) | 838 | **0.64 (0.48, 0.86)** | 439 | 0.76 (0.44, 1.31) |
| Propyl paraben | 1258 | 0.82 (0.66, 1.02) | 119 | 1.68 (0.81, 3.46) | 837 | **0.73 (0.58, 0.93)** | 436 | 0.9 (0.58, 1.41) |

Outcome: Low Birthweight

|  | **Across Pregnancy** | | **Trimester 1** | | **Trimester 2** | | **Trimester 3** | |
| --- | --- | --- | --- | --- | --- | --- | --- | --- |
| **Phenol Name** | **N** | **OR (95% CI)** | **N** | **OR (95% CI)** | **N** | **OR (95% CI)** | **N** | **OR (95% CI)** |
| Sum Bisphenols | 1370 | 1.37 (0.66, 2.84) | 511 | 2.15 (0.85, 5.43) | 1271 | 1.03 (0.53, 2.02) | 527 | 1.19 (0.48, 2.99) |
| Bisphenol A | 2009 | 0.96 (0.57, 1.6) | 655 | 0.82 (0.42, 1.6) | 1621 | 1.03 (0.64, 1.67) | 829 | 1.05 (0.55, 1.99) |
| Bisphenol F | 1405 | **1.6 (1.01, 2.53)** | 539 | **3.28 (1.59, 6.73)** | 1283 | 0.9 (0.52, 1.55) | 531 | 0.96 (0.45, 2.05) |
| Bisphenol S | 1697 | 0.76 (0.44, 1.31) | 522 | 1.47 (0.78, 2.8) | 1411 | 0.7 (0.41, 1.19) | 707 | 1.7 (0.93, 3.11) |
| 2,4-Dichlorophenol | 1115 | 0.68 (0.33, 1.39) | 41 | 0.03 (0, 10.3) | 783 | 0.73 (0.3, 1.79) | 405 | 1.35 (0.57, 3.2) |
| 2,5-Dichlorophenol | 1115 | 0.81 (0.52, 1.26) | 40 | 0.04 (0, 4.51) | 785 | 1.05 (0.62, 1.78) | 405 | 1 (0.52, 1.89) |
| Benzophenone-3 | 1475 | 0.83 (0.59, 1.16) | 232 | 0.69 (0.35, 1.36) | 1134 | 0.83 (0.57, 1.21) | 427 | 1.01 (0.56, 1.83) |
| Triclosan | 1543 | 1.17 (0.87, 1.59) | 279 | 1.18 (0.72, 1.96) | 1144 | 1.15 (0.84, 1.57) | 432 | 1.7 (0.94, 3.08) |
| Butyl paraben | 1216 | 1.23 (0.84, 1.79) | 88 | 1.3 (0.19, 8.77) | 828 | 1.07 (0.7, 1.63) | 429 | 1.5 (0.85, 2.63) |
| Ethyl paraben | 1164 | 0.98 (0.65, 1.48) | 119 | 0.95 (0.34, 2.69) | 835 | 0.83 (0.5, 1.38) |  |  |
| Methyl Paraben | 1266 | 1.35 (0.85, 2.15) | 121 | 1.86 (0.57, 6.14) | 838 | 1.08 (0.64, 1.83) | 441 | 1.68 (0.8, 3.56) |
| Propyl paraben | 1260 | 1.07 (0.74, 1.55) | 119 | 0.69 (0.29, 1.67) | 837 | 1.15 (0.75, 1.75) | 438 | 1.2 (0.66, 2.16) |

Outcome: Low Birthweight (Preterms only)

|  | **Across Pregnancy** | | **Trimester 1** | | **Trimester 2** | | **Trimester 3** | |
| --- | --- | --- | --- | --- | --- | --- | --- | --- |
| **Phenol Name** | **N** | **OR (95% CI)** | **N** | **OR (95% CI)** | **N** | **OR (95% CI)** | **N** | **OR (95% CI)** |
| Sum Bisphenols | 96 | 0.6 (0.16, 2.34) | 28 | 0.15 (0.01, 4.29) | 89 | 0.59 (0.16, 2.09) | 29 | 1.61 (0.08, 31.55) |
| Bisphenol A | 136 | 0.56 (0.22, 1.42) | 44 | 0.19 (0.02, 1.53) | 112 | 0.46 (0.17, 1.21) | 48 | 1.94 (0.31, 12.04) |
| Bisphenol F | 100 | 1.25 (0.49, 3.14) | 31 | 0.61 (0.06, 5.89) | 90 | 0.87 (0.32, 2.38) | 29 | 3.23 (0.17, 61.3) |
| Bisphenol S | 107 | 0.61 (0.2, 1.85) | 28 | 1.32 (0.12, 14.3) | 94 | 0.73 (0.28, 1.94) | 35 | 0.45 (0.06, 3.2) |
| 2,4-Dichlorophenol | 71 | 0.51 (0.09, 2.91) |  |  | 53 | 0.23 (0.02, 2.34) | 24 | 0.19 (0, 9.52) |
| 2,5-Dichlorophenol | 71 | 0.46 (0.12, 1.83) |  |  | 53 | 0.37 (0.07, 2.05) | 24 | 0.03 (0, 18.99) |
| Benzophenone-3 | 96 | 0.89 (0.38, 2.09) | 18 | 0 (0, 5.94) | 78 | 0.78 (0.31, 1.97) | 24 | 0.61 (0.01, 43.29) |
| Triclosan | 101 | 0.85 (0.46, 1.57) | 21 | 0.08 (0, 62.86) |  |  | 24 | 0.12 (0, 4.56) |
| Butyl paraben | 75 | 2.56 (0.9, 7.29) |  |  | 55 | 2.28 (0.56, 9.28) | 24 | 147.15 (0.02, 1227486.43) |
| Ethyl paraben | 69 | 0.87 (0.36, 2.07) |  |  | 56 | 1.08 (0.42, 2.77) |  |  |
| Methyl Paraben | 79 | 1.88 (0.63, 5.63) |  |  | 57 | 1.38 (0.41, 4.66) | 24 | 199.18 (0.3, 131850.37) |
| Propyl paraben | 79 | 0.85 (0.37, 1.97) |  |  | 57 | 1.01 (0.38, 2.65) | 24 | 3.63 (0.36, 36.25) |

Outcome: Low Birthweight (Terms only)

|  | **Across Pregnancy** | | **Trimester 1** | | **Trimester 2** | | **Trimester 3** | |
| --- | --- | --- | --- | --- | --- | --- | --- | --- |
| **Phenol Name** | **N** | **OR (95% CI)** | **N** | **OR (95% CI)** | **N** | **OR (95% CI)** | **N** | **OR (95% CI)** |
| Sum Bisphenols | 1274 | 2.15 (0.72, 6.39) | 483 | **4.17 (1.29, 13.55)** | 1182 | 1 (0.34, 2.93) | 498 | 1.35 (0.37, 4.95) |
| Bisphenol A | 1873 | 1.23 (0.56, 2.72) | 611 | 1.72 (0.66, 4.49) | 1509 | 1.16 (0.54, 2.48) | 781 | 0.89 (0.37, 2.16) |
| Bisphenol F | 1305 | **2.2 (1.1, 4.4)** | 508 | **4.01 (1.49, 10.75)** | 1193 | 1.19 (0.52, 2.71) | 502 | 1.07 (0.37, 3.12) |
| Bisphenol S | 1590 | 0.71 (0.32, 1.6) | 494 | 2.25 (0.9, 5.62) | 1317 | 0.47 (0.21, 1.05) | 672 | 2.03 (0.94, 4.42) |
| 2,4-Dichlorophenol | 1044 | 0.46 (0.16, 1.34) |  |  | 730 | 0.5 (0.12, 2.11) |  |  |
| 2,5-Dichlorophenol | 1044 | 0.77 (0.41, 1.43) | 39 | 0.09 (0, 34.51) | 732 | 1.19 (0.58, 2.45) |  |  |
| Benzophenone-3 | 1379 | 1.06 (0.66, 1.7) | 214 | 1.03 (0.4, 2.65) | 1056 | 0.97 (0.56, 1.71) |  |  |
| Triclosan | 1442 | 1.3 (0.83, 2.03) | 258 | 1.79 (0.76, 4.2) | 1064 | 1.11 (0.68, 1.81) |  |  |
| Butyl paraben | 1141 | 1.3 (0.78, 2.17) |  |  | 773 | 1.23 (0.68, 2.22) |  |  |
| Ethyl paraben | 1095 | 0.81 (0.44, 1.48) | 114 | 1.07 (0.22, 5.29) | 779 | 0.45 (0.2, 1.05) |  |  |
| Methyl Paraben | 1187 | 1.81 (0.93, 3.53) | 116 | 1.66 (0.22, 12.22) | 781 | 1.28 (0.58, 2.83) |  |  |
| Propyl paraben | 1181 | 1.37 (0.81, 2.33) | 114 | 0.39 (0.07, 2.15) | 780 | 1.42 (0.76, 2.67) |  |  |

**Supplement Table 4a1: Parity-Stratified Adjusted Associations of Phenol Exposures with Birth Outcomes (Continuously Measured), Parity=0 Only**Models adjusted for maternal race/ethnicity and education, and child sex. BW for GA model removes parity from covariates.

Outcome: Gestational Age

|  | **Across Pregnancy** | | **Trimester 1** | | **Trimester 2** | | **Trimester 3** | |
| --- | --- | --- | --- | --- | --- | --- | --- | --- |
| **Phenol Name** | **N** | **Beta (95% CI)** | **N** | **Beta (95% CI)** | **N** | **Beta (95% CI)** | **N** | **Beta (95% CI)** |
| Sum Bisphenols | 763 | -0.107 (-0.461, 0.247) | 312 | -0.233 (-0.696, 0.229) | 714 | -0.128 (-0.457, 0.201) | 318 | 0.089 (-0.327, 0.506) |
| Bisphenol A | 1275 | 0.048 (-0.171, 0.267) | 406 | 0.032 (-0.314, 0.377) | 903 | -0.059 (-0.284, 0.165) | 647 | 0.09 (-0.149, 0.33) |
| Bisphenol F | 790 | 0.019 (-0.214, 0.251) | 333 | -0.324 (-0.717, 0.069) | 720 | 0.242 (-0.018, 0.502) | 321 | 0.187 (-0.147, 0.52) |
| Bisphenol S | 994 | **-0.319 (-0.562, -0.077)** | 321 | -0.199 (-0.515, 0.117) | 796 | -0.164 (-0.418, 0.091) | 460 | **-0.304 (-0.558, -0.049)** |
| 2,4-Dichlorophenol | 756 | 0.134 (-0.08, 0.348) | 26 | 0 (-1.522, 1.523) | 415 | 0.087 (-0.271, 0.446) | 372 | 0.032 (-0.24, 0.303) |
| 2,5-Dichlorophenol | 754 | 0.113 (-0.035, 0.261) | 24 | 0.687 (-0.732, 2.106) | 416 | 0.011 (-0.22, 0.241) | 371 | 0.113 (-0.081, 0.306) |
| Benzophenone-3 | 910 | 0.122 (-0.017, 0.262) | 111 | 0.446 (-0.03, 0.923) | 564 | **0.177 (0.002, 0.352)** | 384 | 0.047 (-0.153, 0.247) |
| Triclosan | 957 | 0 (-0.125, 0.124) | 148 | -0.122 (-0.452, 0.207) | 569 | 0.017 (-0.138, 0.173) | 386 | -0.113 (-0.307, 0.082) |
| Butyl paraben | 823 | 0.133 (-0.009, 0.274) | 59 | -0.22 (-0.741, 0.301) | 446 | 0.149 (-0.031, 0.329) | 384 | 0.114 (-0.107, 0.335) |
| Ethyl paraben | 691 | 0.03 (-0.116, 0.177) | 85 | -0.009 (-0.412, 0.394) | 446 | 0.065 (-0.12, 0.249) | 228 | 0.032 (-0.213, 0.277) |
| Methyl Paraben | 859 | 0.141 (-0.015, 0.297) | 85 | 0.15 (-0.289, 0.589) | 447 | 0.181 (-0.017, 0.38) | 395 | -0.03 (-0.258, 0.199) |
| Propyl paraben | 855 | **0.14 (0.012, 0.267)** | 83 | 0.169 (-0.177, 0.515) | 447 | 0.122 (-0.037, 0.281) | 393 | 0.064 (-0.128, 0.257) |

Outcome: Birth Length

|  | **Across Pregnancy** | | **Trimester 1** | | **Trimester 2** | | **Trimester 3** | |
| --- | --- | --- | --- | --- | --- | --- | --- | --- |
| **Phenol Name** | **N** | **Beta (95% CI)** | **N** | **Beta (95% CI)** | **N** | **Beta (95% CI)** | **N** | **Beta (95% CI)** |
| Sum Bisphenols | 738 | -0.305 (-0.857, 0.248) | 300 | -0.176 (-0.901, 0.549) | 689 | -0.256 (-0.769, 0.257) | 304 | -0.192 (-0.875, 0.49) |
| Bisphenol A | 1199 | 0.129 (-0.217, 0.475) | 364 | 0.1 (-0.448, 0.649) | 857 | -0.099 (-0.45, 0.252) | 602 | 0.212 (-0.185, 0.61) |
| Bisphenol F | 761 | **-0.407 (-0.775, -0.038)** | 320 | **-0.89 (-1.523, -0.257)** | 694 | -0.102 (-0.507, 0.302) | 305 | 0.028 (-0.523, 0.578) |
| Bisphenol S | 966 | 0.271 (-0.126, 0.668) | 309 | -0.054 (-0.581, 0.473) | 770 | 0.127 (-0.277, 0.531) | 444 | -0.236 (-0.669, 0.197) |
| 2,4-Dichlorophenol | 731 | 0.224 (-0.133, 0.581) | 26 | 2.747 (-0.888, 6.383) | 407 | 0.097 (-0.502, 0.695) | 354 | 0.121 (-0.315, 0.556) |
| 2,5-Dichlorophenol | 729 | **0.255 (0.009, 0.502)** | 24 | 2.097 (-1.444, 5.638) | 408 | 0.138 (-0.25, 0.526) | 353 | 0.177 (-0.131, 0.485) |
| Benzophenone-3 | 883 | -0.199 (-0.425, 0.028) | 111 | 0.392 (-0.416, 1.201) | 554 | -0.186 (-0.468, 0.097) | 366 | -0.255 (-0.579, 0.069) |
| Triclosan | 860 | -0.134 (-0.344, 0.075) | 99 | -0.2 (-0.825, 0.425) | 545 | **-0.262 (-0.515, -0.01)** | 361 | 0.044 (-0.283, 0.371) |
| Butyl paraben | 744 | -0.022 (-0.267, 0.222) | 24 | -0.538 (-1.507, 0.43) | 423 | -0.274 (-0.582, 0.034) | 362 | 0.151 (-0.213, 0.515) |
| Ethyl paraben | 607 | -0.004 (-0.275, 0.268) | 35 | 0.814 (-0.779, 2.407) | 423 | -0.185 (-0.501, 0.131) | 216 | 0.009 (-0.446, 0.463) |
| Methyl Paraben | 760 | -0.235 (-0.513, 0.043) | 35 | -0.272 (-1.805, 1.26) | 424 | **-0.427 (-0.761, -0.094)** | 368 | -0.205 (-0.592, 0.182) |
| Propyl paraben | 759 | -0.14 (-0.367, 0.087) | 34 | **1.211 (0.055, 2.367)** | 424 | **-0.293 (-0.563, -0.024)** | 368 | -0.169 (-0.49, 0.152) |

Outcome: Birthweight

|  | **Across Pregnancy** | | **Trimester 1** | | **Trimester 2** | | **Trimester 3** | |
| --- | --- | --- | --- | --- | --- | --- | --- | --- |
| **Phenol Name** | **N** | **Beta (95% CI)** | **N** | **Beta (95% CI)** | **N** | **Beta (95% CI)** | **N** | **Beta (95% CI)** |
| Sum Bisphenols | 763 | -46.361 (-149.415, 56.694) | 312 | -3.667 (-135.785, 128.451) | 714 | 3.27 (-91.312, 97.851) | 318 | -0.027 (-134.636, 134.582) |
| Bisphenol A | 1275 | 48.739 (-14.84, 112.317) | 406 | 61.019 (-37.95, 159.987) | 903 | 25.922 (-38.882, 90.727) | 647 | 42.677 (-30.986, 116.339) |
| Bisphenol F | 790 | -57.127 (-124.566, 10.313) | 333 | -105.99 (-217.701, 5.721) | 720 | 7.643 (-67.239, 82.524) | 321 | 30.745 (-76.775, 138.264) |
| Bisphenol S | 994 | -20.677 (-91.817, 50.463) | 321 | -48.775 (-139.964, 42.414) | 796 | 39.258 (-34.209, 112.726) | 460 | **-97.607 (-177.602, -17.613)** |
| 2,4-Dichlorophenol | 756 | 40.406 (-23.714, 104.526) | 26 | 107.942 (-372.073, 587.957) | 415 | 61.758 (-49.227, 172.743) | 372 | 13.317 (-66.743, 93.377) |
| 2,5-Dichlorophenol | 754 | 28.955 (-15.342, 73.251) | 24 | 176.753 (-279.458, 632.964) | 416 | -3.641 (-75.282, 67.999) | 371 | 25.59 (-31.222, 82.402) |
| Benzophenone-3 | 910 | -23.263 (-64.269, 17.744) | 111 | 59.218 (-80.622, 199.058) | 564 | -12.468 (-65.112, 40.175) | 384 | -36.526 (-95.792, 22.741) |
| Triclosan | 957 | -28.825 (-65.662, 8.011) | 148 | -77.874 (-172.038, 16.29) | 569 | -31.224 (-77.734, 15.287) | 386 | -38.812 (-96.601, 18.977) |
| Butyl paraben | 823 | 8.654 (-33.794, 51.101) | 59 | 83.19 (-74.917, 241.298) | 446 | -8.548 (-64.901, 47.805) | 384 | 9.698 (-55.493, 74.888) |
| Ethyl paraben | 691 | -22.587 (-67.617, 22.443) | 85 | 103.547 (-16.303, 223.397) | 446 | -29.627 (-87.263, 28.01) | 228 | -74.245 (-151.042, 2.553) |
| Methyl Paraben | 859 | -10.391 (-57.461, 36.679) | 85 | 79.135 (-52.916, 211.185) | 447 | -42.617 (-104.666, 19.432) | 395 | -32.187 (-99.815, 35.44) |
| Propyl paraben | 855 | 3.381 (-35.002, 41.764) | 83 | 93.052 (-11.171, 197.276) | 447 | -26.762 (-76.582, 23.057) | 393 | 4.834 (-52.095, 61.763) |

Outcome: Birthweight for Gestational Age Z-Score

|  | **Across Pregnancy** | | **Trimester 1** | | **Trimester 2** | | **Trimester 3** | |
| --- | --- | --- | --- | --- | --- | --- | --- | --- |
| **Phenol Name** | **N** | **Beta (95% CI)** | **N** | **Beta (95% CI)** | **N** | **Beta (95% CI)** | **N** | **Beta (95% CI)** |
| Sum Bisphenols | 763 | -0.046 (-0.263, 0.172) | 312 | 0.072 (-0.206, 0.35) | 714 | 0.081 (-0.117, 0.28) | 318 | -0.017 (-0.281, 0.246) |
| Bisphenol A | 1271 | 0.105 (-0.036, 0.247) | 405 | 0.115 (-0.094, 0.325) | 902 | 0.101 (-0.037, 0.239) | 644 | 0.074 (-0.09, 0.239) |
| Bisphenol F | 790 | -0.141 (-0.284, 0.002) | 333 | -0.113 (-0.353, 0.127) | 720 | -0.097 (-0.254, 0.06) | 321 | 0.015 (-0.194, 0.224) |
| Bisphenol S | 994 | 0.09 (-0.064, 0.244) | 321 | -0.083 (-0.277, 0.112) | 796 | **0.19 (0.035, 0.346)** | 460 | -0.126 (-0.29, 0.038) |
| 2,4-Dichlorophenol | 753 | 0.058 (-0.093, 0.21) | 26 | 0.386 (-0.827, 1.6) | 415 | 0.102 (-0.144, 0.348) | 369 | 0.051 (-0.142, 0.244) |
| 2,5-Dichlorophenol | 751 | 0.041 (-0.064, 0.145) | 24 | -0.138 (-1.191, 0.915) | 416 | -0.013 (-0.172, 0.146) | 368 | 0.042 (-0.094, 0.179) |
| Benzophenone-3 | 907 | **-0.127 (-0.221, -0.033)** | 111 | -0.074 (-0.36, 0.213) | 564 | **-0.117 (-0.23, -0.004)** | 381 | -0.129 (-0.272, 0.013) |
| Triclosan | 954 | -0.077 (-0.161, 0.007) | 148 | -0.138 (-0.322, 0.047) | 569 | -0.091 (-0.191, 0.009) | 383 | -0.051 (-0.19, 0.088) |
| Butyl paraben | 820 | -0.04 (-0.14, 0.06) | 59 | 0.276 (-0.113, 0.666) | 446 | -0.096 (-0.22, 0.029) | 381 | -0.009 (-0.165, 0.148) |
| Ethyl paraben | 691 | -0.076 (-0.179, 0.026) | 85 | 0.261 (-0.028, 0.549) | 446 | -0.093 (-0.22, 0.035) | 228 | **-0.225 (-0.39, -0.061)** |
| Methyl Paraben | 856 | -0.106 (-0.217, 0.004) | 85 | 0.103 (-0.217, 0.423) | 447 | **-0.189 (-0.325, -0.053)** | 392 | -0.081 (-0.243, 0.082) |
| Propyl paraben | 852 | -0.064 (-0.155, 0.026) | 83 | 0.156 (-0.098, 0.41) | 447 | **-0.121 (-0.231, -0.011)** | 390 | -0.032 (-0.169, 0.104) |

**Supplement Table 4b1: Parity-Stratified Adjusted Associations of Phenol Exposures with Categorical Birth Outcomes, Parity=0 Only**Models adjusted for maternal race/ethnicity and education, and child sex. Blank cells reflect model non-convergence.

Outcome: Preterm

|  | **Across Pregnancy** | | **Trimester 1** | | **Trimester 2** | | **Trimester 3** | |
| --- | --- | --- | --- | --- | --- | --- | --- | --- |
| **Phenol Name** | **N** | **OR (95% CI)** | **N** | **OR (95% CI)** | **N** | **OR (95% CI)** | **N** | **OR (95% CI)** |
| Sum Bisphenols | 763 | 1.58 (0.75, 3.34) |  |  | 714 | 1.05 (0.52, 2.13) | 318 | 1.62 (0.52, 5.07) |
| Bisphenol A | 1275 | 0.91 (0.53, 1.56) | 406 | 0.85 (0.31, 2.31) | 903 | 1.07 (0.64, 1.8) | 647 | 1.08 (0.52, 2.23) |
| Bisphenol F | 790 | 1.31 (0.8, 2.15) | 333 | 2.96 (0.99, 8.86) | 720 | 0.65 (0.35, 1.22) | 321 | 1.18 (0.47, 2.96) |
| Bisphenol S | 994 | 1.69 (0.94, 3.04) |  |  | 796 | 1.19 (0.67, 2.13) | 460 | 1.49 (0.66, 3.34) |
| 2,4-Dichlorophenol | 756 | 0.72 (0.39, 1.31) |  |  | 415 | 0.8 (0.33, 1.96) | 372 | 1.08 (0.51, 2.28) |
| 2,5-Dichlorophenol | 754 | 1 (0.67, 1.47) |  |  | 416 | 1.19 (0.68, 2.08) | 371 | 1.07 (0.62, 1.83) |
| Benzophenone-3 | 910 | 0.75 (0.53, 1.06) | 111 | 0.39 (0.09, 1.61) | 564 | 0.69 (0.45, 1.03) | 384 | 0.92 (0.51, 1.67) |
| Triclosan | 957 | 1.02 (0.74, 1.39) | 148 | 1.49 (0.73, 3.06) | 569 | 0.88 (0.61, 1.27) | 386 | 1.52 (0.85, 2.7) |
| Butyl paraben | 823 | 0.85 (0.57, 1.25) | 59 | 0.89 (0.12, 6.4) | 446 | 0.78 (0.48, 1.27) | 384 | 1.05 (0.56, 1.97) |
| Ethyl paraben | 691 | 0.88 (0.59, 1.31) | 85 | 1.1 (0.29, 4.24) | 446 | 0.88 (0.55, 1.4) | 228 | 1.07 (0.48, 2.37) |
| Methyl Paraben | 859 | **0.65 (0.43, 0.99)** | 85 | 1.06 (0.29, 3.87) | 447 | **0.6 (0.37, 1)** | 395 | 0.8 (0.4, 1.57) |
| Propyl paraben | 855 | **0.66 (0.47, 0.93)** | 83 | 1.04 (0.38, 2.84) | 447 | **0.64 (0.43, 0.96)** | 393 | 0.66 (0.37, 1.18) |

Outcome: Small for Gestational Age

|  | **Across Pregnancy** | | **Trimester 1** | | **Trimester 2** | | **Trimester 3** | |
| --- | --- | --- | --- | --- | --- | --- | --- | --- |
| **Phenol Name** | **N** | **OR (95% CI)** | **N** | **OR (95% CI)** | **N** | **OR (95% CI)** | **N** | **OR (95% CI)** |
| Sum Bisphenols | 763 | 1.1 (0.57, 2.1) | 312 | 0.96 (0.43, 2.13) | 714 | 0.77 (0.42, 1.41) | 318 | 1.3 (0.61, 2.75) |
| Bisphenol A | 1271 | 0.82 (0.56, 1.21) | 405 | 0.79 (0.45, 1.4) | 902 | 0.77 (0.52, 1.13) | 644 | 1.05 (0.68, 1.64) |
| Bisphenol F | 790 | 1.32 (0.88, 1.99) | 333 | 1.39 (0.75, 2.59) | 720 | 1.15 (0.72, 1.83) | 321 | 0.85 (0.46, 1.57) |
| Bisphenol S | 994 | 0.91 (0.58, 1.42) | 321 | 1.17 (0.69, 1.96) | 796 | 0.66 (0.42, 1.04) | 460 | 1.49 (0.93, 2.41) |
| 2,4-Dichlorophenol | 753 | 0.9 (0.6, 1.36) | 26 | 0.06 (0, 11.26) | 415 | 0.74 (0.33, 1.66) | 369 | 0.99 (0.62, 1.59) |
| 2,5-Dichlorophenol | 751 | 0.9 (0.68, 1.19) | 24 | 0.16 (0, 8.63) | 416 | 1.01 (0.62, 1.67) | 368 | 0.94 (0.67, 1.32) |
| Benzophenone-3 | 907 | 1.3 (1, 1.69) | 111 | 1.25 (0.61, 2.58) | 564 | 1.19 (0.84, 1.68) | 381 | **1.49 (1.03, 2.15)** |
| Triclosan | 954 | 1.2 (0.95, 1.52) | 148 | 1.11 (0.67, 1.86) | 569 | 1.26 (0.93, 1.7) | 383 | 1.16 (0.81, 1.66) |
| Butyl paraben | 820 | 1.21 (0.92, 1.59) | 59 | 0.65 (0.19, 2.2) | 446 | 1.36 (0.95, 1.94) | 381 | 1 (0.67, 1.49) |
| Ethyl paraben | 691 | 1.01 (0.75, 1.36) | 85 | 0.61 (0.26, 1.41) | 446 | 0.74 (0.48, 1.14) | 228 | **1.79 (1.11, 2.87)** |
| Methyl Paraben | 856 | 1.28 (0.94, 1.75) | 85 | 1.23 (0.53, 2.87) | 447 | 1.25 (0.81, 1.93) | 392 | 1.16 (0.75, 1.79) |
| Propyl paraben | 852 | 1.07 (0.83, 1.37) | 83 | 0.79 (0.41, 1.53) | 447 | 1.15 (0.81, 1.63) | 390 | 0.97 (0.68, 1.38) |

Outcome: Large for Gestational Age

|  | **Across Pregnancy** | | **Trimester 1** | | **Trimester 2** | | **Trimester 3** | |
| --- | --- | --- | --- | --- | --- | --- | --- | --- |
| **Phenol Name** | **N** | **OR (95% CI)** | **N** | **OR (95% CI)** | **N** | **OR (95% CI)** | **N** | **OR (95% CI)** |
| Sum Bisphenols | 763 | 1.1 (0.55, 2.23) | 312 | 0.95 (0.34, 2.62) | 714 | 0.91 (0.48, 1.74) | 318 | 1.18 (0.4, 3.49) |
| Bisphenol A | 1271 | 1.36 (0.83, 2.21) | 405 | 1.26 (0.63, 2.55) | 902 | 1 (0.63, 1.59) | 644 | 1.24 (0.66, 2.35) |
| Bisphenol F | 790 | 0.8 (0.49, 1.29) | 333 | 1.02 (0.43, 2.4) | 720 | 0.77 (0.45, 1.32) | 321 | 1.31 (0.54, 3.15) |
| Bisphenol S | 994 | 1.23 (0.71, 2.13) | 321 | 0.6 (0.29, 1.26) | 796 | 1.3 (0.76, 2.22) | 460 | 0.5 (0.22, 1.15) |
| 2,4-Dichlorophenol | 753 | 1.21 (0.74, 1.98) | 26 | 0.3 (0.01, 15.97) | 415 | 1.39 (0.61, 3.17) | 369 | 1.03 (0.53, 2) |
| 2,5-Dichlorophenol | 751 | 1.19 (0.85, 1.68) | 24 | 0.12 (0, 8.17) |  |  | 368 | 1.08 (0.66, 1.75) |
| Benzophenone-3 | 907 | **0.68 (0.5, 0.93)** | 111 | 1.36 (0.56, 3.26) | 564 | 0.71 (0.49, 1.03) | 381 | 0.76 (0.44, 1.33) |
| Triclosan | 954 | 0.87 (0.65, 1.15) | 148 | 0.5 (0.25, 1.02) | 569 | 0.9 (0.64, 1.26) | 383 | 1.26 (0.76, 2.08) |
| Butyl paraben | 820 | 0.94 (0.67, 1.32) | 59 | 1.95 (0.53, 7.2) |  |  | 381 | 1.02 (0.57, 1.83) |
| Ethyl paraben | 691 | 0.96 (0.67, 1.38) | 85 | 1.92 (0.76, 4.87) |  |  | 228 | 1.46 (0.61, 3.5) |
| Methyl Paraben | 856 | 0.9 (0.63, 1.29) | 85 | 2.19 (0.71, 6.76) |  |  | 392 | 0.99 (0.52, 1.86) |
| Propyl paraben | 852 | 0.86 (0.64, 1.15) | 83 | 1.5 (0.64, 3.55) |  |  | 390 | 0.99 (0.59, 1.67) |

Outcome: Low Birthweight

|  | **Across Pregnancy** | | **Trimester 1** | | **Trimester 2** | | **Trimester 3** | |
| --- | --- | --- | --- | --- | --- | --- | --- | --- |
| **Phenol Name** | **N** | **OR (95% CI)** | **N** | **OR (95% CI)** | **N** | **OR (95% CI)** | **N** | **OR (95% CI)** |
| Sum Bisphenols | 763 | 1.55 (0.68, 3.57) | 312 | 2.5 (0.87, 7.15) | 714 | 1.09 (0.5, 2.4) | 318 | 1.96 (0.72, 5.36) |
| Bisphenol A | 1275 | 0.81 (0.46, 1.44) | 406 | 0.98 (0.44, 2.18) | 903 | 0.96 (0.55, 1.67) | 647 | 1.39 (0.69, 2.8) |
| Bisphenol F | 790 | **1.81 (1.07, 3.07)** | 333 | **3.52 (1.58, 7.87)** | 720 | 0.91 (0.47, 1.74) | 321 | 1.19 (0.53, 2.7) |
| Bisphenol S | 994 | 0.9 (0.47, 1.73) | 321 | 1.48 (0.7, 3.13) | 796 | 0.95 (0.51, 1.79) | 460 | 1.82 (0.89, 3.71) |
| 2,4-Dichlorophenol | 756 | 0.5 (0.24, 1.06) | 26 | 0.03 (0, 12.34) | 415 | 0.51 (0.16, 1.68) | 372 | 0.88 (0.39, 1.98) |
| 2,5-Dichlorophenol | 754 | 0.82 (0.52, 1.3) | 24 | 0.04 (0, 5.44) | 416 | 1.11 (0.59, 2.09) | 371 | 0.98 (0.54, 1.77) |
| Benzophenone-3 | 910 | 0.68 (0.45, 1.03) | 111 | 0.61 (0.24, 1.57) |  |  | 384 | 0.81 (0.43, 1.52) |
| Triclosan | 957 | 1.18 (0.83, 1.67) | 148 | 1.85 (0.96, 3.56) |  |  | 386 | 1.51 (0.82, 2.78) |
| Butyl paraben | 823 | 1.08 (0.71, 1.65) | 59 | 0.68 (0.06, 8.09) | 446 | 0.95 (0.56, 1.61) | 384 | 1.01 (0.53, 1.92) |
| Ethyl paraben | 691 | 1.05 (0.67, 1.65) | 85 | 0.72 (0.22, 2.39) | 446 | 0.81 (0.44, 1.48) | 228 | **2.23 (1.04, 4.78)** |
| Methyl Paraben | 859 | 0.9 (0.56, 1.44) | 85 | 0.92 (0.29, 2.91) | 447 | 0.87 (0.46, 1.65) | 395 | 1.09 (0.53, 2.24) |
| Propyl paraben | 855 | 0.8 (0.54, 1.19) | 83 | 0.62 (0.24, 1.6) | 447 | 0.9 (0.53, 1.52) | 393 | 0.76 (0.42, 1.4) |

Outcome: Low Birthweight (Preterms only)

|  | **Across Pregnancy** | | **Trimester 1** | | **Trimester 2** | | **Trimester 3** | |
| --- | --- | --- | --- | --- | --- | --- | --- | --- |
| **Phenol Name** | **N** | **OR (95% CI)** | **N** | **OR (95% CI)** | **N** | **OR (95% CI)** | **N** | **OR (95% CI)** |
| Sum Bisphenols | 58 | 0.25 (0.03, 2.43) |  |  | 56 | 0.73 (0.1, 5.16) | 18 | 9.18 (0.02, 4361.49) |
| Bisphenol A | 83 | 0.3 (0.09, 1.05) | 19 | 0.62 (0.02, 16.58) | 65 | 0.62 (0.18, 2.07) | 33 | 2.61 (0.27, 25.31) |
| Bisphenol F | 61 | 0.78 (0.22, 2.71) |  |  | 56 | 0.32 (0.06, 1.85) | 18 | 24.27 (0.06, 10452.53) |
| Bisphenol S | 66 | 0.45 (0.09, 2.28) |  |  | 58 | 1.12 (0.22, 5.61) | 24 | 0.86 (0.08, 9.43) |
| 2,4-Dichlorophenol | 52 | 0.1 (0, 2.16) |  |  | 35 | 0 (0, 74.29) | 21 | 0.27 (0.01, 5.04) |
| 2,5-Dichlorophenol | 52 | 0.1 (0.01, 1.16) |  |  | 35 | 0 (0, 7.42) | 21 | 0.29 (0.03, 3.19) |
| Benzophenone-3 | 62 | 0.7 (0.18, 2.72) |  |  | 45 | 0.98 (0.19, 5.18) | 21 | 0.51 (0.02, 11.07) |
| Triclosan | 65 | 1.16 (0.46, 2.91) |  |  | 45 | 0.35 (0.08, 1.49) | 21 | 1.92 (0.16, 23.43) |
| Butyl paraben | 56 | 0.99 (0.28, 3.49) |  |  | 36 | 0.39 (0.02, 6.66) | 21 | 1.62 (0.14, 18.85) |
| Ethyl paraben | 48 | 2.12 (0.54, 8.32) |  |  | 36 | 1.38 (0.34, 5.67) |  |  |
| Methyl Paraben | 57 | 1.13 (0.33, 3.9) |  |  | 36 | 1.42 (0.25, 8.2) | 21 | 1.22 (0.04, 34.43) |
| Propyl paraben | 57 | 0.8 (0.29, 2.22) |  |  | 36 | 1.09 (0.3, 3.96) |  |  |

Outcome: Low Birthweight (Terms only)

|  | **Across Pregnancy** | | **Trimester 1** | | **Trimester 2** | | **Trimester 3** | |
| --- | --- | --- | --- | --- | --- | --- | --- | --- |
| **Phenol Name** | **N** | **OR (95% CI)** | **N** | **OR (95% CI)** | **N** | **OR (95% CI)** | **N** | **OR (95% CI)** |
| Sum Bisphenols | 705 | 2.45 (0.8, 7.5) | 301 | **3.69 (1.1, 12.43)** | 658 | 1.1 (0.35, 3.42) | 300 | 2.18 (0.56, 8.41) |
| Bisphenol A | 1192 | 1.1 (0.5, 2.42) | 387 | 1.3 (0.48, 3.51) | 838 | 0.95 (0.43, 2.07) | 614 | 1.46 (0.58, 3.67) |
| Bisphenol F | 729 | **2.6 (1.28, 5.27)** | 319 | **3.67 (1.36, 9.9)** | 664 | 1.59 (0.69, 3.65) | 303 | 1.12 (0.36, 3.46) |
| Bisphenol S | 928 | 0.77 (0.32, 1.86) | 310 | 2.16 (0.86, 5.45) | 738 | 0.65 (0.27, 1.53) | 436 | 1.77 (0.72, 4.33) |
| 2,4-Dichlorophenol | 704 | **0.36 (0.13, 0.98)** |  |  | 380 | 0.26 (0.04, 1.61) | 351 | 0.59 (0.2, 1.72) |
| 2,5-Dichlorophenol | 702 | 0.81 (0.46, 1.45) | 23 | 0.09 (0, 44.8) |  |  | 350 | 0.86 (0.41, 1.8) |
| Benzophenone-3 | 848 | 0.91 (0.54, 1.53) | 103 | 0.82 (0.27, 2.51) |  |  | 363 | 1.04 (0.49, 2.22) |
| Triclosan | 892 | 1.19 (0.75, 1.89) | 137 | 1.91 (0.79, 4.63) |  |  | 365 | 1.1 (0.52, 2.31) |
| Butyl paraben | 767 | 1.24 (0.73, 2.09) | 55 | 1.42 (0.08, 24.44) | 410 | 1.26 (0.63, 2.51) | 363 | 1.01 (0.44, 2.3) |
| Ethyl paraben | 643 | 1.03 (0.57, 1.86) | 80 | 0.64 (0.16, 2.58) | 410 | 0.52 (0.21, 1.3) | 216 | **3.81 (1.37, 10.55)** |
| Methyl Paraben | 802 | 1.13 (0.62, 2.07) | 80 | 0.99 (0.26, 3.76) | 411 | 0.99 (0.39, 2.53) | 374 | 1.56 (0.63, 3.91) |
| Propyl paraben | 798 | 0.98 (0.6, 1.61) | 78 | 0.67 (0.23, 1.95) | 411 | 1.12 (0.53, 2.38) | 372 | 1.08 (0.52, 2.26) |

**Supplement Table 4a2: Parity-Stratified Adjusted Associations of Phenol Exposures with Birth Outcomes (Continuously Measured), Parity=1 Only**Models adjusted for maternal race/ethnicity and education, and child sex. BW for GA model removes parity from covariates.

Outcome: Gestational Age

|  | **Across Pregnancy** | | **Trimester 1** | | **Trimester 2** | | **Trimester 3** | |
| --- | --- | --- | --- | --- | --- | --- | --- | --- |
| **Phenol Name** | **N** | **Beta (95% CI)** | **N** | **Beta (95% CI)** | **N** | **Beta (95% CI)** | **N** | **Beta (95% CI)** |
| Sum Bisphenols | 686 | 0.229 (-0.117, 0.576) | 329 | -0.132 (-0.582, 0.319) | 646 | -0.083 (-0.401, 0.236) | 312 | 0.062 (-0.275, 0.4) |
| Bisphenol A | 1059 | 0.162 (-0.068, 0.393) | 408 | -0.066 (-0.387, 0.255) | 821 | -0.02 (-0.255, 0.216) | 520 | -0.012 (-0.244, 0.22) |
| Bisphenol F | 700 | 0.079 (-0.161, 0.319) | 340 | 0.013 (-0.371, 0.397) | 652 | 0.018 (-0.233, 0.268) | 312 | 0.043 (-0.243, 0.329) |
| Bisphenol S | 829 | 0.061 (-0.18, 0.303) | 331 | -0.22 (-0.542, 0.103) | 709 | -0.013 (-0.246, 0.22) | 391 | 0.176 (-0.044, 0.396) |
| 2,4-Dichlorophenol | 504 | 0.106 (-0.158, 0.371) | 11 | -1.25 (-3.025, 0.526) | 296 | -0.021 (-0.379, 0.337) | 244 | 0.134 (-0.228, 0.495) |
| 2,5-Dichlorophenol | 504 | 0.124 (-0.057, 0.306) | 12 | -1.145 (-2.795, 0.505) | 296 | 0.031 (-0.208, 0.27) | 244 | 0.1 (-0.151, 0.351) |
| Benzophenone-3 | 657 | -0.082 (-0.251, 0.087) | 96 | 0.126 (-0.465, 0.716) | 447 | -0.017 (-0.216, 0.183) | 250 | 0.057 (-0.187, 0.3) |
| Triclosan | 699 | -0.024 (-0.169, 0.12) | 121 | -0.043 (-0.458, 0.371) | 455 | -0.101 (-0.269, 0.068) | 256 | 0.156 (-0.062, 0.373) |
| Butyl paraben | 552 | **0.209 (0.043, 0.374)** | 36 | 0.053 (-0.511, 0.616) | 319 | **0.225 (0.019, 0.431)** | 249 | -0.004 (-0.256, 0.248) |
| Ethyl paraben | 461 | -0.024 (-0.209, 0.161) | 47 | 0.021 (-0.486, 0.528) | 322 | -0.041 (-0.271, 0.19) | 142 | -0.035 (-0.319, 0.25) |
| Methyl Paraben | 576 | 0.084 (-0.123, 0.29) | 48 | 0.194 (-0.413, 0.801) | 325 | 0.04 (-0.213, 0.293) | 257 | -0.025 (-0.33, 0.281) |
| Propyl paraben | 574 | 0.056 (-0.101, 0.213) | 47 | 0.135 (-0.365, 0.635) | 325 | -0.04 (-0.231, 0.152) | 256 | 0.03 (-0.198, 0.258) |

Outcome: Birth Length

|  | **Across Pregnancy** | | **Trimester 1** | | **Trimester 2** | | **Trimester 3** | |
| --- | --- | --- | --- | --- | --- | --- | --- | --- |
| **Phenol Name** | **N** | **Beta (95% CI)** | **N** | **Beta (95% CI)** | **N** | **Beta (95% CI)** | **N** | **Beta (95% CI)** |
| Sum Bisphenols | 665 | 0.338 (-0.251, 0.928) | 318 | 0.077 (-0.62, 0.774) | 626 | 0.214 (-0.336, 0.764) | 301 | 0.275 (-0.306, 0.857) |
| Bisphenol A | 1004 | 0.186 (-0.224, 0.596) | 371 | 0.22 (-0.286, 0.727) | 781 | -0.046 (-0.45, 0.358) | 497 | 0.13 (-0.313, 0.573) |
| Bisphenol F | 679 | 0.173 (-0.234, 0.58) | 329 | 0.087 (-0.512, 0.686) | 632 | 0.184 (-0.247, 0.614) | 301 | 0.312 (-0.184, 0.808) |
| Bisphenol S | 808 | 0.156 (-0.27, 0.582) | 320 | -0.247 (-0.741, 0.248) | 689 | 0.311 (-0.099, 0.722) | 380 | -0.246 (-0.652, 0.159) |
| 2,4-Dichlorophenol | 490 | 0.317 (-0.197, 0.832) | 11 | -0.013 (-3.084, 3.059) | 289 | 0.255 (-0.407, 0.917) | 237 | 0.517 (-0.229, 1.263) |
| 2,5-Dichlorophenol | 490 | **0.361 (0.011, 0.71)** | 12 | -1.174 (-3.924, 1.576) | 289 | 0.224 (-0.217, 0.664) | 237 | 0.48 (-0.032, 0.992) |
| Benzophenone-3 | 642 | **-0.425 (-0.745, -0.105)** | 96 | -0.125 (-0.944, 0.695) | 439 | -0.209 (-0.575, 0.158) | 243 | -0.448 (-0.944, 0.049) |
| Triclosan | 633 | 0.087 (-0.185, 0.36) | 93 | -0.246 (-0.91, 0.418) | 432 | -0.076 (-0.371, 0.218) | 241 | 0.421 (-0.025, 0.867) |
| Butyl paraben | 504 | 0.158 (-0.192, 0.507) | 15 | **-1.238 (-2.09, -0.386)** | 303 | 0.101 (-0.309, 0.511) | 238 | 0.072 (-0.445, 0.589) |
| Ethyl paraben | 403 | 0.214 (-0.181, 0.609) | 19 | -0.172 (-1.743, 1.4) | 300 | **0.586 (0.123, 1.05)** | 134 | -0.45 (-1.04, 0.141) |
| Methyl Paraben | 511 | 0.051 (-0.389, 0.491) | 20 | 0.132 (-1.333, 1.597) | 303 | 0.262 (-0.241, 0.764) | 242 | -0.398 (-1.028, 0.232) |
| Propyl paraben | 511 | -0.048 (-0.381, 0.285) | 20 | -0.322 (-1.352, 0.708) | 303 | 0.051 (-0.334, 0.436) | 242 | -0.238 (-0.707, 0.231) |

Outcome: Birthweight

|  | **Across Pregnancy** | | **Trimester 1** | | **Trimester 2** | | **Trimester 3** | |
| --- | --- | --- | --- | --- | --- | --- | --- | --- |
| **Phenol Name** | **N** | **Beta (95% CI)** | **N** | **Beta (95% CI)** | **N** | **Beta (95% CI)** | **N** | **Beta (95% CI)** |
| Sum Bisphenols | 686 | 47.208 (-58.767, 153.182) | 329 | 31.939 (-99.066, 162.945) | 646 | 30.891 (-67.342, 129.123) | 312 | -23.11 (-137.705, 91.485) |
| Bisphenol A | 1059 | 22.708 (-46.845, 92.261) | 408 | 60.031 (-33.356, 153.419) | 821 | -7.711 (-79.968, 64.545) | 520 | 1.55 (-72.199, 75.299) |
| Bisphenol F | 700 | -3.598 (-77.174, 69.977) | 340 | -12.206 (-125.232, 100.82) | 652 | 14.059 (-63.058, 91.176) | 312 | -24.737 (-121.789, 72.314) |
| Bisphenol S | 829 | 43.909 (-30.568, 118.386) | 331 | -0.278 (-93.682, 93.127) | 709 | 50.776 (-22.004, 123.555) | 391 | 13.639 (-59.376, 86.654) |
| 2,4-Dichlorophenol | 504 | **130.714 (51.172, 210.256)** | 11 | -110.995 (-981.349, 759.359) | 296 | **115.087 (0.543, 229.632)** | 244 | **149.406 (42.763, 256.049)** |
| 2,5-Dichlorophenol | 504 | **71.71 (16.846, 126.573)** | 12 | -36.102 (-677.259, 605.055) | 296 | 17.983 (-58.88, 94.847) | 244 | **97.418 (23.321, 171.515)** |
| Benzophenone-3 | 657 | **-65.07 (-115.963, -14.178)** | 96 | -57.107 (-217.401, 103.187) | 447 | -26.436 (-89.249, 36.377) | 250 | -62.653 (-135.249, 9.943) |
| Triclosan | 699 | 4.057 (-40.508, 48.622) | 121 | -54.545 (-174.216, 65.126) | 455 | 6.423 (-46.926, 59.772) | 256 | 46.02 (-19.603, 111.643) |
| Butyl paraben | 552 | 4.815 (-47.659, 57.288) | 36 | -137.397 (-361.182, 86.388) | 319 | -8.924 (-76.733, 58.885) | 249 | 14.7 (-60.694, 90.094) |
| Ethyl paraben | 461 | -30.751 (-91.917, 30.414) | 47 | -153.351 (-334.119, 27.416) | 322 | -7.097 (-83.228, 69.035) | 142 | -33.22 (-131.789, 65.35) |
| Methyl Paraben | 576 | -16.838 (-82.239, 48.563) | 48 | 1.264 (-223.049, 225.577) | 325 | -43.411 (-125.783, 38.961) | 257 | -27.893 (-120.093, 64.307) |
| Propyl paraben | 574 | 4.583 (-45.17, 54.337) | 47 | -32.623 (-216.932, 151.687) | 325 | -35.813 (-98.252, 26.627) | 256 | 14.559 (-54.272, 83.389) |

Outcome: Birthweight for Gestational Age Z-Score

|  | **Across Pregnancy** | | **Trimester 1** | | **Trimester 2** | | **Trimester 3** | |
| --- | --- | --- | --- | --- | --- | --- | --- | --- |
| **Phenol Name** | **N** | **Beta (95% CI)** | **N** | **Beta (95% CI)** | **N** | **Beta (95% CI)** | **N** | **Beta (95% CI)** |
| Sum Bisphenols | 686 | 0.008 (-0.218, 0.234) | 329 | 0.128 (-0.133, 0.389) | 646 | 0.085 (-0.121, 0.291) | 312 | -0.1 (-0.36, 0.159) |
| Bisphenol A | 1058 | -0.016 (-0.166, 0.133) | 408 | 0.14 (-0.049, 0.329) | 821 | -0.037 (-0.188, 0.115) | 519 | 0.006 (-0.164, 0.176) |
| Bisphenol F | 700 | -0.035 (-0.191, 0.121) | 340 | -0.046 (-0.272, 0.18) | 652 | 0.026 (-0.136, 0.187) | 312 | -0.067 (-0.287, 0.153) |
| Bisphenol S | 829 | 0.055 (-0.106, 0.215) | 331 | 0.112 (-0.072, 0.297) | 709 | 0.107 (-0.046, 0.26) | 391 | -0.06 (-0.228, 0.107) |
| 2,4-Dichlorophenol | 503 | **0.286 (0.099, 0.472)** | 11 | 0.494 (-1.427, 2.415) | 296 | **0.314 (0.044, 0.584)** | 243 | **0.329 (0.079, 0.578)** |
| 2,5-Dichlorophenol | 503 | **0.129 (0, 0.258)** | 12 | 0.118 (-1.19, 1.427) | 296 | 0.031 (-0.151, 0.212) | 243 | **0.229 (0.056, 0.402)** |
| Benzophenone-3 | 656 | **-0.118 (-0.231, -0.004)** | 96 | -0.209 (-0.473, 0.056) | 447 | -0.053 (-0.19, 0.084) | 249 | **-0.169 (-0.339, 0)** |
| Triclosan | 698 | 0.036 (-0.063, 0.134) | 121 | -0.15 (-0.36, 0.06) | 455 | 0.078 (-0.038, 0.193) | 255 | 0.066 (-0.089, 0.22) |
| Butyl paraben | 551 | -0.07 (-0.191, 0.051) | 36 | -0.425 (-0.874, 0.024) | 319 | -0.114 (-0.27, 0.041) | 248 | 0.069 (-0.107, 0.246) |
| Ethyl paraben | 461 | -0.054 (-0.193, 0.086) | 47 | **-0.423 (-0.778, -0.067)** | 322 | 0.027 (-0.146, 0.2) | 142 | -0.084 (-0.308, 0.14) |
| Methyl Paraben | 575 | -0.077 (-0.227, 0.072) | 48 | -0.078 (-0.535, 0.379) | 325 | -0.125 (-0.312, 0.063) | 256 | -0.023 (-0.238, 0.192) |
| Propyl paraben | 573 | -0.005 (-0.118, 0.109) | 47 | -0.157 (-0.531, 0.217) | 325 | -0.06 (-0.202, 0.081) | 255 | 0.056 (-0.105, 0.217) |

**Supplement Table 4b2: Parity-Stratified Adjusted Associations of Phenol Exposures with Categorical Birth Outcomes, Parity=1 Only**Models adjusted for maternal race/ethnicity and education, and child sex. Blank cells reflect model non-convergence.

Outcome: Preterm

|  | **Across Pregnancy** | | **Trimester 1** | | **Trimester 2** | | **Trimester 3** | |
| --- | --- | --- | --- | --- | --- | --- | --- | --- |
| **Phenol Name** | **N** | **OR (95% CI)** | **N** | **OR (95% CI)** | **N** | **OR (95% CI)** | **N** | **OR (95% CI)** |
| Sum Bisphenols | 686 | 0.73 (0.3, 1.79) | 329 | 0.72 (0.23, 2.23) | 646 | 1.65 (0.77, 3.57) | 312 | 0.41 (0.12, 1.41) |
| Bisphenol A | 1059 | 0.94 (0.54, 1.62) | 408 | 0.64 (0.3, 1.34) | 821 | 1.13 (0.64, 1.99) | 520 | 1.2 (0.57, 2.51) |
| Bisphenol F | 700 | 1.31 (0.75, 2.28) | 340 | 1.33 (0.58, 3.06) | 652 | 1.46 (0.82, 2.58) | 312 | 0.75 (0.24, 2.34) |
| Bisphenol S | 829 | 0.56 (0.29, 1.08) | 331 | 1.3 (0.61, 2.76) | 709 | 0.98 (0.53, 1.82) | 391 | 0.44 (0.19, 1.04) |
| 2,4-Dichlorophenol | 504 | 1.05 (0.54, 2.06) |  |  | 296 | 0.88 (0.31, 2.48) | 244 | 1.2 (0.48, 3) |
| 2,5-Dichlorophenol | 504 | 1.04 (0.65, 1.66) |  |  | 296 | 1.02 (0.53, 1.99) | 244 | 1.43 (0.73, 2.8) |
| Benzophenone-3 | 657 | 1.24 (0.82, 1.87) | 96 | 0.92 (0.33, 2.59) | 447 | 1.25 (0.75, 2.09) | 250 | 1 (0.52, 1.92) |
| Triclosan | 699 | 1.33 (0.93, 1.9) | 121 | 0.6 (0.23, 1.6) | 455 | 1.36 (0.9, 2.06) | 256 | 1.26 (0.7, 2.26) |
| Butyl paraben | 552 | 0.94 (0.61, 1.45) | 36 | 0.43 (0.02, 11.83) | 319 | 0.84 (0.46, 1.53) | 249 | 1.36 (0.75, 2.46) |
| Ethyl paraben | 461 | 1.1 (0.63, 1.91) | 47 | 0.7 (0.1, 4.64) | 322 | 1.14 (0.6, 2.16) | 142 | 1.13 (0.33, 3.87) |
| Methyl Paraben | 576 | 1.09 (0.63, 1.88) | 48 | 1.44 (0.18, 11.3) | 325 | 1.08 (0.54, 2.15) | 257 | 1.28 (0.55, 3.01) |
| Propyl paraben | 574 | 1.22 (0.8, 1.86) | 47 | 1.27 (0.3, 5.31) | 325 | 1.46 (0.84, 2.51) | 256 | 1.29 (0.67, 2.45) |

Outcome: Small for Gestational Age

|  | **Across Pregnancy** | | **Trimester 1** | | **Trimester 2** | | **Trimester 3** | |
| --- | --- | --- | --- | --- | --- | --- | --- | --- |
| **Phenol Name** | **N** | **OR (95% CI)** | **N** | **OR (95% CI)** | **N** | **OR (95% CI)** | **N** | **OR (95% CI)** |
| Sum Bisphenols | 686 | 1.07 (0.51, 2.27) | 329 | 0.91 (0.38, 2.17) | 646 | 1.15 (0.56, 2.35) | 312 | **2.28 (1.03, 5.03)** |
| Bisphenol A | 1058 | 1.53 (0.94, 2.49) | 408 | 0.83 (0.45, 1.52) | 821 | 1.38 (0.85, 2.26) | 519 | **1.77 (1.03, 3.05)** |
| Bisphenol F | 700 | 1.18 (0.71, 1.97) | 340 | 1.35 (0.67, 2.73) | 652 | 0.98 (0.56, 1.73) | 312 | 1.57 (0.83, 2.98) |
| Bisphenol S | 829 | 0.88 (0.52, 1.47) | 331 | 1.02 (0.55, 1.9) | 709 | 0.82 (0.49, 1.37) | 391 | 1.38 (0.82, 2.32) |
| 2,4-Dichlorophenol | 503 | 0.65 (0.34, 1.25) |  |  | 296 | 0.73 (0.29, 1.86) | 243 | 0.47 (0.19, 1.16) |
| 2,5-Dichlorophenol | 503 | 0.83 (0.54, 1.27) |  |  | 296 | 0.99 (0.55, 1.76) | 243 | 0.7 (0.4, 1.23) |
| Benzophenone-3 | 656 | 1.34 (0.94, 1.92) |  |  | 447 | 1.14 (0.74, 1.77) | 249 | 1.52 (0.92, 2.52) |
| Triclosan | 698 | 1.16 (0.85, 1.59) |  |  | 455 | 1.23 (0.86, 1.77) | 255 | 1.05 (0.64, 1.71) |
| Butyl paraben | 551 | 1.1 (0.77, 1.57) | 36 | 8.34 (0.7, 99.52) | 319 | 1.15 (0.73, 1.81) | 248 | 0.87 (0.49, 1.55) |
| Ethyl paraben | 461 | 1.25 (0.82, 1.92) | 47 | 11.05 (0.92, 133.07) | 322 | 1.08 (0.63, 1.84) | 142 | 1.37 (0.67, 2.84) |
| Methyl Paraben | 575 | 1.24 (0.77, 2.01) | 48 | 169.72 (0.43, 67666.18) | 325 | 1.58 (0.86, 2.9) | 256 | 0.74 (0.38, 1.44) |
| Propyl paraben | 573 | 1.13 (0.79, 1.62) | 47 | 49.04 (0.19, 12412.39) | 325 | 1.32 (0.83, 2.09) | 255 | 0.81 (0.48, 1.34) |

Outcome: Large for Gestational Age

|  | **Across Pregnancy** | | **Trimester 1** | | **Trimester 2** | | **Trimester 3** | |
| --- | --- | --- | --- | --- | --- | --- | --- | --- |
| **Phenol Name** | **N** | **OR (95% CI)** | **N** | **OR (95% CI)** | **N** | **OR (95% CI)** | **N** | **OR (95% CI)** |
| Sum Bisphenols | 686 | 1.31 (0.69, 2.5) | 329 | 1.69 (0.65, 4.4) | 646 | 1.51 (0.84, 2.69) | 312 | 1.58 (0.63, 3.98) |
| Bisphenol A | 1058 | 1.23 (0.78, 1.92) | 408 | 1.26 (0.64, 2.51) | 821 | 1.14 (0.73, 1.78) | 519 | **1.9 (1.05, 3.44)** |
| Bisphenol F | 700 | 1.14 (0.74, 1.77) | 340 | 1.97 (0.96, 4.03) | 652 | 1.27 (0.81, 1.98) | 312 | 1.09 (0.51, 2.34) |
| Bisphenol S | 829 | 1.18 (0.72, 1.93) | 331 | 0.95 (0.46, 1.97) | 709 | 1.24 (0.78, 1.96) | 391 | 1.02 (0.53, 1.94) |
| 2,4-Dichlorophenol | 503 | **1.68 (1, 2.82)** |  |  | 296 | **2.03 (1.03, 4.01)** | 243 | 2.21 (1, 4.92) |
| 2,5-Dichlorophenol | 503 | 1.26 (0.87, 1.8) |  |  | 296 | 1.26 (0.79, 2.01) | 243 | 1.71 (0.97, 3) |
| Benzophenone-3 | 656 | 0.82 (0.59, 1.12) | 96 | 0.57 (0.21, 1.51) | 447 | 0.97 (0.67, 1.39) | 249 | 0.76 (0.45, 1.31) |
| Triclosan | 698 | 1.21 (0.92, 1.58) | 121 | 0.53 (0.24, 1.14) | 455 | 1.26 (0.93, 1.7) | 255 | **1.7 (1.06, 2.72)** |
| Butyl paraben | 551 | 0.78 (0.56, 1.1) | 36 | 1.3 (0.4, 4.27) | 319 | **0.63 (0.4, 0.98)** | 248 | 0.96 (0.55, 1.69) |
| Ethyl paraben | 461 | 0.92 (0.63, 1.35) | 47 | 0.72 (0.25, 2.11) | 322 | 0.97 (0.63, 1.49) | 142 | 1.91 (0.78, 4.66) |
| Methyl Paraben | 575 | 0.83 (0.56, 1.23) | 48 | 2.03 (0.53, 7.68) | 325 | 0.74 (0.46, 1.18) | 256 | 0.71 (0.36, 1.39) |
| Propyl paraben | 573 | 0.97 (0.72, 1.31) | 47 | 1.52 (0.55, 4.18) | 325 | 0.8 (0.57, 1.14) | 255 | 0.93 (0.55, 1.56) |

Outcome: Low Birthweight

|  | **Across Pregnancy** | | **Trimester 1** | | **Trimester 2** | | **Trimester 3** | |
| --- | --- | --- | --- | --- | --- | --- | --- | --- |
| **Phenol Name** | **N** | **OR (95% CI)** | **N** | **OR (95% CI)** | **N** | **OR (95% CI)** | **N** | **OR (95% CI)** |
| Sum Bisphenols | 686 | 0.72 (0.26, 2.01) | 329 | 0.66 (0.21, 2.09) | 646 | 0.88 (0.34, 2.3) | 312 | 0.92 (0.28, 2.98) |
| Bisphenol A | 1059 | 0.92 (0.48, 1.76) | 408 | 0.68 (0.33, 1.41) | 821 | 1.06 (0.56, 2) | 520 | 0.92 (0.4, 2.12) |
| Bisphenol F | 700 | 1.31 (0.69, 2.47) | 340 | 1.5 (0.67, 3.39) | 652 | 1.17 (0.59, 2.34) | 312 | 0.66 (0.2, 2.14) |
| Bisphenol S | 829 | 0.63 (0.3, 1.33) | 331 | 1.04 (0.48, 2.25) | 709 | 0.6 (0.31, 1.19) | 391 | 1.56 (0.65, 3.78) |
| 2,4-Dichlorophenol | 504 | 0.45 (0.15, 1.4) |  |  | 296 | 0.49 (0.1, 2.34) | 244 | 0.55 (0.13, 2.35) |
| 2,5-Dichlorophenol | 504 | **0.46 (0.21, 0.98)** |  |  | 296 | 0.52 (0.18, 1.5) |  |  |
| Benzophenone-3 | 657 | 1.35 (0.77, 2.36) | 96 | 1.06 (0.39, 2.91) | 447 | 1.24 (0.66, 2.36) |  |  |
| Triclosan | 699 | 0.95 (0.56, 1.61) | 121 | 0.54 (0.19, 1.51) | 455 | 1.07 (0.62, 1.86) |  |  |
| Butyl paraben | 552 | 1.23 (0.72, 2.12) | 36 | 3.08 (0.51, 18.63) | 319 | 1.19 (0.6, 2.38) |  |  |
| Ethyl paraben | 461 | 1.14 (0.55, 2.34) | 47 | 1.49 (0.31, 7.23) | 322 | 0.71 (0.27, 1.84) | 142 | 3.16 (0.67, 14.87) |
| Methyl Paraben | 576 | 1.11 (0.52, 2.36) | 48 | 2.31 (0.24, 21.89) | 325 | 1.1 (0.43, 2.76) | 257 | 1.9 (0.43, 8.36) |
| Propyl paraben | 574 | 1.18 (0.66, 2.11) | 47 | 2.63 (0.59, 11.74) | 325 | 1.12 (0.55, 2.27) |  |  |

Outcome: Low Birthweight (Preterms only)

|  | **Across Pregnancy** | | **Trimester 1** | | **Trimester 2** | | **Trimester 3** | |
| --- | --- | --- | --- | --- | --- | --- | --- | --- |
| **Phenol Name** | **N** | **OR (95% CI)** | **N** | **OR (95% CI)** | **N** | **OR (95% CI)** | **N** | **OR (95% CI)** |
| Sum Bisphenols | 43 | 1.18 (0.17, 8.22) | 21 | 1.94 (0.02, 185.5) | 41 | 0.45 (0.06, 3.61) |  |  |
| Bisphenol A | 74 | 0.59 (0.18, 1.92) | 28 | 0.85 (0.12, 6.2) | 56 | 0.63 (0.13, 2.95) | 32 | 1.1 (0.16, 7.66) |
| Bisphenol F | 48 | 1.33 (0.34, 5.22) | 24 | 0.55 (0.02, 12.27) | 43 | 1.25 (0.31, 5.11) |  |  |
| Bisphenol S | 49 | 1.17 (0.27, 5.14) | 22 | 18.19 (0.03, 11042.19) | 43 | 0.51 (0.14, 1.91) |  |  |
| 2,4-Dichlorophenol | 32 | 0.43 (0.05, 3.55) |  |  | 16 | 0.27 (0.01, 5.4) | 18 | 1.2 (0, 428.38) |
| 2,5-Dichlorophenol | 32 | 0.36 (0.06, 2.08) |  |  | 16 | 0.28 (0.02, 3.18) | 18 | 1.35 (0.01, 149.07) |
| Benzophenone-3 | 42 | 1.07 (0.37, 3.12) |  |  | 26 | 0.72 (0.15, 3.54) | 18 | 0.06 (0, 21.33) |
| Triclosan | 45 | 0.75 (0.33, 1.68) |  |  | 29 | 0.76 (0.3, 1.89) | 18 | 0.68 (0.11, 4.3) |
| Butyl paraben | 38 | 1.11 (0.33, 3.72) |  |  | 21 | 3.66 (0.47, 28.65) | 18 | 1.35 (0.2, 9.19) |
| Ethyl paraben | 27 | 0.89 (0.21, 3.75) |  |  | 21 | 0.55 (0.11, 2.73) |  |  |
| Methyl Paraben | 40 | 0.94 (0.18, 5.03) |  |  | 22 | 0.97 (0.08, 12.27) | 18 | 4.11 (0.12, 136.37) |
| Propyl paraben | 40 | 0.71 (0.19, 2.64) |  |  | 22 | 0.25 (0.03, 2.2) | 18 | 4.06 (0.23, 73.07) |

Outcome: Low Birthweight (Terms only)

|  | **Across Pregnancy** | | **Trimester 1** | | **Trimester 2** | | **Trimester 3** | |
| --- | --- | --- | --- | --- | --- | --- | --- | --- |
| **Phenol Name** | **N** | **OR (95% CI)** | **N** | **OR (95% CI)** | **N** | **OR (95% CI)** | **N** | **OR (95% CI)** |
| Sum Bisphenols |  |  |  |  | 605 | 0.93 (0.14, 6.08) |  |  |
| Bisphenol A | 985 | 1.25 (0.42, 3.78) | 380 | 0.78 (0.26, 2.38) | 765 | 1.57 (0.52, 4.75) | 488 | 0.79 (0.25, 2.47) |
| Bisphenol F | 652 | 1.2 (0.37, 3.9) | 316 | 1.01 (0.27, 3.87) | 609 | 0.98 (0.24, 4.07) |  |  |
| Bisphenol S | 780 | 0.87 (0.23, 3.35) |  |  |  |  | 374 | 2.82 (0.76, 10.45) |
| 2,4-Dichlorophenol | 472 | 0.19 (0.02, 1.65) |  |  | 280 | 0.1 (0, 9.97) | 226 | 0.17 (0.01, 2.23) |
| 2,5-Dichlorophenol | 472 | **0.2 (0.05, 0.85)** |  |  | 280 | 0.19 (0.01, 2.43) |  |  |
| Benzophenone-3 | 615 | 1.42 (0.48, 4.2) |  |  | 421 | 1.46 (0.33, 6.53) |  |  |
| Triclosan | 654 | 0.44 (0.1, 1.89) |  |  | 426 | 0.34 (0.05, 2.46) |  |  |
| Butyl paraben |  |  |  |  |  |  |  |  |
| Ethyl paraben |  |  |  |  | 301 | 0.1 (0, 11.03) | 137 | 44.99 (0, .) |
| Methyl Paraben |  |  |  |  | 303 | 3.64 (0.17, 79.55) | 239 | 1.85 (0.2, 17.16) |
| Propyl paraben | 534 | 1.98 (0.62, 6.32) |  |  | 303 | 2.35 (0.27, 20.2) |  |  |

**Supplement Table 4a3: Parity-Stratified Adjusted Associations of Phenol Exposures with Birth Outcomes (Continuously Measured), Parity=2+ Only**Models adjusted for maternal race/ethnicity and education, and child sex. BW for GA model removes parity from covariates.

Outcome: Gestational Age

|  | **Across Pregnancy** | | **Trimester 1** | | **Trimester 2** | | **Trimester 3** | |
| --- | --- | --- | --- | --- | --- | --- | --- | --- |
| **Phenol Name** | **N** | **Beta (95% CI)** | **N** | **Beta (95% CI)** | **N** | **Beta (95% CI)** | **N** | **Beta (95% CI)** |
| Sum Bisphenols | 218 | -0.451 (-1.108, 0.206) | 60 | -0.601 (-1.789, 0.586) | 197 | -0.079 (-0.683, 0.525) | 70 | -0.092 (-1.098, 0.914) |
| Bisphenol A | 473 | -0.073 (-0.52, 0.374) | 113 | -0.272 (-1.296, 0.752) | 307 | -0.182 (-0.655, 0.292) | 218 | 0.391 (-0.124, 0.907) |
| Bisphenol F | 234 | -0.266 (-0.645, 0.113) | 70 | -0.622 (-1.326, 0.083) | 204 | -0.046 (-0.477, 0.386) | 72 | -0.532 (-1.207, 0.142) |
| Bisphenol S | 307 | -0.308 (-0.692, 0.076) | 62 | -0.726 (-1.592, 0.14) | 237 | -0.198 (-0.628, 0.232) | 118 | -0.168 (-0.726, 0.389) |
| 2,4-Dichlorophenol | 313 | 0.298 (-0.01, 0.607) | 11 | -0.083 (-0.937, 0.772) | 166 | -0.08 (-0.561, 0.401) | 168 | 0.357 (-0.019, 0.733) |
| 2,5-Dichlorophenol | 314 | 0.143 (-0.061, 0.348) | 11 | -0.799 (-2.236, 0.638) | 167 | -0.102 (-0.401, 0.197) | 168 | 0.185 (-0.07, 0.441) |
| Benzophenone-3 | 398 | -0.159 (-0.346, 0.029) | 52 | 0.011 (-0.425, 0.447) | 248 | -0.034 (-0.242, 0.174) | 172 | -0.217 (-0.516, 0.082) |
| Triclosan | 440 | -0.045 (-0.22, 0.13) | 69 | 0.044 (-0.422, 0.511) | 265 | -0.051 (-0.239, 0.136) | 180 | -0.074 (-0.339, 0.191) |
| Butyl paraben | 348 | -0.033 (-0.222, 0.156) | 31 | 0.443 (-0.167, 1.053) | 180 | 0.011 (-0.201, 0.222) | 170 | -0.081 (-0.362, 0.199) |
| Ethyl paraben | 287 | -0.054 (-0.266, 0.159) | 39 | -0.213 (-0.895, 0.469) | 189 | 0.001 (-0.248, 0.25) | 95 | -0.077 (-0.396, 0.242) |
| Methyl Paraben | 376 | -0.05 (-0.285, 0.184) | 40 | -0.312 (-0.936, 0.311) | 190 | -0.134 (-0.375, 0.108) | 180 | 0.045 (-0.299, 0.39) |
| Propyl paraben | 370 | 0.033 (-0.163, 0.23) | 40 | 0.389 (-0.119, 0.896) | 185 | -0.084 (-0.295, 0.128) | 179 | 0.024 (-0.255, 0.302) |

Outcome: Birth Length

|  | **Across Pregnancy** | | **Trimester 1** | | **Trimester 2** | | **Trimester 3** | |
| --- | --- | --- | --- | --- | --- | --- | --- | --- |
| **Phenol Name** | **N** | **Beta (95% CI)** | **N** | **Beta (95% CI)** | **N** | **Beta (95% CI)** | **N** | **Beta (95% CI)** |
| Sum Bisphenols | 207 | -0.107 (-1.335, 1.121) | 54 | -1.237 (-2.747, 0.272) | 189 | -0.575 (-1.759, 0.609) | 62 | -0.213 (-2.126, 1.7) |
| Bisphenol A | 427 | -0.17 (-0.9, 0.56) | 87 | **-1.561 (-2.835, -0.288)** | 276 | -0.455 (-1.247, 0.336) | 197 | 0.312 (-0.645, 1.268) |
| Bisphenol F | 222 | -0.413 (-1.101, 0.274) | 64 | **-1.197 (-2.131, -0.263)** | 196 | -0.549 (-1.374, 0.276) | 63 | -0.257 (-1.512, 0.997) |
| Bisphenol S | 294 | 0.203 (-0.55, 0.955) | 56 | -0.766 (-1.916, 0.384) | 228 | -0.119 (-0.954, 0.716) | 109 | -0.376 (-1.482, 0.73) |
| 2,4-Dichlorophenol | 304 | **0.856 (0.29, 1.422)** | 11 | **2.376 (1.099, 3.653)** | 162 | **1.219 (0.251, 2.187)** | 162 | **0.86 (0.152, 1.568)** |
| 2,5-Dichlorophenol | 305 | **0.512 (0.143, 0.88)** | 11 | **-3.795 (-7.162, -0.428)** | 163 | 0.117 (-0.478, 0.712) | 162 | **0.613 (0.144, 1.083)** |
| Benzophenone-3 | 389 | -0.343 (-0.698, 0.013) | 52 | 0.27 (-0.469, 1.01) | 244 | -0.049 (-0.476, 0.377) | 166 | **-0.638 (-1.191, -0.084)** |
| Triclosan | 383 | -0.124 (-0.47, 0.222) | 48 | 0.232 (-0.58, 1.045) | 242 | -0.19 (-0.6, 0.221) | 166 | -0.011 (-0.516, 0.495) |
| Butyl paraben | 309 | 0.041 (-0.351, 0.433) | 16 | 1.564 (-1.428, 4.555) | 165 | 0.216 (-0.248, 0.681) | 160 | 0.045 (-0.511, 0.601) |
| Ethyl paraben | 233 | 0.002 (-0.465, 0.469) | 18 | -0.862 (-3.259, 1.534) | 166 | 0.272 (-0.239, 0.784) | 84 | -0.463 (-1.365, 0.438) |
| Methyl Paraben | 318 | -0.447 (-0.922, 0.028) | 19 | -1.617 (-3.292, 0.058) | 167 | -0.331 (-0.859, 0.197) | 165 | -0.301 (-0.994, 0.393) |
| Propyl paraben | 318 | -0.367 (-0.751, 0.017) | 19 | -1.426 (-3.177, 0.325) | 167 | -0.319 (-0.758, 0.119) | 165 | -0.2 (-0.747, 0.347) |

Outcome: Birthweight

|  | **Across Pregnancy** | | **Trimester 1** | | **Trimester 2** | | **Trimester 3** | |
| --- | --- | --- | --- | --- | --- | --- | --- | --- |
| **Phenol Name** | **N** | **Beta (95% CI)** | **N** | **Beta (95% CI)** | **N** | **Beta (95% CI)** | **N** | **Beta (95% CI)** |
| Sum Bisphenols | 218 | -27.134 (-236.145, 181.878) | 60 | -26.77 (-349.441, 295.902) | 197 | -80.943 (-280.911, 119.024) | 70 | 107.728 (-221.186, 436.642) |
| Bisphenol A | 473 | -39.281 (-167.668, 89.106) | 113 | -139.075 (-390.465, 112.314) | 307 | -60.583 (-198.05, 76.884) | 218 | 73.223 (-86.026, 232.472) |
| Bisphenol F | 234 | -20.971 (-136.879, 94.937) | 70 | -132.324 (-320.256, 55.609) | 204 | -30.513 (-170.333, 109.306) | 72 | -39.358 (-264.461, 185.745) |
| Bisphenol S | 307 | -21.654 (-148.876, 105.569) | 62 | 34.089 (-207.871, 276.048) | 237 | -24.975 (-167.486, 117.535) | 118 | -56.198 (-252.264, 139.868) |
| 2,4-Dichlorophenol | 313 | 73.404 (-29.072, 175.879) | 11 | -76.621 (-377.086, 223.844) | 166 | 148.49 (-31.649, 328.63) | 168 | 114.388 (-8.126, 236.903) |
| 2,5-Dichlorophenol | 314 | 45.56 (-22.27, 113.39) | 11 | -87.646 (-668.711, 493.419) | 167 | 37.295 (-75.041, 149.631) | 168 | 69.266 (-13.789, 152.321) |
| Benzophenone-3 | 398 | -47.366 (-108.358, 13.626) | 52 | -51.462 (-193.24, 90.315) | 248 | -38.993 (-111.218, 33.231) | 172 | -48.194 (-145.43, 49.041) |
| Triclosan | 440 | 13.658 (-43.796, 71.111) | 69 | 66.701 (-88.636, 222.038) | 265 | -11.116 (-77.079, 54.846) | 180 | 36.522 (-50.155, 123.2) |
| Butyl paraben | 348 | -12.475 (-73.88, 48.931) | 31 | 40.922 (-164.86, 246.704) | 180 | 24.575 (-53.979, 103.129) | 170 | -41.354 (-132.567, 49.858) |
| Ethyl paraben | 287 | -3.67 (-78.547, 71.206) | 39 | -164.009 (-387.843, 59.826) | 189 | 69.749 (-20.385, 159.883) | 95 | -131.355 (-269.588, 6.879) |
| Methyl Paraben | 376 | **-120.974 (-196.009, -45.939)** | 40 | -185.82 (-388.31, 16.669) | 190 | -69.206 (-156.825, 18.412) | 180 | -110.724 (-223.092, 1.644) |
| Propyl paraben | 370 | -62.843 (-126.121, 0.434) | 40 | 14.275 (-160.372, 188.921) | 185 | **-88.008 (-163.791, -12.226)** | 179 | -63.013 (-154.15, 28.125) |

Outcome: Birthweight for Gestational Age Z-Score

|  | **Across Pregnancy** | | **Trimester 1** | | **Trimester 2** | | **Trimester 3** | |
| --- | --- | --- | --- | --- | --- | --- | --- | --- |
| **Phenol Name** | **N** | **Beta (95% CI)** | **N** | **Beta (95% CI)** | **N** | **Beta (95% CI)** | **N** | **Beta (95% CI)** |
| Sum Bisphenols | 218 | 0.153 (-0.326, 0.631) | 60 | 0.069 (-0.56, 0.698) | 197 | -0.146 (-0.602, 0.31) | 70 | 0.322 (-0.35, 0.994) |
| Bisphenol A | 471 | -0.071 (-0.35, 0.208) | 113 | -0.252 (-0.656, 0.152) | 307 | -0.078 (-0.372, 0.216) | 217 | 0.006 (-0.34, 0.352) |
| Bisphenol F | 234 | 0.091 (-0.171, 0.352) | 70 | -0.093 (-0.464, 0.279) | 204 | -0.015 (-0.333, 0.302) | 72 | 0.226 (-0.237, 0.689) |
| Bisphenol S | 307 | 0.073 (-0.216, 0.361) | 62 | 0.194 (-0.271, 0.66) | 237 | 0.024 (-0.304, 0.351) | 118 | -0.142 (-0.552, 0.268) |
| 2,4-Dichlorophenol | 311 | 0.116 (-0.127, 0.359) | 11 | -0.155 (-0.907, 0.597) | 166 | 0.388 (-0.053, 0.828) | 167 | 0.198 (-0.086, 0.481) |
| 2,5-Dichlorophenol | 312 | 0.094 (-0.067, 0.254) | 11 | -0.299 (-1.335, 0.737) | 167 | 0.124 (-0.15, 0.398) | 167 | 0.14 (-0.052, 0.332) |
| Benzophenone-3 | 396 | -0.059 (-0.203, 0.086) | 52 | -0.127 (-0.451, 0.197) | 248 | -0.077 (-0.253, 0.098) | 171 | -0.041 (-0.266, 0.184) |
| Triclosan | 438 | 0.048 (-0.085, 0.181) | 69 | 0.154 (-0.142, 0.451) | 265 | -0.029 (-0.189, 0.131) | 179 | 0.116 (-0.081, 0.313) |
| Butyl paraben | 346 | -0.025 (-0.169, 0.119) | 31 | -0.135 (-0.593, 0.323) | 180 | 0.054 (-0.139, 0.247) | 169 | -0.074 (-0.284, 0.135) |
| Ethyl paraben | 287 | 0.011 (-0.159, 0.181) | 39 | -0.245 (-0.679, 0.189) | 189 | 0.163 (-0.055, 0.381) | 95 | -0.269 (-0.583, 0.046) |
| Methyl Paraben | 374 | **-0.266 (-0.437, -0.096)** | 40 | -0.319 (-0.716, 0.079) | 190 | -0.09 (-0.304, 0.123) | 179 | **-0.278 (-0.532, -0.023)** |
| Propyl paraben | 368 | **-0.165 (-0.308, -0.022)** | 40 | -0.17 (-0.506, 0.166) | 185 | -0.155 (-0.34, 0.029) | 178 | -0.168 (-0.375, 0.039) |

**Supplement Table 4b3: Parity-Stratified Adjusted Associations of Phenol Exposures with Categorical Birth Outcomes, Parity=2+ Only**Models adjusted for maternal race/ethnicity and education, and child sex. Blank cells reflect model non-convergence.

Outcome: Preterm

|  | **Across Pregnancy** | | **Trimester 1** | | **Trimester 2** | | **Trimester 3** | |
| --- | --- | --- | --- | --- | --- | --- | --- | --- |
| **Phenol Name** | **N** | **OR (95% CI)** | **N** | **OR (95% CI)** | **N** | **OR (95% CI)** | **N** | **OR (95% CI)** |
| Sum Bisphenols | 218 | 2.43 (0.5, 11.77) | 60 | 1.21 (0.18, 8.27) | 197 | 0.95 (0.19, 4.88) | 70 | 0.68 (0.04, 12.53) |
| Bisphenol A | 473 | 1.06 (0.39, 2.85) | 113 | 0.86 (0.25, 2.96) | 307 | 1.23 (0.42, 3.58) | 218 | 0.44 (0.15, 1.29) |
| Bisphenol F | 234 | 1.89 (0.86, 4.13) | 70 | 1.53 (0.53, 4.39) | 204 | 1.95 (0.68, 5.56) | 72 | 2.81 (0.49, 15.98) |
| Bisphenol S | 307 | 1.7 (0.59, 4.94) | 62 | 1.27 (0.28, 5.66) | 237 | 0.95 (0.27, 3.29) | 118 | 0.6 (0.11, 3.36) |
| 2,4-Dichlorophenol | 313 | 0.93 (0.34, 2.54) |  |  | 166 | 0.51 (0.06, 4.42) |  |  |
| 2,5-Dichlorophenol | 314 | 1.11 (0.58, 2.12) |  |  | 167 | 1.16 (0.31, 4.27) |  |  |
| Benzophenone-3 | 398 | 1.36 (0.76, 2.43) |  |  | 248 | 1.38 (0.68, 2.81) |  |  |
| Triclosan | 440 | 0.79 (0.45, 1.4) | 69 | 0.63 (0.17, 2.29) | 265 | 0.67 (0.3, 1.5) |  |  |
| Butyl paraben | 348 | 1.28 (0.72, 2.29) |  |  | 180 | 1.42 (0.61, 3.3) |  |  |
| Ethyl paraben | 287 | 1.49 (0.8, 2.78) | 39 | 1.97 (0.26, 15.19) | 189 | 1.84 (0.76, 4.46) | 95 | 4.03 (0.31, 51.65) |
| Methyl Paraben | 376 | 1.38 (0.65, 2.92) | 40 | 239 (0.31, 181668.49) | 190 | **3.97 (1.02, 15.44)** | 180 | 1.57 (0.39, 6.28) |
| Propyl paraben | 370 | 1.43 (0.78, 2.64) | 40 | 0.81 (0.11, 5.96) | 185 | **4.81 (1.31, 17.67)** |  |  |

Outcome: Small for Gestational Age

|  | **Across Pregnancy** | | **Trimester 1** | | **Trimester 2** | | **Trimester 3** | |
| --- | --- | --- | --- | --- | --- | --- | --- | --- |
| **Phenol Name** | **N** | **OR (95% CI)** | **N** | **OR (95% CI)** | **N** | **OR (95% CI)** | **N** | **OR (95% CI)** |
| Sum Bisphenols | 218 | 0.67 (0.09, 5.08) |  |  | 197 | 0.91 (0.15, 5.44) | 70 | 0.04 (0, 4.7) |
| Bisphenol A | 471 | 0.76 (0.27, 2.19) | 113 | 0.46 (0.08, 2.73) | 307 | 0.89 (0.31, 2.61) | 217 | 0.74 (0.17, 3.17) |
| Bisphenol F | 234 | 0.24 (0.04, 1.4) | 70 | 0.06 (0, 8.49) | 204 | 1.03 (0.24, 4.51) | 72 | 0.13 (0.01, 3.05) |
| Bisphenol S | 307 | **3.35 (1.16, 9.69)** | 62 | 0 (0, 26.68) | 237 | 1.21 (0.36, 4.16) | 118 | 3.97 (0.9, 17.47) |
| 2,4-Dichlorophenol | 311 | 0.74 (0.29, 1.9) |  |  | 166 | 0.86 (0.17, 4.38) | 167 | 0.76 (0.26, 2.22) |
| 2,5-Dichlorophenol | 312 | 0.8 (0.44, 1.45) |  |  | 167 | 1.38 (0.53, 3.6) | 167 | 0.65 (0.32, 1.31) |
| Benzophenone-3 | 396 | 1.5 (0.85, 2.66) | 52 | 1.01 (0.18, 5.66) | 248 | 1.5 (0.76, 2.94) | 171 | 1.85 (0.77, 4.45) |
| Triclosan | 438 | 1.01 (0.6, 1.72) | 69 | 0.7 (0.23, 2.1) | 265 | 1.25 (0.67, 2.35) | 179 | 1.63 (0.74, 3.57) |
| Butyl paraben | 346 | 0.91 (0.51, 1.61) | 31 | 0.8 (0.03, 25.47) | 180 | 0.92 (0.44, 1.92) | 169 | 1.12 (0.47, 2.67) |
| Ethyl paraben | 287 | 1.19 (0.64, 2.23) | 39 | 3.79 (0.12, 118.66) | 189 | 0.58 (0.21, 1.57) | 95 | 2.99 (0.99, 9.04) |
| Methyl Paraben | 374 | 2.07 (0.98, 4.38) | 40 | 1.31 (0.09, 19.49) | 190 | 2.53 (0.97, 6.6) | 179 | 1.87 (0.57, 6.15) |
| Propyl paraben | 368 | 1.59 (0.87, 2.89) | 40 | 0.81 (0.08, 8.17) | 185 | **2.36 (1.06, 5.29)** | 178 | 1.43 (0.58, 3.52) |

Outcome: Large for Gestational Age

|  | **Across Pregnancy** | | **Trimester 1** | | **Trimester 2** | | **Trimester 3** | |
| --- | --- | --- | --- | --- | --- | --- | --- | --- |
| **Phenol Name** | **N** | **OR (95% CI)** | **N** | **OR (95% CI)** | **N** | **OR (95% CI)** | **N** | **OR (95% CI)** |
| Sum Bisphenols | 218 | 1.29 (0.4, 4.1) | 60 | 2.23 (0.19, 25.52) | 197 | 0.51 (0.16, 1.6) | 70 | 1.99 (0.33, 11.98) |
| Bisphenol A | 471 | 0.8 (0.37, 1.75) | 113 | 0.51 (0.11, 2.33) | 307 | 0.71 (0.31, 1.6) | 217 | 1.66 (0.57, 4.83) |
| Bisphenol F | 234 | 1.37 (0.7, 2.68) | 70 | 1.07 (0.37, 3.06) | 204 | 0.81 (0.35, 1.84) | 72 | 1.39 (0.43, 4.52) |
| Bisphenol S | 307 | 1.18 (0.54, 2.55) | 62 | 2.53 (0.41, 15.69) | 237 | 0.85 (0.36, 1.98) | 118 | 0.9 (0.28, 2.93) |
| 2,4-Dichlorophenol | 311 | 0.86 (0.42, 1.74) |  |  | 166 | 2.28 (0.7, 7.37) | 167 | 0.9 (0.36, 2.23) |
| 2,5-Dichlorophenol | 312 | 1.04 (0.66, 1.64) |  |  | 167 | 1.82 (0.89, 3.71) | 167 | 0.99 (0.54, 1.84) |
| Benzophenone-3 | 396 | 0.85 (0.56, 1.29) | 52 | 0.09 (0, 113.69) | 248 | 0.87 (0.54, 1.41) | 171 | 0.84 (0.37, 1.88) |
| Triclosan | 438 | 1.44 (0.99, 2.11) | 69 | 5.51 (0.63, 48.39) | 265 | 1.24 (0.8, 1.91) | 179 | **2.33 (1.19, 4.56)** |
| Butyl paraben | 346 | 1.24 (0.83, 1.84) |  |  | 180 | 1.28 (0.79, 2.06) | 169 | 1.43 (0.73, 2.82) |
| Ethyl paraben | 287 | 1.21 (0.71, 2.06) |  |  | 189 | 1.64 (0.91, 2.94) | 95 | 0.64 (0.14, 2.83) |
| Methyl Paraben | 374 | 0.66 (0.39, 1.11) |  |  | 190 | 0.73 (0.4, 1.33) | 179 | 0.64 (0.27, 1.51) |
| Propyl paraben | 368 | 0.83 (0.54, 1.28) | 40 | 0.03 (0, 511.07) | 185 | 0.63 (0.37, 1.07) | 178 | 0.82 (0.41, 1.66) |

Outcome: Low Birthweight

|  | **Across Pregnancy** | | **Trimester 1** | | **Trimester 2** | | **Trimester 3** | |
| --- | --- | --- | --- | --- | --- | --- | --- | --- |
| **Phenol Name** | **N** | **OR (95% CI)** | **N** | **OR (95% CI)** | **N** | **OR (95% CI)** | **N** | **OR (95% CI)** |
| Sum Bisphenols | 218 | 1.22 (0.06, 26.58) |  |  | 197 | 0.95 (0.06, 14.89) | 70 | 0.5 (0.02, 12.93) |
| Bisphenol A | 473 | 2.28 (0.5, 10.46) | 113 | 1.64 (0.23, 11.6) | 307 | 1.51 (0.3, 7.61) | 218 | 1.98 (0.4, 9.72) |
| Bisphenol F | 234 | 1.18 (0.18, 7.69) | 70 | 7.97 (0.08, 756.8) | 204 | 2.14 (0.26, 17.39) | 72 | 0.46 (0.04, 5.53) |
| Bisphenol S | 307 | 2.61 (0.64, 10.66) |  |  | 237 | 0.57 (0.06, 4.96) | 118 | 3.01 (0.82, 11.02) |
| 2,4-Dichlorophenol | 313 | 1 (0.28, 3.56) |  |  | 166 | 13.3 (0.29, 605.36) | 168 | 0.69 (0.18, 2.67) |
| 2,5-Dichlorophenol | 314 | 0.6 (0.25, 1.41) |  |  | 167 | 2.84 (0.45, 18.05) | 168 | 0.46 (0.18, 1.14) |
| Benzophenone-3 | 398 | 1.08 (0.43, 2.67) | 52 | 0.54 (0, 341.59) | 248 | 0.72 (0.18, 2.89) | 172 | 1.46 (0.54, 3.95) |
| Triclosan | 440 | 1.69 (0.8, 3.61) | 69 | 1.28 (0.21, 7.83) | 265 | **4.41 (1.16, 16.79)** | 180 | 1.79 (0.72, 4.45) |
| Butyl paraben | 348 | 1.09 (0.45, 2.64) |  |  |  |  | 170 | 1.18 (0.48, 2.93) |
| Ethyl paraben | 287 | 1.6 (0.72, 3.59) | 39 | 2.3 (0.25, 20.99) | 189 | 0.3 (0.03, 2.77) | 95 | **3.25 (1.08, 9.75)** |
| Methyl Paraben | 376 | 2.84 (0.89, 9) | 40 | 31.24 (0.21, 4744.07) | 190 | 2.43 (0.3, 19.55) | 180 | 1.86 (0.52, 6.66) |
| Propyl paraben | 370 | 1.68 (0.69, 4.06) | 40 | 0.56 (0.07, 4.56) | 185 | 3.16 (0.47, 21.22) | 179 | 1.62 (0.6, 4.34) |

Outcome: Low Birthweight (Preterms only)

|  | **Across Pregnancy** | | **Trimester 1** | | **Trimester 2** | | **Trimester 3** | |
| --- | --- | --- | --- | --- | --- | --- | --- | --- |
| **Phenol Name** | **N** | **OR (95% CI)** | **N** | **OR (95% CI)** | **N** | **OR (95% CI)** | **N** | **OR (95% CI)** |
| Sum Bisphenols |  |  |  |  |  |  |  |  |
| Bisphenol A | 38 | 1.19 (0.04, 31.43) | 19 | 0.66 (0.02, 26.8) | 23 | 0.63 (0.01, 27.66) |  |  |
| Bisphenol F |  |  |  |  |  |  |  |  |
| Bisphenol S |  |  |  |  |  |  |  |  |
| 2,4-Dichlorophenol |  |  |  |  |  |  |  |  |
| 2,5-Dichlorophenol |  |  |  |  |  |  |  |  |
| Benzophenone-3 |  |  |  |  |  |  |  |  |
| Triclosan |  |  |  |  |  |  |  |  |
| Butyl paraben |  |  |  |  |  |  |  |  |
| Ethyl paraben |  |  |  |  |  |  |  |  |
| Methyl Paraben |  |  |  |  |  |  |  |  |
| Propyl paraben | 23 | 0.9 (0.06, 12.65) |  |  |  |  |  |  |

Outcome: Low Birthweight (Terms only)

|  | **Across Pregnancy** | | **Trimester 1** | | **Trimester 2** | | **Trimester 3** | |
| --- | --- | --- | --- | --- | --- | --- | --- | --- |
| **Phenol Name** | **N** | **OR (95% CI)** | **N** | **OR (95% CI)** | **N** | **OR (95% CI)** | **N** | **OR (95% CI)** |
| Sum Bisphenols | 200 | 0.42 (0, 44.25) |  |  | 183 | 0.67 (0.01, 30.85) | 63 | 0.13 (0, 31.08) |
| Bisphenol A | 435 | 3.14 (0.48, 20.43) |  |  | 284 | 2.22 (0.24, 20.45) | 200 | 1.24 (0.2, 7.58) |
| Bisphenol F | 212 | 0.42 (0.03, 6.96) |  |  | 188 | 1.16 (0.11, 12.5) | 65 | 0.05 (0, 6.2) |
| Bisphenol S | 287 | 2.22 (0.39, 12.55) |  |  | 222 | 0.51 (0.03, 8.58) | 110 | 2.74 (0.59, 12.78) |
| 2,4-Dichlorophenol | 297 | 1.42 (0.41, 4.96) |  |  | 159 | 13.02 (0.28, 609.32) | 160 | 0.98 (0.25, 3.82) |
| 2,5-Dichlorophenol | 298 | 0.8 (0.32, 1.99) |  |  | 160 | 2.79 (0.43, 17.98) | 160 | 0.6 (0.23, 1.61) |
| Benzophenone-3 | 377 | 0.87 (0.33, 2.29) |  |  | 236 | 0.74 (0.18, 3.03) | 164 | 1.13 (0.38, 3.37) |
| Triclosan | 416 | 2.33 (0.95, 5.7) |  |  | 254 | **4.43 (1.16, 16.95)** | 171 | 2.04 (0.75, 5.54) |
| Butyl paraben | 329 | 1.18 (0.49, 2.88) |  |  |  |  | 162 | 1.25 (0.5, 3.17) |
| Ethyl paraben | 270 | 1.15 (0.37, 3.62) |  |  | 181 | 0.3 (0.03, 2.84) | 92 | 2.35 (0.65, 8.5) |
| Methyl Paraben | 353 | 2.45 (0.65, 9.21) |  |  | 182 | 2.41 (0.3, 19.08) | 171 | 1.52 (0.42, 5.52) |
| Propyl paraben | 347 | 2.21 (0.77, 6.32) |  |  | 177 | 3.17 (0.48, 20.76) | 170 | 1.36 (0.5, 3.71) |

**Supplement Table 5a: Sensitivity Analysis for Adjusted Associations of Phenol Exposures with Birth Outcomes (Continuously Measured),
Adding Tobacco as a Covariate**Models adjusted for maternal race/ethnicity, parity, tobacco use, and education, and child sex. BW for GA model removes parity from covariates.

Outcome: Gestational Age

|  | **Across Pregnancy** | | **Trimester 1** | | **Trimester 2** | | **Trimester 3** | |
| --- | --- | --- | --- | --- | --- | --- | --- | --- |
| **Phenol Name** | **N** | **Beta (95% CI)** | **N** | **Beta (95% CI)** | **N** | **Beta (95% CI)** | **N** | **Beta (95% CI)** |
| Sum Bisphenols | 1579 | 0.035 (-0.206, 0.277) | 653 | -0.174 (-0.505, 0.157) | 1479 | -0.072 (-0.295, 0.151) | 648 | -0.004 (-0.275, 0.267) |
| Bisphenol A | 2717 | 0.094 (-0.06, 0.249) | 878 | 0.019 (-0.229, 0.267) | 1952 | -0.038 (-0.197, 0.121) | 1332 | 0.037 (-0.128, 0.202) |
| Bisphenol F | 1636 | 0.005 (-0.154, 0.164) | 695 | -0.193 (-0.461, 0.075) | 1498 | 0.134 (-0.039, 0.306) | 653 | -0.008 (-0.233, 0.218) |
| Bisphenol S | 2042 | -0.14 (-0.299, 0.02) | 666 | **-0.244 (-0.473, -0.015)** | 1664 | -0.089 (-0.254, 0.075) | 917 | -0.052 (-0.219, 0.115) |
| 2,4-Dichlorophenol | 1530 | **0.153 (0.004, 0.302)** | 39 | -0.576 (-1.939, 0.787) | 844 | -0.023 (-0.256, 0.21) | 762 | 0.143 (-0.049, 0.335) |
| 2,5-Dichlorophenol | 1529 | **0.124 (0.022, 0.226)** | 38 | 0.004 (-1.157, 1.165) | 846 | 0.002 (-0.149, 0.153) | 761 | 0.128 (-0.006, 0.262) |
| Benzophenone-3 | 1921 | 0.004 (-0.09, 0.099) | 249 | 0.215 (-0.112, 0.541) | 1225 | 0.072 (-0.043, 0.187) | 784 | -0.014 (-0.153, 0.126) |
| Triclosan | 1913 | -0.023 (-0.111, 0.065) | 244 | -0.035 (-0.314, 0.245) | 1212 | -0.049 (-0.153, 0.055) | 788 | -0.029 (-0.161, 0.103) |
| Butyl paraben | 1575 | **0.128 (0.027, 0.229)** | 51 | 0.08 (-0.3, 0.46) | 880 | **0.158 (0.035, 0.282)** | 774 | 0.028 (-0.119, 0.175) |
| Ethyl paraben | 1258 | 0.02 (-0.091, 0.13) | 78 | 0.242 (-0.244, 0.727) | 881 | 0.036 (-0.097, 0.169) | 432 | -0.031 (-0.201, 0.139) |
| Methyl Paraben | 1629 | 0.107 (-0.01, 0.224) | 80 | 0.278 (-0.161, 0.716) | 886 | 0.095 (-0.046, 0.236) | 798 | 0.001 (-0.165, 0.167) |
| Propyl paraben | 1622 | **0.101 (0.007, 0.194)** | 77 | 0.353 (-0.012, 0.719) | 884 | 0.045 (-0.066, 0.156) | 796 | 0.049 (-0.084, 0.182) |

Outcome: Birth Length

|  | **Across Pregnancy** | | **Trimester 1** | | **Trimester 2** | | **Trimester 3** | |
| --- | --- | --- | --- | --- | --- | --- | --- | --- |
| **Phenol Name** | **N** | **Beta (95% CI)** | **N** | **Beta (95% CI)** | **N** | **Beta (95% CI)** | **N** | **Beta (95% CI)** |
| Sum Bisphenols | 1524 | 0.046 (-0.35, 0.443) | 625 | -0.02 (-0.521, 0.481) | 1428 | 0.003 (-0.368, 0.373) | 617 | -0.074 (-0.519, 0.372) |
| Bisphenol A | 2543 | 0.154 (-0.101, 0.409) | 774 | 0.088 (-0.284, 0.46) | 1837 | -0.083 (-0.342, 0.176) | 1246 | 0.182 (-0.107, 0.472) |
| Bisphenol F | 1576 | -0.182 (-0.446, 0.081) | 666 | **-0.427 (-0.844, -0.009)** | 1446 | 0.014 (-0.271, 0.3) | 619 | 0.031 (-0.34, 0.403) |
| Bisphenol S | 1982 | **0.277 (0.003, 0.551)** | 638 | -0.18 (-0.537, 0.177) | 1611 | 0.226 (-0.051, 0.503) | 883 | -0.235 (-0.527, 0.056) |
| 2,4-Dichlorophenol | 1484 | **0.343 (0.077, 0.609)** | 39 | 1.93 (-0.78, 4.64) | 826 | 0.296 (-0.113, 0.706) | 733 | **0.398 (0.056, 0.739)** |
| 2,5-Dichlorophenol | 1483 | **0.355 (0.175, 0.536)** | 38 | 0.644 (-1.665, 2.952) | 828 | 0.203 (-0.062, 0.468) | 732 | **0.399 (0.162, 0.635)** |
| Benzophenone-3 | 1872 | **-0.272 (-0.439, -0.106)** | 249 | 0.2 (-0.295, 0.695) | 1204 | -0.156 (-0.357, 0.045) | 755 | **-0.381 (-0.628, -0.133)** |
| Triclosan | 1834 | -0.071 (-0.224, 0.082) | 230 | -0.196 (-0.607, 0.216) | 1186 | **-0.204 (-0.382, -0.026)** | 748 | 0.133 (-0.104, 0.371) |
| Butyl paraben | 1516 | 0.041 (-0.143, 0.224) | 46 | -0.424 (-1.196, 0.347) | 859 | -0.071 (-0.295, 0.153) | 740 | 0.104 (-0.16, 0.369) |
| Ethyl paraben | 1202 | 0.108 (-0.097, 0.313) | 63 | 0.03 (-1.036, 1.096) | 857 | 0.18 (-0.059, 0.42) | 414 | -0.103 (-0.436, 0.229) |
| Methyl Paraben | 1548 | -0.155 (-0.372, 0.061) | 65 | -0.342 (-1.39, 0.707) | 862 | -0.196 (-0.45, 0.058) | 755 | -0.149 (-0.454, 0.157) |
| Propyl paraben | 1547 | -0.126 (-0.298, 0.047) | 64 | 0.225 (-0.633, 1.083) | 862 | -0.181 (-0.382, 0.019) | 755 | -0.109 (-0.35, 0.132) |

Outcome: Birth Weight

|  | **Across Pregnancy** | | **Trimester 1** | | **Trimester 2** | | **Trimester 3** | |
| --- | --- | --- | --- | --- | --- | --- | --- | --- |
| **Phenol Name** | **N** | **Beta (95% CI)** | **N** | **Beta (95% CI)** | **N** | **Beta (95% CI)** | **N** | **Beta (95% CI)** |
| Sum Bisphenols | 1579 | -4.234 (-76.39, 67.922) | 653 | 16.94 (-76.738, 110.619) | 1479 | 13.124 (-53.652, 79.899) | 648 | -17.99 (-106.508, 70.528) |
| Bisphenol A | 2717 | 30.041 (-15.073, 75.155) | 878 | 52.523 (-15.944, 120.99) | 1952 | 2.198 (-44.596, 48.991) | 1332 | 19.387 (-31.55, 70.323) |
| Bisphenol F | 1636 | -37.779 (-85.198, 9.641) | 695 | -56.34 (-132.389, 19.709) | 1498 | 8.411 (-43.144, 59.966) | 653 | -14.53 (-87.954, 58.895) |
| Bisphenol S | 2042 | 14.672 (-33.639, 62.982) | 666 | -18.948 (-84.184, 46.288) | 1664 | 39.932 (-9.664, 89.527) | 917 | -34.849 (-88.461, 18.762) |
| 2,4-Dichlorophenol | 1530 | **71.865 (26.32, 117.411)** | 39 | 51.503 (-384.708, 487.713) | 844 | **75.782 (0.931, 150.633)** | 762 | **74.477 (17.271, 131.683)** |
| 2,5-Dichlorophenol | 1529 | **46.359 (15.217, 77.501)** | 38 | 63.857 (-291.627, 419.341) | 846 | 4.522 (-43.948, 52.991) | 761 | **60.095 (20.196, 99.994)** |
| Benzophenone-3 | 1921 | **-39.478 (-67.977, -10.979)** | 249 | -7.671 (-98.505, 83.163) | 1225 | -18.015 (-53.743, 17.712) | 784 | **-50.984 (-92.814, -9.154)** |
| Triclosan | 1913 | -11.529 (-38.174, 15.116) | 244 | -39.632 (-118.068, 38.805) | 1212 | -19.109 (-51.273, 13.055) | 788 | -3.035 (-42.82, 36.751) |
| Butyl paraben | 1575 | 0.542 (-30.418, 31.502) | 51 | -77.702 (-236.244, 80.841) | 880 | 1.461 (-38.252, 41.175) | 774 | -5.849 (-49.866, 38.169) |
| Ethyl paraben | 1258 | -20.817 (-55.913, 14.28) | 78 | -81.375 (-244.135, 81.385) | 881 | 2.22 (-40.504, 44.943) | 432 | **-63.483 (-119.64, -7.327)** |
| Methyl Paraben | 1629 | -26.985 (-62.825, 8.856) | 80 | 36.899 (-110.47, 184.269) | 886 | -37.271 (-82.323, 7.781) | 798 | -37.047 (-86.946, 12.851) |
| Propyl paraben | 1622 | -5.853 (-34.621, 22.916) | 77 | 75.706 (-49.055, 200.467) | 884 | -30.751 (-66.339, 4.837) | 796 | -1.486 (-41.361, 38.39) |

Outcome: Birthweight for Gestational Age Z-Score

|  | **Across Pregnancy** | | **Trimester 1** | | **Trimester 2** | | **Trimester 3** | |
| --- | --- | --- | --- | --- | --- | --- | --- | --- |
| **Phenol Name** | **N** | **Beta (95% CI)** | **N** | **Beta (95% CI)** | **N** | **Beta (95% CI)** | **N** | **Beta (95% CI)** |
| Sum Bisphenols | 1579 | -0.022 (-0.177, 0.133) | 653 | 0.082 (-0.109, 0.274) | 1479 | 0.062 (-0.08, 0.204) | 648 | -0.035 (-0.221, 0.151) |
| Bisphenol A | 2710 | 0.03 (-0.069, 0.128) | 877 | 0.087 (-0.049, 0.224) | 1951 | 0.017 (-0.082, 0.116) | 1327 | 0.036 (-0.078, 0.151) |
| Bisphenol F | 1636 | -0.084 (-0.186, 0.018) | 695 | -0.068 (-0.225, 0.088) | 1498 | -0.04 (-0.149, 0.07) | 653 | -0.017 (-0.171, 0.138) |
| Bisphenol S | 2042 | 0.074 (-0.031, 0.179) | 666 | 0.026 (-0.108, 0.16) | 1664 | **0.131 (0.025, 0.237)** | 917 | -0.093 (-0.208, 0.022) |
| 2,4-Dichlorophenol | 1524 | **0.135 (0.028, 0.243)** | 39 | 0.501 (-0.455, 1.456) | 844 | **0.2 (0.028, 0.371)** | 757 | **0.158 (0.023, 0.293)** |
| 2,5-Dichlorophenol | 1523 | **0.08 (0.007, 0.154)** | 38 | 0.039 (-0.703, 0.78) | 846 | 0.013 (-0.099, 0.124) | 756 | **0.126 (0.032, 0.221)** |
| Benzophenone-3 | 1915 | **-0.108 (-0.173, -0.044)** | 249 | -0.117 (-0.291, 0.057) | 1225 | **-0.079 (-0.158, 0)** | 779 | **-0.13 (-0.229, -0.032)** |
| Triclosan | 1907 | -0.018 (-0.079, 0.042) | 244 | -0.092 (-0.236, 0.052) | 1212 | -0.028 (-0.099, 0.043) | 783 | 0.011 (-0.083, 0.104) |
| Butyl paraben | 1569 | -0.055 (-0.128, 0.018) | 51 | -0.297 (-0.668, 0.074) | 880 | -0.07 (-0.161, 0.021) | 769 | -0.012 (-0.116, 0.092) |
| Ethyl paraben | 1258 | -0.062 (-0.143, 0.018) | 78 | -0.342 (-0.693, 0.01) | 881 | 0 (-0.097, 0.098) | 432 | **-0.155 (-0.278, -0.033)** |
| Methyl Paraben | 1623 | **-0.122 (-0.205, -0.038)** | 80 | -0.068 (-0.395, 0.259) | 886 | **-0.135 (-0.238, -0.033)** | 793 | -0.092 (-0.209, 0.025) |
| Propyl paraben | 1616 | -0.06 (-0.127, 0.007) | 77 | -0.014 (-0.289, 0.262) | 884 | **-0.09 (-0.171, -0.009)** | 791 | -0.025 (-0.118, 0.069) |

**Supplement Table 5b: Sensitivity Analysis for Adjusted Associations of Phenol Exposures with Categorical Birth Outcomes, Adding Tobacco as a Covariate**Models adjusted for maternal race/ethnicity, parity, tobacco use, and education, and child sex. Blank cells reflect model non-convergence.

Outcome: Preterm

|  | **Across Pregnancy** | | **Trimester 1** | | **Trimester 2** | | **Trimester 3** | |
| --- | --- | --- | --- | --- | --- | --- | --- | --- |
| **Phenol Name** | **N** | **OR (95% CI)** | **N** | **OR (95% CI)** | **N** | **OR (95% CI)** | **N** | **OR (95% CI)** |
| Sum Bisphenols | 1579 | 1.13 (0.65, 1.98) | 653 | 0.88 (0.39, 1.97) | 1479 | 1.18 (0.71, 1.96) | 648 | 0.92 (0.41, 2.06) |
| Bisphenol A | 2717 | 0.91 (0.63, 1.3) | 878 | 0.66 (0.39, 1.13) | 1952 | 1.06 (0.74, 1.52) | 1332 | 1.09 (0.68, 1.76) |
| Bisphenol F | 1636 | 1.39 (1, 1.94) | 695 | 1.31 (0.76, 2.24) | 1498 | 1.03 (0.69, 1.52) | 653 | 1.3 (0.68, 2.46) |
| Bisphenol S | 2042 | 1.01 (0.67, 1.52) | 666 | 1.08 (0.63, 1.85) | 1664 | 1.06 (0.71, 1.57) | 917 | 0.78 (0.45, 1.37) |
| 2,4-Dichlorophenol | 1530 | 0.9 (0.6, 1.36) |  |  | 844 | 0.86 (0.45, 1.66) | 762 | 1.15 (0.69, 1.92) |
| 2,5-Dichlorophenol | 1529 | 1.03 (0.79, 1.35) |  |  | 846 | 1.13 (0.75, 1.69) | 761 | 1.16 (0.81, 1.68) |
| Benzophenone-3 | 1921 | 0.96 (0.75, 1.23) | 249 | 0.84 (0.44, 1.62) | 1225 | 0.93 (0.69, 1.24) | 784 | 0.95 (0.64, 1.42) |
| Triclosan | 1913 | 1.08 (0.86, 1.35) |  |  | 1212 | 1.03 (0.79, 1.34) | 788 | 1.38 (0.96, 2) |
| Butyl paraben | 1575 | 0.96 (0.73, 1.27) | 51 | 0.02 (0, 148.78) | 880 | 0.86 (0.61, 1.22) | 774 | 1.27 (0.87, 1.87) |
| Ethyl paraben | 1258 | 0.99 (0.73, 1.34) | 78 | 0.34 (0.09, 1.25) | 881 | 1.03 (0.73, 1.46) | 432 | 1.26 (0.71, 2.24) |
| Methyl Paraben | 1629 | 0.85 (0.63, 1.16) | 80 | 0.91 (0.29, 2.88) | 886 | 0.85 (0.58, 1.24) | 798 | 1.01 (0.63, 1.63) |
| Propyl paraben | 1622 | 0.89 (0.7, 1.14) | 77 | 0.54 (0.21, 1.4) | 884 | 0.95 (0.71, 1.28) | 796 | 0.98 (0.67, 1.43) |

Outcome: Small for Gestational Age

|  | **Across Pregnancy** | | **Trimester 1** | | **Trimester 2** | | **Trimester 3** | |
| --- | --- | --- | --- | --- | --- | --- | --- | --- |
| **Phenol Name** | **N** | **OR (95% CI)** | **N** | **OR (95% CI)** | **N** | **OR (95% CI)** | **N** | **OR (95% CI)** |
| Sum Bisphenols | 1579 | 1.16 (0.72, 1.88) | 653 | 0.88 (0.49, 1.58) | 1479 | 0.95 (0.6, 1.5) | 648 | 1.68 (0.98, 2.88) |
| Bisphenol A | 2710 | 1.04 (0.77, 1.39) | 877 | 0.78 (0.52, 1.16) | 1951 | 1 (0.74, 1.35) | 1327 | 1.24 (0.89, 1.74) |
| Bisphenol F | 1636 | 1.21 (0.88, 1.65) | 695 | 1.21 (0.76, 1.93) | 1498 | 1.1 (0.78, 1.56) | 653 | 1.21 (0.77, 1.9) |
| Bisphenol S | 2042 | 0.98 (0.71, 1.36) | 666 | 1.08 (0.72, 1.6) | 1664 | 0.76 (0.55, 1.06) | 917 | **1.53 (1.08, 2.17)** |
| 2,4-Dichlorophenol | 1524 | 0.82 (0.6, 1.14) | 39 | 0.12 (0, 7.87) | 844 | 0.83 (0.47, 1.46) | 757 | 0.8 (0.54, 1.19) |
| 2,5-Dichlorophenol | 1523 | 0.87 (0.7, 1.09) | 38 | 0.33 (0.02, 6.33) | 846 | 1.09 (0.77, 1.53) | 756 | 0.83 (0.64, 1.08) |
| Benzophenone-3 | 1915 | **1.34 (1.1, 1.63)** |  |  | 1225 | 1.21 (0.94, 1.55) | 779 | **1.61 (1.21, 2.13)** |
| Triclosan | 1907 | 1.16 (0.97, 1.4) | 244 | 0.95 (0.6, 1.5) | 1212 | **1.26 (1.01, 1.57)** | 783 | 1.2 (0.91, 1.57) |
| Butyl paraben | 1569 | 1.11 (0.89, 1.37) |  |  | 880 | 1.14 (0.87, 1.5) | 769 | 1 (0.74, 1.36) |
| Ethyl paraben | 1258 | 1.07 (0.83, 1.36) | 78 | 1.77 (0.68, 4.61) | 881 | 0.75 (0.54, 1.05) | 432 | **1.68 (1.16, 2.43)** |
| Methyl Paraben | 1623 | 1.23 (0.96, 1.59) | 80 | 2.23 (0.73, 6.77) | 886 | 1.34 (0.96, 1.88) | 793 | 1.06 (0.75, 1.5) |
| Propyl paraben | 1616 | 1.08 (0.88, 1.32) |  |  | 884 | 1.23 (0.94, 1.6) | 791 | 0.96 (0.73, 1.27) |

Outcome: Large for Gestational Age

|  | **Across Pregnancy** | | **Trimester 1** | | **Trimester 2** | | **Trimester 3** | |
| --- | --- | --- | --- | --- | --- | --- | --- | --- |
| **Phenol Name** | **N** | **OR (95% CI)** | **N** | **OR (95% CI)** | **N** | **OR (95% CI)** | **N** | **OR (95% CI)** |
| Sum Bisphenols | 1579 | 1.17 (0.75, 1.84) | 653 | 1.33 (0.69, 2.57) | 1479 | 1.04 (0.69, 1.57) | 648 | 1.38 (0.7, 2.71) |
| Bisphenol A | 2710 | 1.17 (0.86, 1.58) | 877 | 1.15 (0.72, 1.82) | 1951 | 0.96 (0.71, 1.3) | 1327 | 1.46 (0.97, 2.2) |
| Bisphenol F | 1636 | 0.98 (0.73, 1.32) | 695 | 1.47 (0.91, 2.38) | 1498 | 0.99 (0.72, 1.36) | 653 | 1.25 (0.73, 2.16) |
| Bisphenol S | 2042 | 1.22 (0.87, 1.69) | 666 | 0.82 (0.51, 1.32) | 1664 | 1.18 (0.86, 1.64) | 917 | 0.84 (0.53, 1.34) |
| 2,4-Dichlorophenol | 1524 | 1.31 (0.96, 1.79) | 39 | 0.22 (0, 12.44) | 844 | **1.86 (1.17, 2.97)** | 757 | 1.28 (0.83, 1.98) |
| 2,5-Dichlorophenol | 1523 | 1.17 (0.94, 1.46) | 38 | 0.53 (0.03, 8.8) | 846 | 1.27 (0.93, 1.73) | 756 | 1.18 (0.86, 1.62) |
| Benzophenone-3 | 1915 | **0.77 (0.63, 0.93)** | 249 | 0.82 (0.45, 1.5) | 1225 | 0.87 (0.69, 1.08) | 779 | 0.79 (0.56, 1.12) |
| Triclosan | 1907 | 1.13 (0.94, 1.34) | 244 | 0.68 (0.41, 1.12) | 1212 | 1.12 (0.92, 1.37) | 783 | **1.58 (1.16, 2.14)** |
| Butyl paraben | 1569 | 0.91 (0.73, 1.13) | 51 | 2.46 (0.82, 7.44) | 880 | 0.8 (0.6, 1.05) | 769 | 1.06 (0.75, 1.5) |
| Ethyl paraben | 1258 | 0.97 (0.75, 1.25) | 78 | 0.66 (0.24, 1.84) | 881 | 0.98 (0.74, 1.3) | 432 | 1.54 (0.87, 2.72) |
| Methyl Paraben | 1623 | 0.83 (0.65, 1.06) | 80 | 2.2 (0.84, 5.78) | 886 | **0.68 (0.5, 0.91)** | 793 | 0.9 (0.6, 1.35) |
| Propyl paraben | 1616 | 0.9 (0.74, 1.1) | 77 | 2.07 (0.91, 4.73) | 884 | **0.74 (0.59, 0.93)** | 791 | 0.99 (0.72, 1.37) |

Outcome: Low Birthweight

|  | **Across Pregnancy** | | **Trimester 1** | | **Trimester 2** | | **Trimester 3** | |
| --- | --- | --- | --- | --- | --- | --- | --- | --- |
| **Phenol Name** | **N** | **OR (95% CI)** | **N** | **OR (95% CI)** | **N** | **OR (95% CI)** | **N** | **OR (95% CI)** |
| Sum Bisphenols | 1579 | 1.14 (0.6, 2.16) | 653 | 1.42 (0.67, 3.02) | 1479 | 0.99 (0.54, 1.81) | 648 | 1.43 (0.67, 3.06) |
| Bisphenol A | 2717 | 0.87 (0.57, 1.32) | 878 | 0.83 (0.49, 1.4) | 1952 | 1 (0.67, 1.51) | 1332 | 1.31 (0.77, 2.22) |
| Bisphenol F | 1636 | **1.62 (1.1, 2.39)** | 695 | **2.1 (1.24, 3.54)** | 1498 | 1.09 (0.68, 1.73) | 653 | 0.93 (0.48, 1.79) |
| Bisphenol S | 2042 | 0.87 (0.55, 1.39) | 666 | 1.32 (0.77, 2.25) | 1664 | 0.75 (0.48, 1.18) | 917 | **1.81 (1.08, 3.02)** |
| 2,4-Dichlorophenol | 1530 | **0.57 (0.33, 0.99)** | 39 | 0.52 (0.01, 19.24) | 844 | 0.69 (0.29, 1.66) | 762 | 0.8 (0.43, 1.49) |
| 2,5-Dichlorophenol | 1529 | **0.69 (0.49, 0.98)** | 38 | 0.22 (0.01, 7.47) | 846 | 0.96 (0.57, 1.61) | 761 | 0.75 (0.49, 1.14) |
| Benzophenone-3 | 1921 | 0.87 (0.64, 1.19) | 249 | 0.79 (0.41, 1.49) | 1225 | 0.88 (0.61, 1.28) | 784 | 0.95 (0.59, 1.52) |
| Triclosan | 1913 | 1.12 (0.84, 1.48) | 244 | 1.14 (0.68, 1.91) |  |  | 788 | 1.42 (0.92, 2.19) |
| Butyl paraben | 1575 | 1.16 (0.84, 1.62) |  |  | 880 | 1.04 (0.7, 1.56) | 774 | 1.2 (0.76, 1.9) |
| Ethyl paraben | 1258 | 1.12 (0.78, 1.61) | 78 | 1.22 (0.46, 3.28) | 881 | 0.75 (0.46, 1.24) |  |  |
| Methyl Paraben | 1629 | 0.98 (0.67, 1.45) | 80 | 1.4 (0.48, 4.09) | 886 | 0.94 (0.57, 1.55) | 798 | 1.3 (0.73, 2.3) |
| Propyl paraben | 1622 | 0.88 (0.64, 1.2) | 77 | 0.83 (0.37, 1.84) | 884 | 1 (0.67, 1.48) | 796 | 1 (0.64, 1.58) |

Outcome: Low Birthweight (Preterms only)

|  | **Across Pregnancy** | | **Trimester 1** | | **Trimester 2** | | **Trimester 3** | |
| --- | --- | --- | --- | --- | --- | --- | --- | --- |
| **Phenol Name** | **N** | **OR (95% CI)** | **N** | **OR (95% CI)** | **N** | **OR (95% CI)** | **N** | **OR (95% CI)** |
| Sum Bisphenols | 116 | 0.64 (0.19, 2.1) | 39 | 0.78 (0.08, 7.74) | 110 | 0.6 (0.2, 1.81) | 37 | 5.22 (0.25, 107.98) |
| Bisphenol A | 192 | 0.47 (0.22, 1.03) | 65 | 0.83 (0.23, 2.97) | 143 | 0.69 (0.33, 1.48) | 81 | 1.95 (0.53, 7.24) |
| Bisphenol F | 128 | 1.12 (0.52, 2.42) | 47 | 1.51 (0.33, 6.92) | 114 | 0.74 (0.3, 1.84) | 37 | 1.12 (0.13, 9.77) |
| Bisphenol S | 132 | 0.88 (0.35, 2.21) | 40 | 0.87 (0.18, 4.17) | 115 | 0.68 (0.3, 1.56) | 47 | 2.08 (0.38, 11.34) |
| 2,4-Dichlorophenol | 99 | 0.37 (0.11, 1.29) |  |  | 58 | 0.21 (0.02, 1.78) | 47 | 0.32 (0.04, 2.65) |
| 2,5-Dichlorophenol | 99 | **0.38 (0.15, 0.98)** |  |  | 58 | 0.39 (0.08, 1.91) | 47 | 0.17 (0.02, 1.49) |
| Benzophenone-3 | 124 | 0.98 (0.5, 1.91) | 19 | 0 (0, 5.94) |  |  | 47 | 1.02 (0.22, 4.79) |
| Triclosan | 124 | 0.77 (0.44, 1.36) |  |  |  |  | 48 | 1.58 (0.52, 4.79) |
| Butyl paraben | 105 | 1.18 (0.54, 2.58) |  |  | 62 | 2.16 (0.47, 9.89) | 47 | 1.36 (0.5, 3.7) |
| Ethyl paraben | 82 | 1.32 (0.59, 2.98) |  |  | 62 | 1.01 (0.38, 2.74) |  |  |
| Methyl Paraben | 110 | 0.69 (0.3, 1.58) |  |  | 63 | 0.88 (0.25, 3.08) | 48 | 2.45 (0.49, 12.27) |
| Propyl paraben | 110 | **0.47 (0.24, 0.94)** |  |  | 63 | 0.6 (0.21, 1.67) | 48 | 1.19 (0.41, 3.48) |

Outcome: Low Birthweight (Terms only)

|  | **Across Pregnancy** | | **Trimester 1** | | **Trimester 2** | | **Trimester 3** | |
| --- | --- | --- | --- | --- | --- | --- | --- | --- |
| **Phenol Name** | **N** | **OR (95% CI)** | **N** | **OR (95% CI)** | **N** | **OR (95% CI)** | **N** | **OR (95% CI)** |
| Sum Bisphenols | 1463 | 1.66 (0.64, 4.33) | 614 | 2.2 (0.77, 6.25) | 1369 | 1.11 (0.43, 2.83) | 611 | 1.19 (0.41, 3.51) |
| Bisphenol A | 2525 | 1.25 (0.67, 2.32) | 813 | 1.18 (0.55, 2.53) | 1809 | 1.25 (0.66, 2.37) | 1251 | 1.1 (0.56, 2.18) |
| Bisphenol F | 1508 | **2.15 (1.21, 3.85)** | 648 | **2.56 (1.17, 5.59)** | 1384 | 1.52 (0.77, 3) | 616 | 0.88 (0.34, 2.26) |
| Bisphenol S | 1910 | 0.93 (0.47, 1.81) | 626 | 1.72 (0.79, 3.72) | 1549 | 0.7 (0.36, 1.36) | 870 | **1.98 (1.02, 3.86)** |
| 2,4-Dichlorophenol | 1431 | 0.52 (0.25, 1.07) |  |  | 786 | 0.5 (0.12, 2.04) | 715 | 0.65 (0.29, 1.44) |
| 2,5-Dichlorophenol | 1430 | 0.7 (0.44, 1.1) | 36 | 0.09 (0, 37.76) | 788 | 1.03 (0.49, 2.15) | 714 | 0.64 (0.37, 1.1) |
| Benzophenone-3 | 1797 | 0.94 (0.62, 1.44) |  |  | 1142 | 1 (0.58, 1.73) | 737 | 1.14 (0.64, 2.02) |
| Triclosan | 1789 | 1.2 (0.81, 1.77) |  |  |  |  | 740 | 1.32 (0.77, 2.26) |
| Butyl paraben | 1470 | 1.37 (0.91, 2.06) |  |  | 818 | 1.16 (0.66, 2.03) | 727 | 1.2 (0.68, 2.14) |
| Ethyl paraben | 1176 | 1.12 (0.67, 1.86) | 71 | 2.32 (0.52, 10.37) | 819 | 0.45 (0.2, 1.03) | 412 | **2.47 (1.22, 5)** |
| Methyl Paraben | 1519 | 1.26 (0.74, 2.12) | 73 | 1.43 (0.29, 6.92) | 823 | 1.34 (0.62, 2.86) | 750 | 1.52 (0.75, 3.09) |
| Propyl paraben | 1512 | 1.16 (0.76, 1.77) | 70 | 1.22 (0.37, 4.03) | 821 | 1.43 (0.78, 2.62) | 748 | 1.11 (0.64, 1.94) |

**Supplement Table 6: Nominal Logistic Analysis for Adjusted Associations of Phenol Exposures with 4 Category Gestational Age**Models adjusted for maternal race/ethnicity, parity, and education, and child sex.

Reference = Term Pregnancies

| **Exposure** | **N** | **Preterm [OR (95% CI)]** | **Early Term [OR (95% CI)]** | **Late Term [OR (95% CI)]** |
| --- | --- | --- | --- | --- |
| Sum Bisphenols | 1667 | 1.18 (0.68, 2.05) | 0.86 (0.62, 1.19) | 0.97 (0.6, 1.56) |
| Bisphenol A | 2807 | 0.92 (0.64, 1.32) | 0.96 (0.78, 1.19) | 1.1 (0.82, 1.47) |
| Bisphenol F | 1724 | 1.35 (0.97, 1.89) | 0.9 (0.73, 1.13) | 1.23 (0.91, 1.66) |
| Bisphenol S | 2130 | 1.02 (0.67, 1.55) | 1.06 (0.84, 1.34) | 0.54 (0.39, 0.76) |
| 2,4-Dichlorophenol | 1573 | 0.91 (0.6, 1.37) | 0.97 (0.77, 1.22) | 1.32 (0.98, 1.77) |
| 2,5-Dichlorophenol | 1572 | 1.09 (0.83, 1.44) | 1 (0.85, 1.17) | 1.42 (1.15, 1.75) |
| Benzophenone-3 | 1965 | 0.94 (0.73, 1.21) | 0.9 (0.78, 1.03) | 1 (0.82, 1.21) |
| Triclosan | 2096 | 1.08 (0.87, 1.35) | 1.02 (0.89, 1.15) | 1.01 (0.84, 1.2) |
| Butyl paraben | 1723 | 0.91 (0.7, 1.19) | 0.82 (0.71, 0.96) | 1.09 (0.89, 1.33) |
| Ethyl paraben | 1439 | 1 (0.75, 1.34) | 0.9 (0.76, 1.07) | 0.98 (0.77, 1.26) |
| Methyl Paraben | 1811 | 0.81 (0.6, 1.09) | 0.82 (0.69, 0.97) | 0.89 (0.71, 1.13) |
| Propyl paraben | 1799 | 0.87 (0.68, 1.11) | 0.83 (0.72, 0.95) | 0.97 (0.8, 1.17) |

**Supplement Figure 1: Depicting Correlations Among Measured Phenols**

**Supplement Figure 2a: Leave-One-Out Analysis: Birth Gestational Age**

**Supplement Figure 2b: Leave-One-Out Analysis: Preterm Birth**
